# Phylogeny of Cas9 determines functional exchangeability of dual-RNA and Cas9 among orthologous type II CRISPR-Cas systems

**DOI:** 10.1093/nar/gkt1074

**Published:** 2013-11-21

**Authors:** Ines Fonfara, Anaïs Le Rhun, Krzysztof Chylinski, Kira S. Makarova, Anne-Laure Lécrivain, Janek Bzdrenga, Eugene V. Koonin, Emmanuelle Charpentier

**Affiliations:** ^1^The Laboratory for Molecular Infection Medicine Sweden (MIMS), Umeå Centre for Microbial Research (UCMR), Department of Molecular Biology, Umeå University, Umeå S-90187, Sweden, ^2^Helmholtz Centre for Infection Research, Department of Regulation in Infection Biology, Braunschweig D-38124, Germany, ^3^Deptartment of Biochemistry and Cell Biology, Max F. Perutz Laboratories, University of Vienna, Vienna A-1030, Austria, ^4^National Center for Biotechnology Information, National Library of Medicine, National Institutes of Health, Bethesda, MD 20894, USA and ^5^Hannover Medical School, Hannover D-30625, Germany

## Abstract

The CRISPR-Cas-derived RNA-guided Cas9 endonuclease is the key element of an emerging promising technology for genome engineering in a broad range of cells and organisms. The DNA-targeting mechanism of the type II CRISPR-Cas system involves maturation of tracrRNA:crRNA duplex (dual-RNA), which directs Cas9 to cleave invading DNA in a sequence-specific manner, dependent on the presence of a Protospacer Adjacent Motif (PAM) on the target. We show that evolution of dual-RNA and Cas9 in bacteria produced remarkable sequence diversity. We selected eight representatives of phylogenetically defined type II CRISPR-Cas groups to analyze possible coevolution of Cas9 and dual-RNA. We demonstrate that these two components are interchangeable only between closely related type II systems when the PAM sequence is adjusted to the investigated Cas9 protein. Comparison of the taxonomy of bacterial species that harbor type II CRISPR-Cas systems with the Cas9 phylogeny corroborates horizontal transfer of the CRISPR-Cas loci. The reported collection of dual-RNA:Cas9 with associated PAMs expands the possibilities for multiplex genome editing and could provide means to improve the specificity of the RNA-programmable Cas9 tool.

## INTRODUCTION

Editing genomes using the RNA-guided DNA targeting principle of CRISPR-Cas (Clustered Regularly Interspaced Short Palindromic Repeats-CRISPR associated proteins) immunity has been exploited widely over the past few months (1–13). The main advantage provided by the bacterial type II CRISPR-Cas system lies in the minimal requirement for programmable DNA interference: an endonuclease, Cas9, guided by a customizable dual-RNA structure (14). As initially demonstrated in the original type II system of *Streptococcus pyogenes*, *trans*-activating CRISPR RNA (tracrRNA) (15,16) binds to the invariable repeats of precursor CRISPR RNA (pre-crRNA) forming a dual-RNA (14–17) that is essential for both RNA comaturation by RNase III in the presence of Cas9 (15–17), and invading DNA cleavage by Cas9 (14,17–19). As demonstrated in *Streptococcus*, Cas9 guided by the duplex formed between mature activating tracrRNA and targeting crRNA (14–16) introduces site-specific double-stranded DNA (dsDNA) breaks in the invading cognate DNA (14,17–19). Cas9 is a multi-domain enzyme (14,20,21) that uses an HNH nuclease domain to cleave the target strand (defined as complementary to the spacer sequence of crRNA) and a RuvC-like domain to cleave the non-target strand (14,22,23), enabling the conversion of the dsDNA cleaving Cas9 into a nickase by selective motif inactivation (2,8,14,24,25). DNA cleavage specificity is determined by two parameters: the variable, spacer-derived sequence of crRNA targeting the protospacer sequence (a protospacer is defined as the sequence on the DNA target that is complementary to the spacer of crRNA) and a short sequence, the Protospacer Adjacent Motif (PAM), located immediately downstream of the protospacer on the non-target DNA strand (14,18,23,26–28).

Recent studies have demonstrated that RNA-guided Cas9 can be employed as an efficient genome editing tool in human cells (1,2,8,11), mice (9,10), zebrafish (6), drosophila (5), worms (4), plants (12,13), yeast (3) and bacteria (7). The system is versatile, enabling multiplex genome engineering by programming Cas9 to edit several sites in a genome simultaneously by simply using multiple guide RNAs (2,7,8,10). The easy conversion of Cas9 into a nickase was shown to facilitate homology-directed repair in mammalian genomes with reduced mutagenic activity (2,8,24,25). In addition, the DNA-binding activity of a Cas9 catalytic inactive mutant has been exploited to engineer RNA-programmable transcriptional silencing and activating devices (29,30).

At present, RNA-guided Cas9 from *S. pyogenes*, S. thermophilus** and *Neisseria meningitidis* have been developed into tools for genome manipulation (1–13,24,25,31–34). Here, we explore the possibilities of expanding the RNA-programmable Cas9 toolbox to additional orthologous systems. We investigated the diversity and interchangeability of dual-RNA:Cas9 in eight representatives of phylogenetically defined type II CRISPR-Cas groups. The results of this work not only introduce a wider range of Cas9 enzymes, dual-RNA structures and associated specific PAMs but also enlighten the evolutionary aspects of type II CRISPR-Cas systems, including coevolution and horizontal transfer of the system components.

## MATERIALS AND METHODS

### Bacterial strains and culture conditions

Supplementary Table S1 lists bacterial strains used in this study. *Streptococcus pyogenes*, *Streptococcus mutans*, *Campylobacter jejuni*, *N. meningitidis*, *Escherichia coli* and *Francisella novicida* were grown as previously described (15,16). Brain Heart Infusion (BHI, Becton Dickinson) agar and BHI broth medium supplemented with 1% glucose and 1% lactose were used to culture *S. thermophilus* at 42°C in a 5% CO_2_ environment (16). *Pasteurella multocida* and *Staphylococcus aureus* were grown at 37°C on BHI agar plates and in BHI broth with shaking. Cell growth was monitored by measuring the optical density of cultures at 620 nm (OD_620_) using a microplate reader (BioTek PowerWave).

### Bacterial transformation

*Escherichia coli* was transformed with plasmid DNA according to standard protocols (35). Transformation of *S. pyogenes* was performed as previously described (36) with some modifications. *Streptococcus pyogenes* pre-cultures were diluted 1:100 in fresh THY medium and grown at 37°C, 5% CO_2_ until OD_620_ reached 0.3. Glycine was added to the medium to 10% final concentration and growth was maintained for an additional hour. Cells were spun down at 4°C at 2500 × g and washed three times with electroporation buffer (5 mM KH_2_PO_4_, 0.4 M D-sorbitol, 10% glycerol, pH 4.5), finally suspended in the same buffer and equalized to the same OD_620_. For electroporation, 1 µg of plasmid was incubated with the competent cells on ice for 10 min. The conditions were 25 µF, 600 Ω and 1.5 V using 1 mm electroporation cuvettes (Biorad). After a regeneration time of 3 h, bacteria were spread on agar medium supplemented with kanamycin (300 µg/ml). Transformation assays were performed at least three times independently with technical triplicates. The efficiencies were calculated as colony-forming units (CFU) per µg of plasmid DNA. Positive and negative control transformations were done with backbone plasmid pEC85 and sterile H_2_O, respectively.

### DNA manipulations

DNA manipulations including DNA preparation (QIAprep Spin MiniPrep Kit, Qiagen), polymerase chain reaction (PCR) (Phusion*®* High-Fidelity DNA Polymerase*,* Finnzyme), DNA digestion (restriction enzymes, Fermentas), DNA ligation (T4 DNA ligase, Fermentas), DNA purification (QIAquick PCR Purification Kit, Qiagen) and agarose gel electrophoresis were performed according to the standard techniques or manufacturers’ protocols with some modifications (35). Site-directed mutagenesis was done using QuikChange II XL kit (Stratagene) or PCR-based mutagenesis (37). Synthetic oligonucleotides (Sigma-Aldrich and Biomers) and plasmids used and generated in this study are listed in Supplementary Table S1. The integrity of all constructed plasmids was verified by enzymatic digestion and sequencing at LGC Genomics.

### Construction of plasmids for complementation studies in *S. pyogenes*

The backbone shuttle vector pEC85 was used for complementation study (38,39). The RNase-III encoding genes (*rnc* genes) of *S. pyogenes*, *S. mutans*, *S. thermophilus*, *C. jejuni*, *N. meningitidis*, *P. multocida*, *F. novicida*, *E. coli* and *S. aureus*, and the genes encoding truncated and inactive RNase III variants (truncated and inactive (D51A) *rnc* mutants) of *S. pyogenes* were cloned in pEC483 (pEC85 containing the native promoter of *S. pyogenes rnc*) using NcoI and EcoRI restriction sites (Supplementary Table S1, Supplementary Figure S6). The orthologous and mutant *cas9* genes were cloned in pEC342 (pEC85 containing a sequence encoding tracrRNA-171 nt (16) and the native promoter of the *S. pyogenes cas* operon) using SalI and SmaI restriction sites (Supplementary Table S1). Note that in a previous study, we observed low abundance of tracrRNA in the *cas9* deletion mutant. For this reason, plasmids used in *cas9* complementation studies were designed to encode tracrRNA in addition to *cas9* (16). The generated *rnc* and *cas9* recombinant plasmids were introduced in *S. pyogenes* Δ*rnc* and Δ*cas9* deletion strains, respectively (Supplementary Table S1). Plasmid integrity in all complemented strains was checked by plasmid DNA extraction and digestion.

### Construction of plasmids for transformation studies in *S. pyogenes*

Plasmid pEC85 was used as backbone vector for transformation studies. A DNA fragment containing WT *speM* protospacer sequence was cloned in the PstI site of plasmids containing coding sequences of WT or mutated *cas9* from *S. pyogenes* (Supplementary Table S1).

### Construction of plasmids for protein purification

The overexpression vector pET16b (Novagen) was modified by inserting three additional restriction sites (SalI, SacI, NotI) into the NdeI restriction site, generating pEC621. The genes coding for the orthologous Cas9 proteins were PCR amplified from genomic DNA of the corresponding strains using primers containing a SalI and a NotI restriction site (Supplementary Table S1). The *S. pyogenes cas9* mutant genes were PCR amplified from the complementation plasmids mentioned above. All orthologous and mutant *cas9* genes were cloned into the SalI and NotI sites of pEC621.

### Construction of substrate plasmids for *in vitro* cleavage assays

Plasmid pEC287 that contains the *speM* protospacer sequence was used as a vector to construct all substrate plasmids. The PAM sequence located in 3′ just next to the crRNA-targeted sequence of the *speM* protospacer (GGG on this plasmid) was modified by PCR-mediated site-directed mutagenesis (37) using one standard oligonucleotide (OLEC3140 or OLEC3194) that either introduced or removed a XbaI restriction site for screening purposes, and a second mutagenic oligonucleotide to exchange the protospacer adjacent sequence (Supplementary Table S1).

### RNA preparation

Total RNA from *S. pyogenes* SF370 WT, deletion mutants and complemented strains was prepared from culture samples collected at the mid-logarithmic phase of growth using TRIzol (Sigma-Aldrich). The total RNA samples were treated with DNase I (Fermentas) according to the manufacturer’s instructions. The concentration of RNA in each sample was measured using NanoDrop.

### Northern blot analysis

Northern blot analysis was carried out essentially as described previously (40–42). Total RNA was separated on 10% polyacrylamide 8 M urea gels and further processed for blotting on nylon membranes (Hybond™ N+, GE healthcare; Trans-Blot® SD semi-dry transfer apparatus, Biorad; 1X TBE, 2 h at 10 V/cm), chemical cross-linking with EDC (1-Ethyl-3-(3-dimethylaminopropyl) carbodiimide hydrochloride) (41) and prehybridization (Rapid-hyb buffer, GE healthcare; 1 h at 42°C). Oligonucleotide probes (40 pmol) were labeled with ^32^P (20 μCi) using the T4-polynucleotide kinase (10 U, Fermentas) and purified using G-25 columns (GE Healthcare) prior use. Visualization of the radioactive signal was done using a phosphorimager. 5S rRNA served as loading control.

### Protein purification

*Escherichia coli* Rosetta2(DE3) and *E. coli* NiCo21(DE3) (New England Biolabs) were transformed with overexpression plasmids coding for *S. pyogenes* WT and mutant or orthologous Cas9, respectively. Cells were grown at 37°C to reach an OD_600_ of 0.7–0.8, protein expression was induced by adding IPTG to a final concentration of 0.5 mM and cultures were further grown at 13°C overnight. The cells were harvested by centrifugation and the pellet was resuspended in lysis buffer (20 mM HEPES, pH 7.5, 500 mM KCl [1 M for S. thermophilus* Cas9], 0.1% Triton X-100, 25 mM imidazole) and lysed by sonication. The lysate was cleared by centrifugation (>20 000 × g) and incubated with Ni-NTA (Qiagen) for 1 h at 4°C. After washing the Ni-NTA with lysis buffer and wash buffer (20 mM HEPES, pH 7.5, 300 mM KCl, 0.1% Triton X-100, 25 mM imidazole), the recombinant protein was eluted with elution buffer (20 mM HEPES, pH 7.5, 150 mM KCl, 0.1 mM DTT, 250 mM imidazole, 1 mM (EDTA)) and the fractions were analyzed by sodium dodecyl sulphate-polyacrylamide gel electrophoresis (SDS-PAGE). In the case of *S. pyogenes* Cas9 WT and mutants, the protein containing eluates were pooled and further purified via HiTrap SP FF (GE Healthcare) cation-exchange chromatography. Briefly, the protein was loaded on the column equilibrated with buffer A (20 mM HEPES pH 7.5, 100 mM KCl) using an FPLC system (Äkta, GE Healthcare). Cas9 was eluted with a gradient of buffer B (20 mM HEPES pH 7.5, 1 M KCl) over 12 ml. 1 ml fractions were collected and analyzed by (SDS–PAGE). The protein containing fractions were pooled and dialyzed overnight (20 mM HEPES, pH 7.5, 150 mM KCl, 50% glycerol). For Cas9 orthologs, the eluates from Ni-NTA purification were checked for purity by SDS–PAGE. In case of contaminants, a second purification over chitin beads was performed as described in the manual for NiCo21(DE3) cells from New England Biolabs. Briefly, 1 ml chitin beads (New England Biolabs) equilibrated with buffer A was incubated with the Ni^2+^-IMAC eluates for 1 h at 4°C. Afterwards, the beads were added onto a column and the Cas9 containing flowthroughs were collected and again checked for purity by SDS–PAGE (Supplementary Figure S1). The purified proteins were dialyzed overnight. The protein concentration was calculated by measuring the OD_280_ using the extinction coefficient. The detailed characteristics of purified proteins are summarized in Supplementary Figure S1A.

### *In vitro* transcription

RNA for *in vitro* DNA cleavage assays was generated by *in vitro* transcription using the AmpliScribe™ T7-*Flash*™ Transcription Kit (Epicentre) according to the manufacturer’s instructions. PCR products or synthetic oligonucleotides used as templates are listed in Supplementary Table S1. The synthesized tracrRNA and repeat region of crRNA from each bacterial species correspond to the mature forms of RNAs as determined by deep RNA sequencing (15) or bioinformatics predictions. The spacer region of all crRNAs used in this study targets the *speM* protospacer (encoding superantigen; targeted by spacer 2 of *S. pyogenes* SF370 CRISPR array, Spyo1h_002 (16)). Transcribed RNAs were precipitated and further purified from 10% polyacrylamide 8 M urea denaturing gel. The RNA concentration was determined by measuring the OD_260_ and the molarity was calculated. Equimolar amounts of crRNA and tracrRNA were mixed in 5X RNA annealing buffer (1 M NaCl, 100 mM HEPES, pH 7.5), heated up to 95°C for 5 min and slowly cooled to room temperature before use.

### *In vitro* DNA cleavage assays

For the cleavage assays using Cas9 mutant proteins, 25 nM of Cas9 were incubated with equimolar amounts of prehybridized *S. pyogenes* dual-RNA in cleavage buffer (20 mM HEPES, pH 7.5, 150 mM KCl, 10 mM MgCl_2_, 0.5 mM DTT, 0.1 mM EDTA) for 15 min at 37°C. Plasmid DNA (5 nM) containing *speM* (NGG PAM) was added and further incubated for 1 h at 37°C. The reaction was stopped by addition of 5X loading buffer (250 mM EDTA, 30% glycerol, 1.2% SDS, 0.1% (w/v) bromophenol blue) and analyzed by 1% agarose gel electrophoresis in 1X TAE. Cleavage products were visualized by ethidium bromide staining. All other cleavage assays were carried out using the same conditions with the following modifications: KGB (43) (100 mM potassium glutamate, 25 mM Tris/acetate, pH 7.5, 10 mM Mg-acetate, 0.5 mM 2-mercaptoethanol, 10 µg/ml bovine serum albumin) was used as cleavage buffer and different concentrations of dual-RNA:Cas9 complex were analyzed. The concentration of plasmid DNA was kept constant in all experiments, i.e. 5 nM.

### Search for PAM motifs

Spacer sequences of the selected bacterial species were extracted from the CRISPRdatabase (http://crispr.u-psud.fr/crispr/) and used to find cognate protospacer candidates using megaBLAST (http://blast.ncbi.nih.gov/Blast). Protospacer candidates were defined as containing a sequence with ≥90% similarity to the crRNA spacer sequence and originating from phage, plasmid or genomic DNA related to the bacterial species of the targeting CRISPR-Cas. For the investigated CRISPR-Cas loci, the orientation of transcription was determined previously by RNA sequencing or northern blot analysis (15,16). It was also shown before that in type II CRISPR-Cas, the PAM sequence is located in 3′ of the protospacer, juxtaposed to the sequence targeted by cognate crRNA on the non-target strand (14,18,23,44). To identify possible PAMs in each bacterial species, 10 nt sequences on the non-target strand directly downstream of each protospacer sequence were aligned. A logo plot (http://weblogo.berkeley.edu/) showing the most abundant nucleotides was created and PAM sequences were predicted. In the cases of CRISPR-Cas loci for which no suitable protospacer sequences could be identified (*S. mutans* UA159, *C. jejuni* NCTC 11168, *P. multocida* Pm70, *F. novicida* U112), closely related strains of the same species were selected (Supplementary Table S2). The spacer contents of the type II CRISPR arrays in selected strains were analyzed (http://crispr.u-psud.fr/Server/). The spacer sequences were then used to select cognate protospacer sequences as described above.

### Protein sequence analysis

Position-Specific Iterated (PSI)-BLAST program (45) was used to retrieve orthologs of the Cas9 family in the NCBI nr database. Sequences shorter than 800 amino acids were discarded. The BLASTClust program (46) set up with a length coverage cutoff of 0.8 and a score coverage threshold (bit score divided by alignment length) of 0.8 was used to cluster the remaining sequences (Supplementary Table S2). This procedure produced 82 clusters. In the case of sequences reported in this study, one or several representatives from each cluster were selected and aligned using the MUSCLE program (47) with default parameters, followed by a manual correction on the basis of local alignments obtained using PSI-BLAST (45) and HHpred programs (48). The confidently aligned blocks (Supplementary Figure S2) with 285 informative positions were used for maximum likelihood tree reconstruction using the FastTree program (49) with the default parameters: JTT evolutionary model, discrete gamma model with 20 rate categories. The same program was used to calculate the bootstrap values. Cas1 sequences were selected from the corresponding *cas* operons (Supplementary Table S2). A few incomplete sequences were substituted by other Cas1 sequences from the same Cas9 cluster (Supplementary Table S2). Several Cas1 proteins from subtypes I-A, B, C and E were included as an outgroup. Cas1 sequences were aligned using the same approach described above and 252 informative positions (Supplementary Figure S3) were used for maximum likelihood tree reconstruction using the FastTree program. RNase III multiple sequence alignment was prepared using the MUSCLE program.

### RNA sequence and structure analysis

RNA duplex secondary structures were predicted using RNAcofold of the Vienna RNA package (50,51) and RNAhybrid (http://bibiserv.techfak.uni-bielefeld.de/rnahybrid/). The structure predictions were then visualized using VARNA (52).

## RESULTS

### Diversity of Cas9 orthologs

**Figure 1.**
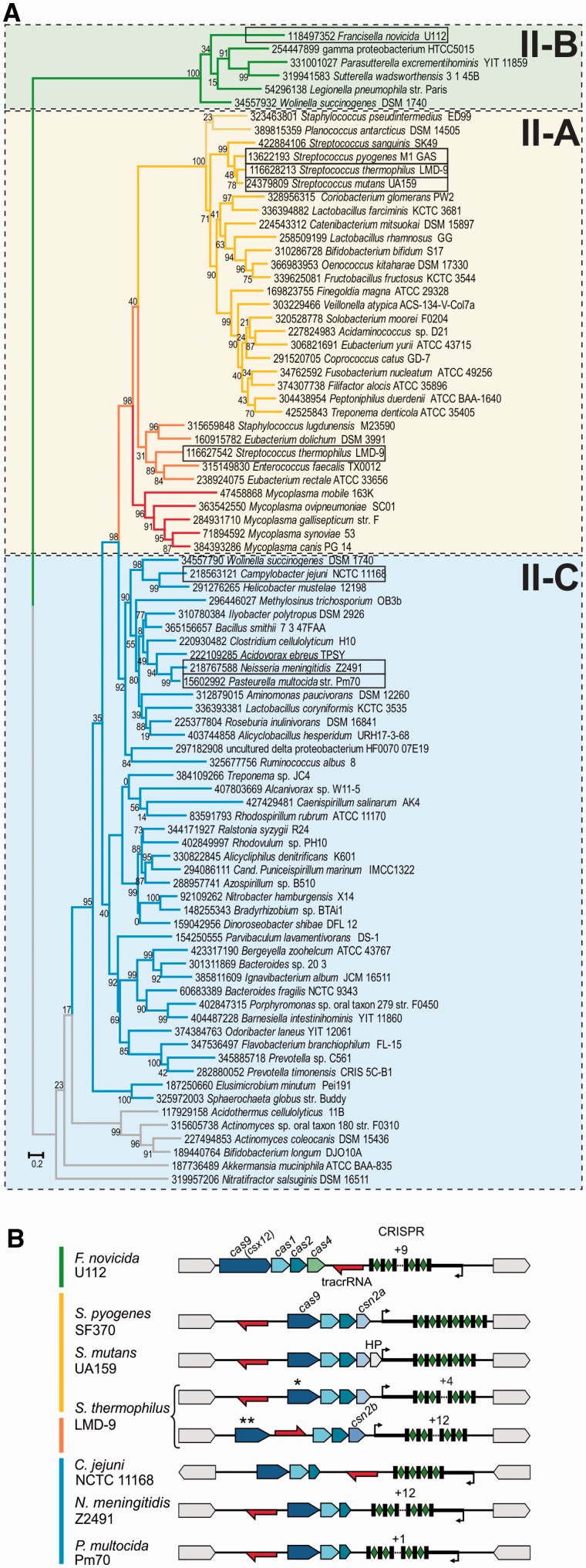
Phylogeny of representative Cas9 orthologs and schematic representation of selected bacterial type II CRISPR-Cas systems. (**A**) Phylogenetic tree of Cas9 reconstructed from selected, informative positions of representative Cas9 orthologs multiple sequence alignment is shown (Supplementary Figure S2 and Supplementary Table S2). The Cas9 orthologs of the subtypes classified as II-A, II-B and II-C are highlighted with shaded boxes. The colored branches group distinct proteins of closely related loci with similar locus architecture (15). Each protein is represented by the GenInfo (GI) identifier followed by the bacterial strain name. The bootstrap values are given for each node (see ‘Materials and Methods’ section). Note that the monophyletic clusters of subtypes II-A and II-B are supported by high bootstrap values. The scale bar for the branch length is given as the estimated number of amino acid substitution per site. (**B**) Genetic loci of type II (Nmeni/CASS4) CRISPR-Cas in *Streptococcus pyogenes* SF370, *Streptococcus mutans* UA159, *Streptococcus thermophilus* LMD-9 *(CRISPR3), **(CRISPR1), *Campylobacter jejuni* NCTC 11168, *Neisseria meningitidis* Z2491, *Pasteurella multocida* Pm70 and *Francisella novicida* U112. Red arrow, transcription direction of tracrRNA; blue arrows, *cas* genes; black rectangles, CRISPR repeats; green diamonds, spacers; thick black line, leader sequence; black arrow, putative pre-crRNA promoter; HP, Hypothetical Protein. The colored bars represented on the left correspond to Cas9 tree branches colors. The transcription direction and putative leader position of *C. jejuni* and *N. meningitidis* pre-crRNAs were derived from previously published RNA sequencing data (15). The CRISPR-Cas locus architecture of *P. multocida* was predicted based on its close similarity to that of *N. meningitidis* and further confirmed by bioinformatics prediction of tracrRNA based on a strongly predicted promoter and a transcriptional terminator as described in (15). Type II CRISPR-Cas loci can differ in the *cas* gene composition, mostly with *cas9*, *cas1* and *cas2* being the minimal set of genes (type II-C, blue), sometimes accompanied with a fourth gene *csn2a*/*b* (type II-A, yellow and orange) or *cas4* (type II-B, green). The CRISPR array can be transcribed in the same (type II-A, yellow and orange) or in the opposite (types II-B and C, blue and green) direction of the *cas* operon. The location of tracrRNA and the direction of its transcription differ within the groups (compare type II-A of S. thermophilus** with type II-A from the other species indicated here (yellow) and compare type II-C of *C. jejuni* with type II-C of *N. meningitidis* and *P. multocida* (blue)).

To investigate the evolution and diversity of dual-RNA:Cas9 systems, we subjected publicly available genomes to multiple rounds of BLAST search using previously retrieved Cas9 sequences as queries (15). Cas9 orthologs were identified in 653 bacterial strains representing 347 species (Supplementary Table S2). After removing incomplete or highly similar sequences, we selected 83 diverse, representative Cas9 orthologs for multiple sequence alignment and phylogenetic tree reconstruction (Figure 1A, Supplementary Table S2, Supplementary Figures S2 and S4, see ‘Materials and Methods’ section). The Cas9 tree topology largely agrees with the phylogeny of the corresponding Cas1 proteins (Supplementary Table S2, Supplementary Figures S3 and S4) and fully supports the previously described classification of type II CRISPR-Cas into three subtypes, II-A (specified by *csn2*), II-B (characterized by long and most diverged *cas9* variants (formerly *csx12*) and *cas4*) and II-C (three-*cas* gene operon) (15).

Analysis of the composition of *cas* genes, transcription direction of the CRISPR arrays with respect to that of the *cas* operon, and location and orientation of tracrRNAs resulted in the division of subtypes into groups with distinct locus characteristics, especially within the subtype II-A (Figure 1, clusters marked with different colors) (15). We selected Cas9 enzymes representative of the major type II groups. Cas9 orthologs of S. pyogenes, S. thermophilus* (CRISPR3) and *S. mutans* were chosen for type II-A systems associated with shorter, ∼220 amino acid Csn2 variants (Csn2a). Cas9 of S. thermophilus** (CRISPR1) represents a distinct group of type II-A sequences associated with longer, ∼350 amino acid version of Csn2 orthologs (Csn2b). Cas9 of *F. novicida* was selected for type II-B. The closely related Cas9 orthologs of *P. multocida* and *N. meningitidis* and the distinct, short Cas9 of *C. jejuni* were chosen for type II-C (Figure 1B). Expression of associated tracrRNAs and crRNAs in *S. pyogenes*, *S. mutans*, *F. novicida, N. meningitidis* and *C. jejuni* was already validated by deep RNA sequencing (15,16). The RNAs in *S. thermophilus* and *P. multocida* were predicted bioinformatically based on the sequences from related species within the same type II group. Figure 1B shows the organization of the eight selected type II CRISPR-Cas loci and highlights our previous findings demonstrating that the type II loci architectures are highly variable among subtypes, yet conserved within each group (15). These variations are in good agreement with the clustering derived from the Cas9 and Cas1 phylogenetic trees (Figure 1A, Supplementary Figure S4).

### Bacterial RNases III are interchangeable in dual-RNA maturation

**Figure 2.**
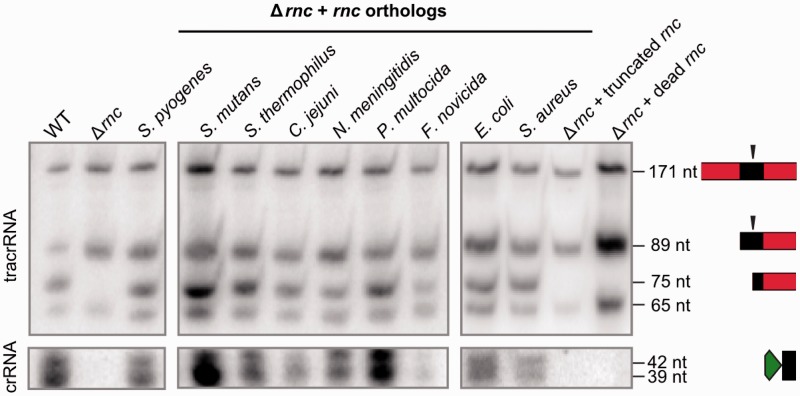
RNase III is a general executioner of tracrRNA:pre-crRNA processing in type II CRISPR-Cas. Northern blot analysis of total RNA from *S. pyogenes* WT, Δ*rnc* and Δ*rnc* complemented with *rnc* orthologs or mutants (truncated *rnc* and inactivated (dead) (D51A) *rnc*) probed for tracrRNA (top) and crRNA repeat (bottom). RNA sizes in nucleotide and schematic representations of tracrRNA (red-black) and crRNA (green-black) are indicated on the right (16). The vertical black arrows indicate the processing sites. tracrRNA-171 nt and tracrRNA-89 nt forms correspond to primary tracrRNA transcripts. The presence of tracrRNA-75 nt and crRNA 39-42 nt forms indicates tracrRNA and pre-crRNA co-processing. *S. pyogenes* tracrRNA and pre-crRNA are coprocessed by all analyzed RNase III orthologs. The truncated version and catalytic inactive mutant of *S. pyogenes* RNase III are both deficient in tracrRNA:pre-crRNA processing.

As described in *S. pyogenes* and *S. thermophilus*, RNase III plays an essential role in the biogenesis of dual-RNA:Cas9 systems by coprocessing tracrRNA and pre-crRNA at the level of antirepeat:repeat duplexes (16,17). We analyzed the interchangeability of *S. pyogenes* RNase III with RNases III from selected bacterial species in the coprocessing of *S. pyogenes* tracrRNA:pre-crRNA, including strains that lack type II CRISPR-Cas (*S. aureus* COL, *E. coli* TOP10). Northern blot analysis shows that all RNases III studied here can coprocess the RNA duplex (Figure 2, Supplementary Figure S5), indicating that there is no species-specificity for tracrRNA:pre-crRNA cleavage by RNase III. Multiple sequence alignment of RNase III orthologs demonstrates conservation of the catalytic aspartate residue and the dsRNA binding domain (Figure 2, Supplementary Figure S6) that are both required for RNA coprocessing (Figure 2, Supplementary Figure S5). These data imply that the conservation of tracrRNA:pre-crRNA coprocessing by bacterial RNase III provides a degree of flexibility allowing the functionality of dual-RNA:Cas9 systems in multiple species upon horizontal transfer.

### Cas9 HNH and split RuvC domains are the catalytic moieties for DNA interference

**Figure 3.**
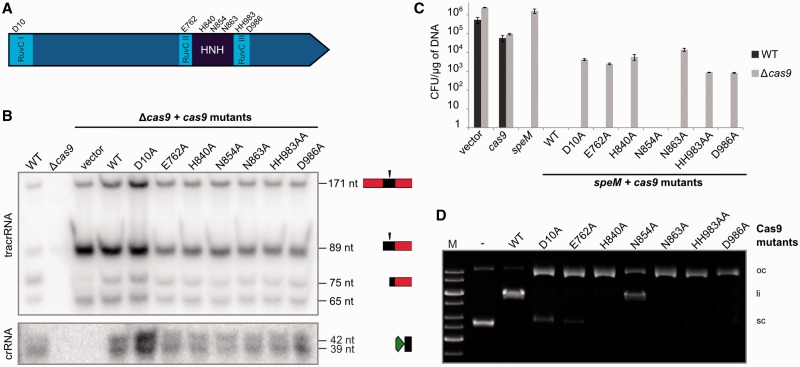
Conserved motifs of Cas9 are required for DNA interference but not for dual-RNA processing by RNase III. (**A**) Schematic representation of *S. pyogenes* Cas9. The conserved HNH and splitted RuvC motifs and analyzed amino acids are indicated. (**B**) Northern blot analysis of total RNA from *S. pyogenes* WT, Δ*cas9* and Δ*cas9* complemented with pEC342 or pEC342 containing *cas9* WT or mutant genes, probed for tracrRNA and crRNA repeat. Maturation of tracrRNA and pre-crRNA generating tracrRNA-75 nt and crRNA-39-42 nt forms is observed in all Δ*cas9* strains complemented with the *cas9* mutants. (**C**) *In vivo* protospacer targeting. Transformation assays of *S. pyogenes* WT and Δ*cas9* with pEC85 (vector), pEC85Ω*cas9* (*cas9*), pEC85Ω*speM* (*speM*), and pEC85ΩtracrRNA-171 nt plasmids containing *speM* and *cas9* mutants. The CFUs per µg of plasmid DNA were determined in at least three independent experiments. The results ±SD of technical triplicates of one representative experiment are shown. Cas9 N854A is the only mutant that did not tolerate the protospacer plasmid as observed for WT Cas9, indicating that this residue is not involved in DNA interference. (**D**) *In vitro* plasmid cleavage. Agarose gel electrophoresis of plasmid DNA (5 nM) containing *speM* protospacer (pEC287) incubated with 25 nM Cas9 WT or mutants in the presence of equimolar amounts of dual-RNA*-speM* (see ‘Materials and Methods’ section). Cas9 WT and N854A generated linear cleavage products while the other Cas9 mutants created only nicked products. M, 1 kb DNA ladder (Fermentas); oc: open circular, li: linear; sc: supercoiled.

Comparison of Cas9 sequences revealed high diversity in amino acid composition and length (984 amino acid for *C. jejuni* to 1648 amino acids for *F. novicida*), especially in the linker sequence between the highly conserved N-terminal RuvC and central RuvC-HNH-RuvC regions and in the C-terminal extension (Supplementary Figure S2). Several studies demonstrated the importance of the nuclease motifs for dsDNA cleavage activity by mutating one aspartate in the N-terminal motif of the RuvC domain and one or several residues in the predicted catalytic motif of the HNH domain of the Cas9 enzyme (14,22,23). To investigate the relevance of all catalytic motifs for tracrRNA:pre-crRNA processing and/or DNA interference, alanine substitutions of selected residues were created (Figure 3A). In addition to the already published catalytic amino acids, we created Cas9 point mutants of conserved amino acid residues in the central RuvC motifs (14) (Figure 3A, Supplementary Figure S2). Northern blot analysis of *S. pyogenes cas9* deletion mutant complemented with each of the *cas9* point mutants revealed the presence of mature tracrRNA and crRNA forms, demonstrating that none of the catalytic motifs is involved in dual-RNA maturation by RNase III. This is in agreement with previous data showing that RNase III is the enzyme that specifically cleaves tracrRNA:pre-crRNA duplex (16). Cas9 seems to have a stabilizing function on dual-RNA. We show that the catalytic motifs are not involved in RNA duplex stabilization (Figure 3B, Supplementary Figure S7).

To investigate the involvement of the conserved motifs of Cas9 in DNA interference *in vivo*, we used a previously described plasmid-based read-out system that mimics infection with invading protospacer-containing DNA elements (16). Transformation assays were done in *S. pyogenes* WT or a *cas9* deletion mutant using plasmids containing the *speM* protospacer gene (complementary to the second spacer of *S. pyogenes* SF370 type II CRISPR array (16)) and WT or mutant *cas9* (Figure 3C). In this assay, Cas9 expressed following plasmid delivery in bacterial cells catalyzes its own vector cleavage, when active. Control experiments showed that the *speM* protospacer-containing plasmid was not tolerated in WT *S. pyogenes*, demonstrating activity of WT CRISPR-Cas. Similarly, a plasmid containing the *speM* protospacer and encoding WT Cas9 could not be maintained in the *cas9* deletion mutant, demonstrating that Cas9 is able to cleave the plasmid from which it is expressed. Except for Cas9 N854A, all plasmids encoding Cas9 mutants were tolerated in the *cas9* deletion strain, indicating abrogation of Cas9 interference activity for these variants.

The *in vivo* DNA targeting data were confirmed with *in vitro* DNA cleavage assays. Purified WT and mutant Cas9 proteins were incubated with tracrRNA:crRNA targeting *speM* and subjected to cleavage of plasmid DNA containing the *speM* protospacer. WT and N854A Cas9 show dsDNA cleavage activity, whereas the other Cas9 mutants cleave only one strand of the dsDNA substrate, yielding nicked open circular plasmid DNA (Figure 3D). This corroborates the results obtained *in vivo* showing the importance of the conserved nuclease motifs for DNA interference by Cas9. In addition to the previously published data demonstrating the importance of the N-terminal RuvC motif and the catalytic motif of HNH, we thus defined new catalytic residues in the central RuvC motifs.

### Only Cas9 from closely related CRISPR-Cas systems can substitute for *S. pyogenes* Cas9 in tracrRNA-directed pre-crRNA maturation by RNase III

**Figure 4.**
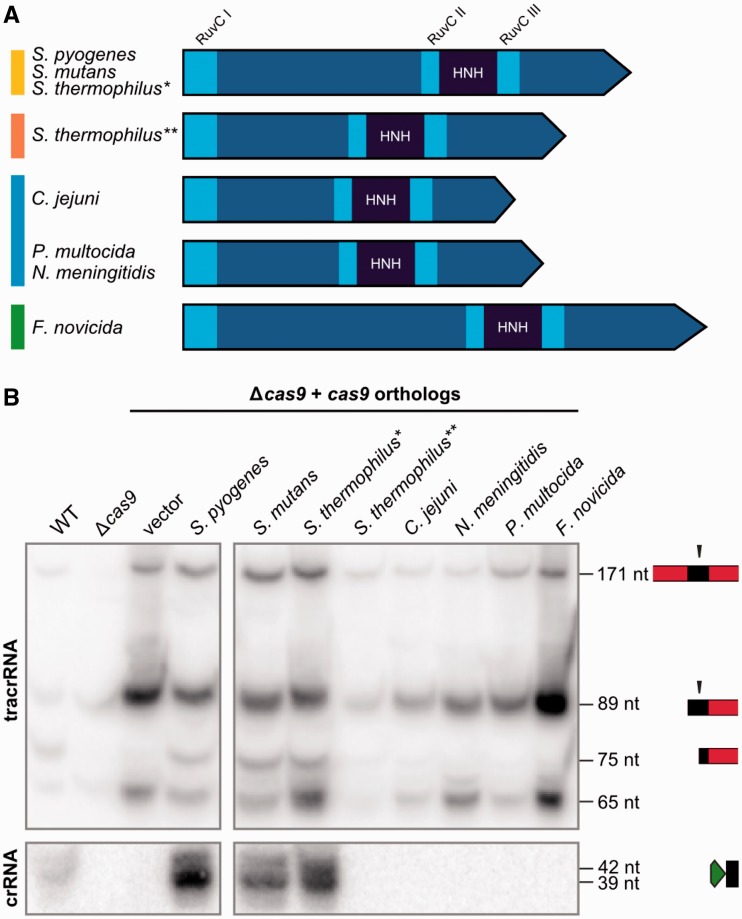
Cas9 from closely related CRISPR-Cas systems can substitute the role of *S. pyogenes* Cas9 in RNA processing by RNase III. (**A**) Schematic representation of Cas9 from selected bacterial species. The protein sizes and distances between conserved motifs (RuvC and HNH) are drawn in scale. See Supplementary Figure S2. (**B**) Northern blot analysis of total RNA extracted from *S. pyogenes* WT, Δ*cas9* and Δ*cas9* complemented with pEC342 (backbone vector containing tracrRNA-171 nt and the *cas* operon promoter from *S. pyogenes*) or pEC342-based plasmids containing *cas9* orthologous genes, probed for tracrRNA and crRNA repeat. Mature forms of *S. pyogenes* tracrRNA and pre-crRNA are observed only in the presence of *S. pyogenes* Cas9 WT or closely related Cas9 orthologs from *S. mutans* and S. thermophilus*.

Beside the conservation of the HNH and split RuvC domains involved in DNA cleavage (14,15), the length of Cas9 orthologs and the amino acid sequences of Cas9 are highly variable among the different groups of type II CRISPR-Cas systems (Figure 4A, Supplementary Figure S2). Hence, we investigated whether this variability plays a role in the specificity of Cas9 with regard to tracrRNA:pre-crRNA duplex and mature crRNA stabilization. We complemented *S. pyogenes cas9* deletion mutant with Cas9 from selected bacterial species representative of the various type II groups and analyzed tracrRNA:pre-crRNA processing by northern blot. Cas9 proteins from *S. mutans* and S. thermophilus* can substitute for the stabilizing role of *S. pyogenes* Cas9 in RNA processing by RNase III (Figure 4B, Supplementary Figure S8). In contrast, Cas9 from S. thermophilus**, *C. jejuni*, *N. meningitidis*, *P. multocida* and *F. novicida* could not complement the lack of RNA processing in the *cas9* mutant of *S. pyogenes*. In these strains, the 75-nt processed form of tracrRNA is observed as a very weak signal of background level of dual-RNA processed by RNase III in the absence of Cas9. Overall, only Cas9 from closely related systems of *S. pyogenes* in the type II-A cluster can substitute endogenous Cas9 role in dual-RNA stabilization and subsequent maturation by RNase III.

### Cas9 orthologs require their specific PAM sequence for DNA cleavage activity

**Figure 5.**
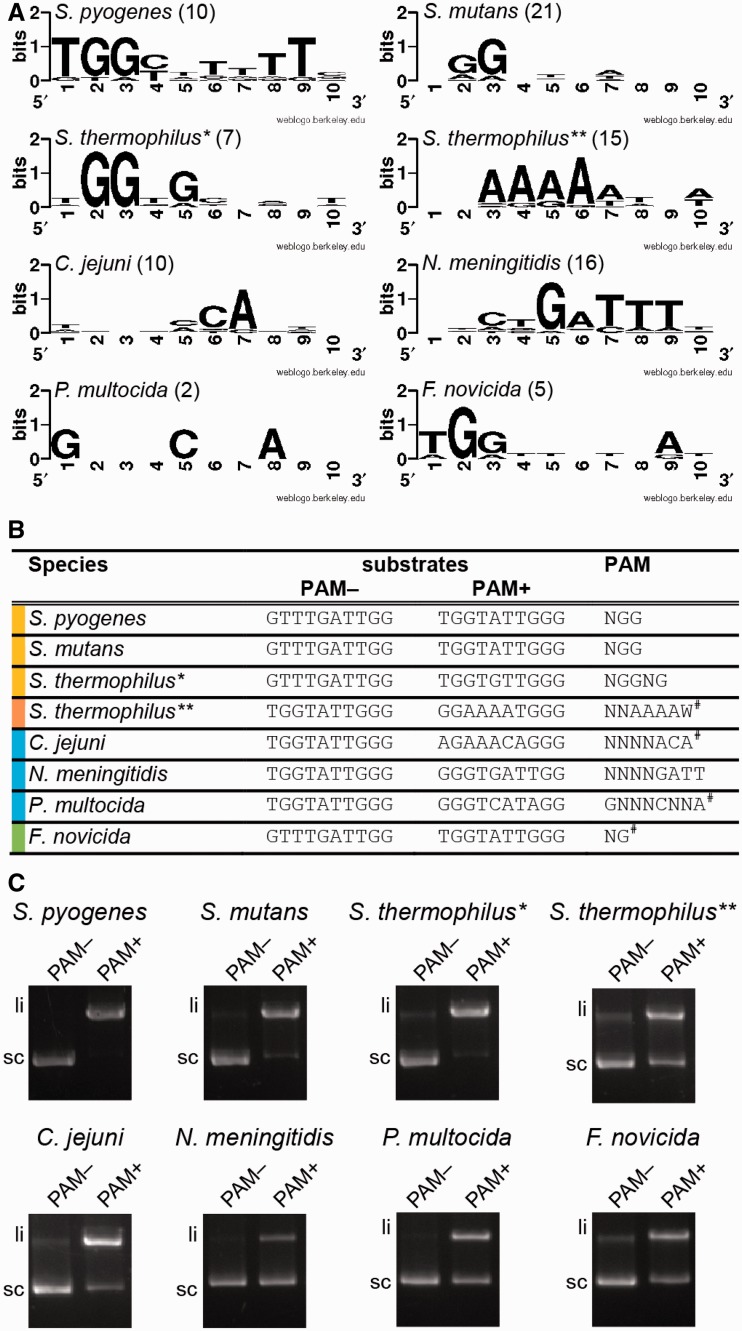
Cas9 orthologs cleave DNA in the presence of their cognate dual-RNA and specific PAM *in vitro.* (**A**) Logo plot of protospacer adjacent sequences derived from BLAST analysis of spacer sequences for selected bacterial species. The logo plot gives graphical representation of most abundant nucleotides downstream of the protospacer sequence. The numbers in brackets correspond to the number of analyzed protospacers. (**B**) DNA substrates designed for specific PAM verification. Based on the logo plot for each species, plasmid DNA substrates were designed to contain the *speM* protospacer and the indicated sequence downstream, either comprising (PAM+) or not (PAM−) the proposed PAM. The predicted PAMs were verified by cleavage assays narrowing down the necessary nucleotides for activity (data not shown); therefore the sequence used differs slightly from the logoplot shown in (**A**). The high abundance of other nucleotides not being part of the PAM can be explained by redundancy of the coding sequences containing the protospacers, and by the limited number of found protospacer targets. The last column shows the PAM sequence for each species, which was already published (no symbol) or derived from this work (^#^). (**C**) *In vitro* plasmid cleavage assays by dual-RNA:Cas9 orthologs on plasmid DNA with the 10-bp protospacer adjacent sequence (summarized in (B)). Each Cas9 ortholog in complex with its cognate dual-RNA cleaves plasmids containing the corresponding species-specific PAM (PAM+). No cleavage is observed with plasmids that did not contain the specific PAM (PAM−). li: linear cleavage product, sc: supercoiled plasmid DNA.

In *S. pyogenes* and S. thermophilus* types II-A, PAMs were identified as NGG and NGGNG, respectively. In these two species, mutating the PAM abrogates DNA interference by dual-RNA:Cas9 (14,22,23). To identify the functional PAMs for Cas9 from bacterial species other than *S. pyogenes* and *S. thermophilus*, we searched for potential protospacers matching spacer sequences in the selected CRISPR arrays using BLAST. For *S. mutans* UA159, *C. jejuni* NCTC 11168, *P. multocida* Pm70 and *F. novicida* U112, we were unable to identify potential protospacers. Therefore, we searched for strains that harbor a closely related variant of Cas9 (Supplementary Table S2) and analyzed their spacer sequences following the same approach (Supplementary Table S3). We aligned the identified 10 nt sequences located directly downstream of the protospacer sequence and delineated the most common nucleotides that could represent PAM sequences. Based on the data visualized as a logo plot (Figure 5A), we designed plasmid DNA substrates containing the *speM* protospacer followed by different adjacent sequences either comprising the predicted PAM or not (Figure 5B). The Cas9 orthologous proteins were purified (Supplementary Figure S1) and dual-RNA orthologs were designed based on deep RNA sequencing data (15), with the spacer sequence of crRNA targeting *speM*. To determine the protospacer-adjacent sequences critical for efficient DNA targeting, the purified Cas9 orthologs and their cognate dual-RNAs were used in DNA cleavage assays with different plasmid substrates (Figure 5C, Supplementary Figure S9). The previously published PAMs for Cas9 from *S. pyogenes* (NGG), *S. mutans* (NGG), S. thermophilus* (NGGNG) and *N. meningitidis* (NNNNGATT) (27,28,53,54) were confirmed by multiple sequence alignments and *in vitro* cleavage assay, validating our approach. However, dual-RNA guided Cas9 from S. thermophilus* could efficiently cleave target DNA in the presence of only NGG instead of NGGNG (Supplementary Figure S9). This is in contrast to data obtained *in vivo*, where mutation of the third G abrogates interference by Cas9 of S. thermophilus* (23). For S. thermophilus**, the PAM was published as NNAGAAW (27), which differs by one base from the sequence that we derived (NNAAAAW). *In vitro* cleavage assays with these two sequences demonstrate that the DNA substrate with the ‘NNAAAAW’ PAM is cleaved more efficiently by Cas9 of S. thermophilus** compared to the ‘NNAGAAW’ PAM (Supplementary Figure S9). Using the same approach, we also validated the PAM activity of the most common protospacer-downstream sequences for *C. jejuni*, *F. novicida* and *P. multocida* by *in vitro* cleavage assays, resulting in the most probable PAM sequences being NNNNACA (*C. jejuni*), GNNNCNNA (*P. multocida*) and NG (*F. novicida*) (Figure 5C, Supplementary Figure S9). Analysis of the protospacer-adjacent sequence from *C. jejuni* shows the same frequency of C and A (‘NNNNCCA’ or ‘NNNNACA’) at position 5 downstream of the protospacer (Supplementary Table S3). Hence, we tested both substrates for cleavage activity by *C. jejuni* dual-RNA:Cas9. Only the DNA target containing A at this position was cleaved efficiently (Supplementary Figure S9). This result could be explained by the origin of the protospacer, with the ‘NNNNCCA’ PAM being mostly found in genomic DNA or prophages of *Campylobacter* strains. In this case, the mutated PAM sequence on the chromosomally located protospacer prevents self-targeting. The *P. multocida* PAM requires further verification given that the multiple sequence alignment was derived from only two protospacer sequences. Thus, we identified a series of specific PAMs that enable dsDNA cleavage by dual-RNA:Cas9 complexes from different bacterial species *in vitro*. For gene editing purposes, it would be advisable to test a range of potential motifs to select those PAMs that would allow efficient targeting with limited off-site effect.

### Phylogenetic clustering of Cas9 defines dual-RNA:Cas9 exchangeability

**Figure 6.**
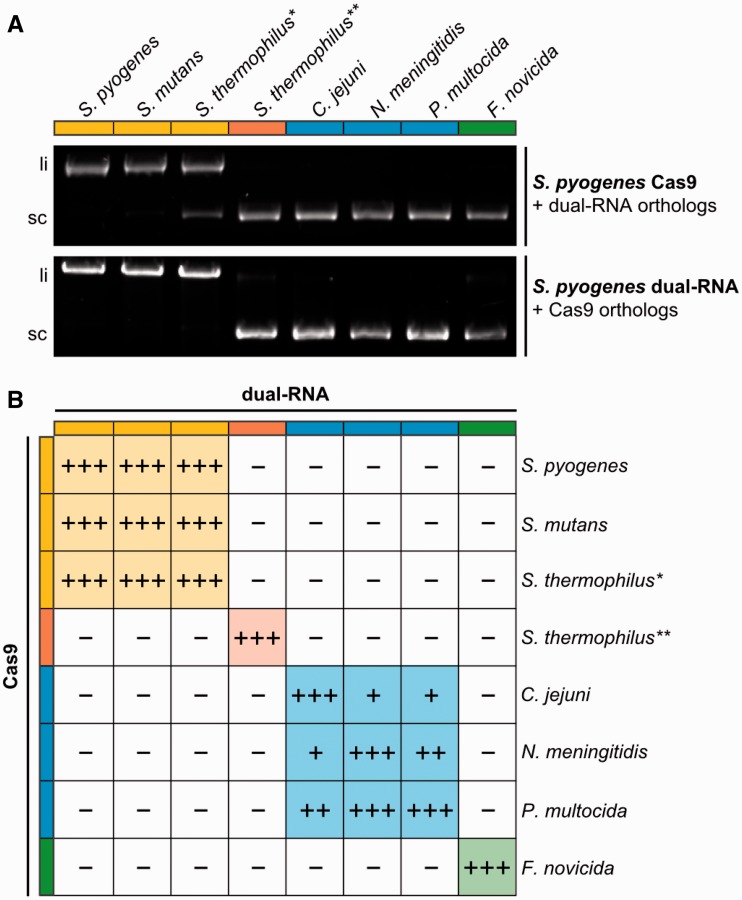
Cas9 and dual-RNA coevolved. (**A**) *In vitro* plasmid cleavage assays using *S. pyogenes* Cas9 in complex with orthologous dual-RNA (upper panel) and orthologous Cas9 enzymes in complex with *S. pyogenes* dual-RNA (lower panel). Plasmid DNA containing protospacer *speM* and *S. pyogenes* PAM (NGG) was incubated with different dual-RNAs in complex with *S. pyogenes* Cas9. tracrRNA and crRNA-repeat sequences of the dual-RNAs are from the indicated bacterial species, with crRNA spacer targeting *speM*. In the lower panel, plasmid DNA containing *speM* protospacer and the specific PAM was incubated with Cas9 orthologs in complex with *S. pyogenes* dual-RNA. *S. pyogenes* Cas9 can cleave plasmid DNA only in the presence of dual-RNA from *S. pyogenes*, *S. mutans* and S. thermophilus* (yellow). Dual-RNA from *S. pyogenes* can mediate DNA cleavage only with Cas9 from *S. pyogenes*, *S. mutans* and S. thermophilus* (yellow). li: linear cleavage product; sc: supercoiled plasmid DNA. (**B**) Summary of Cas9 and dual-RNA orthologs exchangeability. Specific PAM sequences were used according to Figure 5. The color code reflects the type II CRISPR-Cas subgroups (Figure 1). +++: 100–75% cleavage activity; ++: 75–50% cleavage activity; +: 50–25% cleavage activity; −: 25–0% cleavage activity observed under the conditions tested. Cas9 and dual-RNA duplexes from the same type II group can be interchanged and still mediate plasmid cleavage providing that the PAM sequence is specific for Cas9. See also Supplementary Figure S10.

As described above, clustering of Cas9 orthologs correlates with the ability to substitute for the RNA-stabilizing role of *S. pyogenes* Cas9 in tracrRNA:pre-crRNA processing by RNase III *in vivo* (Figure 4B). We investigated the exchangeability between Cas9 and dual-RNA in closely related CRISPR-Cas systems at the level of DNA interference. Plasmid cleavage assays were performed using *S. pyogenes* Cas9 complexed with dual-RNAs from selected CRISPR-Cas systems representative of the clustering of the type II CRISPR-Cas systems. As shown in Figure 6A (upper panel), *S. pyogenes* Cas9 can cleave target DNA in the presence of dual-RNAs from *S. mutans* and S. thermophilus* (type II-A, yellow subcluster), but not from any other tested species. The same result was observed when the dual-RNA from *S. pyogenes* was incubated with Cas9 orthologs from different bacteria (Figure 6A, lower panel). Cleavage assays were also performed with all Cas9 orthologs incubated with cognate and non-cognate dual-RNAs on their PAM-specific plasmid DNA. Only the combinations of Cas9 and dual-RNA within the same type II subcluster conferred dsDNA cleavage activity (Figure 6B, Supplementary Figure S10). More striking was the gradient of activity dependent on how closely related the species are in the corresponding type II group. This effect can be observed for *C. jejuni* Cas9 that is able to cleave DNA in the presence of dual-RNA from *P. multocida* and *N. meningitidis*, but not as efficient as with its own RNA (type II-C, blue subcluster). This finding is in good agreement with the phylogenetic tree of Cas9 (Figure 1A) showing that all three Cas9 orthologs belong to type II-C but *C. jejuni* Cas9 clusters more distantly from *P. multocida* and *N. meningitidis* Cas9. This effect was even greater for S. thermophilus** Cas9, which belongs to type II-A together with *S. pyogenes*, *S. mutans* and S. thermophilus*. However, none of the dual-RNAs from the three latter loci could direct DNA cleavage by S. thermophilus** Cas9. This result supports the recent findings demonstrating the lack of exchangeability between Cas9 from CRISPR1 and CRISPR3 of *S. thermophilus* DGCC7710 with regard to dual-RNA binding (17). We conclude that Cas9 and tracrRNA:crRNA interchangeabilty directly results from Cas9 coevolution with dual-RNA and follows the Cas9 phylogeny that may differ from the phylogeny of the respective bacterial species due to horizontal transfer.

## DISCUSSION

In this work, we identified and characterized dual-RNA and PAM requirements for eight Cas9 orthologous enzymes representative of the Cas9 phylogenetic grouping. To evaluate dual-RNA:Cas9 diversity, we performed bioinformatics analysis of type II CRISPR-Cas systems from available genomes and identified Cas9 orthologs in a plethora of bacterial species that belong to 12 phyla and were isolated from diverse environments (Supplementary Tables S2 and S4). Most of the strains that harbor type II CRISPR-Cas systems (and accordingly Cas9) are pathogens and commensals of vertebrates. A majority of these strains were isolated from gastrointestinal tracts and feces of mammals, fish and birds, but also from wounds, abscesses and spinocereberal fluid of septicaemia patients. We also identified strains isolated from invertebrates and environmental samples, including fresh and sea water, plant material, soil and food, the latter comprising species used in fermentation processes. Cas9 is also present in species from extreme environments such as deep sea sediments, hot springs and Antarctic ice, further demonstrating the wide spread of type II CRISPR-Cas systems in bacteria. A comparison of the taxonomy and habitats of representative strains with the phylogenetic clustering of Cas9 sequences shows little correlation (Supplementary Figure S11). In particular, we identified clusters of Cas9 genes from taxonomically distant bacteria that were isolated from similar habitats. Examples include diverse Firmicutes, Molicutes, Spirochaete and Fusobacteria, that were all isolated from gastrointestinal tracts of mammals, and members of different Proteobacteria, Firmicutes and Fusobacteria families mostly found in environmental samples (Supplementary Figure S11, clusters 1 and 3). A few exceptions involve grouping of Cas9 genes from closely related species isolated from diverse habitats such as Actinobacteria isolated from human and dog specimens but also from hot springs (Supplementary Figure S11, clusters 2, 4 and 5). This complex distribution of Cas9 across bacterial genomes indicates that evolution of dual-RNA:Cas9 systems in bacteria occurs both vertically and horizontally (55).

To investigate the basis for the horizontal dissemination of CRISPR-Cas modules among bacteria, we assessed the specificity of RNase III utilized by type II CRISPR-Cas for dual-RNA maturation. Complementation analysis shows that RNase III from a variety of species, including bacteria that lack type II CRISPR-Cas, can process *S. pyogenes* tracrRNA:pre-crRNA, suggesting that type II CRISPR-Cas systems can exploit any double-stranded RNA cleavage activity. This finding is consistent with the observation of *S. pyogenes* dual-RNA maturation in human cells which is apparently mediated by host RNases (2).

Dual-RNA and Cas9 sequences have widely evolved in bacteria (15). However, despite the high sequence variability among Cas9 sequences, certain motifs are conserved. In addition to the previously identified central HNH and N-terminal RuvC catalytic motifs (20,21,44,56), we show that the two middle RuvC motifs are required for interference activity *in vivo* and *in vitro*. In agreement with previous findings, deactivation of either one of the catalytic motifs (RuvC or HNH) results in nicking activity of Cas9 originating from the other motif (2,8,24,25). None of the mutations introduced in these conserved motifs affected the role of Cas9 in tracrRNA:pre-crRNA maturation by RNase III *in vivo*. The specificity of Cas9 towards different dual-RNAs might be explained by the high variability among Cas9 orthologs from different type II groups. To test this hypothesis, we selected representative bacterial species from the three major type II subtypes, namely *S. pyogenes, S. mutans* and *S. thermophilus* (II-A), *F. novicida* (II-B) and *C. jejuni*, *N. meningitidis* and *P. multocida* (II-C). Substitution of orthologs from the selected species for the endogenous *S. pyogenes* Cas9 shows that only Cas9 proteins from the *S. pyogenes* subcluster are capable of assisting tracrRNA:pre-crRNA processing by RNase III. This result indicates that the less-conserved inter-motif regions, which are the basis for the Cas9 subgrouping, could be responsible for Cas9 specificity for certain dual-RNAs.

To investigate the interchangeability between type II subgroups at the level of DNA interference, we first determined the PAMs specific for each of the eight selected Cas9 orthologs (28). By aligning potential crRNA-targeted sequences, we identified conserved motifs adjacent to the protospacers in all selected species. We then showed that these motifs are essential for DNA interference activity of the cognate dual-RNA:Cas9 complex *in vitro*. The interchangeability between dual-RNA and Cas9 from different subclusters was tested using plasmid cleavage assays. Only closely related Cas9 proteins can exchange their cognate dual-RNAs and still exert cleavage activity when using the Cas9 specific PAM. The specificity of Cas9 towards dual-RNAs is highly sensitive to the Cas9 sequence relatedness. This sensitivity is observed with Cas9 from *C. jejuni* that displays full cleavage activity with its cognate dual-RNA but reduced activity with dual-RNAs from *N. meningitidis* or *P. multocida* which belong to different subclusters of type II-C. We hypothesize that Cas9 possesses specificity for the secondary structure of dual-RNAs, given that bioinformatics predictions suggest similar structures of repeat:antirepeat duplexes in closely related CRISPR-Cas systems (Supplementary Figure S12).

This work provides the first experimental evidence in support of tracrRNA:crRNA duplex and Cas9 protein coevolution. Previously, we investigated the diversity of type II CRISPR-Cas system with respect to the loci architecture, tracrRNA sequence and position, and showed their correlation with the phylogenetic grouping of Cas9 (15). Here, the biological relevance of similarities within the Cas9 tree subclusters is demonstrated by limited exchangeability of the RNA and protein components. Thus, Cas9 and dual-RNA coevolved to maintain the functionality of the type II system. This finding also indicates that in bacteria harboring two distinct type II CRISPR-Cas systems (e.g *S. thermophilus* (18,57) or *Wolinella succinogenes* (15)), each system functions independently, with its own repeat-spacer array.

While this article was in preparation, a study by Hou *et al.* (34) showed the successful application of Cas9 from *N. menigitidis* for gene targeting in mammalian cells using the same PAM sequence as described here. This finding demonstrates that the various dual-RNA and Cas9 orthologs with their associated PAM sequences presented in this work have the potential to substantially enhance this novel genome editing tool by offering increased versatility and possibly specificity.

## SUPPLEMENTARY DATA

Supplementary Data are available at NAR Online, including [58–59].

## FUNDING

Swedish Research Council [K2010-57X-21436-01-3, K2013-57X-21436-04-3, 621-2011-5752-LiMS to E.C.]; the Kempe Foundation to E.C.; Umeå University [Dnr: 223- 2728-10, Dnr: 223-2836-10, Dnr: 223-2989-10 to E.C.]; the Laboratory for Molecular Infection Medicine Sweden to E.C. and the Helmholtz Association to E.C., K.S.M. and E.V.K. are supported by intramural funds of the US Department of Health and Human Services (to the National Library of Medicine). K.C. was a fellow of the Austrian Doctoral Program in RNA Biology. Funding for open access charge: Helmholtz Centre for Infection Research.

*Conflict of interest statement*. None declared.

## Supplementary Material

Supplementary Data
