# Supplementary material for: Phylogeny of Cas9 determines functional exchangeability of dual-RNA and Cas9 among orthologous type II CRISPR-Cas systems
Source: Nucleic Acids Res. 2013 Nov 21;42(4):2577–90. doi: 10.1093/nar/gkt1074 (PMC3936727; doi:10.1093/nar/gkt1074)
Supplement: Supplementary Data [file supp_42_4_2577__index.html]

Phylogeny of Cas9 determines functional exchangeability of dual-RNA and Cas9 among orthologous type II CRISPR-Cas systems — Phylogeny of Cas9 determines functional exchangeability of dual-RNA and Cas9 among orthologous type II CRISPR-Cas systems — Supplementary Data 

# Phylogeny of Cas9 determines functional exchangeability of dual-RNA and Cas9 among orthologous type II CRISPR-Cas systems

## Supplementary Data

files

**Files in this Data Supplement:**

- Supplementary Data - pdf file
