# Supplementary material for: Phylogeny of Cas9 determines functional exchangeability of dual-RNA and Cas9 among orthologous type II CRISPR-Cas systems
Source: Nucleic Acids Res. 2013 Nov 21;42(4):2577–90. doi: 10.1093/nar/gkt1074 (PMC3936727; doi:10.1093/nar/gkt1074)
Supplement: Supplementary Data [file supp_gkt1074_nar-02672-z-2013-File008.pdf]

Supplementary Table S1. Strains, plasmids and primers used in the study.

Strain	Relevant characteristics	Source
Streptococcus pyogenes		
**WT**		
EC904	SF370 (M1 serotype) (WT)	ATCC 700294
**Δ*cas9***		
EC1788	EC904Δ*cas9*	(16)
**Δ*rnc***		
EC1636	EC904Δ*rnc*	(16)
**Δ*cas9* in SF370 + *cas9* complementations *in trans***		
EC2121	EC1788 + pEC714 (P*cas9*(Spy)-*cas9*(Spy)-CtHis)	This study
EC2127	EC1788 + pEC710 (171 tracrRNA-P*cas9*(Spy)-CtHis)	This study
EC2150	EC1788 + pEC553 (P*cas9*(Spy)-*cas9*-HH983AA(Spy)-CtHis)	This study
EC2151	EC1788 + pEC554 (P*cas9*(Spy)-*cas9*-D10A(Spy)-CtHis)	This study
EC2152	EC1788 + pEC555 (P*cas9*(Spy)-*cas9*-H840A(Spy)-CtHis)	This study
EC2153	EC1788 + pEC556 (P*cas9*(Spy)-*cas9*-N854A(Spy)-CtHis)	This study
EC2154	EC1788 + pEC557 (P*cas9*(Spy)-*cas9*-N863A(Spy)-CtHis)	This study
EC2155	EC1788 + pEC558 (P*cas9*(Spy)-*cas9*-D986A(Spy)-CtHis)	This study
EC2156	EC1788 + pEC559 (P*cas9*(Spy)-*cas9*-E762A(Spy)-CtHis)	This study
EC2118	EC1788 + pEC518 (P*cas9*(Spy)-*cas9*(Cje)-CtHis)	This study
EC2128	EC1788 + pEC538 (P*cas9*(Spy)-*cas9*(Fno)-CtHis)	This study
EC2199	EC1788 + pEC544 (P*cas9*(Spy)-*cas9*(Nme)-CtHis)	This study
EC2119	EC1788 + pEC520 (P*cas9*(Spy)-*cas9*(Pmu)-CtHis)	This study
EC2111	EC1788 + pEC519 (P*cas9*(Spy)-*cas9*(Smu)-CtHis)	This study
EC2112	EC1788 + pEC521 (P*cas9*(Spy)-*cas9*(Sth*)-CtHis)	This study
EC2120	EC1788 + pEC522 (P*cas9*(Spy)-*cas9*(Sth**)-CtHis)	This study
**Δ*rnc* in SF370 + *rnc* complementations *in trans***		
EC2076	EC1636 + pEC484 (P*rnc*(Spy)-*rnc*(Spy))	This study
EC2084	EC1636 + pEC505 (P*rnc*(Spy)-*rnc*-catalytically inactive(Spy))	This study
EC2083	EC1636 + pEC504 (P*rnc*(Spy)-*rnc*-RNA binding inactive(Spy))	This study
EC2078	EC1636 + pEC486 (P*rnc*(Spy)-*rnc*(Cje))	This study
EC2080	EC1636 + pEC492 (P*rnc*(Spy)-*rnc*(Eco))	This study
EC2126	EC1636 + pEC537 (P*rnc*(Spy)-*rnc*(Fno))	This study
EC2085	EC1636 + pEC506 (P*rnc*(Spy)-*rnc*(Nme))	This study
EC2077	EC1636 + pEC485 (P*rnc*(Spy)-*rnc*(Pmu))	This study
EC2086	EC1636 + pEC507 (P*rnc*(Spy)-*rnc*(Sau))	This study
EC2082	EC1636 + pEC494 (P*rnc*(Spy)-*rnc*(Smu))	This study
EC2131	EC1636 + pEC534 (P*rnc*(Spy)-*rnc*(Sth))	This study
Campylobacter jejuni		
EC437	NCTC 11168; ATCC 700819 (WT), CIP 107370	Pasteur Institute
Francisella novicida		
EC1041	U112 (WT)	Anders Sjöstedt
Neisseria meningitidis		
EC438	CIP 107858	Pasteur Institute
Pasteurella multocida		
EC439	Pm70 (WT), ATCC BAA-1113	Pasteur Institute
Staphylococcus aureus		
EC36	COL (WT)	Lab strain collection
Streptococcus mutans		
EC1293	UA159 (WT)	(16)
Streptococcus thermophilus		
EC810	LMD-9 (WT)	(16)
E. coli		
RDN204	TOP10, host for cloning	Invitrogen
EC1265	Rosetta	Novagen

[a] Cje: *Campylobacter jejuni* NCTC 11168; Eco: *Escherichia coli* TOP10; Fno: *Francisella novicida* U112; Nme: *Neisseria meningitidis* A Z2491; Pmu: *Pasteurella multocida* Pm70; Sau: *Staphylococcus aureus* COL; Smu: *Streptococcus mutans* UA159; Spy: *Streptococcus pyogenes* SF370; Sth: *Streptococcus thermophilus* LMD-9.

Plasmid	Relevant characteristics	Source
**Vectors for *S. pyogenes***		
pEC85	*repDEG*-pAMβ1, pJH1-*aphIII*, ColE1	Bernhard Roppenser
**Plasmids for *cas9* domain functional and co-evolution analysis in *S. pyogenes* SF370**		
pEC268	pEC85Ω171 tracrRNA (171 nt form)	(16)
pEC309	pEC85Ω P*cas9*(Spy)-*cas9*(Spy)	(16)
pEC368	pEC85Ω171 tracrRNA-P*cas9*(Spy)-*cas9*(Spy)	(16)
pEC710	pEC85Ω171 tracrRNA-P*cas9*(Spy)-CtHis	This study
pEC714	pEC710Ω*cas9*(Spy)	This study
pEC553	pEC710Ω*cas9*-HH983AA(Spy)-CtHis	This study
pEC615	pEC553Ω*speM*	This study
pEC554	pEC710Ω*cas9*-D10A(Spy)-CtHis	This study
pEC659	pEC554ΩspeM	This study
pEC555	pEC710Ω*cas9*-H840A(Spy)-CtHis	This study
pEC660	pEC555ΩspeM	This study
pEC556	pEC710Ω*cas9*-N854A(Spy)-CtHis	This study
pEC661	pEC556ΩspeM	This study
pEC557	pEC710Ω*cas9*-N863A(Spy)-CtHis	This study
pEC618	pEC557Ω*speM*	This study
pEC558	pEC710Ω*cas9*-D986A(Spy)-CtHis	This study
pEC662	pEC558ΩspeM	This study
pEC559	pEC710Ω*cas9*-E762A(Spy)-CtHis	This study
pEC619	pEC559ΩspeM	This study
pEC518	pEC710Ω*cas9*(Cje)-CtHis	This study
pEC538	pEC710Ω*cas9*(Fno)-CtHis	This study
pEC544	pEC710Ω*cas9*(Nme)-CtHis	This study
pEC520	pEC710Ωcas9(Pmu)-CtHis	This study
pEC519	pEC710Ω*cas9*(Smu)-CtHis	This study
pEC521	pEC710Ωcas9(Sth*)-CtHis	This study
pEC522	pEC710Ωcas9(Sth**)-CtHis	This study
**Plasmids for *rnc* co-evolution analysis in *S. pyogenes* SF370**		
pEC483	pEC85ΩP*rnc*(Spy)	This study
pEC484	pEC85ΩP*rnc*(Spy)-*rnc*(Spy)	This study
pEC505	pEC85ΩP*rnc*(Spy)-*rnc*-catalytically inactive(Spy)	This study
pEC504	pEC85ΩP*rnc*(Spy)-*rnc*-RNA binding inactive(Spy)	This study
pEC486	pEC85ΩP*rnc*(Spy)-*rnc*(Cje)	This study
pEC492	pEC85ΩP*rnc*(Spy)-*rnc*(Eco)	This study
pEC537	pEC85ΩP*rnc*(Spy)-rnc(Fno)	This study
pEC506	pEC85ΩP*rnc*(Spy)-rnc(Nme)	This study
pEC485	pEC85ΩP*rnc*(Spy)-*rnc*(Pmu)	This study
pEC507	pEC85ΩP*rnc*(Spy)-*rnc*(Sau)	This study
pEC494	pEC85ΩP*rnc*(Spy)-*rnc*(Smu)	This study
pEC534	pEC85ΩP*rnc*(Spy)-*rnc*(Sth)	This study
**Plasmids for protospacer study *in vitro***		
pEC287	pEC85ΩP*speM*-*speM*<br>(10 bp downstream protospacer: GGGTATTGGG)	Lab plasmid collection
pEC691	pEC287 (10 bp downstream protospacer: TGGTATTGGG)	This study
pEC692	pEC287 (10 bp downstream protospacer: TGGTGTTGGG)	This study
pEC693	pEC287 (10 bp downstream protospacer: GGGTGATTGG)	This study
pEC694	pEC287 (10 bp downstream protospacer: GGAGAATGGG)	This study
pEC696	pEC287 (10 bp downstream protospacer: GGGTCATAGG)	This study
pEC697	pEC287 (10 bp downstream protospacer: AGAAACAGGG)	This study
pEC698	pEC287 (10 bp downstream protospacer: AGAACCAGGG)	This study
pEC701	pEC287 (10 bp downstream protospacer: GTTTGATTGG)	This study
pEC706	pEC287 (10 bp downstream protospacer: GGAAAATGGG)	This study
**Plasmids for Cas9 overexpression**		
pEC225	pET16b	Novagen
pEC621	pEC225 inserted with cassette harboring NotI, SacI, SalI site	This study
pEC626	pEC621Ω*cas9*(Spy)	This study
pEC627	pEC621Ω*cas9*-D10A(Spy)	This study
pEC628	pEC621Ω*cas9*-E762A(Spy)	This study
pEC629	pEC621Ωcas9-H840A(Spy)	This study
pEC630	pEC621Ωcas9-N854A(Spy)	This study
pEC631	pEC621Ωcas9-HH983AA(Spy)	This study
pEC632	pEC621Ω*cas9*(Cje)	This study
pEC633	pEC621Ω*cas9*(Pmu)	This study
pEC634	pEC621Ω*cas9*(Nme)	This study
pEC635	pEC621Ω*cas9*(Smu)	This study
pEC638	pEC621Ω*cas9*-N863A(Spy)	This study
pEC639	pEC621Ω*cas9*-D986A(Spy)	This study
pEC640	pEC621Ω*cas9*(Sth*)	This study
pEC641	pEC621Ω*cas9*(Sth**)	This study
pEC657	pEC621Ω*cas9*(Fno)	This study

Purpose	Primer	Sequence 5'-3'[a]	F/R[b]	Usage[c]
**tracrRNA expression in *S. pyogenes* SF370**				
tracrRNA	OLEC1014	GGACTAGCCTTATTTTAACTTG	R	NB (3' probe)
**crRNA (CRISPR01 (type II-A) expression in *S. pyogenes* SF370**				
crRNA	OLEC1049	GGACCATTCAAAACAGCATAGCTCTAAAAC	R	NB (repeat)
**Loading controls for Northern blots**				
5S rRNA	OLEC288	CTAAGCGACTACCTTATCTCA	R	NB
**His-tagged *cas9* constructs (pEC85-based)**				
pEC710	OLEC2151	GCAGGAATTCATCAGTGATGGTGATGGTGATGCCCGGGTTTGTCGACCT CCTAAAATAAAAAGTTTAAATTAAATC	F	Cloning
	OLEC2066	GGTGGTCTGCAGGTTTGCAGTCAGAGTAGAATAGAAG	R	
pEC714	OLEC2096	ATGCAGGTCGACATGGATAAGAAATACTCAATAGGC	F	Expression *cas9*(Spy)
	OLEC2097	ATGCAGCCCGGGGTCACCTCCTAGCTGACTCAAATC	R	
speM	OLEC2867	ATGCAGCCTGCAGGGTGACAGAGAGAAACTTGATTCAAC	F	Cloning of *speM* in other plasmids
	OLEC2868	ATGCAGCCTGCAGGCTTCGTTTAAGTAAACATCAAAGTG	R	
pEC518	OLEC2104	ATGCAGGTCGACGTGGCAAGAATTTTGGCATTTG	F	Cloning *cas9*(Cje)
	OLEC2105	ATGCAGCCCGGGTTTTTTAAAATCTTCTCTTTGTC	R	
pEC538	OLEC2840	ATTAGTCGACATGAATTTCAAAATATTGCCAATAG	F	Cloning *cas9*(Fno)
	OLEC2841	ATTACCCGGGATTATTAGATGTTTCATTATAAATAC	R	
pEC544	OLEC2092	ATGCAGGTCGACATGGCTGCCTTCAAACCTAATCC	F	Cloning *cas9*(Nme)
	OLEC2093	ATGCAGCCCGGGACGGACAGGCGGGCGTTTTTTCAG	R	
pEC520	OLEC2100	ATGCAGGTCGACATGCAAACAACAAATTTAAGTTA	F	Cloning *cas9*(Pmu)
	OLEC2101	ATGCAGCCCGGGACGCACAGGTTGTCTTTGCTGAG	R	
pEC519	OLEC2090	ATGCAGGTCGACATGAAAAAACCTTACTCTATTGGAC	F	Cloning *cas9*(Smu)
	OLEC2091	ATGCAGCCCGGGGTCTCCTCCTAACTTATTGAGATC	R	
pEC521	OLEC2098	ATGCAGGTCGACATGACTAAGCCATACTCAATTGG	F	Cloning *cas9*(Sth*)
	OLEC2099	ATGCAGCCCGGGACCCTCTCCTAGTTTGGCAAGGTC	R	
pEC522	OLEC2102	ATGCAGGTCGACATGAGTGACTTAGTTTTAGGACTTG	F	Cloning *cas9*(Sth**)
	OLEC2103	ATGCAGCCCGGGAAAATCTAGCTTAGGCTTATCACC	R	
pEC553	OLEC2229	GTACGTGAGATTAACAATTACGCTGCTGCCCATGATGCGTATCTA	F	Mutagenesis *cas9*-HH983AA(Spy)
	OLEC2230	TAGATACGCATCATGGGCAGCAGCGTAATTGTTAATCTCACGTAC	R	
pEC554	OLEC2128	GAAATACTCAATAGGCTTAGCTATCGGCACAAATAGCGTCG	F	Mutagenesis *cas9*-D10A(Spy)
	OLEC2129	CGACGCTATTTGTGCCGATAGCTAAGCCTATTGAGTATTTC	R	
pEC555	OLEC2223	TTTAAGTGATTATGATGTCGATGCCATTGTTCCACAAAGTTTCCT	F	Mutagenesis *cas9*-H840A(Spy)
	OLEC2224	AGGAAACTTTGTGGAACAATGGCATCGACATCATAATCACTTAAA	R	
pEC556	OLEC2225	CCTTAAAGACGATTCAATAGACGCTAAGGTCTTAACGCGTTCTGA	F	Mutagenesis *cas9*-N854A(Spy)
	OLEC2226	TCAGAACGCGTTAAGACCTTAGCGTCTATTGAATCGTCTTTAAGG	R	
pEC557	OLEC2227	GGTCTTAACGCGTTCTGATAAAGCTCGTGGTAAATCGGATAACGT	F	Mutagenesis *cas9*-N863A(Spy)
	OLEC2228	ACGTTATCCGATTTACCACGAGCTTTATCAGAACGCGTTAAGACC	R	
pEC558	OLEC2231	GTAACAATTACCATCATGCCCATGCTGCGTATCTAAATGCCGTCG	F	Mutagenesis *cas9*-D986A(Spy)
	OLEC2232	CGACGGCATTTAGATACGCAGCATGGGCATGATGGTAATTGTTAC	R	
pEC559	OLEC2221	CAGAAAATATCGTTATTGCAATGGCACGTGAAAATCAGACA	F	Mutagenesis *cas9*-E762A(Spy)
	OLEC2222	TGTCTGATTTTCACGTGCCATTGCAATAACGATATTTTCTG	R	
***rnc* constructs (pEC85-based)**				
pEC483	OLEC2149	ATGCAGGCATGCCCTGTAGTTTTGGCTTGTCTGATC	F	Cloning in pEC85
	OLEC3274	ATGCAGAGCTCCATGGAAAATCCCTTTCATATTTGTCAGTAGACC	R	
pEC484	OLEC2109	ATGCAGCCATGGAACAGCTTGAAGAGTTACTCTCAAC	F	Cloning *rnc*(Spy), SEQ
	OLEC1668	CTTTTAAAAACATCTAAACCTCAC	R	
pEC504	OLEC2109	ATGCAGCCATGGAACAGCTTGAAGAGTTACTCTCAAC	F	Cloning of *rnc* RNA binding inactive(Spy)
	OLEC2656	ATGCAGGAATTCCTACCCTTTTTCCACCTGAGGAATC	R	
pEC505	OLEC2142	GAACGCTTGGAATTTTTAGGAGCCGCTGTTCTACAATTGATTATT	F	Mutagenesis of *rnc* catalytically inactive(Spy)
	OLEC2143	AATAATCAATTGTAGAACAGCGGCTCCTAAAAATTCCAAGCGTTC	R	
pEC486	OLEC2116	ATGCAGCCATGGAAAACATTGAAAAGCTAGAGCAGAG	F	Cloning *rnc*(Cje), SEQ
	OLEC2117	ATGCAGGAATTCCTATAAAGCTCCTAATTTCTCAAG	R	
pEC492	OLEC2124	ATGCAGCCATGGACCCCATCGTAATTAATCGGCTTC	F	Cloning *rnc*(Eco), SEQ
	OLEC2125	ATGCAGGAATTCTCATTCCAGCTCCAGTTTTTTCAACG	R	
pEC537	OLEC2842	ATTACCATGGTTCCTGAATATTCACGATTTTATAAC	F	Cloning *rnc*(Fno), SEQ
	OLEC2843	ATTGAATTCCTATTTTTTTTCATGTAAGCCTTGTTGTG	R	
pEC506	OLEC2118	ATGCAGCCATGGAAGACGATGTTTTGAAACAGCAGG	F	Cloning *rnc*(Nme), SEQ
	OLEC2119	ATGCAGGAATTCTCATTTCTTTTTCTTCTTCAGCGGC	R	
pEC485	OLEC2114	ATGCAGCCATGGCTCAAAATTTAGAACGTTTACAACG	F	Cloning *rnc*(Pmu), SEQ
	OLEC2115	ATGCAGGAATTCTCATTTCATTTCCAATAATTGT	R	
pEC507	OLEC2126	ATGCAGCCATGGCTAAACAAAAGAAAAGTGAGATAG	F	Cloning *rnc*(Sau), SEQ
	OLEC2127	ATGCAGGAATTCCTATTTAATTTGTTTTAATTGCTTATAGG	R	
pEC494	OLEC2110	ATGCAGCCATGGAAACATTAGAAAAAAAACTGGCAG	F	Cloning *rnc*(Smu), SEQ
	OLEC2111	ATGCAGGAATTCTTAAGAACCTCGTTGAAGTTTTTC	R	
pEC534	OLEC2849	ATTACCATGGATCAACTTGAACAAAAACTTGAACAGGACTTTGG	F	Cloning *rnc*(Sth), SEQ
	OLEC2850	ATTAGAATTCTTAATTACCTAGTTGTTCAAGGGCAGACTTCGC	R	
**Cas9 overexpression (pEC621 based)**				
pEC621	OLEC2978	TAGCGGCCGCGAGCTCGTCGACGC	F	Cassette inserting NotI, SacI, SalI site in pEC225
	OLEC2979	TAGCGTCGACGAGCTCGCGGCCGC	R	

pEC626, 627, 628, 629, 630, 631, 638, 639	OLEC2097	ATGCAGGTCGACATGGATAAGAAATACTCAATAGGC	F	Cloning *cas9*(Spy and all mutants)
	OLEC2983	AGCTAGCGGCCGCTCAGTCACCTCCTAGCTGACTCAAATC	R	
pEC632	OLEC2104	ATGCAGGTCGACGTGGCAAGAATTTTGGCATTTG	F	Cloning *cas9*(Cje)
	OLEC2986	ATGCAGCGGCCGCTCATTTTTTAAAATCTTCTCTTTGTC	R	
pEC633	OLEC2100	ATGCAGGTCGACATGCAAACAACAAATTTAAGTTA	F	Cloning *cas9*(Pmu)
	OLEC2173	ATGACGCGGCCGCTTAACGCACAGGTTGTCTTTGCTG	R	
pEC634	OLEC2092	ATGCAGGTCGACATGGCTGCCTTCAAACCTAATCC	F	Cloning *cas9*(Nme)
	OLEC2982	ATGACGCGGCCGCTTAACGGACAGGCGGGCGTTTTTTCAG	R	
pEC635	OLEC2090	ATGCAGGTCGACATGAAAAAACCTTACTCTATTGGAC	F	Cloning *cas9*(Smu)
	OLEC2981	ATGACGCGGCCGCTTAGTCTCCTCCTAACTTATTGAG	R	
pEC640	OLEC2098	ATGCAGGTCGACATGACTAAGCCATACTCAATTGG	F	Cloning *cas9*(Sth*)
	OLEC2984	ATGACGCGGCCGCTTAACCCTCTCCTAGTTTGGCAAG	R	
pEC641	OLEC2102	ATGCAGGTCGACATGAGTGACTTAGTTTTAGGACTTG	F	Cloning *cas9*(Sth**)
	OLEC2985	ATGACGCGGCCGCTTAAAAATCTAGCTTAGGCTTATCAC	R	
pEC657	OLEC2840	ATTAGTCGACATGAATTTCAAAATATTGCCAATAG	F	Cloning *cas9*(Fno)
	OLEC2987	ATGCAGCGGCCGCCTAATTATTAGATGTTTCATTATAAATAC	R	
**Mutagenesis 10 bp downstream of *speM* protospacer**				
pEC691	OLEC3140	CAACCACTAATTTCTAGAAAAATCTTCG	R	Mutagenesis on pEC287
	OLEC3141	CAATTTGTAAAAAATGGTATTGGGGAATTC	F	
pEC692	OLEC3140	CAACCACTAATTTCTAGAAAAATCTTCG	R	Mutagenesis on pEC287
	OLEC3142	CAATTTGTAAAAAATGGTGTTGGGGAATTC	F	
pEC693	OLEC3140	CAACCACTAATTTCTAGAAAAATCTTCG	R	Mutagenesis on pEC287
	OLEC3144	CAATTTGTAAAAAAGGGTGATTGGGAATTC	F	
pEC694	OLEC3140	CAACCACTAATTTCTAGAAAAATCTTCG	R	Mutagenesis on pEC287
	OLEC3143	CAATTTGTAAAAAAGGAGAATGGGGAATTC	F	
pEC696	OLEC3194	CAACCACTAATTTTTAGAAAAATCTTCG	R	Mutagenesis on pEC693
	OLEC3197	CAATTTGTAAAAAAGGGTCATAGGGAATTC	F	
pEC697	OLEC3194	CAACCACTAATTTTTAGAAAAATCTTCG	R	Mutagenesis on pEC694
	OLEC3198	CAATTTGTAAAAAAAGAAACAGGGGAATTC	F	
pEC698	OLEC3194	CAACCACTAATTTTTAGAAAAATCTTCG	R	Mutagenesis on pEC694
	OLEC3199	CAATTTGTAAAAAAAGAACCAGGGGAATTC	F	
pEC701	OLEC3194	CAACCACTAATTTTTAGAAAAATCTTCG	R	Mutagenesis on pEC693
	OLEC3204	CAATTTGTAAAAAAGTTTGATTGGGAATTC	F	
pEC706	OLEC3194	CAACCACTAATTTTTAGAAAAATCTTCG	R	Mutagenesis on pEC694
	OLEC3208	CAATTTGTAAAAAAGGAAAATGGGGAATTC	F	
***In vitro* tracrRNA and crRNA of *Streptococcus pyogenes* SF370 (*speM* spacer underlined)**				
T7-tracrRNA	OLEC1521	GAAAT**TAATACGACTCACTATAG**AAAACAGCATAGCAAGTTAAAATAA	F	T7-tracrRNA 5'
	OLEC1522	AAAAAAAGCACCGACTCGGTGCCAC	R	T7-tracrRNA 3'
T7-crRNA (template)	OLEC2177	GAAAT**TAATACGACTCACTATAGG**ATAACTCAATTTGTAAAAAAGTTTTAGAGCTATGCTGTTTTG	F	crRNA speM 5’
	OLEC2179	CAAAACAGCATAGCTCTAAAACTTTTTTACAAATTGAGTTAT**CCTATAGTGAGTCGTATTA**ATTTC	R	crRNA speM 3’
***In vitro* tracrRNA and crRNA of *Neisseria meningitidis* A Z2491 (*speM* spacer underlined)**				
T7-tracrRNA (template)	OLEC3083	**GAAATTAATACGACTCACTATAG**GGAGAGCGAAATGAGAACCGTTGCTACAATAAGGCGTCTGAAAAGATGTGCCGCAACGCTCTGCCCCTTAAAGCTTCTGCTTTAAGGGGCATCGTTTATT	F	T7-tracrRNA 5'
	OLEC3084	AATAAACGATGCCCCTTAAAGCAGAAGCTTTAAGGGGCAGAGCGTTGCGGCACATCTTTTCAGACGCCTTATTGTAGCAACGGTTCTCATTTCGCTCTCC**CTATAGTGAGTCGTATTAATTTC**	R	T7-tracrRNA 3'
T7-crRNA (template)	OLEC2209	G**AAATTAATACGACTCACTATAG**ATGATAACTCAATTTGTAAAAAAGTTGTAGCTCCCTTTCTCATTT	F	crRNA speM 5'
	OLEC2214	AAATGAGAAAGGGAGCTACAACTTTTTTACAAATTGAGTTATCAT**CTATAGTGAGTCGTATTAATTTC**	R	crRNA speM 3'
***In vitro* tracrRNA and crRNA of *Streptococcus mutans* UA159 (*speM* spacer underlined)**				
T7-tracrRNA	OLEC3098	**GAAATTAATACGACTCACTATAG**GAAACAACACAGCAAGTTAAAATAAG	F	T7-tracrRNA 5'
	OLEC3099	AAATAAAAAAGCACCGAATCGG	R	T7-tracrRNA 3'
T7-crRNA (template)	OLEC3085	**GAAATTAATACGACTCACTATAG**GATAACTCAATTTGTAAAAAAGTTTTAGAGCTGTGTTGT	F	crRNA speM 5'
	OLEC3086	ACAACACAGCTCTAAAACTTTTTTACAAATTGAGTTATC**CTATAGTGAGTCGTATTAATTTC**	R	crRNA speM 3'
***In vitro* tracrRNA and crRNA of *Campylobacter jejuni* NCTC 11168 (*speM* spacer underlined)**				
T7-tracrRNA (template)	OLEC3128	**GAAATTAATACGACTCACTATAG**GAAGGGACTAAAATAAAGAGTTTGCGGGACTCTGCGGGGTTACAATCCCCTAAAACCGC	F	T7-tracrRNA 5'
	OLEC3129	GCGGTTTTAGGGGATTGTAACCCCGCAGAGTCCCGCAAACTCTTTATTTTAGTCCCTTCC**TATAGTGAGTCGTATTAATTTC**	R	T7-tracrRNA 3'
T7-crRNA (template)	OLEC3087	**GAAATTAATACGACTCACTATAG**GATAACTCAATTTGTAAAAAAGTTTTAGTCCCT	F	crRNA speM 5'
	OLEC3088	AGGGACTAAAACTTTTTTACAAATTGAGTTATC**CTATAGTGAGTCGTATTAATTTC**	R	crRNA speM 3'

***In vitro* tracrRNA and crRNAs of *Francisella novicida* U112 (*speM* spacer underlined)**				
T7-tracrRNA	OLEC3102	**GAAATTAATACGACTCACTATAG***GGTACCAAATAATTAATGCTCTG*	F	T7-tracrRNA 5'
	OLEC3103	*GTTATTCAGACGTGTCAAACAG*	R	T7-tracrRNA 3'
T7-crRNA (template)	OLEC3089	**GAAATTAATACGACTCACTATAG**GATAACTCAATTTGTAAAAAAGTTTCAGTTGCTGAATTATTTGGTAAAC	F	crRNA speM 5'
	OLEC3090	GTTTACCAAATAATTCAGCAACTGAAACTTTTTTACAAATTGAGTTATC**CTATAGTGAGTCGTATTAATTTC**	R	crRNA speM 3'
***In vitro* tracrRNA and crRNAs of *Streptococcus thermophilus** LMD-9 (*speM* spacer underlined)**				
T7-tracrRNA	OLEC3104	**GAAATTAATACGACTCACTATAG***GAACAACACAGCGAGTTAAAATAAGG*	F	T7-tracrRNA 5'
	OLEC3105	*AAAAAAAACACCGAATCGGTG*	R	T7-tracrRNA 3'
T7-crRNA (template)	OLEC3085	**GAAATTAATACGACTCACTATAG**GATAACTCAATTTGTAAAAAAGTTTTAGAGCTGTGTTGT	F	crRNA speM 5'
	OLEC3086	ACAACACAGCTCTAAAACTTTTTTACAAATTGAGTTATC**CTATAGTGAGTCGTATTAATTTC**	R	crRNA speM 3'
In vitro* tracrRNA and crRNAs of *Streptococcus thermophilus LMD-9 (*speM* spacer underlined)**				
T7-tracrRNA	OLEC3106	**GAAATTAATACGACTCACTATAG***GCTTAAATCTTGCAGAAGCTACAAAG*	F	T7-tracrRNA 5'
	OLEC3107	*AAATAACGAAAACACCCTGCC*	R	T7-tracrRNA 3'
T7-crRNA (template)	OLEC3091	**GAAATTAATACGACTCACTATAG**GATAACTCAATTTGTAAAAAAGTTTTTGTACTCTCAAGATTTA	F	crRNA speM 5'
	OLEC3092	TAAATCTTGAGAGTACAAAAACTTTTTTACAAATTGAGTTATC**CTATAGTGAGTCGTATTAATTTC**	R	crRNA speM 3'
***In vitro* tracrRNA and crRNAs of *Pasteurella multocida* Pm70 (*speM* spacer underlined)**				
T7-tracrRNA	OLEC3108	**GAAATTAATACGACTCACTATAG***GCTGCGAAATGAGAGACGTTGCTAC*	F	T7-tracrRNA 5'
	OLEC3109	*AAAAACGATGCCCCTTGCAATTAAG*	R	T7-tracrRNA 3'
T7-crRNA (template)	OLEC3093	**GAAATTAATACGACTCACTATAG**GATAACTCAATTTGTAAAAAAGTTGTAGTTCCCTCTCTCATTTCGC	F	crRNA speM 5'
	OLEC3094	GCGAAATGAGAGAGGGAACTACAACTTTTTTACAAATTGAGTTATC**CTATAGTGAGTCGTATTAATTTC**	R	crRNA speM 3'
**Primers for sequencing analysis**				
***cas9 Streptococcus mutans* UA159**				
cas9(Smu)	OLEC2792	*ATGAAAAAAACCTTACTCTATTGGA*	F	SEQ
	OLEC2793	*GATTTTAAAAAGCATTTTGAATTA*	F	SEQ
	OLEC2794	*TACTTGCCAAATCAAAAAGTTCTT*	F	SEQ
	OLEC2795	*ATTATGGGACATCAACCTGAAAAT*	F	SEQ
	OLEC2796	*TACCCACAATTGGAACCTGAATTT*	F	SEQ
***cas9 Neisseria meningitidis* A Z2491**				
cas9(Nme)	OLEC2797	*ATGGCTGCCTTCAAACCTAATCCA*	F	SEQ
	OLEC2798	*GTTCAAAAAAATGTTGGGGCATTGC*	F	SEQ
	OLEC2799	*ATCCATATTGAAACTGCAAGGGAA*	F	SEQ
	OLEC2800	*AACGCGTTTGACGGTAAAACCATA*	F	SEQ
***cas9 Streptococcus thermophilus** LMD-9**				
cas9(Sth*)	OLEC2807	*ATGACTAAGCCATACTCAATTGGA*	F	SEQ
	OLEC2808	*GATTTTAGGAAATGTTTTAATTTA*	F	SEQ
	OLEC2809	*TATTTGCCAGAAGAGAAGGTACTT*	F	SEQ
	OLEC2810	*GTAATGGGAGGAAGAAAACCCGAG*	F	SEQ
	OLEC2811	*GCAAGTGCTTTACTTAAGAAATAC*	F	SEQ
	OLEC2812	*TTACTTTATCATGCTAAGAGAATA*	F	SEQ
cas9 Streptococcus thermophilus LMD-9**				
cas9(Sth**)	OLEC2817	*ATGAGTGACTTAGTTTTAGGACTT*	F	SEQ
	OLEC2818	*ATTTTTGGAATTCTAATTGGGAAA*	F	SEQ
	OLEC2819	*GGAGACTTTGACAATATTGTCATC*	F	SEQ
	OLEC2820	*TTGAATTTGTGGAAAAAACAAAAG*	F	SEQ
	OLEC2821	*CAGGAAAAATACAATGACATTAAG*	F	SEQ
***cas9 Pasteurella multocida* Pm70**				
cas9(Pmu)	OLEC2813	*ATGCAAACAACAAATTTAAGTTAT*	F	SEQ
	OLEC2814	*ACGCATGAAAAAAATGAGTTTAAA*	F	SEQ
	OLEC2815	*CTTGGGAAATCTTTTAAAGAACGT*	F	SEQ
	OLEC2816	*TATGAAATGGTGGATCAAGAAAGC*	F	SEQ
***cas9 Campylobacter jejuni* NCTC 11168**				
cas9(Cje)	OLEC2822	*GTGGCAAGAATTTTGGCATTTGAT*	F	SEQ
	OLEC2823	*GATGAAAAAAGAGCGCCAAAAAAT*	F	SEQ
	OLEC2824	*AACTACAAGGCCAAAAAAGACGCC*	F	SEQ
	OLEC2825	*AACAAAAGGAAGTTTTTTGAGCCT*	F	SEQ
***cas9 Francisella novicida* U112**				
cas9(Fno)	OLEC2869	*ATGAATTTCAAAATATTGCCAATA*	F	SEQ
	OLEC2870	*TTAGATACTCTTTTAACTGATGAT*	F	SEQ
	OLEC2871	*TTAAAAGTCTTAAAGTCAAGTAAA*	F	SEQ
	OLEC2872	*GGTTCAGAAGATAAAAAAGGTAAT*	F	SEQ
	OLEC2873	*AGAATTTTCTGCCTACGTGATCTT*	F	SEQ
	OLEC2874	*CCAATACTAATCCATAAAGAACTA*	F	SEQ
	OLEC2875	*ACATCAAAAAATATTTTTTGGCTG*	F	SEQ

[a] *italic*, sequence annealing to the template; underlined, restriction site; **bold**, T7 promoter.
[b] F, forward primer; R, reverse primer.
[c] NB, probe for Northern blot; SEQ, sequencing

A

**Strain**[a]	**GI accession number**[b]	**Amino acids (including purification-tag)**	**Molecular weight [kDa] (including purification-tag)**	**ε [M^{-1} cm^{-1}]**[c]
S. pyogenes SF370	13622193	1368 (1392)	158.4 (161.2)	110470
S. mutans UA159	24379809	1345 (1369)	156.6 (159.4)	114190
*S. thermophilus** LMD-9	116628213	1388 (1412)	161.0 (163.8)	128390
*S. thermophilus*** LMD-9	116627542	1121 (1145)	129.4 (132.2)	103860
C. jejuni NCTC 11168	218563121	984 (1008)	114.9 (117.7)	78640
N. meningitidis A Z2491	218767588	1082 (1106)	124.3 (127.1)	101950
P. multocida Pm70	15602992	1056 (1080)	121.8 (124.6)	121460
F. novicida U112	118497352	1629 (1653)	190.4 (193.2)	189080

B

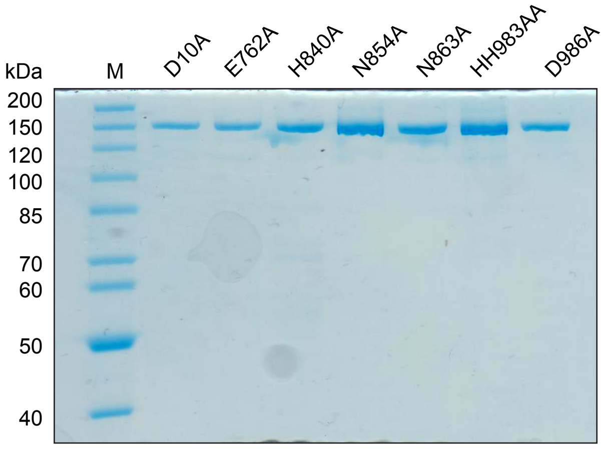

C

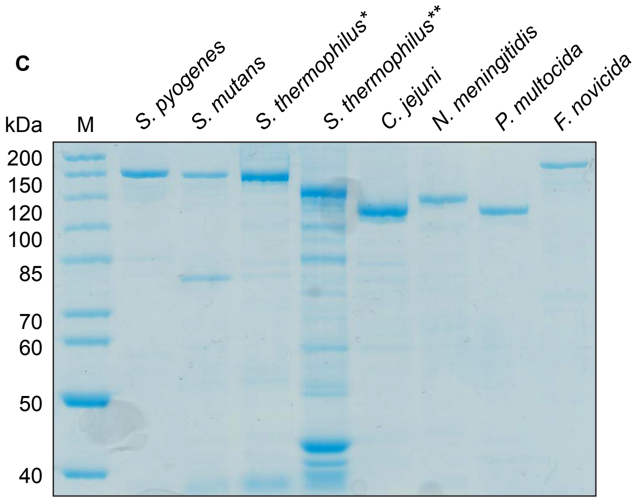

Supplementary Figure S1. Biochemical characteristics and SDS-PAGE analysis of Cas9 proteins purified in this study. (**A**) Overview of characteristics of Cas9 orthologous proteins [a]Note that the biochemical characteristics of *S. pyogenes* Cas9 WT and mutants are identical; [b]GenInfo (GI) Identifier; [c]ε, Extinction coefficient. (**B**) SDS PAGE analysis of purified mutants of Cas9 from *S. pyogenes*. (**C**) SDS PAGE analysis of purified Cas9 orthologs. M: PageRuler[TM] Unstained Protein Ladder (Thermo Scientific).

Supplementary Table S2. List of bacterial strains with identified Cas9 orthologs.

Cluster[a]	Strain[b]	Cas9 length (aa)	Cas9 GI	Cas1 GI[c]	Subtype[d]
	Dolosigranulum pigrum ATCC 51524	1332	375088882		
	Enterococcus faecalis ATCC 29200	1337	229548613		
	Enterococcus faecalis ATCC 4200	1337	256617555		
	Enterococcus faecalis D6	1337	257086028		
	Enterococcus faecalis E1Sol	1337	257080914		
	Enterococcus faecalis OG1RF	1337	384512368		
	Enterococcus faecalis TX0470	1337	312900261		
	Enterococcus faecalis TX4244	1337	422695652		
	Enterococcus faecium 1,141,733	1339	257888853		
	Enterococcus faecium 1,231,408	1340	257893735		
	Enterococcus faecium E1133	1339	430847551		
	Enterococcus faecium E3083	1340	431757680		
	Enterococcus faecium PC4.1	1340	293379700		
	Enterococcus faecium TX1330	1340	227550972		
	Enterococcus faecium TX1337RF	1340	424765774		
	Enterococcus hirae ATCC 9790	1336	392988474		
	Enterococcus italicus DSM 15952	1330	315641599		
	Lactobacillus animalis KCTC 3501	1314	335357451		
	Listeria innocua ATCC 33091	1337	423101383		
	Listeria innocua Clip11262	1334	16801805		
	Listeria innocua FSL S4-378	1103	422414122		
	Listeria ivanovii FSL F6-596	953	315305353		
	Listeria monocytogenes 10403S	1334	386044902		
	Listeria monocytogenes FSL J1-175	1099	255520581		
	Listeria monocytogenes FSL J1-194	1334	254825045		
	Listeria monocytogenes FSL J1-208	1334	422810631		
	Listeria monocytogenes FSL N3-165	1334	254829042		
	Listeria monocytogenes FSL R2-503	1334	254854201		
	Listeria monocytogenes str. 1/2a F6854	1334	47097148		
**1**	*Streptococcus agalactiae* 2603V/R	1370	22537057		**Type II-A**
	Streptococcus agalactiae 515	1377	77413160		
	Streptococcus agalactiae A909	1370	76788458		
	Streptococcus agalactiae ATCC 13813	1378	339301617		
	Streptococcus agalactiae CJB111	1370	77411010		
	Streptococcus agalactiae COH1	1370	77407964		
	Streptococcus agalactiae FSL S3-026	1370	417005168		
	Streptococcus agalactiae GB00112	1370	421147428		
	Streptococcus agalactiae H36B	1370	77405721		
	Streptococcus agalactiae NEM316	1377	25010965		
	Streptococcus agalactiae SA20-06	1370	410594450		
	Streptococcus agalactiae STIR-CD-17	1370	421532069		
	Streptococcus anginosus F0211	1345	315223162		
	Streptococcus anginosus SK1138	1386	421490579		
	Streptococcus anginosus SK52 = DSM 20563	1396	335031483		
	Streptococcus bovis ATCC 700338	1373	306833855		
	Streptococcus canis FSL Z3-227	1375	392329410		
	Streptococcus constellatus subsp. *constellatus* SK53	1345	418965022		
	Streptococcus dysgalactiae subsp. *equisimilis* AC-2713	1371	410494913		
	Streptococcus dysgalactiae subsp. *equisimilis* ATCC 12394	1371	386317166		
	Streptococcus dysgalactiae subsp. *equisimilis* GGS_124	1371	251782637		
	Streptococcus dysgalactiae subsp. *equisimilis* RE378	1371	408401787		
	Streptococcus equi subsp. *zooepidemicus* MGCS10565	1348	195978435		
	Streptococcus equinus ATCC 9812	1377	320547102		
	Streptococcus gallolyticus subsp. *gallolyticus* ATCC BAA-2069	1370	325978669		
	Streptococcus gallolyticus subsp. *gallolyticus* TX20005	1370	306831733		
	Streptococcus gallolyticus UCN34	1371	288905639		
	Streptococcus infantarius subsp. *infantarius* CJ18	1375	379705580		
	Streptococcus iniae 9117	1368	406658208		
	Streptococcus macacae NCTC 11558	1338	357636406		

Cluster[a]	Strain[b]	Cas9 length (aa)	Cas9 GI	Cas1 GI[c]	Subtype[d]
1 (continued)	*Streptococcus mitis* SK321	1392	307710946		Type II-A
	Streptococcus mutans 11SSST2	1345	449165720		
	Streptococcus mutans 11SSST2	1345	449951835		
	Streptococcus mutans 11VS1	1345	449976542		
	Streptococcus mutans 14D	1345	450149988		
	Streptococcus mutans 15VF2	1355	449170557		
	Streptococcus mutans 15VF2	1355	449965974		
	Streptococcus mutans 1SM1	1345	449158457		
	Streptococcus mutans 1SM1	1345	449920643		
	Streptococcus mutans 24	1350	449247589		
	Streptococcus mutans 24	1350	450180942		
	Streptococcus mutans 2VS1	1345	449174812		
	Streptococcus mutans 2VS1	1345	449968746		
	Streptococcus mutans 3SN1	1345	449162653		
	Streptococcus mutans 3SN1	1345	449931425		
	Streptococcus mutans 4SM1	1345	449159838		
	Streptococcus mutans 4SM1	1345	449927152		
	Streptococcus mutans 4VF1	1345	449167132		
	Streptococcus mutans 4VF1	1345	449961027		
	Streptococcus mutans 5SM3	1345	449176693		
	Streptococcus mutans 5SM3	1345	449980571		
	Streptococcus mutans 66-2A	1359	449240165		
	Streptococcus mutans 66-2A	1359	450160342		
	Streptococcus mutans 8ID3	1345	449154769		
	Streptococcus mutans 8ID3	1345	449872064		
	Streptococcus mutans A19	1345	449187668		
	Streptococcus mutans A19	1345	450013175		
	Streptococcus mutans B	1345	450166294		
	Streptococcus mutans G123	1345	450029806		
	Streptococcus mutans GS-5	1345	397650022		
	Streptococcus mutans LJ23	1345	387785882		
	Streptococcus mutans M21	1345	449194333		
	Streptococcus mutans M21	1345	450036249		
	Streptococcus mutans M230	1345	449260994		
	Streptococcus mutans M230	1345	449903532		
	Streptococcus mutans M2A	1345	449209586		
	Streptococcus mutans M2A	1345	450074072		
	Streptococcus mutans N29	1345	449182997		
	Streptococcus mutans N29	1345	450003067		
	Streptococcus mutans N3209	1345	449210660		
	Streptococcus mutans N3209	1345	450077860		
	Streptococcus mutans N66	1345	449212466		
	Streptococcus mutans N66	1345	450083993		
	Streptococcus mutans NFSM1	1350	449202104		
	Streptococcus mutans NFSM1	1350	450051112		
	Streptococcus mutans NLML1	1345	450140393		
	Streptococcus mutans NLML4	1338	449202681		
	Streptococcus mutans NLML4	1338	450059882		
	Streptococcus mutans NLML9	1345	449209148		
	Streptococcus mutans NLML9	1345	450066176		
	Streptococcus mutans NMT4863	1355	449186850		
	Streptococcus mutans NMT4863	1355	450007078		
	Streptococcus mutans NN2025	1345	290580220		
	Streptococcus mutans NV1996	1345	450086338		
	Streptococcus mutans NVAB	1345	449181424		
	Streptococcus mutans NVAB	1345	449990810		
	Streptococcus mutans R221	1345	449258042		
	Streptococcus mutans R221	1345	449899675		
	Streptococcus mutans S1B	1345	449251227		
	Streptococcus mutans S1B	1345	449877120		
	Streptococcus mutans SF1	1345	450098705		
	Streptococcus mutans SF14	1345	449221374		

Cluster[a]	Strain[b]	Cas9 length (aa)	Cas9 GI	Cas1 GI[c]	Subtype[d]
	Streptococcus mutans SF14	1345	450107816		
	Streptococcus mutans SM1	1345	449245264		
	Streptococcus mutans SM1	1345	450176410		
	Streptococcus mutans SM4	1345	449246010		
	Streptococcus mutans SM4	1345	450170248		
	Streptococcus mutans SM6	1345	449223000		
	Streptococcus mutans SM6	1345	450112022		
	Streptococcus mutans ST6	1350	449227252		
	Streptococcus mutans ST6	1350	450123011		
	***Streptococcus mutans* UA159**	**1345**	**24379809**	**24379808**	
	Streptococcus mutans W6	1345	450094364		
	Streptococcus oralis SK304	1373	421488030		
	Streptococcus oralis SK610	1371	419782534		
	Streptococcus pseudoporcinus LQ 940-04	1374	416852857		
	***Streptococcus pyogenes* SF370 (M1 GAS)**	**1368**	**13622193**	**13622194**	
	Streptococcus pyogenes MGAS10270	1368	94543903		
	Streptococcus pyogenes MGAS10750	1371	94994317		
	Streptococcus pyogenes MGAS15252	1367	383479946		
	Streptococcus pyogenes MGAS2096	1368	94992340		
**1 (continued)**	*Streptococcus pyogenes* MGAS315	1368	21910213		**Type II-A**
	Streptococcus pyogenes MGAS5005	1368	71910582		
	Streptococcus pyogenes MGAS6180	1368	71903413		
	Streptococcus pyogenes MGAS9429	1368	94988516		
	Streptococcus pyogenes NZ131	1368	209559356		
	Streptococcus pyogenes SSI-1	1368	28896088		
	Streptococcus ratti FA-1 = DSM 20564	1370	400290495		
	Streptococcus salivarius K12	1385	421452908		
	Streptococcus sanguinis SK115	1377	422848603		
	Streptococcus sanguinis SK330	1392	422860049		
	Streptococcus sanguinis SK353	1370	422821159		
	Streptococcus sp. C300	1377	322375978		
	Streptococcus sp. F0441	1371	414157437		
	Streptococcus sp. M334	1375	322378004		
	Streptococcus sp. oral taxon 56 str. F0418	1371	339640839		
	Streptococcus suis ST1	1381	389856936		
	Streptococcus thermophilus	1388	343794781		
	***Streptococcus thermophilus* LMD-9**	**1388**	**116628213**	**116628212**	
	Streptococcus thermophilus MN-ZLW-002	1388	387910220		
	Streptococcus thermophilus ND03	1388	386087120		
	Campylobacter coli 1098	984	419564797		
	Campylobacter coli 111-3	984	419536531		
	Campylobacter coli 132-6	987	419572019		
	Campylobacter coli 151-9	984	419603415		
	Campylobacter coli 1909	984	419576091		
	Campylobacter coli 1957	965	419581876		
	Campylobacter coli 2692	984	419553162		
	Campylobacter coli 59-2	984	419578074		
	Campylobacter coli 67-8	965	419587721		
	Campylobacter coli 80352	965	419558307		
	Campylobacter coli 80352	987	419559505		
**2**	*Campylobacter jejuni* subsp. *doylei* 269.97	984	153952471		**Type II-C**
	Campylobacter jejuni subsp. *jejuni* 110-21	987	419676124		
	Campylobacter jejuni subsp. *jejuni* 129-258	987	419619138		
	Campylobacter jejuni subsp. *jejuni* 1336	987	283956897		
	Campylobacter jejuni subsp. *jejuni* 140-16	984	419681578		
	Campylobacter jejuni subsp. *jejuni* 1577	984	419685099		
	Campylobacter jejuni subsp. *jejuni* 1854	987	419689467		
	Campylobacter jejuni subsp. *jejuni* 1997-10	984	419666522		
	Campylobacter jejuni subsp. *jejuni* 2008-1025	987	419650041		
	Campylobacter jejuni subsp. *jejuni* 2008-872	984	419654778		
	Campylobacter jejuni subsp. *jejuni* 2008-979	987	419660762		
	Campylobacter jejuni subsp. *jejuni* 2008-988	965	419656328		

Cluster[a]	Strain[b]	Cas9 length (aa)	Cas9 GI	Cas1 GI[c]	Subtype[d]
**2 (continued)**	*Campylobacter jejuni* subsp. *jejuni* 2008-988	984	419655317		**Type II-C**
	Campylobacter jejuni subsp. *jejuni* 260.94	961	86152042		
	Campylobacter jejuni subsp. *jejuni* 414	985	283953849		
	Campylobacter jejuni subsp. *jejuni* 51037	984	419674189		
	Campylobacter jejuni subsp. *jejuni* 51494	984	419619463		
	Campylobacter jejuni subsp. *jejuni* 53161	987	419647275		
	Campylobacter jejuni subsp. *jejuni* 60004	984	419629136		
	Campylobacter jejuni subsp. *jejuni* 81116	984	157415744		
	Campylobacter jejuni subsp. *jejuni* 84-25	984	88596565		
	Campylobacter jejuni subsp. *jejuni* 87459	984	419680124		
	Campylobacter jejuni subsp. *jejuni* ATCC 33560	984	419643715		
	Campylobacter jejuni subsp. *jejuni* CF93-6	987	86149266		
	Campylobacter jejuni subsp. *jejuni* CG8486	984	148925683		
	Campylobacter jejuni subsp. *jejuni* HB93-13	984	86152450		
	Campylobacter jejuni subsp. *jejuni* LMG 23210	987	419696801		
	Campylobacter jejuni subsp. *jejuni* LMG 23211	984	419697443		
	Campylobacter jejuni subsp. *jejuni* LMG 23263	984	419628620		
	Campylobacter jejuni subsp. *jejuni* LMG 23264	984	419632476		
	Campylobacter jejuni subsp. *jejuni* LMG 23269	987	419634246		
	Campylobacter jejuni subsp. *jejuni* LMG 23357	987	419641132		
	***Campylobacter jejuni* subsp. *jejuni* NCTC 11168**	**984**	**218563121**	**218563120**	
	Campylobacter jejuni subsp. *jejuni* NW	983	424845990		
	Campylobacter jejuni subsp. *jejuni* PT14	987	407942868		
	Campylobacter lari	1003	345468028		
	Helicobacter canadensis MIT 98-5491	1007	253828136		
	Helicobacter cinaedi ATCC BAA-847	1023	396079277		
	Helicobacter cinaedi CCUG 18818	1023	313144862		
	Helicobacter cinaedi PAGU611	1023	386762035		
**3**	*Catellicoccus marimammalium* M35/04/3	1140	424780480		**Type II-A**
	Lactobacillus farciminis KCTC 3681	1126	336394701		
	Listeriaceae bacterium TTU M1-001	1087	381184145		
	Streptococcus anginosus 1_2_62CV	1125	319939170		
	Streptococcus gallolyticus UCN34	1130	288905632		
	Streptococcus gordonii str. Challis substr. CH1	1136	157150687		
	Streptococcus infantarius ATCC BAA-102	1129	171779984		
	Streptococcus macedonicus ACA-DC 198	1130	374338350		
	Streptococcus mitis ATCC 6249	1134	306829274		
	Streptococcus mutans NLML5	1128	449203378		
	Streptococcus mutans NLML5	1128	450064617		
	Streptococcus mutans NLML8	1125	449151037		
	Streptococcus mutans NLML8	1125	450133520		
	Streptococcus mutans ST1	1134	449228751		
	Streptococcus mutans ST1	1134	450114718		
	Streptococcus mutans U2A	1125	449232458		
	Streptococcus mutans U2A	1125	450125471		
	Streptococcus oralis SK1074	1121	418974877		
	Streptococcus oralis SK313	1134	417940002		
	Streptococcus parasanguinis F0449	1140	419799964		
	Streptococcus pasteurianus ATCC 43144	1130	336064611		
	Streptococcus salivarius JIM8777	1127	387783792		
	Streptococcus salivarius PS4	1135	419707401		
	Streptococcus sp. BS35b	1026	401684660		
	Streptococcus sp. C150	1139	322372617		
	Streptococcus sp. GMD6S	1121	406576934		
	Streptococcus suis 89/1591	1122	223932525		
	Streptococcus suis D9	1122	386584496		
	Streptococcus suis ST3	1122	330833104		
	Streptococcus thermophilus CNRZ1066	1128	55822627		
	Streptococcus thermophilus JIM 8232	1121	386344353		
	***Streptococcus thermophilus* LMD-9**	**1121**	**116627542**	**116627543**	
	Streptococcus thermophilus LMG 18311	1122	55820735		
	Streptococcus thermophilus MN-ZLW-002	1121	387909441		

Cluster[a]	Strain[b]	Cas9 length (aa)	Cas9 GI	Cas1 GI[c]	Subtype[d]
**3 (continued)**	*Streptococcus thermophilus* MTCC 5460	1122	445374534		**Type II-A**
	Streptococcus thermophilus ND03	1121	386086348		
	Streptococcus vestibularis ATCC 49124	1128	322517104		
**4**	*Actinobacillus minor* NM305	1056	240949037		**Type II-C**
	Actinobacillus pleuropneumoniae serovar 10 str. D13039	1054	307256472		
	Actinobacillus succinogenes 130Z	1062	152978060		
	Actinobacillus suis H91-0380	1054	407692091		
	Haemophilus parainfluenzae ATCC 33392	1054	325578067		
	Haemophilus parainfluenzae CCUG 13788	1052	359298684		
	Haemophilus parainfluenzae T3T1	1052	345430422		
	Haemophilus sputorum HK 2154	1052	402304649		
	Kingella kingae PYKK081	1060	381401699		
	Neisseria bacilliformis ATCC BAA-1200	1077	329117879		
	Neisseria cinerea ATCC 14685	1082	261378287		
	Neisseria flavescens SK114	1081	241759613		
	Neisseria lactamica 020-06	1082	313669044		
	Neisseria meningitidis 053442	1082	161869390		
	Neisseria meningitidis 2007056	1082	433531983		
	Neisseria meningitidis 63049	1082	433514137		
	Neisseria meningitidis 8013	1082	385324780		
	Neisseria meningitidis 92045	1082	421559784		
	Neisseria meningitidis 93003	1081	421538794		
	Neisseria meningitidis 93004	1081	421541126		
	Neisseria meningitidis 96023	1082	433518260		
	Neisseria meningitidis 98008	1081	421555531		
	Neisseria meningitidis alpha14	1082	254804356		
	Neisseria meningitidis alpha275	1082	254672046		
	Neisseria meningitidis ATCC 13091	1082	304388355		
	Neisseria meningitidis N1568	1081	416164244		
	Neisseria meningitidis NM140	1081	421545139		
	Neisseria meningitidis NM220	1082	418291220		
	Neisseria meningitidis NM233	1082	418288950		
	Neisseria meningitidis WUE 2594	1082	385337435		
	***Neisseria meningitidis* Z2491**	**1082**	**218767588**	**218767587**	
	Neisseria sp. oral taxon 14 str. F0314	1089	298369677		
	Neisseria wadsworthii 9715	1097	350570326		
	Pasteurella multocida subsp. *gallicida* X73	1058	425063822		
	Pasteurella multocida subsp. *multocida* str. P52VAC	1056	421263876		
	***Pasteurella multocida* subsp. *multocida* str. Pm70**	**1056**	**15602992**	**15602991**	
	Simonsiella muelleri ATCC 29453	1063	404379108		
**5**	*Lactobacillus brevis* subsp. *gravesensis* ATCC 27305	1377	227509761		**Type II-A**
	Lactobacillus buchneri CD034	1371	406027703		
	Lactobacillus buchneri NRRL B-30929	1371	331702228		
	Lactobacillus casei BL23	1361	191639137		
	Lactobacillus casei Lc-10	1361	418010298		
	Lactobacillus casei M36	1363	417996992		
	Lactobacillus casei str. Zhang	1361	301067199		
	Lactobacillus casei T71499	1360	417999832		
	Lactobacillus casei UCD174	1366	418002962		
	Lactobacillus casei W56	1389	409997999		
	Lactobacillus coryniformis subsp. *coryniformis* KCTC 3167	1354	333394446		
	Lactobacillus curvatus CRL 705	1368	354808135		
	Lactobacillus fermentum 28-3-CHN	1313	260662220		
	Lactobacillus fermentum ATCC 14931	1381	227514633		
	Lactobacillus florum 2F	1327	408790128		
	Lactobacillus gasseri JV-V03	1391	300361537		
	Lactobacillus hominis CRBIP 24.179	1386	395244248		
	Lactobacillus jensenii 269-3	1391	238854567		
	Lactobacillus jensenii 27-2-CHN	1395	256852176		
	Lactobacillus johnsonii DPC 6026	1375	385826041		
	Lactobacillus mucosae LM1	1382	377831443		
	Lactobacillus paracasei subsp. *paracasei* 8700:2	1362	239630053		

Cluster[a]	Strain[b]	Cas9 length (aa)	Cas9 GI	Cas1 GI[c]	Subtype[d]
	Lactobacillus pentosus IG1	1382	339637353		
	Lactobacillus pentosus KCA1	1361	392947436		
	Lactobacillus pentosus MP-10	1358	334881121		
	Lactobacillus plantarum ZJ316	1358	448819853		
	***Lactobacillus rhamnosus* GG**	**1363**	**258509199**	**258509198**	
	Lactobacillus rhamnosus HN001	1361	199597394		
**5**	*Lactobacillus rhamnosus* R0011	1361	418072660		
**(continued)**	*Lactobacillus ruminis* ATCC 25644	1375	323340068		**Type II-A**
	Lactobacillus salivarius SMXD51	1339	418960525		
	Lactobacillus sanfranciscensis TMW 1.1304	1331	347534532		
	Lactobacillus sp. 66c	1419	408410332		
	Pediococcus acidilactici DSM 20284	1364	304386254		
	Pediococcus acidilactici MA18/5M	1366	418068659		
	Psychroflexus torquis ATCC 700755	1509	408489713		
	Anaerophaga sp. HS1	1552	371776944		
	Anaerophaga thermohalophila DSM 12881	1515	346224232		
	Bacteroides coprophilus DSM 18228	1509	224026357		
	Bacteroides coprosuis DSM 18011	1504	333031006		
	Bacteroides dorei DSM 17855	1504	212694363		
	Bacteroides eggerthii 1_2_48FAA	1509	317474201		
	Bacteroides faecis 27-5	1526	380696107		
	Bacteroides fluxus YIT 12057	1509	329965125		
	Bacteroides nordii CL02T12C05	1512	393788929		
	***Bacteroides* sp. 20_3**	**1517**	**301311869**	**301311870**	**Type II-C**
	Bacteroides sp. D2	1510	383115507		
	Bacteroides uniformis CL03T00C23	1508	423303159		
	Bacteroides vulgatus CL09T03C04	1504	423312075		
	Capnocytophaga gingivalis ATCC 33624	1436	228473057		
	Capnocytophaga sp. CM59	1437	402830627		
	Capnocytophaga sp. oral taxon 324 str. F0483	1471	429756885		
	Capnocytophaga sp. oral taxon 326 str. F0382	1450	429752492		
**6**	*Capnocytophaga* sp. oral taxon 412 str. F0487	1450	393778597		
	Chryseobacterium sp. CF314	1419	399023756		
	Fibrobacter succinogenes subsp. *succinogenes* S85	1512	261414553		
	Flavobacteriaceae bacterium S85	1516	372210605		
	Flavobacterium columnare ATCC 49512	1459	365960762		
	Fluviicola taffensis DSM 16823	1458	327405121		
	Mucilaginibacter paludis DSM 18603	1473	373954054		
	Myroides odoratus DSM 2801	1466	374597806		
	Ornithobacterium rhinotracheale DSM 15997	1535	392391493		
	Prevotella bivia JCVIHMP010	1485	282858617		**Type II-C**
	Prevotella buccae ATCC 33574	1457	315607525		
	Prevotella nigrescens ATCC 33563	1506	340351024		
	Prevotella sp. MSX73	1483	402307189		
	Prevotella timonensis CRIS 5C-B1	1487	282881485		
	Prevotella veroralis F0319	1496	260592128		
	Sphingobacterium spiritivorum ATCC 33861	1426	300771242		
	Weeksella virosa DSM 16922	1440	325955459		
	Bacteroides fragilis 638R	1436	375360193		
	***Bacteroides fragilis* NCTC 9343**	**1436**	**60683389**	**60683388**	
	Bacteroides sp. 2_1_16	1436	265767599		
	Bacteroides sp. 3_1_19	1424	298377533		
	Bacteroides sp. D2	1436	383110723		
	Bacteroidetes oral taxon 274 str. F0058	1434	298373376		
	Belliella baltica DSM 15883	1352	390944707		
**7**	*Bergeyella zoohelcum* CCUG 30536	1430	406673990		**Type II-C**
	Capnocytophaga canimorsus Cc5	1430	340622236		
	Capnocytophaga ochracea DSM 7271	1426	256819408		
	Capnocytophaga sp. oral taxon 329 str. F0087	1435	332882466		
	Capnocytophaga sp. oral taxon 335 str. F0486	1426	420149252		
	Capnocytophaga sp. oral taxon 380 str. F0488	1432	429748017		
	Capnocytophaga sputigena Capno	1426	213962376		

Cluster[a]	Strain[b]	Cas9 length (aa)	Cas9 GI	Cas1 GI[c]	Subtype[d]
**7 (continued)**	*Flavobacterium psychrophilum* JIP02/86	1354	150025575		**Type II-C**
	Galbibacter sp. ck-I2-15	1391	408370397		
	Indibacter alkaliphilus LW1	1354	404451234		
	Joostella marina DSM 19592	1397	386818981		
	Kordia algicida OT-1	1391	163754820		
	Marinilabilia sp. AK2	1345	410030899		
	Myroides injenensis M09-0166	1401	399927444		
	Niabella soli DSM 19437	1426	374372722		
	Parabacteroides johnsonii DSM 18315	1443	218258638		
	Parabacteroides sp. D13	1424	256840409		
	Prevotella histicola F0411	1375	357042839		
	Prevotella intermedia 17	1380	387132277		
	Prevotella nigrescens F0103	1380	445119230		
	Prevotella oralis ATCC 33269	1391	323344874		
	Prevotella sp. oral taxon 306 str. F0472	1375	383811446		
	Riemerella anatipestifer RA-CH-1	1405	407451859		
	Riemerella anatipestifer RA-GD	1400	386321727		
	Zunongwangia profunda SM-A87	1388	295136244		
**8**	*Actinomyces coleocanis* DSM 15436	1105	227494853		**Type II-C**
	Actinomyces georgiae F0490	1113	420151340		
	Actinomyces naeslundii str. Howell 279	1101	400293272		
	Actinomyces sp. ICM47	1144	396585058		
	Actinomyces sp. oral taxon 175 str. F0384	1095	343523232		
	Actinomyces sp. oral taxon 181 str. F0379	1103	429758968		
	Actinomyces sp. oral taxon 848 str. F0332	1120	269219760		
	Actinomyces turicensis ACS-279-V-Col4	1114	405979650		
	Bifidobacterium dentium Bd1	1138	283456135		
	***Bifidobacterium longum* DJO10A**	**1187**	**189440764**	189440765	
	Bifidobacterium longum subsp. *longum* 2-2B	1124	419852381		
	Bifidobacterium longum subsp. *longum* KACC 91563	1138	384200944		
	Bifidobacterium sp. 12_1_47BFAA	1151	317482066	**317482065**	
	Corynebacterium accolens ATCC 49725	1099	227502575		
	Corynebacterium accolens ATCC 49726	1099	306835141		
	Corynebacterium diphtheriae 241	1084	375289763		
	Corynebacterium diphtheriae 31A	1084	376283539		
	Corynebacterium diphtheriae BH8	1084	376286566		
	Corynebacterium diphtheriae bv. *intermedius* str. NCTC 5011	1084	419861895		
	Corynebacterium diphtheriae C7 (beta)	1084	376289243		
	Corynebacterium diphtheriae HC02	1084	376292154		
	Corynebacterium diphtheriae NCTC 13129	1084	38232678		
	Corynebacterium diphtheriae VA01	1084	376256051		
	Corynebacterium matruchotii ATCC 14266	1089	305681510		
	Corynebacterium matruchotii ATCC 33806	1069	225021644		
	Gardnerella vaginalis 1500E	1186	415717744		
	Gardnerella vaginalis 284V	1186	415703177		
	Gardnerella vaginalis 5-1	1186	298252606		
	Mobiluncus curtisii subsp. *holmesii* ATCC 35242	1123	315656340		
	Mobiluncus mulieris 28-1	1091	269977848		
	Mobiluncus mulieris FB024-16	1091	307700167		
	Scardovia inopinata F0304	1178	294790575		
**9**	*Bacillus cereus* BAG4X12-1	1068	423439645		**Type II-C**
	Bacillus cereus BAG4X2-1	1078	423445130		
	Bacillus cereus Rock1-15	1069	229113166		
	***Bacillus smithii* 7_3_47FAA**	**1088**	**365156657**	365156658	
	Bacillus thuringiensis serovar *finitimus* YBT-020	1069	384183447		
	Brevibacillus laterosporus GI-9	1092	421874297	**421874296**	
	Clostridium perfringens C str. JGS1495	1065	169343975		
	Clostridium perfringens D str. JGS1721	1065	182624245		
	Sporolactobacillus vineae DSM 21990 = SL153	1084	404330915		
**10**	*Gemella haemolysans* ATCC 10379	1392	241889924		**Type II-A**
	Gemella morbillorum M424	1385	317495358		
	Megasphaera sp. UPII 135-E	1352	342218215		

Cluster[a]	Strain[b]	Cas9 length (aa)	Cas9 GI	Cas1 GI[c]	Subtype[d]
**10 (continued)**	***Veillonella atypica* ACS-134-V-Col7a**	**1398**	**303229466**	**303229394**	**Type II-A**
	Veillonella parvula ATCC 17745	1398	282849530		
	Veillonella sp. 6_1_27	1395	294792465		
	Veillonella sp. oral taxon 780 str. F0422	1120	342213964		
**11**	*Treponema denticola* AL-2	1395	449103686		**Type II-A**
	Treponema denticola ASLM	1395	449106292		
	***Treponema denticola* ATCC 35405**	**1395**	**42525843**	**42525844**	
	Treponema denticola H1-T	1395	449118593		
	Treponema denticola H-22	1395	449117322		
	Treponema denticola OTK	1395	449125136		
	Treponema denticola SP37	1395	449130155		
**12**	***Mycoplasma canis* PG 14**	**1233**	**384393286**	**384393287**	**Type II-A**
	Mycoplasma canis PG 14	1233	419703974		
	Mycoplasma canis UF31	1233	384937953		
	Mycoplasma canis UF33	1233	419704625		
	Mycoplasma canis UFG1	1233	419705269		
	Mycoplasma canis UFG4	1233	419705920		
	Mycoplasma cynos C142	1239	433625054		
**13**	*Enterococcus faecalis* Fly1	1150	257084992		**Type II-A**
	Enterococcus faecalis R508	1150	424761124		
	Enterococcus faecalis T11	1150	257419486		
	***Enterococcus faecalis* TX0012**	**1150**	**315149830**	**315149831**	
	Enterococcus faecalis TX0012	1150	422729710		
	Enterococcus faecalis TX1342	1150	422701955		
	Facklamia hominis CCUG 36813	1142	406671118		
**14**	*Gluconacetobacter diazotrophicus* PAl 5	1003	209542524		**Type II-C**
	Gluconacetobacter diazotrophicus PAl 5	1050	162147907		
	Methylocystis sp. ATCC 49242	1080	323139312		
	***Methylosinus trichosporium* OB3b**	**1082**	**296446027**	**296446028**	
	Rhodopseudomonas palustris BisB18	1066	90425961		
	Rhodopseudomonas palustris BisB5	1064	91975509		
	Tistrella mobilis KA081020-065	1049	389874754		
**15**	*Francisella* cf. *novicida* 3523	1646	387824704		**Type II-B**
	Francisella cf. *novicida* Fx1	1629	385792694		
	Francisella novicida FTG	1629	208779141		
	Francisella novicida GA99-3548	1629	254374175		
	***Francisella novicida* U112**	**1629**	**118497352**	**118497353**	
	Francisella tularensis subsp. *novicida* GA99-3549	1629	254372717		
**16**	*Acidovorax avenae* subsp. *avenae* ATCC 19860	1045	326315085		**Type II-C**
	Alicycliphilus denitrificans BC	1029	319760940		
	***Alicycliphilus denitrificans* K601**	**1029**	**330822845**	**330822846**	
	gamma proteobacterium HdN1	1025	304313029		
	Nitrosomonas sp. AL212	1044	325983496		
	Verminephrobacter eiseniae EF01-2	1068	121608211		
**17**	*Mycoplasma gallisepticum* NC95_13295-2-2P	1269	401767318		**Type II-A**
	Mycoplasma gallisepticum NY01_2001.047-5-1P	1224	401768851		
	***Mycoplasma gallisepticum* str. F**	**1269**	**284931710**	**284931711**	
	Mycoplasma gallisepticum str. F	1269	385326554		
	Mycoplasma gallisepticum str. R(low)	1270	294660600		
**18**	*Prevotella buccalis* ATCC 35310	1218	282878504		**Type II-C**
	Prevotella ruminicola 23	1204	294674019		
	Prevotella stercorea DSM 18206	1216	359406728		
	Prevotella tannerae ATCC 51259	1234	258648111		
	***Prevotella timonensis* CRIS 5C-B1**	**1218**	**282880052**	**282880053**	
**19**	*Phascolarctobacterium succinatutens* YIT 12067	1087	323142435		**Type II-C**
	Roseburia intestinalis L1-82	1140	257413184		
	Roseburia intestinalis M50/1	1128	291537230		
	***Roseburia inulinivorans* DSM 16841**	**1152**	**225377804**	**225377803**	
	Subdoligranulum sp. 4_3_54A2FAA	1084	365132400		
**20**	***Coriobacterium glomerans* PW2**	**1384**	**328956315**	**328956316**	**Type II-A**
	Eggerthella sp. YY7918	1380	339445983		

Cluster[a]	Strain[b]	Cas9 length (aa)	Cas9 GI	Cas1 GI[c]	Subtype[d]
**20 (continued)**	*Gordonibacter pamelaeae* 7-10-1-b	1371	295106015		**Type II-A**
	Olsenella uli DSM 7084	1399	302336020		
**21**	***Fusobacterium nucleatum* subsp. *vincentii* ATCC 49256**	**1374**	**34762592**	34762593	**Type II-A**
	Fusobacterium sp. 1_1_41FAA	1367	294782278		
	Fusobacterium sp. 3_1_27	1367	294785695		
	Fusobacterium sp. 3_1_36A2	1367	256845019	**256845020**	
**22**	*Finegoldia magna* ACS-171-V-Col3	1347	302380288		**Type II-A**
	***Finegoldia magna* ATCC 29328**	**1348**	**169823755**	**169823756**	
	Finegoldia magna SY403409CC001050417	1348	417926052		
	Helcococcus kunzii ATCC 51366	1338	375092427		
**23**	*Prevotella denticola* CRIS 18C-A	1422	325859619		**Type II-C**
	Prevotella micans F0438	1425	373501184		
	***Prevotella* sp. C561**	**1424**	**345885718**	**345885719**	
**24**	*Leuconostoc gelidum* KCTC 3527	1355	333398273		**Type II-A**
	***Oenococcus kitaharae* DSM 17330**	**1389**	**366983953**	**366983954**	
	Oenococcus kitaharae DSM 17330	1389	372325145		
**25**	*Anaerococcus tetradius* ATCC 35098	1361	227501312		**Type II-A**
	Lactobacillus iners LactinV 11V1-d	1369	309803917		
	***Peptoniphilus duerdenii* ATCC BAA-1640**	**1364**	**304438954**	**304438953**	
**26**	***Coprococcus catus* GD/7**	**1338**	**291520705**	**291520706**	**Type II-A**
	Dorea longicatena DSM 13814	1340	153855454		
	Ruminococcus lactaris ATCC 29176	1341	197301447		
**27**	***Staphylococcus pseudintermedius* ED99**	**1334**	**323463801**	**323463802**	**Type II-A**
	Staphylococcus pseudintermedius ED99	1334	386318630		
	Staphylococcus simulans ACS-120-V-Sch1	1112	414160476		
**28**	***Dinoroseobacter shibae* DFL 12**	**1079**	**159042956**	**159042957**	**Type II-C**
	Sphingobium sp. AP49	1110	398385143		
	Sphingomonas sp. S17	1090	332188827		
**29**	***Flavobacterium branchiophilum* FL-15**	**1473**	**347536497**	no *cas1*	**Type II-C**
	Flavobacterium columnare ATCC 49512	1535	365959402		
**30**	***Bifidobacterium bifidum* S17**	**1420**	**310286728**	**310286727**	**Type II-A**
	Scardovia wiggsiae F0424	1471	423349694		
**31**	*Burkholderiales* bacterium 1_1_47	1428	303257695		**Type II-B**
	***Parasutterella excrementihominis* YIT 11859**	**1428**	**331001027**	**331001028**	
**32**	***Streptococcus sanguinis* SK49**	**1421**	**422884106**	**422884107**	**Type II-A**
	Streptococcus sp. oral taxon 71 str. 73H25AP	1420	306826314		
**33**	*Eubacterium* sp. AS15	1391	402309258		**Type II-A**
	***Eubacterium yurii* subsp. *margaretiae* ATCC 43715**	**1391**	**306821691**	**306821690**	
**34**	*Legionella pneumophila* 130b	1372	307608922		**Type II-B**
	***Legionella pneumophila* str. Paris**	**1372**	**54296138**	**54296139**	
**35**	*Acidaminococcus intestini* RyC-MR95	1358	352684361		**Type II-A**
	***Acidaminococcus* sp. D21**	**1358**	**227824983**	**227824982**	
**36**	***Lactobacillus farciminis* KCTC 3681**	**1356**	**336394882**	**336394883**	**Type II-A**
	Lactobacillus versmoldensis KCTC 3814	1289	365906066		
**37**	*Mycoplasma synoviae* 53	1304	144575181		**Type II-A**
	***Mycoplasma synoviae* 53**	**1314**	**71894592**	**71894593**	
**38**	***Elusimicrobium minutum* Pei191**	**1195**	**187250660**	**187250661**	**Type II-C**
	uncultured Termite group 1 bacterium phylotype Rs-D17	1032	189485059		
**39**	*Clostridium spiroforme* DSM 1552	1116	169349750		**Type II-A**
	***Eubacterium dolichum* DSM 3991**	**1096**	**160915782**	**160915783**	
**40**	***Eubacterium rectale* ATCC 33656**	**1114**	**238924075**	**238924076**	**Type II-A**
	Eubacterium ventriosum ATCC 27560	1107	154482474		
**41**	*Staphylococcus aureus* subsp. aureus	1053	403411236		**Type II-A**
	***Staphylococcus lugdunensis* M23590**	**1054**	**315659848**	**315659847**	
**42**	***Ignavibacterium album* JCM 16511**	**1688**	**385811609**	**385811610**	**Type II-C**
**43**	***Odoribacter laneus* YIT 12061**	**1498**	**374384763**	**374384762**	**Type II-C**
**44**	***Caenispirillum salinarum* AK4**	**1442**	**427429481**	**427429479**	**Type II-C**
**45**	***Sutterella wadsworthensis* 3_1_45B**	**1422**	**319941583**	**319941582**	**Type II-B**
**46**	***Bergeyella zoohelcum* ATCC 43767**	**1415**	**423317190**	**423317188**	**Type II-C**
**47**	***Wolinella succinogenes* DSM 1740**	**1409**	**34557932**	**34557933**	**Type II-B**

Cluster[a]	Strain[b]	Cas9 length (aa)	Cas9 GI	Cas1 GI[c]	Subtype[d]
**48**	***gamma proteobacterium* HTCC5015**	**1397**	**254447899**	no *cas1*	**Type II-B**
**49**	***Filifactor alocis* ATCC 35896**	**1365**	**374307738**	**374307737**	**Type II-A**
**50**	***Planococcus antarcticus* DSM 14505**	**1333**	**389815359**	**389815358**	**Type II-A**
**51**	***Catenibacterium mitsuokai* DSM 15897**	**1329**	**224543312**	**224543313**	**Type II-A**
**52**	***Solobacterium moorei* F0204**	**1327**	**320528778**	**320528779**	**Type II-A**
**53**	***Fructobacillus fructosus* KCTC 3544**	**1323**	**339625081**	**339625080**	**Type II-A**
**54**	***Mycoplasma ovipneumoniae* SC01**	**1265**	**363542550**	**363542551**	**Type II-A**
54	*Streptobacillus moniliformis* DSM 12112	1259	269123826		
**55**	***Mycoplasma mobile* 163K**	**1236**	**47458868**	**47458867**	**Type II-A**
**56**	***Porphyromonas* sp. oral taxon 279 str. F0450**	**1197**	**402847315**	**402847305**	**Type II-C**
**57**	***Actinomyces* sp. oral taxon 180 str. F0310**	**1181**	**315605738**	**315605739**	**Type II-C**
**58**	***Sphaerochaeta globus* str. Buddy**	**1179**	**325972003**	**325972002**	**Type II-C**
**59**	***Rhodospirillum rubrum* ATCC 11170**	**1173**	**83591793**	**83591790**	**Type II-C**
**60**	***Azospirillum* sp. B510**	**1168**	**288957741**	**288957738**	**Type II-C**
**61**	***Nitrobacter hamburgensis* X14**	**1166**	**92109262**	no *cas1*	**Type II-C**
**62**	***Ruminococcus albus* 8**	**1156**	**325677756**	**325677757**	**Type II-C**
**63**	***Barnesiella intestinihominis* YIT 11860**	**1153**	**404487228**	**404487227**	**Type II-C**
**64**	***Alicyclobacillus hesperidum* URH17-3-68**	**1146**	**403744858**	**403744859**	**Type II-C**
**65**	***Acidothermus cellulolyticus* 11B**	**1138**	**117929158**	**117929157**	**Type II-C**
**66**	***Nitratifractor salsuginis* DSM 16511**	**1132**	**319957206**	**319957207**	**Type II-C**
**67**	***Acidovorax ebreus* TPSY**	**1131**	**222109285**	**222109284**	**Type II-C**
67	*Francisella tularensis* subsp. *tularensis* WY96-3418	1125	134302318		
**68**	***Lactobacillus coryniformis* subsp. *torquens* KCTC 3535**	**1119**	**336393381**	**336393380**	**Type II-C**
**69**	***Alcanivorax* sp. W11-5**	**1113**	**407803669**	**407803668**	**Type II-C**
**70**	***Akkermansia muciniphila* ATCC BAA-835**	**1101**	**187736489**	**187736488**	**Type II-C**
**71**	***Ilyobacter polytropus* DSM 2926**	**1092**	**310780384**	**310780383**	**Type II-C**
**72**	***Bradyrhizobium* sp. BTAi1**	**1064**	**148255343**	no *cas1*	**Type II-C**
**73**	***Ralstonia syzygii* R24**	**1062**	**344171927**	**344171926**	**Type II-C**
**74**	***Treponema* sp. JC4**	**1062**	**384109266**	**384109265**	**Type II-C**
**75**	***Wolinella succinogenes* DSM 1740**	**1059**	**34557790**	**34557789**	**Type II-C**
**76**	***Rhodovulum* sp. PH10**	**1059**	**402849997**	**402849996**	**Type II-C**
**77**	***Aminomonas paucivorans* DSM 12260**	**1052**	**312879015**	**312879014**	**Type II-C**
77	*Bacteroides* sp. 3_1_33FAA	1055	265750948		
**78**	***Parvibaculum lavamentivorans* DS-1**	**1037**	**154250555**	**154250554**	**Type II-C**
**79**	***Candidatus Puniceispirillum* marinum IMCC1322**	**1035**	**294086111**	**294086112**	**Type II-C**
80	*Blastopirellula marina* DSM 3645	1027	87307579		
**80**	***Helicobacter mustelae* 12198**	**1024**	**291276265**	**291276264**	**Type II-C**
**81**	***Clostridium cellulolyticum* H10**	**1021**	**220930482**	**220930481**	**Type II-C**
82	*Lactobacillus crispatus* FB077-07	857	423321767		
**82**	**uncultured delta proteobacterium HF0070_07E19**	**1011**	**297182908**	no *cas1*	**Type II-C**
	Acetobacter aceti NBRC 14818	240	340779894		
	Acetobacter aceti NBRC 14818	376	340779669		
	Acetobacter aceti NBRC 14818	400	340779439		
	Actinobacillus ureae ATCC 25976	239	322514756		
	Actinobacillus ureae ATCC 25976	400	322514772		
	Bacillus cereus BAG2X1-3	333	423408783		
	Bacteroides cellulosilyticus DSM 14838	206	224535831		
	Bacteroides cellulosilyticus DSM 14838	1219	224535832		
	Bacteroides coprosuis DSM 18011	349	333031028		
	Bacteroides oleiciplenus YIT 12058	653	427387687		
	Bacteroides oleiciplenus YIT 12058	779	427387686		
	Bacteroides sp. 9_1_42FAA	1055	237710146		
	Bacteroides uniformis CL03T12C37	286	423308124		
	Bacteroides uniformis CL03T12C37	1210	423308121		
	Bifidobacterium bifidum IPLA 20015	1281	421736922		
	Bifidobacterium dentium ATCC 27678	1121	171742822		
	Bifidobacterium longum subsp. *longum* 1-6B	182	419848319		
	Bifidobacterium longum subsp. *longum* 1-6B	354	419847807		
	Bifidobacterium longum subsp. *longum* 1-6B	441	419848320		
	Bifidobacterium longum subsp. *longum* 44B	166	419856168		

Cluster[a]	Strain[b]	Cas9 length (aa)	Cas9 GI	Cas1 GI[c]	Subtype[d]
	Bifidobacterium longum subsp. *longum* 44B	967	419856216		
	Butyrivibrio fibrisolvens 16/4	103	291518094		
	Butyrivibrio fibrisolvens 16/4	177	291518096		
	Butyrivibrio fibrisolvens 16/4	765	291518097		
	Campylobacter coli 2685	933	419548338		
	Campylobacter jejuni subsp. *jejuni* 2008-894	666	419652996		
	Campylobacter jejuni subsp. *jejuni* 305	190	317510779		
	Campylobacter jejuni subsp. *jejuni* 305	759	317510780		
	Campylobacter jejuni subsp. *jejuni* 327	462	415747744		
	Campylobacter jejuni subsp. *jejuni* 327	512	415747743		
	Campylobacter jejuni subsp. *jejuni* CG8421	721	205356639		
	Campylobacter jejuni subsp. *jejuni* M1	861	384442103		
	candidate division TM7 single-cell isolate TM7c	372	167957190		
	Capnocytophaga ochracea F0287	303	315224863		
	Capnocytophaga ochracea F0287	1117	315224862		
	Coprococcus comes ATCC 27758	686	226325213		
	Diplosphaera colitermitum TAV2	210	225164109		
	Enterococcus faecalis TX1467	921	422867931		
	Enterococcus faecalis TX4248	936	307270261		
	Enterococcus faecium E2620	892	431752788		
	Enterococcus sp. 7L76	116	295113136		
	Francisella tularensis subsp. *holarctica* 257	878	254367943		
	Francisella tularensis subsp. *holarctica* FSC022	158	254369498		
	Francisella tularensis subsp. *holarctica* FSC022	244	254369502		
	Francisella tularensis subsp. *holarctica* FSC022	292	254369497		
	Francisella tularensis subsp. *holarctica* FSC022	393	254369499		
	Francisella tularensis subsp. *holarctica* FSC022	501	254369496		
	Francisella tularensis subsp. *holarctica* LVS	158	89256630		
	Francisella tularensis subsp. *holarctica* LVS	393	89256631		
	Francisella tularensis subsp. *holarctica* URFT1	53	290953529		
	Francisella tularensis subsp. *holarctica* URFT1	285	290953528		
	Francisella tularensis subsp. *tularensis* SCHU S4	1123	56707712		
	Gemella haemolysans M341	1258	329766883		
	Haemophilus pittmaniae HK 85	121	343519651		
	Haemophilus pittmaniae HK 85	203	343519677		
	Haemophilus pittmaniae HK 85	650	343519679		
	Helicobacter hepaticus ATCC 51449	131	32266975		
	Helicobacter pullorum MIT 98-5489	344	242308998		
	Helicobacter pullorum MIT 98-5489	702	242309214		
	Kingella kingae ATCC 23330	1000	333374624		
	Lactobacillus buchneri ATCC 11577	1239	227512703		
	Lactobacillus casei 21/1	234	417984225		
	Lactobacillus casei 21/1	1128	417984226		
	Lactobacillus casei CRF28	566	417994652		
	Lactobacillus casei CRF28	700	417993346		
	Lactobacillus casei UW1	315	418005912		
	Lactobacillus casei UW1	330	418005913		
	Lactobacillus casei UW1	412	418005908		
	Lactobacillus casei UW4	236	418008739		
	Lactobacillus casei UW4	330	418008740		
	Lactobacillus crispatus 214-1	534	293381764		
	Lactobacillus crispatus CTV-05	298	312978192		
	Lactobacillus crispatus FB049-03	206	423318602		
	Lactobacillus crispatus FB049-03	347	423318603		
	Lactobacillus crispatus FB049-03	857	423318600		
	Lactobacillus crispatus JV-V01	278	227878395		
	Lactobacillus crispatus JV-V01	544	227878705		
	Lactobacillus crispatus MV-1A-US	277	256850790		
	Lactobacillus crispatus MV-1A-US	538	256850346		
	Lactobacillus crispatus MV-3A-US	279	262048056		
	Lactobacillus delbrueckii subsp. *bulgaricus* 2038	544	385815564		
	Lactobacillus delbrueckii subsp. *bulgaricus* 2038	669	385815562		

Cluster[a]	Strain[b]	Cas9 length (aa)	Cas9 GI	Cas1 GI[c]	Subtype[d]
	Lactobacillus iners LactinV 09V1-c	255	309804524		
	Lactobacillus iners LactinV 09V1-c	343	309804534		
	Lactobacillus iners LactinV 09V1-c	447	309804536		
	Lactobacillus iners SPIN 2503V10-D	270	309809475		
	Lactobacillus iners SPIN 2503V10-D	667	309809480		
	Lactobacillus ruminis ATCC 25644	1352	417973941		
	Lactobacillus salivarius ACS-116-V-Col5a	629	301299400		
	Lactobacillus salivarius CECT 5713	897	385839899		
	Lactobacillus salivarius UCC118	1149	90961083		
	Leptospira inadai serovar Lyme str. 10	125	398345609		
	Leptospira inadai serovar Lyme str. 10	418	398341884		
	Leptospira inadai serovar Lyme str. 10	907	398345610		
	Leuconostoc pseudomesenteroides 4882	468	399517481		
	Leuconostoc pseudomesenteroides 4882	883	399517482		
	Listeria ivanovii FSL F6-596	232	315301622		
	Listeria ivanovii FSL F6-596	849	315301624		
	Listeria monocytogenes FSL F2-208	782	422410878		
	Listeria monocytogenes FSL J1-208	300	255024093		
	Listeria seeligeri FSL N1-067	874	313631816		
	Listeria seeligeri FSL N1-067	874	422420175		
	Maritimibacter alkaliphilus HTCC2654	997	84685065		
	Mycoplasma iowae 695	226	350547050		
	Mycoplasma iowae 695	933	350546886		
	Neisseria lactamica ATCC 23970	408	269215119		
	Neisseria lactamica ATCC 23970	666	269215120		
	Neisseria lactamica Y92-1009	241	422110930		
	Neisseria lactamica Y92-1009	828	422110931		
	Neisseria meningitidis NM3001	67	421568320		
	Neisseria meningitidis NM3001	976	421568319		
	Neisseria mucosa C102	220	319639577		
	Neisseria sp. oral taxon 20 str. F0370	392	429743981		
	Neisseria sp. oral taxon 20 str. F0370	701	429743980		
	Neisseria subflava NJ9703	587	284799897		
	Nitritalea halalkaliphila LW7	79	390445315		
	Nitrobacter hamburgensis X14	641	92118334		
	Oribacterium sinus F0268	653	227873236		
	Parabacteroides merdae ATCC 43184	103	154493351		
	Parabacteroides merdae CL03T12C32	84	423346601		
	Parabacteroides merdae CL09T00C40	82	423723156		
	Pasteurella bettyae CCUG 2042	398	387770127		
	Pasteurella bettyae CCUG 2042	610	387770112		
	Pasteurella multocida subsp. *multocida* str. Anand1_buffalo	199	421253447		
	Pasteurella multocida subsp. *multocida* str. Anand1_cattle	53	421259752		
	Pasteurella multocida subsp. *multocida* str. Anand1_cattle	63	421259756		
	Pasteurella multocida subsp. *multocida* str. Anand1_cattle	134	421259749		
	Pediococcus acidilactici 7_4	1229	270290729		
	Pediococcus lolii NGRI 0510Q	270	427443367		
	Pediococcus lolii NGRI 0510Q	1016	427441502		
	Peptoniphilus sp. oral taxon 386 str. F0131	1341	299144352		
	Porphyromonas catoniae F0037	211	429741290		
	Porphyromonas catoniae F0037	1009	429741242		
	Prevotella denticola F0289	1218	327314511		
	Prevotella disiens FB035-09AN	443	303235616		
	Prevotella disiens FB035-09AN	795	303237415		
	Prevotella melaninogenica D18	1354	288802595		
	Prevotella multiformis DSM 16608	129	325268382		
	Prevotella multiformis DSM 16608	535	325268383		
	Prevotella oulorum F0390	691	345881543		
	Prevotella oulorum F0390	774	345881542		
	Prevotella saccharolytica F0055	242	429739781		
	Prevotella sp. oral taxon 317 str. F0108	593	288929745		
	Prevotella sp. oral taxon 317 str. F0108	1174	288930149		

Cluster[a]	Strain[b]	Cas9 length (aa)	Cas9 GI	Cas1 GI[c]	Subtype[d]
	Prevotella sp. oral taxon 472 str. F0295	241	260910968		
	Prevotella sp. oral taxon 472 str. F0295	992	260910970		
	Pseudoramibacter alactolyticus ATCC 23263	586	315926102		
	Pseudoramibacter alactolyticus ATCC 23263	770	315926103		
	Rhizobium etli GR56	103	218671711		
	Riemerella anatipestifer ATCC 11845 = DSM 15868	1145	383485594		
	Sphingobacterium spiritivorum ATCC 33300	116	227540450		
	Sphingobacterium spiritivorum ATCC 33300	1306	227540451		
	Staphylococcus massiliensis S46	475	425737243		
	Staphylococcus massiliensis S46	581	425737242		
	Staphylococcus simulans ACS-120-V-Sch1	1112	410878248		
	Streptococcus agalactiae 18RS21	773	76799343		
	Streptococcus downei F0415	994	312866154		
	Streptococcus dysgalactiae subsp. *equisimilis* SK1249	538	417753185		
	Streptococcus dysgalactiae subsp. *equisimilis* SK1250	1155	417926916		
	Streptococcus mutans SA38	1229	449253007		
	Streptococcus mutans SA38	1229	449880497		
	Streptococcus oralis SK255	550	417794716		
	Streptococcus oralis SK255	670	417793840		
	Streptococcus pseudoporcinus SPIN 20026	1326	313890160		
	Streptococcus pyogenes M49 591	1052	56808315		
	Streptococcus sanguinis VMC66	1167	323351495		
	Streptococcus sp. BS35b	93	401683465		
	Streptococcus sp. GMD4S	206	419816637		
	Streptococcus sp. GMD4S	317	419819606		
	Streptococcus thermophilus CNCM I-1630	302	418027683		
	Streptococcus thermophilus CNCM I-1630	595	418027684		
	Streptococcus thermophilus MTCC 5461	39	445389093		
	Streptococcus vestibularis F0396	97	312863468		
	Streptococcus vestibularis F0396	1038	312863582		
	Sutterella parvirubra YIT 11816	406	378822098		
	Sutterella parvirubra YIT 11816	951	378821855		
	Sutterella wadsworthensis 2_1_59BFAA	389	422348538		
	Tannerella sp. 6_1_58FAA_CT1	976	365118488		
	Treponema denticola ATCC 33520	631	449107910		
	Treponema denticola ATCC 33520	769	449107911		
	Treponema denticola F0402	357	422340642		
	Treponema denticola F0402	370	422340641		
	Treponema denticola F0402	631	422340640		
	Treponema phagedenis F0421	591	320536383		
	Treponema phagedenis F0421	738	320536384		
	Treponema vincentii ATCC 35580	281	257456747		
	Treponema vincentii ATCC 35580	992	257456748		
	uncultured bacterium	600	406975829		
	uncultured bacterium	1017	406999582		
	uncultured bacterium T3_7_42578	675	411001094		
	uncultured Termite group 1 bacterium phylotype Rs-D17	166	189485058		
	uncultured Termite group 1 bacterium phylotype Rs-D17	1032	189485225		
	Verminephrobacter aporrectodeae subsp. *tuberculatae* At4	983	347820874		

[a]Cas9 sequences are grouped according to the BLASTClust clustering program. Truncated sequences were not selected for the clustering and are listed at the bottom of the table without any cluster number (see Materials and Methods).
[b]Bacterial strains harboring a *cas9* orthologous gene are listed; GI, GenInfo Identifier. Bold, cluster representatives chosen for the alignment and tree reconstruction (Supplementary Figures S1 and S2). Grey, discarded, incomplete Cas9 sequences (see Materials and Methods). Note that the incomplete sequences were all confirmed to be truncated Cas9 orthologs based on the presence of conserved motifs and/or similarity to other Cas9 orthologs.
[c]Cas1 GenInfo Identifier of the representative sequences chosen for the alignment and tree reconstruction are given (Supplementary Figures S2 and S3). Grey, discarded, incomplete sequences. Whenever possible, an alternative Cas1 sequence from the same cluster as that of the discarded Cas1 sequence was selected (clusters 8, 9 and 21, in bold).
[d]Subtype of the CRISPR-Cas loci belonging to a same Cas9 cluster as inferred from the representative Cas1 and Cas9 tree topology.

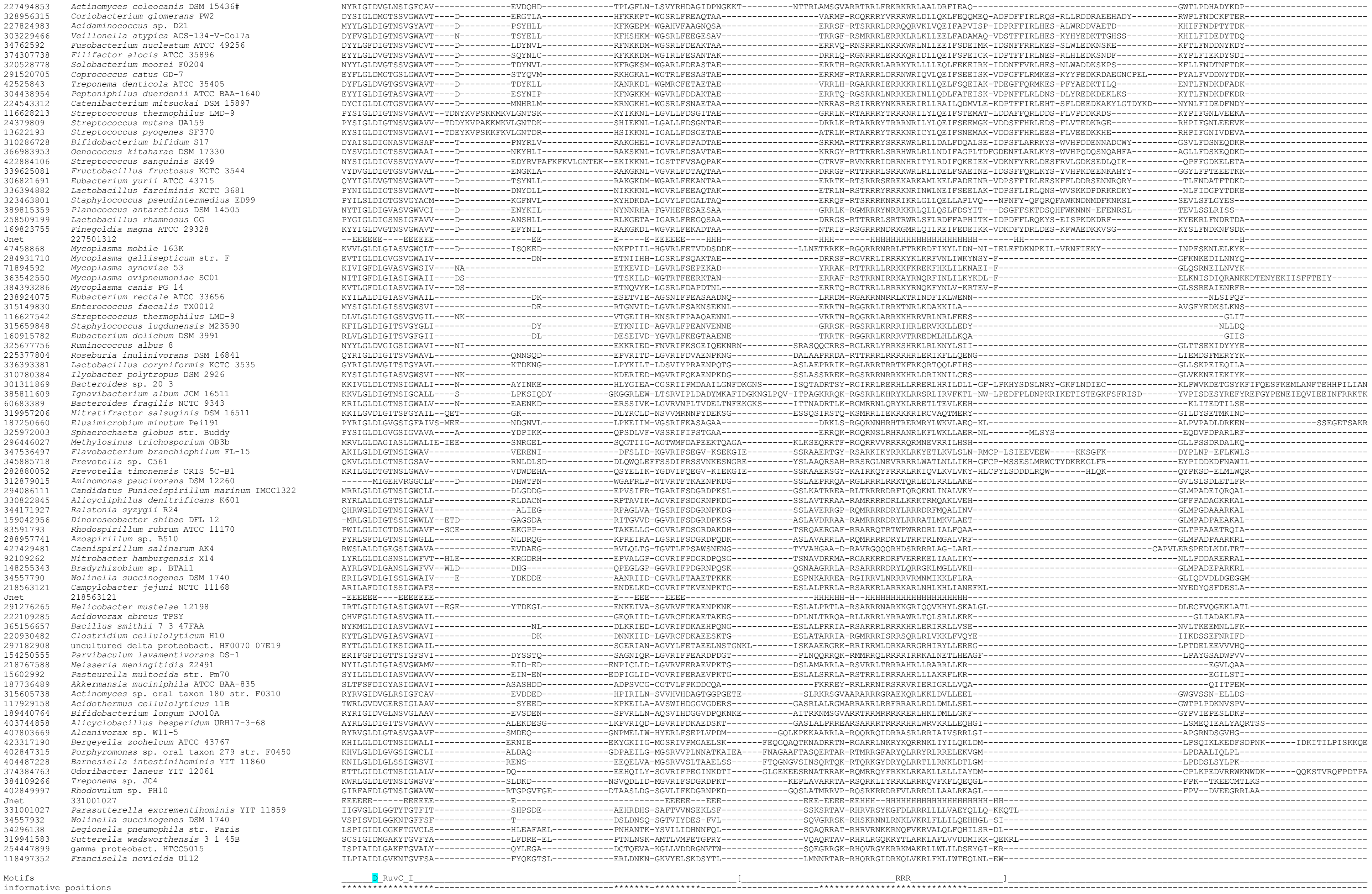

Supplementary Figure S2. Multiple sequence alignment of representative Cas9 sequences (see Supplementary Table S2 and Material and Methods). The rows described as Jnet with following GI identifier of a selected Cas9 sequence provide the predicted secondary structure of Cas9 within the corresponding subgroups (sequences indicated below each Jnet). Conserved motifs are marked below the alignment and the mutated amino acid residues are highlighted. Asterisks indicate informative positions chosen for the Cas9 tree reconstruction.

227494853	*Actinomyces coleocanis* DSM 15436#	--YTPWL---VRAELAQTP---IRDEN---DLH--------EKLAIAVRHIARHRGWRSPWVP--
328956315	*Coriobacterium glomerans* PW2	--------------------------------------DYYQRFPTIYH---VR-SWLM-----ETDEQ---ADI--------RLIYLALHNIVKHRGNFLREGQ--
227824983	*Acidaminococcus* sp. D21	--------------------------------------EYYSDYPTIHH---LI-VDLM-----ESSEK---HDP--------RLVYLAVAWLVAHRGHFLNEVD--
303229466	*Veillonella atypica* ACS-134-V-Col7a	--------------------------------------DYFTEYPTIYH---LR--KDL-----MENGT---DDI--------RKLFLAVHHILKYRGNFLYEGA--
34762592	*Fusobacterium nucleatum* ATCC 49256	--------------------------------------DFYKQYPTIFH---LR-DELI-----KNPEK---KDI--------RLIYLALHSIFKSRGHFLFEGQ--
374307738	*Filifactor alocis* ATCC 35896	--------------------------------------EYYKEFPTIFH---LR-KHLI-----ESEEK---QDI--------RLIYLALHNIIKTRGHFLIDGD--
320528778	*Solobacterium moorei* F0204	--------------------------------------DYLKKYPTIYH---LR-SDLI-----HNSTE---HDI--------RLVFLALHHLIKYRGHFIYDNS--
291520705	*Coprococcus catus* GD-7	--------------------------------------NYHKDYPTIYH---LR-KMLM-----ETTEI---PDI--------RLVYLVLHHMMKHRGHFLLSGD--
42525843	*Treponema denticola* ATCC 35405	--------------------------------------TYHKAYPTINH---LI-KAWI-----ENKVK---PDP--------RLLYLACHNIIKKRGHFLFEGD--
304438954	*Peptoniphilus duerdenii* ATCC BAA-1640	--------------------------------------DYHKKYPTIHH---LI-MDLI-----EDEGK---KDI--------RLLYLACHYLLKNRGHFIFEGQ--
224543312	*Catenibacterium mitsuokai* DSM 15897	--------------------------------------TYYHKYPTIYH---LR-KALC-----ESTEK---ADP--------RLIYLALHHIVKYRGNFLYEGQ--
116628213	*Streptococcus thermophilus* LMD-9	---------------------------------------YHDEFPTIYH---LR-KYLA-----DSTKK---ADL--------RLVYLALAHMIKYRGHFLIEGE--
24379809	*Streptococcus mutans* UA159	---------------------------------------YHENFPTIYH---LR-QYLA-----DNPEK---VDL--------RLVYLALAHIIKFRGHFLIEGK--
13622193	*Streptococcus pyogenes* SF370	---------------------------------------YHEKYPTIYH---LR-KKLV-----DSTDK---ADL--------RLIYLALAHMIKFRGHFLIEGD--
310286728	*Bifidobacterium bifidum* S17	---------------------------------------FYEKYPTIYH---LR-KALM-----EDDSQ---HDI--------REIYLAIHHMVKYRGNFLVEGT--
366983953	*Oenococcus kitaharae* DSM 17330	---------------------------------------FYRKYPTIYH---LR-LALM-----NDDQK---HDL--------REV-----HLVKYRGHFLIEGD--
422884106	*Streptococcus sanguinis* SK49	---------------------------------------YHKKYPTIYH---LR-KHLA-----DADKNSPVADI--------REVYMAISHILKYRGHFLTLDK--
339625081	*Fructobacillus fructosus* KCTC 3544	---------------------------------------FHRSYPTIYH---LR-QELM-----AQPNKR--FDI--------REIYLAIHHLVKYRGHFLSSQE--
306821691	*Eubacterium yurii* ATCC 43715	---------------------------------------YYEKYKTIFH---LR-SALI-----NSDEK---FDV--------RLVFLAILNLFSHRGHFLNASL--
336394882	*Lactobacillus farciminis* KCTC 3681	---------------------------------------YYREFPTIFH---LR-KELI-----LNKDK---ADI--------RLIYLALHNILKYRGNFTYEHQ--
323463801	*Staphylococcus pseudintermedius* ED99	---KKYPTIYH---LQ-EALL-----LKDEK---FDP--------ELIYMALYHLVKYRGHFLFDHL--
389815359	*Planococcus antarcticus* DSM 14505	---RKYPTIYH---LR-SDLI-----ESNKK---MDL--------RLVYLALHNLVKYRGHFLQEGN--
258509199	*Lactobacillus rhamnosus* GG	--------------------------------------EFYEDYPSMYH---LR-LHLM-----THTHK---ADP--------REIFLAIHHILKSRGHFLTPGA--
169823755	*Finegoldia magna* ATCC 29328	--------------------------------------QYFEKFPTIFH---LR-KYLM-----EEHGK---VDI--------RYYFLAINQMMKRRGHFLIDGQ--
Jnet	227501312	--------------------------------------HHH-----HHH---HH--HHH-----H---------H--------HHHHHHHHHHHHH---
47458868	*Mycoplasma mobile* 163K	--------------------------------------SVTNLPIGFHN---LR-KAAI-----NEKYK---LDK--------SELIVLLYFYLSLRGAFFDNPE--
284931710	*Mycoplasma gallisepticum* str. F	--------------------------------------EQQKLHNTVLN---LK-SEAL-------NAK---IDP--------KALSWILHDYLKNRGHFYEDNR--
71894592	*Mycoplasma synoviae* 53	--------------------------------------DQSSKYRNILK---LK-INAL-------KEE---IKP--------SELVWILRDYLQNRGYFYKN--
363542550	*Mycoplasma ovipneumoniae* SC01	-------------------------------------KKCAAKHSNILE---VK-VKAL-------DSK---IEK--------LDLIWILHDYLENRGFFYDLEEE---
384393286	*Mycoplasma canis* PG 14	--------------------------------------ELSIKYPNIIE---LK-TKAL-------SQE---VCP--------DEIAWILHDYLKNRGYFYDEKE--
238924075	*Eubacterium rectale* ATCC 33656	--KSTEIVG---LK-VRAI-------TEE---ITL--------DELYLILYSYLKHRGISYLEDALDDT--
315149830	*Enterococcus faecalis* TX0012	---CPYQ---LR-VKGL-------TEP---LSR--------GEIYKVTLHILKKRGISYLDEV--
116627542	*Streptococcus thermophilus* LMD-9	------------------------------------DFTKISINLNPYQ---LR-VKGL-------TDE---LSN--------EELFIALKNMVKHRGISYLDDAS---
315659848	*Staphylococcus lugdunensis* M23590	--------------------------------------SQIPQSTNPYA---IR-VKGL-------SEA---LSK--------DELVIALLHIAKRRGIHKIDVIDS--
160915782	*Eubacterium dolichum* DSM 3991	--------------------------------------TSFHPLNNPYD---VR-VKGL-------NER---LNG--------EELATALLHLCKHRGSSVETIEDDEAK---
325677756	*Ruminococcus albus* 8	---TADNNVIQ---LR-NKGL-------SEK---LTP--------EEIAACLIHICNNRGYKDFYEVNVEDIED---
225377804	*Roseburia inulinivorans* DSM 16841	---GNLPDVYQ---LR-YEGL-------DRK---LKD--------EELAQVLIHIAKHRGFRSTRKAETK---
336393381	*Lactobacillus coryniformis* KCTC 3535	---------------------------------------TPQAKYSVYE---LR-VAGL-------DRR---LTN--------SELFRVLYFFIGHRGFKSNRKAELNP--
310780384	*Ilyobacter polytropus* DSM 2926	--NAYLKSPWE---LR-AKSL-------EAK---ISN--------KEIAQILLHIAKRRGFKSFRKTDRN---
301311869	*Bacteroides* sp. 20 3	-------------------------------------NKKVPYDWTIYY---LR-KKAL-------TQK---ISK--------EELAWILLNFNQKRGYYQLRGE-EEETPNKLVEYYSLKVEKVEDSG--
385811609	*Ignavibacterium album* JCM 16511	GKNKNPMIKL------------------------------LPEDWVVYY---LR-KKAL-------IKP---TTK--------EELIRIIYLFNQRRGFKSSRKD-LTETA--ILDYDEFAKRLAEKEKYSAENYETKFVSITKVKEVVELK---
60683389	*Bacteroides fragilis* NCTC 9343	--------------------------------------NGNRTTFETYR---LR-AKAV-------TEE---ISL--------EEFARVLLMINKKRGYKSSRKAKGVEEGTLIDGMDIARELYNNNLTPGELCLQLLDAGKKFLPDFYRSDL--
319957206	*Nitratifractor salsuginis* DSM 16511	--------------------------------------PKNNPIKNRWQ---LRAVDAW-------KRP---LSP--------QELFAIFAHMAKHRGYKSIATE----------------------DLIYELELELGLNDPEKE--
187250660	*Elusimicrobium minutum* Pei191	-------------------------------------FLGDVLQKDIYE---LR-VKSL-------DER---LSL--------QELGYVLYHIAGHRGSSAIRTFENDSE---
325972003	*Sphaerochaeta globus* str. Buddy	--------------------------------------EDAVVRANPYE---LR-LKGL-------NEQ---LTL--------SELGYALYHIANHRGSSSVRTFLDEEK---
296446027	*Methylosinus trichosporium* OB3b	--PGLDPWR---IR-AEAL-------DRL---LGP--------VELAVALGHIARHRGFKSNSKGAKTNDP--
347536497	*Flavobacterium branchiophilum* FL-15	---------------------------------TDEESNVN-----PYF---FR-DRAS-------KHK---VSL--------FELGRAFYHIAQRRGFLSNRLDQSAEGILEEHCPKIEAIVEDLISIDEISTNITDYFFETGILD--
345885718	*Prevotella* sp. C561	---------------------------------LDFNGDGRPDYSSPYQ---LR-RELV-------TRQ---FDFEQPIER--YKLGRALYHIAQHRGFKSSKGETLSQQETNSKPSSTD---
282880052	*Prevotella timonensis* CRIS 5C-B1	---------------------------------TSDEEGKN-----PYY---DR-HRCL-------HEK---LDLTVEADR--YTLGRALYHLTQRRGFLSNRLDTSAD--
312879015	*Aminomonas paucivorans* DSM 12260	--ETPAKDPYQ---LR-AEGL-------DRP---LSF--------PEWVRVLYHITKHRGFQS--
294086111	*Candidatus Puniceispirillum marinum* IMCC1322	--AYKDPYP---IR-KKAL-------DEA---IDP--------YEMGRAIFHINQRRGFKS--
330822845	*Alicycliphilus denitrificans* K601	--EQLNPYA---LR-AKGL-------QEA---LLP--------GEFARALFHINQRRGFKS--
344171927	*Ralstonia syzygii* R24	--VTENPYV---LR-QRGL-------DQA---LTL--------PEFGRALFHLNQRRGFQS--
159042956	*Dinoroseobacter shibae* DFL 12	--EALDPFA---LR-AAGL-------DEP---LPL--------PHLGRALFHLNQRRGFKS--
83591793	*Rhodospirillum rubrum* ATCC 11170	---LA---LR-REAV-------SRP---LAP--------DALWAALLHLAHHRGFRS--
288957741	*Azospirillum* sp. B510	---EVAVDPYL---AR-ERAT-------RER---LEP--------FEIGRALFHLNQRRGYKP--
427429481	*Caenispirillum salinarum* AK4	---PPKADPRAIFFLR-ADAA-------RRP---LDG--------PELFRVLHHMAAHRGIRLAELQ--
92109262	*Nitrobacter hamburgensis* X14	--EVLDPYA---LR-KTAL-------TDT---LPA--------HHVGRALFHLNQRRGFQS--
148255343	*Bradyrhizobium* sp. BTAi1	--ECLDPYG---LR-AKAL-------DEV---LPL--------HHVGRALFHLNQRRGLFA--
34557790	*Wolinella succinogenes* DSM 1740	-------------------------------------FYSKANRADVWE---LR-HDGL-------YRL---LKG--------DELARVLIHIAKHRGYKF--
218563121	*Campylobacter jejuni* NCTC 11168	-------------------------------------KAYKGSLISPYE---LR-FRAL-------NEL---LSK--------QDFARVILHIAKRRGYDD--
Jnet	218563121	--HHH---HH-HHHH-------HH---------------HHHHHHHHHHHHH---
291276265	*Helicobacter mustelae* 12198	-------------------------------------FQTSKDFLSPWE---LR-ERAL-------YRV---LDK--------EELARVILHIAKRRGYDD--
222109285	*Acidovorax ebreus* TPSY	--------------------------------------KAPSYGDSAWE---LR-RQGL-------DRL---LTP--------LEWARVIYHQCKHRGFHW--
365156657	*Bacillus smithii* 7 3 47FAA	--QKKQIDVWQ---LR-VDAL-------ERK---LNN--------DELARVLLHLAKRRGFKS--
220930482	*Clostridium cellulolyticum* H10	--------------------------------------TSRDGWKDPWE---LR-YNAL-------SRI---LKP--------YELVQVLTHITKRRGFKS--
297182908	uncultured delta proteobact. HF0070 07E19	--SNRTLWD---VR-AEAV-------ERK---LTK--------QELAAVLFHLVRHRGYFP--
154250555	*Parvibaculum lavamentivorans* DS-1	--MADEPYE---LR-RRGL-------EEG---LSA--------YEFGRAIYHLAQHRHFKG--
218767588	*Neisseria meningitidis* Z2491	--------------------------------DFDENGLIKSLPNTPWQ---LR-AAAL-------DRK---LTP--------LEWSAVLLHLIKHRGYLS--
15602992	*Pasteurella multocida* str. Pm70	-------------------------------------DLEKGLPNQAWE---LR-VAGL-------ERR---LSA--------IEWGAVLLHLIKHRGYLS--
187736489	*Akkermansia muciniphila* ATCC BAA-835	--------------------------------------KETSGHPAPFY---LA-SEAL-----KGHRT---LAP--------IELWHVLRWYAHNRGYDN--
315605738	*Actinomyces* sp. oral taxon 180 str. F0310	--HAPWH---IRKRLVSEY---IEDET---ERR--------QCLSVAMAHIARHRGWRNSFSK--
117929158	*Acidothermus cellulolyticus* 11B	---DAWL---ARKRLAEEY---VVDET---ERR--------RLLGYAVSHMARHRGWRNPWTT--
189440764	*Bifidobacterium longum* DJO10A	--FEEWH---VRAELATRY---IEDDE---LRR--------ESISIALRHMARHRGWRN--
403744858	*Alicyclobacillus hesperidum* URH17-3-68	--PDVYA---LRVAGLD--------RC---LIA--------EEIARVLIHIAHRRGFQS--
407803669	*Alcanivorax* sp. W11-5	--NDVPT---LRAMAVN--------ER---IEL--------GQLRAVLLRMGKKRGYGG--
423317190	*Bergeyella zoohelcum* ATCC 43767	QLTA--FDLVS---LRVKALT--------EK---VGL--------EDLGKIIYKYNQLRGYAGGSLEPEKEDIFDEEQSKDKKNKSFIAFSKIVFLGEPQEEIFK--
402847315	*Porphyromonas* sp. oral taxon 279 str. F0450	--LELWE---LRERAATAG------RR---LTL--------PELGRVLCHINQKRGYRH--
404487228	*Barnesiella intestinihominis* YIT 11860	--LQLWG---LRAKAVT--------QR---IEL--------NELGRVLLHLNQKRGYKS--
374384763	*Odoribacter laneus* YIT 12061	FREWLK--------------------------------------QNPYE---LRKQAVT--------ED---VTR--------PELGRILYQMIQRRGFLS--
384109266	*Treponema* sp. JC4	--LNPYE---LRIKALD--------EK---LEP--------YELGRALFNLAVRRGFKS--
402849997	*Rhodovulum* sp. PH10	--TDPYH---LRAKALD--------ES---LTP--------HEMGRVIFHLNQRRGFRS--
Jnet	331001027	---HH-------HHHHHHHHHHHHH----EEE---------------------H---------------------------HHHHHHHH----------------HHHHHH---------HH---------
331001027	*Parasutterella excrementihominis* YIT 11859	---APEE-------RENLRIALSGYLKRRGYARTEAE---------------TDTSVLESLDPSVFSSAPSFTNFFN--DSEPLNIQWEAIANSPE------------TTKALN--------KELSGQK----
34557932	*Wolinella succinogenes* DSM 1740	---DVLPDEIRGLFNKRGYTYAGFE---------------LDEKKKDALESDTL--KEFLSEKLQSIDRDSDVEDFLNQIAS--------------NAESFK---------DYKKGF----
54296138	*Legionella pneumophila* str. Paris	--NAKEETALCHYLNNRGYTYVDTD---------------LDEYIKDETTINLL--KELLPS-----ESEHNFIDW--------------------FLQKMQ-------------------
319941583	*Sutterella wadsworthensis* 3 1 45B	---TDEE-------WKRGREALSGLLKRRGYSRPNADG--------------EDLTPLENVRADVFAAHPAFSTYFS--EVRSLAEQWEEFTANIS------------NVEKFL--------GDPNIPA----
254447899	gamma proteobact. HTCC5015	--EEV-------TEPLLKFINGLLNRRGYTYI------------------SEEVDEESMNVSPLPFSEMMPDYFN--SSAPLLEQLAKLLSDKN------------KLVRFR--------AEGKIPS----
118497352	*Francisella novicida* U112	--DKDTQQAISFLFNRRGFSFI------------------TDGYSPEYLNIVPEQVKAILMDIFDDYNGEDDLDSYLKLATEQESKISEIYNKLMQKILEFKLMKLCTDIKDDKVSTKTLK
Motifs		____E___________R____
informative positions		---******************--

```
227494853    Actinomyces coleocanis DSM 15436#              ----------VRSLHVEQPP-------------------------------------------------------------------------------------------------------------------SDQYLALK----------------ERVEAKTLLQMPEGATPAEMV----------VALDLSVDVN
328956315    Coriobacterium glomerans PW2                   ----------------SLSAKSARPDEALNHLRETLRVWSSERGFECSIADNGSILAMLTHPDLSPSDRRKKIAPLFDVKSDDAAADKKLGIALAGAVIGLKTEFKNIFGDFPCEDSSIYLS-------------NDEAVDAVRSACPDD---------CAELFDRLCEVYSAYVLQGLL-----SYAPGQTISANMVEK
227824983    Acidaminococcus sp. D21                        ------------------KDNIGDVLSFDAFYPEFLAFLSDNGVSPWVCESKALQATLLSRNSVNDKYKALKSLIFGSQKPEDNFDANISEDGLIQLLAGKKVKVNKLFPQESNDASFTL---------------NDKEDAIEEI--LGTLTPD-----ECEWIAHIRRLFDWAIMKHAL-------KDGRTISESKVKL
303229466    Veillonella atypica ACS-134-V-Col7a            ----------TFNSNAF-TFEDVLKQALVNITFNCFDTNSAISSISNILMESGKTKSDKAKAIERLVDTYTVFDEVNTPDKPQKEQVKEDKKTLKAFANLVLGLSANLIDLFGSVEDIDDDLKKLQ--IVGDTY-DEKRDELAKV--WGD---------EIHIIDDCKSVYDAIILMSIK-------EPGLTISQSKVKA
34762592     Fusobacterium nucleatum ATCC 49256           -----------------NLKEIKNFETLYNNLISFLEDNGINKSIDKDNIEKLEKIICDSGKGLKDKEKEFKGIFNSDKQLVAIFKLSVGSSVSLNDLFDTDEYKKEEVEKEKISFREQIY--------------EDDKPIYYSI--LGE---------KIELLDIAKSFYDFMVLNNIL-------SDSNYISEAKVKL
374307738    Filifactor alocis ATCC 35896                   -----------------LQSAKQLRPILDTFLLSLQEEQNLSVSLSENQKDEYEEILKNRSIAKSEKVKKLKNLFEISDELEKEEKKAQSAVIENFCKFIVGNKGDVCKFLRVSKEELEIDSFSFSEGKYE----DDIVKNLEEK--VPE---------KVYLFEQMKAMYDWNILVDIL-------ETEEYISFAKVKQ
320528778    Solobacterium moorei F0204                     -----------------ANGDVKTLDEAVSDFEEYLNENDIEFNIENKKEFINVLSDKHLTKKEKKISLKKLYGDITDSENINISVLIEMLSGSSISLSNLFKDIEFDGKQNLSLDSDI----------------EETLNDVVDI--LGD---------NIDLLIHAKEVYDIAVLTSSL-------GKHKYLCDAKVEL
291520705    Coprococcus catus GD-7                         ------------------ISQIKEFKSTFEQLIQNIQDEELEWHISLDDAAIQFVEHVLKDRNLTRSTKKSRLIKQLNAKSACEKAILNLLSGGTVKLSDIFNNKELDESERPKVSFADSGY-------------DDYIGIVEAE--LAE---------QYYIIASAKAVYDWSVLVEIL-------GNSVSISEAKIKV
42525843     Treponema denticola ATCC 35405               -----------------FDSENQFDTSIQALFEYLREDMEVDIDADSQKVKEILKDSSLKNSEKQSRLNKILGLKPSDKQKKAITNLISGNKINFADLYDNPDLKDAEKNSISFSKDDF----------------DALSDDLASI--LGD---------SFELLLKAKAVYNCSVLSKVI-------GDEQYLSFAKVKI
304438954    Peptoniphilus duerdenii ATCC BAA-1640          ----------KFDTKNSFDKSINDLKIHLRDEYNIDLEFNNEDLIEIITDTTLNKTNKKKELKNIVGDTKFLKAISAIMIGSSQKLVDLFEDGEFEETTVKSVDFSTTAF-------------------------DDKYSEYEEA--LGD---------TISLLNILKSIYDSSILENLLKDADKSKDGNKYISKAFVKK
224543312    Catenibacterium mitsuokai DSM 15897            ----------KFNMDASNIEDKLSDIFTQFTSFNNIPYEDDEKKNLEILEILKKPLSKKAKVDEVMTLIAPEKDYKSAFKELVTGIAGNKMNVTKMILCEPIKQGDSEIKLKFSDSNY-----------------DDQFSEVEKD--LGE---------YVEFVDALHNVYSWVELQTIM---GATHTDNASISEAMVSR
116628213    Streptococcus thermophilus LMD-9              -----------------FNSKNNDIQKNFQDFLDTYNAIFESDLSLENSKQLEEIVKDKISKLEKKDRILKLFPGEKNSGIFSEFLKLIVGNQADFRKCFNLDEKASLHFSKESY--------------------DEDLETLLGY--IGDD--------YSDVFLKAKKLYDAILLSGFL--TVTDNETEAPLSSAMIKR
24379809     Streptococcus mutans UA159                     -----------------FDTRNNDVQRLFQEFLAVYDNTFENSSLQEQNVQVEEILTDKISKSAKKDRVLKLFPNEKSNGRFAEFLKLIVGNQADFKKHFELEEKAPLQFSKDTY--------------------EEELEVLLAQ--IGDN--------YAELFLSAKKLYDSILLSGIL--TVTDVGTKAPLSASMIQR
13622193     Streptococcus pyogenes SF370                   -----------------LNPDNSDVDKLFIQLVQTYNQLFEENPINASGVDAKAILSARLSKSRRLENLIAQLPGEKKNGLFGNLIALSLGLTPNFKSNFDLAEDAKLQLSKDTY--------------------DDDLDNLLAQ--IGDQ--------YADLFLAAKNLSDAILLSDIL--RVNTEITKAPLSASMIKR
310286728    Bifidobacterium bifidum S17                    ----------LESSNAFKEDELLKLLGRITRYEMSEGEQNSDIEQDDENKLVAPANGQLADALCATRGSRSMRVDNALEALSAVNDLSREQRAIVKAIFAGLEGNKLDLAKIFVSKEFSSENKKILGIYFNKSDYEEKCVQIVDSGLLDDE--------EREFLDRMQGQYNAIALKQLL-------GRSTSVSDSKCAS
366983953    Oenococcus kitaharae DSM 17330                 ----------VKADSAF---DVHTFADAIQRYAESNNSDENLLGKIDEKKLSAALTDKHGSKSQRAETAETAF--DILDLQSKKQIQAIL------KSVVGNQANLMAIFGLDSSAISKDEQKNYKFSFDDADI-DEKIADSEAL--LSDT--------EFEFLCDLKAAFDGLTLKMLL-------GDDKTVSAAMVRR
422884106    Streptococcus sanguinis SK49                   --------INP--NNINMQNSWIDFI------ESCQEVFDLEISDES---------KNIADIFKSSE-NRQEKVKKILPYFQ--QELLKKDKSIFKQLLQLLFGLKTKFKDCFELEEEPD--------LNFSKENYDENLENFL--GSLEED-------FSDVFAKLKVLRDTILLSGMLTY---TGATHARFSATMVER
339625081    Fructobacillus fructosus KCTC 3544             --------KIT-IGSTYNPEDLANAI------EVYADEKGLSWELNNPEQL-----TEIISGEAGYGLNKSMKADEALKLFE---FDNNQDKVAIKTLLAGLTGNQIDFAKLFG-KDISDKDEAKLWKLKLDDEALEEKSQTIL--SQLTDE-------EIELFHAVVQAYDGFVLIGLL-------NGADSVSAAMVQL
306821691    Eubacterium yurii ATCC 43715                   --------KGD--GDIQGMDVFYN--------DLVESCEYFEIELPRITNI-----DNFEKILSQKGKSRTKILEELSEELS----ISKKDKSKYN-LIKLISGLEASVVELYNIEDIQDENKKI--KIGFRESDYEESSLKVK--EIIGDE-------YFDLVERAKSVHDMGLLSNII-------GNSKYLCEARVEA
336394882    Lactobacillus farciminis KCTC 3681             --------KFN--ISNLNNNLSKELI------ELNQQLIKYDISFPDDCDW-----NHISDILIGRG-NATQKSSNILKDFT----LDKETKKLLKEVINLILGNVAHLNTIFKTSLTKDEEK-----LNFSGKDIESKLDDLD--SILDDD-------QFTVLDAANRIYSTITLNEIL-------NGESYFSMAKVNQ
323463801    Staphylococcus pseudintermedius ED99           --------KIENLTNNDNMHDFVELI------ETYENLNNIKLNLDYEKT------KVIYEILKDNEMTKNDRAKRV-----------KNMEKKLEQFSIMLLGLKFNEGKLFNHADNAEELKGANQSHTFAD-NYEENLT-----PFLTVE-------QSEFIERANKIYLSLTLQDIL-------KGKKSMAMSKVAA
389815359    Planococcus antarcticus DSM 14505             --------WSEAASAEGMDDQLLELV------TRYAELENLSPLDLSESQW-----KAAETLLLNRNLTKTDQSKELTAMFG----------KEYEPFCKLVAGLGVSLHQLFPSSEQALAYKETKTKVQLSNENVEEVM------ELLLEE-------ESALLEAVQPFYQQVVLYELL-------KGETYVAKAKVSA
258509199    Lactobacillus rhamnosus GG                     ----------AKDFNTDKVDLEDIFPALTEAYAQVYPDLELTFDLAKADDFKAKLLDEQATPSDTQKALVNLLLSSDGEKEIVKKRKQVLTEFAKAITGLKTKFNLALGTEVDEADASNWQFSMGQL--------DDKWSNIETS--MTDQ--------GTEIFEQIQELYRARLLNGIV-------PAGMSLSQAKVAD
169823755    Finegoldia magna ATCC 29328                    ----------ISHVTDDKPLKEQLILLINDLLKIELEEELMDSIFEILADVNEKRTDKKNNLKELIKGQDFNKQEGNILNSIFESIVTGKAKIKNIISDEDILEKIKEDNKEDFVLTGDSY--------------EENLQYFEEV--LQE---------NITLFNTLKSTYDFLILQSIL-------KGKSTLSDAQVER
Jnet         227501312                                      ----------------HHHHHHHHHHHHHH---------HHHHH-HHHHH----HHHHHHHHHHHHH------HHHHHHH----HHH----E----------------------------H--------------HHHHHHHHH---------------HHHHHHHHHHHHHHHHH---------------HHHHHHHHH
47458868     Mycoplasma mobile 163K                         ---------------------------------------------------------------------------------------------------------------------------------------DTKSKEMNKN--------------EIEIFDKNESIKNAEFPIDKI-----------------IEF
284931710    Mycoplasma gallisepticum str. F                -------------------------------------------------------------------------------------------------------------------------------------------DF---------------------------NVYPTKELAKYF---------------DKYGY
71894592     Mycoplasma synoviae 53                         ---------------------------------------------------------------------------------------------------------------------------------------EKLTDEFVSNSF------------PSKKLHEHYEKYGFFRGSVKL-----------------DNK
363542550    Mycoplasma ovipneumoniae SC01                  ----------------------------------------------------------------------------------------------------------------------------------------NVADKYEGIEH------------PSILLYDFFKKNGFFKSNSSI--------------------
384393286    Mycoplasma canis PG 14                         -----------------------------------------------------------------------------------------------------------------------------------------TKEDFDQQTVESM---------PSYKLNEFYKKYGYFKGALSQ--------------------
238924075    Eubacterium rectale ATCC 33656                 ---------------------------------------------------------------------------------------------------------------------------------------VSGSSAYANG--------------LKLNAKELETHYPCEIQQERL---------------NTIGK
315149830    Enterococcus faecalis TX0012                   ---------------------------------------------------------------------------------------------------------------------------------------DTEAAKESQD--------------YKEQVRKNAQLLTKYTPGQIQ-----------------LQR
116627542    Streptococcus thermophilus LMD-9              ---------------------------------------------------------------------------------------------------------------------------------------DDGNSSVGD---------------YAQIVKENSKQLETKTPGQIQ-----------------LER
315659848    Staphylococcus lugdunensis M23590              ---------------------------------------------------------------------------------------------------------------------------------------NDDVGNELS---------------TKEQLNKNSKLLKDKFVCQIQ-----------------LER
160915782    Eubacterium dolichum DSM 3991                  ---------------------------------------------------------------------------------------------------------------------------------------AKEAGETKKV--LSM---------NDQLL------KSGKYVCEIQ-----------------KER
325677756    Ruminococcus albus 8                           ---------------------------------------------------------------------------------------------------------------------------------------PDERNEYKEE---HD---------SIVLISNLMNEGGYCTPAEMI---------------CNCRE
225377804    Roseburia inulinivorans DSM 16841              ---------------------------------------------------------------------------------------------------------------------------------------EKEGGAVLKA--TTE---------NQKIM----QEKGYRTVGEML--------------YLDEAF
336393381    Lactobacillus coryniformis KCTC 3535           ---------------------------------------------------------------------------------------------------------------------------------------ENEADKKQMGQLLN----------SIEEIRKAIAEKGYRTVGELY---------------LKDPK
310780384    Ilyobacter polytropus DSM 2926                 ---------------------------------------------------------------------------------------------------------------------------------------ADDTGKLLSG--IQE---------NKKIM----EEKGYLTIGDMV---------------AKDPK
301311869    Bacteroides sp. 20 3                           ---------------------------------------------------------------------------------------------------------------------------------------ERKGKDTWYNVHLENGM-------IYRRTSNIPLDWEGKTKEFIV-----------------TTD
385811609    Ignavibacterium album JCM 16511                -------------------------------------------------------------------------------------------------------------------------------------TDGRKGKKR-FKVILEDSR-----IEPYEIERKEKPDWEGKEYTFLV-----------------TQK
60683389     Bacteroides fragilis NCTC 9343                 ---------------------------------------------------------------------------------------------------------------------------------------QNELDRIWEK--QKEYYPEILTDVLKEELRGKKRDAVWAICAKYF---------------VWKEN
319957206    Nitratifractor salsuginis DSM 16511            ---------------------------------------------------------------------------------------------------------------------------------------SEKKADERRQ--VYN---------ALRHLEELRKKYGGETIAQTI---------HRAVEAGDLRS
187250660    Elusimicrobium minutum Pei191                  ---------------------------------------------------------------------------------------------------------------------------------------EAQKENTENKKIAGN---------IKRLM----AKKNYRTYGEYL-----------------YKE
325972003    Sphaerochaeta globus str. Buddy                ---------------------------------------------------------------------------------------------------------------------------------------SSDDKKLEE---------------QQAMTEQLAKEKGISTFIEVL------------------TA
296446027    Methylosinus trichosporium OB3b                ---------------------------------------------------------------------------------------------------------------------------------------ADDTSKMKRA--VNE---------TREKLARFGSAAKMLVEDESF-----------------VLR
347536497    Flavobacterium branchiophilum FL-15            ---------------------------------------------------------------------------------------------------------------------------------------SNEKNGYAKD--LDEGDKKLVSLYKSLLAILKKNESDFENCKSEI--------------IERLNK
345885718    Prevotella sp. C561                            ------------------------------------------------------------------------------------------------------------------------------------------------------------------------------------------------------EI
282880052    Prevotella timonensis CRIS 5C-B1               ------------------------------------------------------------------------------------------------------------------------------------------------------------------------------------------------------NK
312879015    Aminomonas paucivorans DSM 12260               -------------NRRNPVEDGQERSR------------------------------------------------------------------------------------------------------------QEEEGKLLSG--VGE---------NERLL----REGGYRTAGEML---------------ARDPK
294086111    Candidatus Puniceispirillum marinum IMCC1322   -------------NRKSA---------------------------------------------------------------------------------------------------------------------DNEAGVVKQ---------------SIADLEMKLGEAGARTIGEFL-----------------ADR
330822845    Alicycliphilus denitrificans K601              -------------NRKTDKK-------------------------------------------------------------------------------------------------------------------DNDSGVLKKA--IGQ---------LRQQMAEQGS----RTVGEYL-----------------WTR
344171927    Ralstonia syzygii R24                          -------------NRKT-DRAT-----------------------------------------------------------------------------------------------------------------AKESGKVKNA--IAA---------FRAGM------GNARTVGEAL-----------------ARR
159042956    Dinoroseobacter shibae DFL 12                  -------------NRKTDRG-------------------------------------------------------------------------------------------------------------------DNESGKIKD---------------ATARLDMEMMANGARTYGEFL-----------------HKR
83591793     Rhodospirillum rubrum ATCC 11170              -------------NRIDKRERAAAKALAKAKPAKATAKATAPAKE------------------------------------------------------------------------------------------ADDEAGFWEG--------------AEAALRQRMAASGAPTVGALL-----------------ADD
288957741    Azospirillum sp. B510                          -------------VRTATKP-------------------------------------------------------------------------------------------------------------------DEEAGKVKE---------------AVERLEAAIAAAGAPTLGAWF-----------------AWR
427429481    Caenispirillum salinarum AK4                   --------------EVDPPP-------------------------------------------------------------------------------------------------------------------ESDADDAAP---------------AATEDEDGTRRAAADER---------------------AFR
92109262     Nitrobacter hamburgensis X14                   -------------NRKT-DSK------------------------------------------------------------------------------------------------------------------QSEDGAIKQA--------------ASRLATDKGNETLGVFFADMH----------------LRKS
148255343    Bradyrhizobium sp. BTAi1                       -------------NRAI-EQG------------------------------------------------------------------------------------------------------------------DKDASAIKAA--AGR---------LQTSM----QACGARTLGEFL---------------NRRHQ
34557790     Wolinella succinogenes DSM 1740                -------------IGDDEA--------------------------------------------------------------------------------------------------------------------DEESGKVKKA---------------GVVLRQNFEAAGCRTVGEWL-----------------WRE
218563121    Campylobacter jejuni NCTC 11168                -------------IKNS-D--------------------------------------------------------------------------------------------------------------------DKEKGAILKA--IKQ---------NEEKL------ANYQSVGEYL-------------YKEYFQK
Jnet         218563121                                      -----------------------------------------------------------------------------------------------------------------------------------------HHHHHHHH--HHH---------HHHHH------H----HHHHH-------------HHHHHHH
291276265    Helicobacter mustelae 12198                    -------------ITYGVE--------------------------------------------------------------------------------------------------------------------DNDSGKIKKA--IAE---------NSKRI----KEEQCKTIGEMM-----------------YKL
222109285    Acidovorax ebreus TPSY                         -------------TSKAEEAKADS---------------------------------------------------------------------------------------------------------------DAEGGRVKQG--LAH---------TKALM----QAKNYRSAAEMV-----------------LAE
365156657    Bacillus smithii 7 3 47FAA                     -------------NRKSE-------------------------------------------------------------------------------------------------------------------RNSKESSEFLKN--IEE---------NQSIL------AQYRSVGEMI---------------VKDSK
220930482    Clostridium cellulolyticum H10                 -------------NRKE-DLS------------------------------------------------------------------------------------------------------------------TTKEGVVITS--IKN---------NSEML----RTKNYRTIGEMI-----------------FME
297182908    uncultured delta proteobact. HF0070 07E19      -------------NTKK-LPP-----------------------------------------------------------------------------------------------------------DDESDSADEEQGKINRA--TSR---------LREEL----KASDCKTIGQFL--------------------
154250555    Parvibaculum lavamentivorans DS-1              --------------RELEESDTPD---------------------------------------------------------------------------------------------------------------PDVDDEKEAA--------------NERAATLKALKNEQTTLGAWL-----------------ARR
218767588    Neisseria meningitidis Z2491                   -------------QRKNE-GETAD---------------------------------------------------------------------------------------------------------------K-ELGALLKG--VAD---------NAHAL----QTGDFRTPAELA--------------LNKFEK
15602992     Pasteurella multocida str. Pm70                -------------KRKNE-SQTNN---------------------------------------------------------------------------------------------------------------K-ELGALLSG--VAQ---------NHQLL----QSDDYRTPAELA--------------LKKFAK
187736489    Akkermansia muciniphila ATCC BAA-835           -------------NASWSNSL------------------------------------------------------------------------------------------------------------------SEDGGNGEDT---------ERVKHAQDLMDKHGTATMAETICREL-------------------K
315605738    Actinomyces sp. oral taxon 180 str. F0310      ----------------VDTLLLEQAP-------------------------------------------------------------------------------------------------------------SDRMQGLK----------------ERVEDRTGLQFSEEVTQGELV---------ATLLEHDGDVT
117929158    Acidothermus cellulolyticus 11B                ----------------IKDLKNLPQP-------------------------------------------------------------------------------------------------------------SDSWERTRESLEARY---------SVSLEPGTVGQWAGYLLQRAP-----------------GIR
189440764    Bifidobacterium longum DJO10A                  -------------PYRQVDSLISDNPY------------------------------------------------------------------------------------------------------------SKQYGELKEK--------------AKAYNDDATAAEEESTPAQLV-----------------VAM
403744858    Alicyclobacillus hesperidum URH17-3-68         -------------NRK-------SEIK------------------------------------------------------------------------------------------------------------DSDAGKLLKA------VQENE-NLMQ--------SKGYRTVAEML--------------VSEATK
407803669    Alcanivorax sp. W11-5                          -------------TFKAVRKVGEAGEV------------------------------------------------------------------------------------------------------------ASGASRLEEE------MVAL--ASVQ--------NKDSVTVGEYLAARVEHGLPSKLKVAANNEY
423317190    Bergeyella zoohelcum ATCC 43767                -------------NKKLNRRAIIVETE------------------------------------------------------------------------------------------------------------EGNFEGSTFL----ENIKVG--DSLEL-----LINISASKSGDTITIKLPNKTNWRKKMENIENQ
402847315    Porphyromonas sp. oral taxon 279 str. F0450    -------------VKSDAAAIVGDEGE------------------------------------------------------------------------------------------------------------KKKDSNSAYL----AGIRAND-EKLQ---------AEHKTVGQYF--------------------
404487228    Barnesiella intestinihominis YIT 11860         -------------IKS-------DFSG------------------------------------------------------------------------------------------------------------DKKITDYVKT------VKTRY-DELK---------EMRLTIGELF--------------------
374384763    Odoribacter laneus YIT 12061                   -------------SRK-----------------------------------------------------------------------------------------------------------------------GKEEGKIFTG------KDRMV-GIDET-----RKNLQKQTLGAYL--------------------
384109266    Treponema sp. JC4                              -------------NRKDGSREEVSEKK------------------------------------------------------------------------------------------------------------SPDEIKTQAD------MQTHLEKAIK--------ENGCRTITEFL--------------------
402849997    Rhodovulum sp. PH10                            -------------NRK-------ADRQ------------------------------------------------------------------------------------------------------------DREKGKIAEG------SKRLA-ETLA--------ATNCRTLGEFL--------------------
Jnet         331001027                                      -----------------------------------------------HHHH--------HHH-HHE---------------------------HHHHHHHHHHHHHHHHHHHHHHHHHHH-----HHHHHHHHHHHHHHHHHHHHHHHH----------------------HHHHHH------------------HHHHH
331001027    Parasutterella excrementihominis YIT 11859     -----------------------------------------------EADF--------KKY-IKTSFPEYS---------------------AKEILANYVEGRRAILDASKYIANLQSLGHKHRSKYLSDILQDMKRDSRITRLSEAFG----------------STDNLWRIIGNIS------------NLQERAVR
34557932     Wolinella succinogenes DSM 1740                -----------------------------------------------EAVFASATHSPNKKLELKDELKSEY----------------------GENAKELLAGLRVTKEILDEFDKQENQGNLPRAKYFEELGEYIATNEKVKSFFDSNSL---------------KLTDMTKLIGNIS------------NYQLKELR
54296138     Legionella pneumophila str. Paris              -----------------------------------------------SSEF--------RKI-LVSKVEE------------------------KKDDKELKNAVKNIKNFITGFEKNSVEGHRHRKVYFENIKSDITKDNQLDSIKKKI-----------------PSVCLSNLLGHLS------------NLQWKNLH
319941583    Sutterella wadsworthensis 3 1 45B              -----------------------------------------------DKEF--------IEFAVAEGLID------------------------KTEKKAYQSALSTLRANANVLTGLRQMGHKPRSEYFKAIEADLKKDSRLAKINEAFG----------------GAERLARLLGNLS------------NLQLRAER
254447899    gamma proteobact. HTCC5015                     ----------------------------------------------NKNEF--------KKL-LDTALDGKY----------------------KDEKKELSEAWGNILIASENVLKSTVDGHKSRSEYLANIKEDIKSNEELEKQISSK-----------------EIDGFYNLVGHLS------------NFQLRLLR
118497352    Francisella novicida U112                      EITSYEFELLADYLANYSESLKTQKFSYTDKQGNLKELSYYHHDKYNIQEFLKRHATINDRI-LDTLLTDDLDIWNFNFEKFDFDKNEEKL-QNQEDKDHIQAHLHHFVFAVNKIKSEMASGGRHRSQYFQEITNVLDENNHQEGYLKNFCENLHN-----KKYSNLSVKNLVNLIGNLS------------NLELKPLR

Motifs                                                      ________________________________________________________________________________________________________________________________________________________________________________________________________
informative positions                                       --------------------------------------------------------------------------------------------------------------------------------------------------------------------------------------------------------
```

```
227494853   Actinomyces coleocanis DSM 15436#            LRPKNREKTDTRPENKKPGF-----------------------------------------------------------------------------------------------------------------------------------------LGGKLMQSDNANELRKIAKIQGL---------DDALLRE
328956315   Coriobacterium glomerans PW2                 YRRYGEDLALLKKLVKIYAPDQYRMFFSGATYPGTGIYDAAQARGYTKYNL-------------------------------------------------------GPKKSEYKPSESMQYDDFRKAVEKLFA--KTDARADERYRMMMDRFDKQQFLRRLKTSDNGSIYHQLHEELKAIVENQGRFYPFL----KRDADKL
227824983   Acidaminococcus sp. D21                      YEQHHHDLTQLKYFVKTYLAKEYDDIFRNVDSETTKNYVAYSYHVKEVKGTLP-----------------------------------------------------KNKATQEEFCKYVLGKVKNI---------ECSEADKVDFDEMIQRLTDNSFMPKQVSGENRVIPYQLYYYELKTILNKAASYLPFL----TQCGKDA
303229466   Veillonella atypica ACS-134-V-Col7a          FDKHKEDLVILKSLLKLDRNVYNEMFKSDKKGLHNYVHYIKQGRTE------------------------------------------------------------ETSCSREDFYKYTKKIVEGLAD-------SKDKEY------ILNEIELQTLLPLQRIKDNGVIPYQLHLEELKVILDKCGPKFPFL-----HTVSDG
34762592    Fusobacterium nucleatum ATCC 49256         YEEHKKDLKNLKYIIRKYNKENYDKLFKDKNENNYPAYIGLNKEKD------------------------------------------------------------KKEVVEKSRLKIDDLIKVIKGYLPKPE--RIEEKDKTIFNEILNKIELKTILPKQRISDNGTLPYQIHEVELEKILENQSKYYDFL--NYEENGVST
374307738   Filifactor alocis ATCC 35896                YEKHKTNLRLLRDIILKYCTKDEYNRMFNDEKEAGSYTAYVGKLKK------------------------------------------------------------NNKKYWIEKKRNPEEFYKSLGKLLDKI--EPLKEDLEVLTMMIEECKNHTLLPIQKNKDNGVIPHQVHEVELKKILENAKKYYSFL----TETDKDG
320528778   Solobacterium moorei F0204                   FEKNKKDLMILKKYIKKNHPEDYKKIFSSPTEKKNYAAYSQTNS--------------------------------------------------------------KNVCSQEEFCLFIKPYIR-----------DMVKSENEDEVRIAKEVEDKSFLTKLKGTNNSVVPYQIHERELNQILKNIVAYLPFM----NDEQEDI
291520705   Coprococcus catus GD-7                       YQKHQADLKTLKKIVRQYMTKEDYKRVFVDTEEKLNNYSAYIGMTKKNGKKVDLK---------------------------------------------------SKQCTQADFYDFLKKNV-----------IKVIDHKEITQEIESEIEKENFLPKQVTKDNGVIPYQVHDYELKKILDNLGTRMPFI-----KENAEK
42525843    Treponema denticola ATCC 35405               YEKHKTDLTKLKNVIKKHFPKDYKKVFGYNKNEKNNNNYSGYVG--------------------------------------------------------------VCKTKSKKLIINNSVNQEDFYKFLKTI--LSAKSEIKEVNDILTEIETGTFLPKQISKSNAEIPYQLRKMELEKILSNAEKHFSFL----KQKDEKG
304438954   Peptoniphilus duerdenii ATCC BAA-1640        FNKHGKDLKTLKRIIKKYLPSEYANIFRNKSINDNYVAYTKSNITSNKRTK-------------------------------------------------------ASKFTKQEDFYKFIKKHLDTIKETKLN--SSENEDLKLIDEMLTDIEFKTFIPKLKSSDNGVIPYQLKLMELKKILDNQSKYYDFL--NESDEYGTV
224543312   Catenibacterium mitsuokai DSM 15897          YNKHHDDLKLLKDCIKNNVPNKYFDMFRNDSEKSKGYYNYINR---------------------------------------------------------------PSKAPVDEFYKYVKK-------------CIEKVDTPEAKQILNDIELENFLLKQNSRTNGSVPYQMQLDEMIKIIDNQAEYYPIL-----KEKREQ
116628213   Streptococcus thermophilus LMD-9            YNEHKEDLALLKEYIRNISLKTYNEVFKDDTKNGYAGYIDGKTNQEDFYVYL--------------------------------------------------------------------------------KKLLAEFEGADYFLEKIDREDFLRKQRTFDNGSIPYQIHLQEMRAILDKQAKFYPFL-----AKNKER
24379809    Streptococcus mutans UA159                   YNEHQMDLAQLKQFIRQKLSDKYNEVFSDVSKDGYAGYIDGKTNQEAFYKYL--------------------------------------------------------------------------------KGLLNKIEGSGYFLDKIEREDFLRKQRTFDNGSIPHQIHLQEMRAIIRRQAEFYPFL-----ADNQDR
13622193    Streptococcus pyogenes SF370                 YDEHHQDLTLLKALVRQQLPEKYKEIFFDQSKNGYAGYIDGGASQEEFYKFI--------------------------------------------------------------------------------KPILEKMDGTEELLVKLNREDLLRKQRTFDNGSIPHQIHLGELHAILRRQEDFYPFL-----KDNREK
310286728   Bifidobacterium bifidum S17                  YDAHRANWNLIKLQLRTKENEKDINENYGILVGWKIDS--------------------------------------------------------------------GQRKSVRGESAYENMRKKANVFFKKMI--ETSDLSETDKNRLIHDIEEDKLFPIQRDSDNGVIPHQLHQNELKQIIKKQGKYYPFL----LDAFEKD
366983953   Oenococcus kitaharae DSM 17330               FNEHQKDWEYIKSHIRNAKN----AGNGLYEKSKKFDG--------------------------------------------------------------------INAAYLALQSDNEDDRKKAKKIFQDEI--SSADIPDDVKADFLKKIDDDQFLPIQRTKNNGTIPHQLHRNELEQIIEKQGIYYPFL----KDTYQEN
422884106   Streptococcus sanguinis SK49                 YEEHRKDLQRFK-FFIKQNLSEQDYLDIF---GRKTQNGFDVDKETKGY---------------------------------------------------------VGYITNKMVLTNPQKQKTIQQNFYDYI--SGKITGIEGAEYFLNKISDGTFLRKLRTSDNGAIPNQIHAYELEKIIERQGKDYPFL----LENK---
339625081   Fructobacillus fructosus KCTC 3544           YDQHREDRKLLKSLAQKAGLKHKRFSEIY-----------------------------------------------------------------------------EQLALATDEATIKNGISTARELV--EESNLSKEVKEDTLRRLDENEFLPKQRTKANSVIPHQLHLAELQKILQNQGQYYPFL----LDTFEKE
306821691   Eubacterium yurii ATCC 43715                 YENHHKDLLKIKELLKKYD--KKAYNDMFRKMTDKNYSAYVGSVNSNIAKE-------------------------------------------------------RRSVDKRKI---EDLYKYIEDTALKNIPDD-----NKDKIEILEKIKLGEFLKKQLTASNGVIPNQLQSRELRAILKKAENYLPFL----KEKGEKN
336394882   Lactobacillus farciminis KCTC 3681           YENHAIDLCKLRDMWHTTKNEEAVEQ------SRQAYDDYINKPKYGT----------------------------------------------------------------------KELYTSLKKFLKVALPTN-------LAKEAEEKISKGTYLVKPRNSENGVVPYQLNKIEMEKIIDNQSQYYPFL----KENK---
323463801   Staphylococcus pseudintermedius ED99         YDKFRNELKQVKDIVYKADSTRTQFKKIFVS-SKKSLKQYDATPNDQTFSS-------------------------------------------------------LCLFDQYLIRP-KKQYSLLIKELKKIIPQD---------SELYFEAENDTLLKVLNTTDNASIPMQINLYEAETILRNQQKYHAEI----TDEM---
389815359   Planococcus antarcticus DSM 14505            FKQYQKDMASLKNLLDKT-FGEKVYRSYFIS-DKNSQREYQKSHKVEVLCK-------------------------------------------------------LDQFNKEAKFA-ETFYKDLKKLLEDKSKTSIGTTEKDEMLRIIKAIDSNQFLQKQKGIQNAAIPHQNSLYEAEKILRNQQAHYPFI----TTEW---
258509199   Lactobacillus rhamnosus GG                   YGQHKEDLELFKTYLKKLNDHELAKTIRGLYDRYINGDDAKPFLREDFVKALTK------------------------------------------------------------------------------EVT--AHPNEVSEQLLNRMGQANFMLKQRTKANGAIPIQLQQRELDQIIANQSKYYDWLA--APNPVEAH
169823755   Finegoldia magna ATCC 29328                  YDEHKKDLEILKKVIKKYDEDGKLFKQVFKEDNGNGYVSYIGYYLNKNKKITA-----------------------------------------------------KKKISNIEFTKYVKGILE---------KQC--DCEDEDVKYLLGKIEQENFLLKQISSINSVIPHQIHLFELDKILENLAKNYPSFN--NKKEEFTK
Jnet        227501312                                    HHHHHHHHHHHHHHHH----HHH-HHHHHHHHHH------------------------------------------------------------------------------HHHHHHHHHHHH-------------------HHHHHHHHHHHH------E--------HHHHHHHHHHHHHHHHH-----------------
47458868    Mycoplasma mobile 163K                       YKISGKIRST-----------------------------------------------------------------------------------------------------------------------------------------------INLKFGHQDYLKEIKQVFEKQNIDFMNY-----EKFAME
284931710   Mycoplasma gallisepticum str. F              YKGIIDSKEDNDNKLEEEL--------------------------------------------------------------------------------------------------------------------------------------TKYKFSNKHWLEEVKKVLSNQTGL--------PEKFKEE
71894592    Mycoplasma synoviae 53                       LDNKKDKAKEKDEEEESDAKKES----------------------------------------------------------------------------------------------------------------------------------EELIFSNKQWINEIVKVFENQSYL--------TESFKEE
363542550   Mycoplasma ovipneumoniae SC01                -------------------PKDL----------------------------------------------------------------------------------------------------------------------------------GGYSFSNLQWVNEIKKLFEVQEIN---------PEFSEK
384393286   Mycoplasma canis PG 14                       --------PTESEMKDNKDLKEA----------------------------------------------------------------------------------------------------------------------------------FFFDFSNKEWLKEINYFFNVQKNILS-------ETFIEE
238924075   Eubacterium rectale ATCC 33656               YRGQSQIINENGEVLD-----------------------------------------------------------------------------------------------------------------------------------------LSNVFTIGAYRKEIQRVFEIQKKYHPEL----TDEFCDG
315149830   Enterococcus faecalis TX0012                 LKENNRVKTGINAQGNY----------------------------------------------------------------------------------------------------------------------------------------QLNVFKVSAYANELATILKTQQAFYPNEL---TDDWIAL
116627542   Streptococcus thermophilus LMD-9            YQTYGQLRGDFTV-EKDGKKHR-----------------------------------------------------------------------------------------------------------------------------------LINVFPTSAYRSEALRILQTQQEFNPQI----TDEFINR
315659848   Staphylococcus lugdunensis M23590           MNEGQVRG-------------------------------------------------------------------------------------------------------------------------------------------------EKNRFKTADIIKEIIQLLNVQKNFHQL-----DENFINK
160915782   Eubacterium dolichum DSM 3991                LRTNGHIRG------------------------------------------------------------------------------------------------------------------------------------------------HENNFKTRAYVDEAFQILSHQDL---------SNELKSA
325677756   Ruminococcus albus 8                         FDEPNSVYRKFHNSAASK---------------------------------------------------------------------------------------------------------------------------------------NHYLITRHMLVKEVDLILENQSKYYGIL----DDKTIAK
225377804   Roseburia inulinivorans DSM 16841            HTECLWNEKGY--------------------------------------------------------------------------------------------------------------------------------VLTPRNRPDDYKHTILRSMLVEEVHAIFAAQRAHGNQKA---TEGLEEA
336393381   Lactobacillus coryniformis KCTC 3535         YNDHKRNKGYIDG--------------------------------------------------------------------------------------------------------------------------------------------YLSTPNRQMLVDEIKQILDKQRELGNEKL---TDEFYAT
310780384   Ilyobacter polytropus DSM 2926               FNTHVRNKAGS----------------------------------------------------------------------------------------------------------------------------------------------YLFSFSRKLLEDEVRKIQAKQKELGNTHF---TDDVLEK
301311869   Bacteroides sp. 20 3                         LEADGSPKKDKEGNIKRSFRAPKDDDWTLIKKKTEADIDKI-----------------------------------------------------------------------------------KMTVGAYIYDTLLQKPDQ-KIRGK--LVRTIERKYYKNELYQILKTQSEFHEELR---DKQLYIA
385811609   Ignavibacterium album JCM 16511              LE-KGKFKQNKPD-------LPKEEDWALCTTALDNRMG---------------------------------------------------------------------------------------SKHPGEFFFDELLKAFKE-K-RGYKIRQYPVNRWRYKKELEFIWTKQCQLNPELN---NLNINKE
60683389    Bacteroides fragilis NCTC 9343              YTEWNKEKGKTEQQEREHKLEGIYSKRKRDEAKRENLQWRVNGLKEKL----------------------------------------------------------SLEQLVIVFQEMNTQINNSSGYLGAISDRS--KELYFNKQTVGQYQMEMLDKNPNASLRNMVFYRQDYLDEFNMLWEKQAVYHKEL----TEELKKE
319957206   Nitratifractor salsuginis DSM 16511          YRNHDD---------------------------------------------------------------------------------------------------------------------------------------------------YEKMIRREDIEEEIEKVLLRQAELGALGL--PEEQVSEL
187250660   Elusimicrobium minutum Pei191                FFENKEKHKREKISNAANN--------------------------------------------------------------------------------------------------------------------------------------HKFSPTRDLVIKEAEAILKKQAGKDGFHKE-LTEEYIEK
325972003   Sphaerochaeta globus str. Buddy              FNTNGLIGYRNSESVKSK---------------------------------------------------------------------------------------------------------------------------------------GVPVPTRDIISNEIDVLLQTQKQFYQEIL---SDEYCDR
296446027   Methylosinus trichosporium OB3b              QTPTKNGASEIVRRFRNREGD------------------------------------------------------------------------------------------------------------------------------------YSRSLLRDDLAAEMRALFTAQARFQSAIA---TADLQTA
347536497   Flavobacterium branchiophilum FL-15          KDVLGKVKGKIKDISQA---------------------------------------------------------------------------------------MLDGNYKTLGQYFYSLY----SKEKIR--NQYTSREEHYLSEFITICKVQGIDQINE----EEKINEK
345885718   Prevotella sp. C561                          PDVAGAMKASEEKLSKGLSTY-----------------------------------------------------------------------------------MKEHNLLTVGAAFAQLED---EGVRVRNNNDYRAIRSQFQHEIETIFKFQ--QGLSV----ESELYER
282880052   Prevotella timonensis CRIS 5C-B1             ED--GVVKSGISQLSTE---------------------------------------------------------------------------------------MEEAGCEYLGDYFYKLYDAQGNKVRIR--QRYTDRNKHYQHEFDAICEKQ----------------E
312879015   Aminomonas paucivorans DSM 12260             FQDHRRNRAGD----------------------------------------------------------------------------------------------------------------------------------------------YSHTLSRSLLLEEARRLFQSQRTLGNPHA---SSNLEEA
294086111   Candidatus Puniceispirillum marinum IMCC1322 QATNDTVRARRLSGTNAL---------------------------------------------------------------------------------------------------------------------------------------YEFYPDRYMLEQEFDTLWAKQAAFNPSLY---IEAARER
330822845   Alicycliphilus denitrificans K601            LQ------QGQGVRARYREKPYTTEEGKKRIDKS-----------------------------------------------------------------------------------------------------------------------YDLYIDRAMIEQEFDALWAAQAAFNPTLF---HEAARAD
344171927   Ralstonia syzygii R24                        LEDGRPVRARMVGQGKDEH--------------------------------------------------------------------------------------------------------------------------------------YELYIAREWIAQEFDALWASQQRFHAEVL---ADAARDR
159042956   Dinoroseobacter shibae DFL 12                RQKATDPRHVPSVRTRLSIANRGGPDGKEEAG---------------------------------------------------------------------------------------------------------------------------YDFYPDRRHLEEEFHKLWAAQGAHHPEL----TETLRDL
83591793    Rhodospirillum rubrum ATCC 11170             LDRGQPVRMRYNQSDRD----------------------------------------------------------------------------------------------------------------------------------------GVVAPTRALIAEELAEIVARQSSAYPGL------DWPAV
288957741   Azospirillum sp. B510                        KTRGETLRARLAGKGKEAA--------------------------------------------------------------------------------------------------------------------------------------YPFYPARRMLEAEFDTLWAEQARHHPDLL---TAEAREI
427429481   Caenispirillum salinarum AK4                 RLMAEHMHRHGTQPTCGEIMAGRLRETPAGAQPVTRARDGLRVGG------------------------------------------------------------------------------------------------------------GVAVPTRALIEQEFDAIRAIQAPRHPDL-------PWDS
92109262    Nitrobacter hamburgensis X14                 YEDRQTAIRAELVRLGKDHLTGNARKKIWAKVRK-------------------------------------------------------------------------------------RLF--GDEVLPRADAPHGVRARATITGTKASYDYYPTRDMLRDEFNAIWAGQSAHHATI----TDEARTE
148255343   Bradyrhizobium sp. BTAi1                     LRATVRARSPVGGDVQAR---------------------------------------------------------------------------------------------------------------------------------------YEFYPTRAMVDAEFEAIWAAQAPHHPTM----TAEAHDT
34557790    Wolinella succinogenes DSM 1740              RGANGKKRNKHGD--------------------------------------------------------------------------------------------------------------------------------------------YEISIHRDLLVEEVEAIFVAQQEMRSTIA---TDALKAA
218563121   Campylobacter jejuni NCTC 11168              FKENSKEFTNVRNKKES----------------------------------------------------------------------------------------------------------------------------------------YERCIAQSFLKDELKLIFKKQREFGFSF----SKKFEEE
Jnet        218563121                                    -----------------------------------------------------------------------------------------------------------------------------------------------------------------HHHHHHHHHHHH-------------HHHHHHH
291276265   Helicobacter mustelae 12198                  YFQKSLNVRNKKES-------------------------------------------------------------------------------------------------------------------------------------------YNRCVGRSELREELKTIFQIQQELKSPWV---NEELIYK
222109285   Acidovorax ebreus TPSY                       FPDAQRNKRGQ----------------------------------------------------------------------------------------------------------------------------------------------YDKALSRVLLGEELALLFATQRRLGNPH----ASDFFEK
365156657   Bacillus smithii 7 3 47FAA                  FAYHKRNKLDS----------------------------------------------------------------------------------------------------------------------------------------------YSNMIARDDLEREIKLIFEKQREFNNPVC---TERLEEK
220930482   Clostridium cellulolyticum H10               TPENSNKRNKVDE--------------------------------------------------------------------------------------------------------------------------------------------YIHTIAREDLLNEIKYIFSIQRKLGSPFV---TEKLEHD
297182908   uncultured delta proteobact. HF0070 07E19    AQNRDRQRNREGD--------------------------------------------------------------------------------------------------------------------------------------------YSNLMARKLVFEEALQILAFQRKQGHE-L---SKDFEKT
154250555   Parvibaculum lavamentivorans DS-1            PPSDRK---------------------------------------------------------------------------------------------------------------------------------------------------RGIHAHRNVVAEEFERLWEVQSKFHPALK---SEEMRAR
218767588   Neisseria meningitidis Z2491                 ESGHIRNQRGD----------------------------------------------------------------------------------------------------------------------------------------------YSHTFSRKDLQAELILLFEKQKEFGNPHV---SGGLKEG
15602992    Pasteurella multocida str. Pm70              EEGHIRNQRGA----------------------------------------------------------------------------------------------------------------------------------------------YTHTFNRLDLLAELNLLFAQQHQFGNPHC---KEHIQQY
187736489   Akkermansia muciniphila ATCC BAA-835         LEEGKADAPMEVSTPAYKN--------------------------------------------------------------------------------------------------------------------------------------LNTAFPRLIVEKEVRRILELSAPLIPGL----TAEIIEL
315605738   Actinomyces sp. oral taxon 180 str. F0310    IRGFVRKGGKA---TKVHGV-------------------------------------------------------------------------------------------------------------------------------------LEGKYMQSDLVAELRQICRTQRV---------SETTFEK
117929158   Acidothermus cellulolyticus 11B              LNPTQQSAGRRAELSNATA--------------------------------------------------------------------------------------------------------------------------------------FETRLRQEDVLWELRCIADVQGL---------PEDVVSN
189440764   Bifidobacterium longum DJO10A                LDAGYAEAPRLRWRTGSKKPDAEGY--------------------------------------------------------------------------------------------------------------------------------LPVRLMQEDNANELKQIFRVQRVP--------ADEWKPL
403744858   Alicyclobacillus hesperidum URH17-3-68       TDAEGKLVHGKKHGYVSNVRNKAGEY--------------------------------------------------------------------------------------------------------------------------------RHTVSRQAIVDEVRKIFAAQRAL---GNDVMSEELEDS
407803669   Alcanivorax sp. W11-5                        YAPEYALFRQYLGL--PAIKGRPDCLP-------------------------------------------------------------------------------------------------------------------------------NMYALRHQIEHEFERIWATQSQF---HDVMKDHGVKEE
423317190   Bergeyella zoohelcum ATCC 43767              LKEKSKEMGREFYISEFLLELLKENRWAKIR---------------------------------------------------------------------------------------------------------------------------NNTILRARYESEFEAIWNEQVKH---YPFLENLDKKTL
402847315   Porphyromonas sp. oral taxon 279 str. F0450  ----AEQLRQNQSES-----PTGGISYR-IK---------------------------------------------------------------------------------------------------------------------------DQIFSRQCYIDEYDQIMAVQRVH---YPDILTDEFIRM
404487228   Barnesiella intestinihominis YIT 11860       ----FRRLTENAFF-----RCKEQVY------------------------------------------------------------------------------------------------------------------------------------PRQAYVEEFDCIMNCQRKF---YPDILTDETIRC
374384763   Odoribacter laneus YIT 12061                 ----YDIAPKNGEK----YRFRTERVR-------------------------------------------------------------------------------------------------------------------------------ARYTLRDMYIREFEIIWQRQAGHLGLAHEQATRKKNIF
384109266   Treponema sp. JC4                            ----YKNQGENGGI-----RFAPGRM--------------------------------------------------------------------------------------------------------------------------------TYYPTRKMYEEEFNLIRSKQEKY---YPQVDWDDIYKA
402849997   Rhodovulum sp. PH10                          ---WSRHRGTPRTRSPTRIRMEGEGAKA-LY--------------------------------------------------------------------------------------------------------------------------AFYPTREMVRAEFERLWTAQSRF---APDLLTPERHEE
Jnet        331001027                                    HH-------------HHHHHHHHHHHHHHH---------HHHHHHHHHHHHH---------------------------------------------------------------------------------HHHHHH------------------------EEE--HHHH---------
331001027   Parasutterella excrementihominis YIT 11859   WYFNDAKFEQGQEQLDAVKLKNVLVRALKY-LRSDDKEWSASQKQIIQSLEQ----------------------------------------------------------------------------SGDVLDVLAGLDPDRTIPPYEDQNNRRPPEDQTLYLNPKALSSEYG----
34557932    Wolinella succinogenes DSM 1740              RYFNDKEMEKG-DIWIPNKLHKITERFVRS-WHPKNDADRQRRAELMKDLKS----------------------------------------------------------------------------KE-IMELLTTTEPVMTIPPYDDMNNRGAVKCQTLRLNEEYLDKHL-----
54296138    Legionella pneumophila str. Paris            RYLAKNP-----KQFDEQTFGNEFLRMLKNFRHLKGSQESLAVRNLIQQLEQ----------------------------------------------------------------------------SQDYISILEKTPPEITIPPYEARTNTGMEKDQSLLLNPEKLNNLY-----
319941583   Sutterella wadsworthensis 3 1 45B            WYFNAPDIMKD-RGWEPDRFKKTLVRAFKF-FHP-AKDQNKQHLELIKQIEN----------------------------------------------------------------------------SEDIIETLCTLDPNRTIPPYEDQNNRRPPLDQTLLLSPEKLTRQYG----
254447899   gamma proteobact. HTCC5015                   KYFNDPNMSGV-SYWDEKRLEKYFYQWVQG-WHTKGGTDEAEKKNIILKTKG----------------------------------------------------------------------------AP-LLKTLKSLSADLTIPPYEDQNNRRPPKCQSVLLSDEKLTMHY-----
118497352   Francisella novicida U112                    KYFNDKIHAKA-DHWDEQKFTETYCHWILG-EWRVGVKDQDKKDGAKYSYKDLCNELKQK------------------------------------------------------------------VTKAGLVDFLLELDPCRTIPPYLDNNNRKPPKCQSLILNPKFLDNQY-----

Motifs                                                   ___________________________________________________________________________________________________________________________________________________________________________________________________
informative positions                                    ---------------------------------------------------------------------------------------------------------------------------------------------------------------------------------------------------
```

227494853	*Actinomyces coleocanis* DSM 15436#	L---IELVFAADSPKGA--SGELVGYDV------LPGQHGK-----------------------RR------AEKAHP
328956315	*Coriobacterium glomerans* PW2	V--SLVSFRIPYYVGPL---------STRNARTDQHGEN-RFAWSERKPGMQDEPIFPWNWESIIDRSKSAEKFILRMTGMCT------YLQQE-P-----------------------VL------P-KSSL
227824983	*Acidaminococcus* sp. D21	I---SNQDKLLSIMTFRIPYFVGPL---------RKDNSEHAWL------------ERKAGKIYPWNFNDKVDLDKSEEAFIRRMTNTCT------YYPGE-D-----------------------VL------P-LDSL
303229466	*Veillonella atypica* ACS-134-V-Col7a	F---SVTEKLIKMLEFRIPYYVGPL---------NTHHNIDNGG--------FSWAVRKQAGRVTPWNFEEKIDREKSAAAFIKNLTNKCT------YLFGE-D-----------------------VL------P-KSSL
34762592	*Fusobacterium nucleatum* ATCC 49256	K--DKLLKTFKFRIPYYVGPL---------NSYHKDKGGN--------SWIVRKEEGKILPWNFEQKVDIEKSAEEFIKRMTNKCT------YLNGE-D-----------------------VI------P-KDSF
374307738	*Filifactor alocis* ATCC 35896	Y---SVVQKIESIFRFRIPYYVGPL---------STRHQEKGSN------VWMVRKPGREDRIYPWNMEEIIDFEKSNENFITRMTNKCT------YLIGE-D-----------------------VL------P-KHSL
320528778	*Solobacterium moorei* F0204	S--VVDKIKLIFKFKIPYYVGPL---------NTKSTRSWVY-------------RSDEKIYPWNFSNVIDLDKTAHEFMNRLIGRCT------YTNDP-------------------------VL------P-MDSL
291520705	*Coprococcus catus* GD-7	I---QQLFEFRIPYYVGPL---------NRVDDGKDGK-------FTWSVRKSDARIYPWNFTEVIDVEASAEKFIRRMTNKCT------YLVGE-D-----------------------VL------P-KDSL
42525843	*Treponema denticola* ATCC 35405	L---SHSEKIIMLLTFKIPYYIGPI---------NDNHKKFFPD-----RCWVVKKEKSPSGKTTPWNFFDHIDKEKTAEAFITSRTNFCT------YLVGE-S-----------------------VL------P-KSSL
304438954	*Peptoniphilus duerdenii* ATCC BAA-1640	K--DKVESIMEFRIPYYVGPL---------NPDSKYAWIK-------------RENTKITPWNFKDIVDLDSSREEFIDRLIGRCT------YLKEE-K-----------------------VL------P-KASL
224543312	*Catenibacterium mitsuokai* DSM 15897	L---LSILTFRIPYYFGPL---------NETSEHAWIK---------RLEGKENQRILPWNYQDIVDVDATAEGFIKRMRSYCT------YFPDE-E-----------------------VL------P-KNSL
116628213	*Streptococcus thermophilus* LMD-9	I---EKILTFRIPYYVGPL-------ARGNSDFAWSI-------------RKRNEKITPWNFEDVIDKESSAEAFINRMTSFDL------YLPEE-K-----------------------VL------P-KHSL
24379809	*Streptococcus mutans* UA159	I---EKLLTFRIPYYVGPL-------ARGKSDFAWLS-------------RKSADKITPWNFDEIVDKESSAEAFINRMTNYDL------YLPNQ-K-----------------------VL------P-KHSL
13622193	*Streptococcus pyogenes* SF370	I---EKILTFRIPYYVGPL-------ARGNSRFAWMT-------------RKSEETITPWNFEEVVDKGASAQSFIERMTNFDK------NLPNE-K-----------------------VL------P-KHSL
310286728	*Bifidobacterium bifidum* S17	G---KQINKIEGLLTFRVPYFVGPL-VVPEDLQKSDNSENHWMV------------RKKKGEITPWNFDEMVDKDASGRKFIERLVGTDS------YLLGE-P-----------------------TL------P-KNSL
366983953	*Oenococcus kitaharae* DSM 17330	SHE---LNKITALINFRVPYYVGPL-VEEEQKIADDGKNIPDPTNHWMV------RKSNDTITPWNLSQVVDLDKSGRRFIERLTGTDT------YLIGE-P-----------------------TL------P-KNSL
422884106	*Streptococcus sanguinis* SK49	---DKLLSILTFKIPYYVGPL------AKGSNSRFAWIKRATSSDILDDNDEDTRNGKIRPWNYQKLINMDETRDAFITNLIGNDI------ILLNE-K-----------------------VL------P-KRSL
339625081	*Fructobacillus fructosus* KCTC 3544	D---GQDNKIEELLRFRIPYYVGPLVTKKDVEHAGGDADNHWVER---------NEGFEKSRVTPWNFDKVFNRDKAARDFIERLTGNDT------YLIGE-K-----------------------TL------P-QNSL
306821691	*Eubacterium yurii* ATCC 43715	L---TVSEMIIQLFEFQIPYYVGPL----DKNPKKDKNKANSWAK-----------IKQGGRILPWNFEDKVDVKGSRKEFIEKMVRKCT------YISDE-H-----------------------TL------P-KQSL
336394882	*Lactobacillus farciminis* KCTC 3681	---EKLLSILSFRIPYYVGPL------QSAEKNPFAWME-------------RKSNGHARPWNFDEIVDREKSSNKFIRRMTVTDS------YLVGE-P-----------------------VL------P-KNSL
323463801	*Staphylococcus pseudintermedius* ED99	--IEKVLSLIQFRIPYYVGPL-----VNDHTASKFGWME-------------RKSNESIKPWNFDEVVDRSKSATQFIRRMTNKCS------YLINE-D-----------------------VL------P-KNSL
389815359	*Planococcus antarcticus* DSM 14505	--IEKVKQILAFRIPYYIGPL-----VKDTTQSPFSWVE-------------RKGDAPITPWNFDEQIDKAASAEAFISRMRKTCT------YLKGQ-E-----------------------VL------P-KSSL
258509199	*Lactobacillus rhamnosus* GG	R--WKMPYQLDELLNFHIPYYVGPLITPKQQAESG---ENVFAWMVRKDPSG---------NITPYNFDEKVDREASANTFIQRMKTTDT------YLIGE-D-----------------------VL------P-KQSL
169823755	*Finegoldia magna* ATCC 29328	I--EKIRKTFTFRIPYYVGPLNDYHKNNGG--------NAWIFRNKGE---------KIRPWNFEKIVDLHKSEEEFIKRMLNQCT------YLPEE-T-----------------------VL------P-KSSI
Jnet	227501312	H--HHHHHHHH-----EE-----------------------------------EEEEEE-----------------HHHHHHHHHHHH--
47458868	*Mycoplasma mobile* 163K	E---KSFFSRIRNYSEGPGNEKSFSKYGL---------YANENGN--------PELIINEKGQKIYTKIFKTLWESKIGKCS------YDKKL-Y-----------------------RA------P-KNSF
284931710	*Mycoplasma gallisepticum* str. F	Y---ESLFSYVRNYSEGPGSINSVSPYGI---------------------------YHLDEKEGKVVQKYNNIWDKTIGKCN------IFPDE-Y-----------------------RA------P-KNSP
71894592	*Mycoplasma synoviae* 53	Y---LKLFNYVRPFNKGPGSKNSRTAYGV--------------------------FSTDIDPETNKFKDYSNIWDKTIGKCS------LFEEE-I-----------------------RA------P-KNLP
363542550	*Mycoplasma ovipneumoniae* SC01	F---LNLFTSVRDYAKGPGSEHSASEYGI--------------------------FQK--DEKGKVFKKYDNIWDKTIGKCS------FFVEE-N-----------------------RS------P-VNYP
384393286	*Mycoplasma canis* PG 14	F---KKIFSFTRDISKGPGSDNMPSPYGI--------------------------FGEF-GDNGQG-GRYEHIWDKNIGKCS------IFTNE-Q-----------------------RA------P-KYLP
238924075	*Eubacterium rectale* ATCC 33656	Y---MLIFNRKRKYYEGPGNEKSRTDYGR---------------------------FTTKLDANGNYITEDNIFEKLIGKCS------VYPDE-L-----------------------RA------A-AASY
315149830	*Enterococcus faecalis* TX0012	F---VQPGIAEEAGLIYRKRPYYHGPGNEANNSPYGR-------------------------------WSDFQKTGEPATNIFDKLIGKD------FQGEL-------------------------RA------S-GLSL
116627542	*Streptococcus thermophilus* LMD-9	Y---LEILTGKRKYYHGPGNEKSRTDYGR---------------------------------YRTSGETLDNIFGILIGKCT------FYPDE-F-----------------------RA------A-KASY
315659848	*Staphylococcus lugdunensis* M23590	Y---IELVEMRREYFEGPGKGSPYGWEGD--PKAWYETLMGHCT------YFPDELR-----------------------SV-------KYAY
160915782	*Eubacterium dolichum* DSM 3991	I---ITIISRKRMYYDGPGGPLSPTPYGR--------------------------------YTYFGQKEPIDLIEKMRGKCS------LFPNE-P-----------------------RA------P-KLAY
325677756	*Ruminococcus albus* 8	I---KDIIFAQRDFEIGPGKNERFRRFTG--YLDSIGKCQ------FFKDQ-E-----------------------RG------S-RFTV
225377804	*Roseburia inulinivorans* DSM 16841	Y---VEIMTSQRSFDMGPGLQPDGKPS--PYAMEGFGDRVGKCT------FEKDE-Y-----------------------RA------P-KATY
336393381	*Lactobacillus coryniformis* KCTC 3535	Y--LLGDENRAGIFQAQRDFDEGPGAGPYAGDQ--IKKMVGKDI------FEPTE-D-----------------------RA------A-KATY
310780384	*Ilyobacter polytropus* DSM 2926	Y---IEVFNSQRNFDEGPSKPSPYYSEIGQ---IAKMIGNCT------FESSE-K-----------------------RT------A-KNTW
301311869	*Bacteroides* sp. 20 3	CL---NE----LYPNNE----------------------------PRRNSISTRDFCHLFIEDIIFYQRPLKS--KKSLIDNCP------YEENR-YI-------------DKESGEIKHA-SIKCIA-KSHP
385811609	*Ignavibacterium album* JCM 16511	IL---RKLATVLYPSQSKF------------------------FGPKIKEFENSDVLHIISEDIIYYQRDLKS--QKSLISECR------YEKRK-GI-------------D---GEI--Y-GLKCIP-KSSP
60683389	*Bacteroides fragilis* NCTC 9343	I---RDIIIFYQRRLKS--QKGLIGFCE------FESRQIEV-------------DIDGKKKIKTVGNRVIS-RSSP
319957206	*Nitratifractor salsuginis* DSM 16511	I--DELKACITDQEMPTID---ESLFGKCT------FYKDE-L-----------------------AA------P-AYSY
187250660	*Elusimicrobium minutum* Pei191	L--TKAIGYESEK--LIPESGFCP------YLKDE-K-----------------------RL------P-ASHK
325972003	*Sphaerochaeta globus* str. Buddy	I--VSAILFENEK--IVPEAGCCP------YFPDE-K-----------------------KL------P-RCHF
296446027	*Methylosinus trichosporium* OB3b	F--TKAAFFQRPLQD--SEKLVGPCP------FEVDE-K-----------------------RA------P-KRGY
347536497	*Flavobacterium branchiophilum* FL-15	K--FDGLAKDLYKAIFFQRPLKS--QKGLIGKCS------FEKSK-S-----------------------RC------A-ISHP
345885718	*Prevotella* sp. C561	L--ISE--KKNVGTIFYKRPLRS--QRGNVGKCT------LERSK-P-----------------------RC------A-IGHP
282880052	*Prevotella timonensis* CRIS 5C-B1	L--SSELIEDLQRAIFFQLPLKS--QRHGVGRCT------FERGK-P-----------------------RC------A-DSHP
312879015	*Aminomonas paucivorans* DSM 12260	F---LHLVAFQNPFASGED--IRNKAGHCS------LEPDQ-I-----------------------RA------P-RRSA
294086111	*Candidatus Puniceispirillum marinum* IMCC1322	L--KEIVFFQRKL--KPQEVGRCI------FLSDE-D-----------------------RI------S-KALP
330822845	*Alicycliphilus denitrificans* K601	L--KDTLLHQRPL--RPVKPGRCT------LLPEE-E-----------------------RA------P-LALP
344171927	*Ralstonia syzygii* R24	L--RAILLFQRKL--LPVPVGKCF------LEPNQ-P-----------------------RV------A-AALP
159042956	*Dinoroseobacter shibae* DFL 12	L--FEKIFFQRPL--KEPEVGLCLFSGHHGVPPKD-P-----------------------RL------P-KAHP
83591793	*Rhodospirillum rubrum* ATCC 11170	T---RLVLDQRPL--RSKGAGPCA------FLPGE-D-----------------------RA------L-RALP
288957741	*Azospirillum* sp. B510	L--RHRIFHQRPL--KPPPVGRCT------LYPDD-G-----------------------RA------P-RALP
427429481	*Caenispirillum salinarum* AK4	L--RRLVLDQAPI--AVPPATPCL------FLEELRR-------RGETFQGRTITREAIDRG------L-TVDP
92109262	*Nitrobacter hamburgensis* X14	I--EHIIFYQRPL--KPAIVGKCT------LDPAT-RP--------------FKEDPEGYRA------P-WSHP
148255343	*Bradyrhizobium* sp. BTAi1	I--REAIFSQRAM--KRPSIGKCS------LDPATSQD--------------DVDGF---RC------A-WSHP
34557790	*Wolinella succinogenes* DSM 1740	Y--REIAFFVRPMQR--IEKMVGHCT------YFPEE-R-----------------------RA------P-KSAP
218563121	*Campylobacter jejuni* NCTC 11168	V--LSVAFYKRALKD--FSHLVGNCS------FFTDE-K-----------------------RA------P-KNSP
Jnet	218563121	H--HHHHHHHH--H
291276265	*Helicobacter mustelae* 12198	LLGNP--DAQSKQEREGLIFYQRPLKG--FGDKIGKCS------HIKKGENS--------------PY------RA------C-KHAP
222109285	*Acidovorax ebreus* TPSY	L---ILGDGDRKSGLFWQQKPALSGAD--LLKMLGKCT------FEKGE-Y-----------------------RA------P-KASF
365156657	*Bacillus smithii* 7 3 47FAA	Y--LNIWSSQRPFASKED---IEKKVGFCT------FEPKE-K-----------------------RA------P-KATY
220930482	*Clostridium cellulolyticum* H10	F--LNIWEFQRPFASGDS---ILSKVGKCT------LLKEE-L-----------------------RA------P-TSCY
297182908	uncultured delta proteobact. HF0070 07E19	Y--LDVLMGQRSGR--SPKLGNCS------LIPSE-L-----------------------RA------P-SSAP
154250555	*Parvibaculum lavamentivorans* DS-1	I--SDTIFAQRPVFW--RKNTLGECR------FMPGE-P-----------------------LC------P-KGSW
218767588	*Neisseria meningitidis* Z2491	I--ETLLMTQRPALSGDA---VQKMLGHCT------FEPAE-P-----------------------KA------A-KNTY
15602992	*Pasteurella multocida* str. Pm70	M--TELLMWQKPALSGEA---ILKMLGKCT------HEKNE-F-----------------------KA------A-KHTY
187736489	*Akkermansia muciniphila* ATCC BAA-835	I--AQHHPLTTEQRGVLLQHGIKLARRYRGS------------------------LLFGQLIPRFDNRIISRCPVTWAQVYEAELKKG--------------NSEQSARERAEKLSKVPTANCP
315605738	*Actinomyces* sp. oral taxon 180 str. F0310	L--VLSIFHSKEPAPS---AARQRERVGLDELQ--LALDPAAKQ--------------------PR------AERAHP
117929158	*Acidothermus cellulolyticus* 11B	V--IDAVFCQKRPSV--PAERIGRDP------LDPSQ-L-----------------------RA------S-RACL
189440764	*Bifidobacterium longum* DJO10A	F--RSVFYAVSPKGS--AEQRVGQDP------LAPEQ-A-----------------------RA------L-KASL
403744858	*Alicyclobacillus hesperidum* URH17-3-68	Y---LKILCSQRNFDDGPGGDSPYGHGSVSPDGVR---QSIYERMVGSCT------FETGE------------------------------KRAP-RSSY
407803669	*Alcanivorax* sp. W11-5	I--RNAIFFQRPLKS--PADKVGRCS------LQTNL------------------------------PRAP-RAQI
423317190	*Bergeyella zoohelcum* ATCC 43767	-----------------------------IEIVSFIFPGEKESQKKYRELGLEKGLKYIIKNQVVFYQRELKD--QSHLISDCR------YEPNE------------------------------KAIA-KSHP
402847315	*Porphyromonas* sp. oral taxon 279 str. F0450	L--RDEVIFMQRPLKS---CKHLVSLCE------FEKQERVM------RVQQDDGKGGWQLVERRVKFGPKVAP-KSSP
404487228	*Barnesiella intestinihominis* YIT 11860	I--RDEIIYYQRPLKS---CKYLVSRCE------FEKRF-----------------YLNAAGKKTEAGPKVSP-RTSP
374384763	*Odoribacter laneus* YIT 12061	L-------EGSATNVRNSKLITHLQAKYGRGHVLIEDTRITVTFQLPLKEVLGGKIEIEEEQLKFKSNESVLFWQRPLRS---QKSLLSKCV------FEGRN----------------FYDPVHQKWIIAGPTPAP-LSHP
384109266	*Treponema* sp. JC4	I--FYQRPLK---PQQRGYCI------YENDK------------------------------ERTF-KAMP
402849997	*Rhodovulum* sp. PH10	I--AGILFRQRDLA---PPKIGCCT------FEPSE------------------------------RRLP-RALP
Jnet	331001027	---HHHHHHHHHH---HHH----HHHHHHHH-------E--------------------EEE--------HHHH
331001027	*Parasutterella excrementihominis* YIT 11859	---EKWKSWANKF---AGAYPLLTEDLTEILKNTDRKSR------------------IKIRSDVLPDSDYRLAY
34557932	*Wolinella succinogenes* DSM 1740	---PNWRDIAKRL-----NHGKFNDDLADSTVKGYS----------------------------------EDST
54296138	*Legionella pneumophila* str. Paris	---PNWRNLIPGI---IDAHPFLEKDLEHTKLRDRKR-----------------------IISPSKQDEKRDSY
319941583	*Sutterella wadsworthensis* 3 1 45B	---EIWKTWSARL---TSAEPTLAPAAEILERSTDRKSR-----------------VAVNGHEPLPTLAYQLSY
254447899	gamma proteobact. HTCC5015	---PKWKEWVGQLVK-QNDNAYLNENVT---LAN
118497352	*Francisella novicida* U112	---PNWQQYLQELKKLQSIQNYLDSFETDLKVLKSSKDQPYF--V--------------EYKSSNQQIASGQRDYKDLDAR
Motifs		____
informative positions		---***

227494853	*Actinomyces coleocanis* DSM 15436#	AF-QRYRIAS-IVSNLRI-------------------------RHLGSGADERLDVETQKRVFEYLLN----AKPTAD------IT---WSD----VA-------EEIGVER---NLLMGTATQT--------------ADGERASAKPPVDVT----------NVAFATCKIKPLKEWWLNADYEARCVMVSALSH
328956315	*Coriobacterium glomerans* PW2	LY-EEFCVLN-ELNGAHW-------------------------SIDGDDEHRFDAADREGIIEELFR-----R-KRT------VS---YGD----VA-------GWMERERNQIGAHVC------------------GGQGEKGFESKLGSY-IF--FCK--DVFKVERLEQS---------------DYPMIER
227824983	*Acidaminococcus* sp. D21	IY-EKFMILN-EINNIRI---------------------------------DGYPISVDVKQQVFGLFEK-----K--RR------VT---VKD----IQ-------NLLLSLG---ALDKHGK-------------LTGIDTTIHSNYNTY-HH--FK---SLMERGVL-----------------TRDDVER
303229466	*Veillonella atypica* ACS-134-V-Col7a	LY-SEFMLLN-ELNNVRI---------------------------------DGKALAQGVKQHLIDSIFK-----QDHKK------MT---KNR----IE-------LFLKDNN---YITKKHKPE-------------ITGLDGEIKNDLTSY-RDMV------RILGNNF--------------------DVSMAED
34762592	*Fusobacterium nucleatum* ATCC 49256	LY-SEYIILN-ELNKVQV---------------------------------NDEFLNEENKRKIIDELFK-----E-NKK------VS---EKK----FK-------EYLLVNQI--ANRTVE--------------LKGIKDSFNSNYVSYIKFKD------IFGEKLNLDI------------------YKEISEK
374307738	*Filifactor alocis* ATCC 35896	LY-SKYMVLN-ELNNVKV---------------------------------RGKKLPTSLKQKVFEDLFE-----N-KSK------VT---GKN----LL-------EYLQIQDK--DIQIDD--------------LSGFDKDFKTSLKSY-----LDFKKQIFGEEIEKES--------------IQNMIED
320528778	*Solobacterium moorei* F0204	LY-SKYNVLN-EINPIKV---------------------------------NGKAIPVEVKQAIYTDLFE-----NSKK-------VT---RKS----IY-------LYLLKNG---YIEKEDI------------VSGIDIEIKSKLKSH-HD--FT---QIVQENKC-----------------TPEEIER
291520705	*Coprococcus catus* GD-7	VY-SKFMVLN-ELNNLRL---------------------------------NGEKISVELKQRIYEELFC-----K-YRK------VT---RKK----LE-------RYLVIE---GIAKKGVE------------ITGIDGDFKASLTAY-HD--FKE--RLTDVQL-------------------SQRAKEA
42525843	*Treponema denticola* ATCC 35405	LY-SEYTVLN-EINNLQI---------------------------IIDGKNICDIKLKQKIYEDLFK-----K-YKK------IT---QKQ----IS-------TFIKHEG---ICNKTDEVI-------------ILGIDKECTSSLKSY------IELKNIFGKQVDEIS----------------TKNMLEE
304438954	*Peptoniphilus duerdenii* ATCC BAA-1640	IY-NEFMVLN-ELNNLKL---------------------------------NEFLITEEMKKAIFEELFK-----T-KKK------VT---LKA----VS-------NLLKK----EFNLTGDIL-------------LSGTDGDFKQGLNSY------IDFKNIIGDKVDRDD-----------------YRIKIEE
224543312	*Catenibacterium mitsuokai* DSM 15897	IV-SKYEVYN-ELNKIRV---------------------------------DDKLLEVDVKNDIYNELFM-----K-NKT------VT---EKK----LK-------NWLVNNQ---CCSKDAEIK-----------GFQKENQFSTSLTPW-IDFT-----NIFGKIDQS------------------NFDLIEN
116628213	*Streptococcus thermophilus* LMD-9	LY-ETFNVYN-ELTKVRF-----------------------IAESMRDYQFLDSKQKKDIVRLYFK-----D-KRK------VT---DKD----II-------E-YLHAIY---GYDGIE---------------LKGIEKQFNSSLSTYHDLLNIINDKEFLDDSS-------------------NEAIIEE
24379809	*Streptococcus mutans* UA159	LY-EKFTVYN-ELTKVKY-----------------------KTEQ-GKTAFFDANMKQEIFDGVFK-----V-YRK------VT---KDK----LM-------DFLEKEFD---EFRIVD---------------LTGLDKENKVFNASYGTYHDLCKIL-DKDFLDNSK-------------------NEKILED
13622193	*Streptococcus pyogenes* SF370	LY-EYFTVYN-ELTKVKY-----------------------VTEGMRKPAFLSGEQKKAIVDLLFK-----T-NRK------VT---VKQ----LK-------EDYFKKIE---CFDSVE--------------ISGVEDRFNASLGTYHDLLKIIKDKDFLDNEE-------------------NEDILED
310286728	*Bifidobacterium bifidum* S17	LY-QEYEVLN-ELNNVRL--------------------SVRTGNHWNDKRRMRLGREEKTLLCQRLFM-----K-GQT------VT---KRT----AE-------NLLRKEY---GRTYELS---------------GLSDESKFTSSLSTY------GKMCRIFGEKYVNE-----------------HRDLMEK
366983953	*Oenococcus kitaharae* DSM 17330	LY-QKFDVLQ-ELNNIRV---------------------------------SGRRLDIRAKQDAFEHLFK-----V-QKT------VS---ATN----LK-------DFLVQAGYISEDTQIE-------------GL-ADVNGKNFNNALTTY------NYLVSVLGREFVENPS----------------NEELLEE
422884106	*Streptococcus sanguinis* SK49	IY-EEVMLQN-ELTRVKY------------------KDKYG------KAHFFDSELRQNIINGLFK----NN-SKR------VN---AKS----LI-------KYLSDNHKDL--------NAIEIV---------SGVEKGKSFNSTLKTY------NDLKTIFSEELLDSEI--------------YQKELEE
339625081	*Fructobacillus fructosus* KCTC 3544	RY-QLFTVLN-ELNNVRV---------------------------------NGKKFDSKTKADLINDLFK-----A-RKT------VS---LSA----LK-------DYLKAQG---------KGDVTI----------TGLADESKFNSSLSSY------NDLKKTFDAEYLENED------------------NQETLEK
306821691	*Eubacterium yurii* ATCC 43715	LY-EKFMVLN-EINNIKI---------------------------------DGEKISVEAKQKIYNDLFV-----K-GKK------VS---QKD----IK-------KELISLNIM---------DKDSVL----------SGT------DTVCNAY------LSSIGKFTGVFKEEINKQS-----------IVDMIED
336394882	*Lactobacillus farciminis* KCTC 3681	IY-QRYEVLN-ELNNIRI---------------------TENLK---TNPIGSRLTVETKQRIYNELFK-----K-YKK------VS---VKK----LT-------KWLIAQGYY---------KNPIL----------IGLSQKDEFNSTLTTY------LDMKKIFGSSFMEDNK-------------NYDQIEE
323463801	*Staphylococcus pseudintermedius* ED99	LY-QEMEVLN-ELNATQI---------------------RLQTD---PKNRKYRMMPQIKLFAVEHIFK-----K-YKT------VS---HSK----FL-------EIMLNSNHRENFMNH--GEKLSI----------FGTQDDKKFASKLSSY------QDMTKIFGDIEG-------------------KRAQIEE
389815359	*Planococcus antarcticus* DSM 14505	TY-ERFEVLN-ELNGIQL---------------------RTTGA---ESDFRHRLSYEMKCWIIDNVFK-----Q-YKT------VS---TKR----LL-------QELKKSPYADELYDEHTGEIKEV---------FGTQKENAFATSLSGY------ISMKSILGAVVDD------------------NPAMTEE
258509199	*Lactobacillus rhamnosus* GG	LY-QKYEVLN-ELNNVRI---------------------------------NNECLGTDQKQRLIREVFE-----R-HSS------VT---IKQ----VA-------DNLVAHGD---FARRPEIR-------------GLADEKRFLSSLSTY----------HQLKEILHEAIDDPT-----------KLLDIEN
169823755	*Finegoldia magna* ATCC 29328	LY-SEYMVLN-ELNNIRV---------------------------------NGKPLDTDVKLKIIEELFK-----K-KTK------VT---LKS----IR-------DYMVRNN---FADKEDFD-------------NSEKNLEIASNMKSY-ID--FN---NILEDKF--------------------DVEMVED
Jnet	227501312	HH-HHHHHHH-H---EEE---------------------------------HHHHHHHHHHHHHH------------------HHH----HH-------HHHHH--------------EE----------------EEE---------HHH-HH--HH---HHHHHHH----------------------H
47458868	*Mycoplasma mobile* 163K	SA-KVFDITN-KLTDWKH---------------------------------KNEYISERLKRKILLSRFLN-----KDSKS-------AVEKILKEENIKFE--------NLSEIAY---NKDDNKINLPIINAYHSLTT----IFKKHLINFENYLIS-NENDLSKLMSFYKQQSEKLFVPNEKGSYEINQNNNVLHIFDA
284931710	*Mycoplasma gallisepticum* str. F	IA-MIFNEIN-ELSTIRS--------------------------YSIYLTGWFINQEFKKAYLNKLLDLLIKTNGEKP-------IDARQFKKLREETIAESI----GKETLKDVENEEKLEKEDHKWKLKGLKLNT----NGKIQYNDLSSLAKFVHKLKQHLKLDFLLEDQYATLDKINFLQSLFVYLGKHLRYSNR
71894592	*Mycoplasma synoviae* 53	SA-LIFNLQN-EICTIKN----------------------------EFTEFKNWWLNAEQKSEILKFVFTELFNWKDKKY-------SDKKFNKNLQDKIK--------KYLLNFA---LENFNLNEEILKNRDLENDTVLGLKGVKYYEKSNATADAALEFSSLKPLYVFIKFLKEKKLDLNYLLGLE-----NTEILYF
363542550	*Mycoplasma ovipneumoniae* SC01	SY-EIFNLLN-QLINLST-------------------DLKT------TNKKIWQLSSNDRNELLDELLKVKEKAKI-----------ISISLKKNEIKKII----------------LKDFGFEKSDIDDQD-------TIEGRKIIKEEPTT---KLEVTKHLLATIYSHSSDS------NWININN-----ILEFLPY
384393286	*Mycoplasma canis* PG 14	SA-LIFNFLN-ELANIRL-------------------YSTD------KKNIQPLWKLSSVNTKLNLLFNLPISEK-KKKL------TSTNINDIVKKESI--------KSIM-IS---VEDIDMIKDEWAGKE-PNVYGVGLSGLNI--ESSAK-ENKFKFQDLKILNVLINLLDNVGIKFEFKDRND-----IIKNLEL
238924075	*Eubacterium rectale* ATCC 33656	TA-QEYNVLN-DLNNLTI---------------------------------NGRKLEENEKHEIVERIKS-----S--NT------IN---------MR-------KIISDCMGENIDDFAGAR-------------IDKSGKEIFHKFEVY-----------NKMRKALLEIGIDISNY----------SREELDE
315149830	*Enterococcus faecalis* TX0012	SA-QQYNLLN-DLTNLKI---------------------------------DGEVPLSSEQKEYILTELMT-----KEFTR------FG---VND----VV-------KLLGVK---KERLSGWR-------------LDKKGKPEIHTLKGY-----------RNWRKIFAEAGIDLATL---------PTETIDC
116627542	*Streptococcus thermophilus* LMD-9	TA-QEFNLLN-DLNNLTV---------------------------------PTETKKLSKEQKNQIINYVKN-----E--KA------MG---PAKLFKYIA-------KLLSCD---VADIKGYR-------------IDKSGKAEIHTFEAY-----------RKMKTLETLDIEQMD------------RETLDK
315659848	*Staphylococcus lugdunensis* M23590	SA-DLFNALN-DLNNLVI---------------------------------QRDGLSKLEYHEKYHIIENVFK-----QKKKP------T---LKQ----IA-------NEINVN---PEDIKGYR-------------ITKSGKPQFTEFKLY-----------HDLKSVLFDQSILE------------NEDVLDQ
160915782	*Eubacterium dolichum* DSM 3991	SA-ELFNLLN-DLNNLSI---------------------------------EGEKLTSEQKAMILKIVHE-----K--GK------IT---PKQ----LA-------KEVGVS---LEQIRGFR-------------IDTKGSPLLSELTGY-----------KMIREVLEKSNDEHLE----------DHVFYDE
325677756	*Ruminococcus albus* 8	IA-DIYAFVN-VLSQYTY---------------------------------TNNRGESVFDTSFANDLINSALK-----N--GS------MD---KRE----LK-------AFVIVN---AKSYHIDIS------------DKNSDTSLTKCFKYI-----------KVVKPLFEKY--GYDWDKLIENYTDTDNNVLNR
225377804	*Roseburia inulinivorans* DSM 16841	TA-ELFVALQ-KINHTKL---------------------------------IDEFGTGRFFSEEERKTIIGLLLS-----S--KE------LK---YGT----IR-------KKLNIDP---SLKFNSLNYS---------AKKEGETEEERVLDTEKAKF------------ASMFWTYEYSKCLKDRTEEMPVGEKADLFDR
336393381	*Lactobacillus coryniformis* KCTC 3535	TF-QYFNLLQ-KMTSLNY---------------------------------QNTTGDTWHTLNGLDRQAIIDAVFA-----KAEKP---TKTYKPTDFGE---LR-------KKLLLPD---DARFNLVNYGSLQTQKEI------ETVEKKTRFVDFKAY-----------HDLVKVLPEEMWQ-----------SRQLLDH
310780384	*Ilyobacter polytropus* DSM 2926	SG-ERFVFLQ-KLNNFRI---------------------------------VGLSGKRPLTEERDIVEKEVYL-----K--KE------VR---YEK----LR-------KILYLKE---EERFGDLNYS-----------------KDEKQDKKTEKTKF-------------ISLIGNYTIKKLNLSEKLKSEIEEDKSKLDK
301311869	*Bacteroides* sp. 20 3	LY-QEFRLWQ-FIVNLRI---------------------------------YRKETDVDVTQELLP-----T-EAD------YVT----LF-------EWLNEKKEIDQKAFFKYPP------FGFKK----TTSNYRWNYVEDKPY-PCNETHA--QIIARLGKAHIPKAFL------------SKEKEET
385811609	*Ignavibacterium album* JCM 16511	LY-QEFRIWQ-DIHNIKV---------------------------------IRKESEVNGKKKINI-----D-ETQL---------YINENIKEKLF-------ELFNSKDSLSEKDILELISLNIINSGIKISK-KEEETHRINLFANRKELKGNETKS--RYRKVFKKLGFDGEYILN--------HPSKLNR
60683389	*Bacteroides fragilis* NCTC 9343	LF-QEFKIWQ-ILNNIEVTVVGKKRKRRKLKENYSALFEELNDAEQLELNGSRRLQCEEKALLAQELFI-----R--DK------MT---KSE----VL-------KLLFDNPQELDLNFKTIDGNKTGY----------ALFQAYSKMIEMSGH-EPVDFKKPVEKVVEYIKAVFDLLNWNTDILGFN--SNEELDN
319957206	*Nitratifractor salsuginis* DSM 16511	LY-DLYRLYK-KLADLNI---------------------------------DGYEVTQEDREKVIEWVEKKIAQGKNLKK-------IT---HKD----LR-------KILGLAP---EQKIFGVED------ERIV------KGKKEPRTFVPFFFL-AD--IAKFKELFASIQKHPD---------------ALQIFRE
187250660	*Elusimicrobium minutum* Pei191	LN-EERRLWE-TLNNARY-------------------SDPIVDIVTGEITGYYEKQFTKEQKQKLFDYLLT-----G--SE------IL---PAQ----TK-------KKLLEVS---NTNFEDI----------------ILQGRDKKAQKIKGY-----------KLIKLES-----MPFWARL---------SEAQQDS
325972003	*Sphaerochaeta globus* str. Buddy	LN-EERRLWE-AINNARI---------------------------------KMPMQEGAAKRYQSASFS-----DEQRH------IL---FHI----AR-------SGTDITPKLVQKEFPALKTSII------------VLQGKEKAIQKIAGF---------------RFRRLEEKSFWKRL-------SEEQKDD
296446027	*Methylosinus trichosporium* OB3b	SF-ELFRFLS-RLNHVTL---------------------------------RDGKQERTLTRDELALAAADFGA-----A--AK------VS---FTA----LR-------KALPLKPE---TTVFVGVK---------------ADEESKLDVVARSGK------AAEGTARLRSVIVDALGELAWGALLC-----SPEKLDK
347536497	*Flavobacterium branchiophilum* FL-15	DF-EEYRMWT-YLNTIKI-------------------------GTQSDKKLRFLTQDEKLKLVPK-FYR-----K--ND------FN---FDV----LA-------KELIEK---GSSFGFYKSSKKNDFF-------YWFNYKPTDTVAACQVAASLKNAIGEDWK--------TKSFKYQTINSNKEQVSRTVDY
345885718	*Prevotella* sp. C561	LF-EKFRAWT-LINNIKV-------------------------RMSVDTLDEQLPMKLRLDLYNECFLAFV--R--TE------FK---FED----I--------RKYLEK---RLQIHFSYNDKT------------INYKDSTSVAGCPITARFRKMLGEEWE------SFRVEGQKERQAHSKNNISFHRVSY
282880052	*Prevotella timonensis* CRIS 5C-B1	DY-EEFMLC-FVNNIQV-------------------------KGPHDLELRPLTYEEREKIEPL-FFRKS--K--PN------FD---FED----IA-------KALAGK----K---NYAWIHDKEERA-------YKFNYRMTQGVPGCPTIAQLKSIFGDDWK-----TGIAETYTLIQKKNGSKSLQEMVD-
312879015	*Aminomonas paucivorans* DSM 12260	SA-ETFMLLQ-KTGNLRL---------------------------------IEEGRKRPLTEEQRQIHLLAWK-----Q--EK------VT---HKT----LR-------RHLEIPE---GLFGPLYHRS------------GDKAEEKLFVHLAGI-----------HEIRKALDKGPDPAVWDTLRS-----RRDLLDS
294086111	*Candidatus Puniceispirillum marinum* IMCC1322	SF-QRFRIYQ-ELSNLAW-------------------------IDHDGVAHRITASLALRDHLFDELEH-----K--KK------LT---FKA----MR-------AILRKQGV--VDYPVGFNL--------------ESDNRDHLIGNLTSC---------------IMRDAKKMIGSAWDRL-------DEEEQDS
330822845	*Alicycliphilus denitrificans* K601	ST-QRFRIHQ-EVNHLRL---------------------------------LDENLREVALTTAEQRQLLAHV-----K--AK------LS---FEQ----IR-------KLLKLSG---SVQFN------------------LEDAKRTLKGNATS-----------AALARKELFGAAWSGF------DEALQDE
344171927	*Ralstonia syzygii* R24	SA-QRFRLMQ-ELNHLRV-------------------------MTLADKRERPLSFQERNDLLAQLVA-----R--PK------CG---FDM----LR-------KIVF-------GANKEAYRF--------------TIESERRK--ELKGC-------------DTAAKLAKVNALGTRWQAL-------SLDEQDR
159042956	*Dinoroseobacter shibae* DFL 12	LT-QRRVLYE-TVNQLRV-------------------------RTISGDQRLTREERDQVIHALDN-----KKPTK-SLSSMVLK---LPA----LA-------KVLKRDGERFTLETGVRD-----------AIACDPLRASPAHP------------DRFGPRWSILDADAQW------------EVISR
83591793	*Rhodospirillum rubrum* ATCC 11170	TV-QDFIIRQ-TLANLRL-------------------------PSTSADEPRPLTDEEHAKALALLST-----ARFVE----------WPA----LR-------RALGLKR---GVKFTAETE---------------RNGAKQAARGTAGNL-------------TEAILAPLIPGWSGW--------DLDRKDR
288957741	*Azospirillum* sp. B510	SA-QRLRLFQ-ELASLRV-------------------------IHLDLSERPLTDEERDRIVAFVQG-----RPPKAGRKPGKVQKSVPFEK---IR-------GLLELPP---GYEFNSLTG----------------ESDKRPELLGDETGA----------------RIAPAFGPGWTAL-------PLEEQDA
427429481	*Caenispirillum salinarum* AK4	LI-QALRIRE-TVGNLRLHE---------RITEPDGRQRYVPRAMPELGLSHGELTAPERDTLVRALMH-----DPDGLAAKDGR----IPYTR----LR-------KLIGYDNS--PVCFAQ------------------ERDTSGGGITVNPTD----------------PLMARWIDGWVDL-------PLKARSL
92109262	*Nitrobacter hamburgensis* X14	LA-QRFRILS-EARNLEI-------------------------RDTGKGSRRTLTKEQSDLVVAALA-----N--RE------VK---FDK----LR-------TLLKLPA---EARFNL-----------------ESDRRAALDGDQTA-------------ARLSDKKGF---NKAWRGF--------PPERQIA
148255343	*Bradyrhizobium* sp. BTAi1	LA-QRFRIWQ-DVRNLAV-------------------------VETGPTSSRLGKEDQDKVARALLQ-----T--DQ------LS---FDE----IR-------GLLGLPS---DARFNL-----------------ESDRRDHLKGDATG-------------AILSARRHF---GPAWHDR-------SLDRQID
34557790	*Wolinella succinogenes* DSM 1740	TA-EKFIAISKFFSTVII-------------------------DNEGWEQKIIERKTLEELLDFAVS-----R--EK------VE---FRH----LR-------KFLDLSD---NEIFKGLHYKGKPKTAK---------KEATLFDPNEEL-IFDKVEAEKKAWISLRGAAKLREALGNEFYGRFVALGKHADE
218563121	*Campylobacter jejuni* NCTC 11168	LA-FMFVALTRIINLLNN-------------------------LKNTEGILYTKDDLNALLNEVLK-----N--GT------LT---YKQ----TK-------KLLGLSD---DYEFKG---------------------EKGTYFIEFKKYKEF-IK--------ALGEHNL-------------------SQDDLNE
Jnet	218563121	HH-HHHHHHHHHH--EEE---------------------------------HHHHHHHHHHHHHH------------------HHH----HH-------HHH---HHH-HH--------H-------------------------------HHHHH
291276265	*Helicobacter mustelae* 12198	SA-EEFVALTKSINFLKN-------------------------LTNRHGLCFSQEDMCVYLGKILQ-----EAQKN----EKGLT---YSK----LK-------LLLDLPS---DFEFLGLDYS-------------GKNPEKAVFLSLPST------------FKLNKITQ-------------------DRKTQDK
222109285	*Acidovorax ebreus* TPSY	SV-ERHVWLT-RLNNLRI---------------------------------VVDGRSRPLNEAERQAALLLPYQ-----TETSK------------YKT----LK-------NAFIKAGLWGDGVRFGGLAYPSQAQIDAEK------TKDPFDQFLVKLPAW----------HELRKAFKAAGHEALWQQISTPALDGDPTLLDQ
365156657	*Bacillus smithii* 7 3 47FAA	TF-QSFIVWE-HINKLRL---------------------------------VSPDETRALTEIERNLLYKQAFS-----K--NK------MT---YYD----IR-------KLLNLSD---DIHFKGLLYDPKSS--------------LKQIENIRFLELDSY------------HKIRKCIENVYGKDGIRMF-------NETDIDT
220930482	*Clostridium cellulolyticum* H10	TS-EYFGLLQ-SINNLVL---------------------------------VEDNNTLTLNNDQRAKIIEYAHF-----K--NE------IK---YSE----IR-------EILFKAHNLTHKNP------------SGNNESKKFFKYMPSY------------HKLKSTLPTDIWGKLHS---------NKESLDN
297182908	uncultured delta proteobact. HF0070 07E19	ST-EWFKFLQ-NLGNLQI---------------------------------SNAYREEWSIDAPRRAQIIDACSQ-----R--ST------SS---YWQ----IR-------RDFQIPD---EYRFNLVNYERRDP--------------DVDLQEYLQQQERKTLA-----NFRNWKQLEKIIGTGHP------------IQTLDE
154250555	*Parvibaculum lavamentivorans* DS-1	LS-QQRRMLE-KLNNLAI---------------------------------AGGNARPLDAEERDAILSKLQQ-----Q--AS------MS---WPGVRSALK-------ALYKQRGEPGAEKLSKFNL-------------ELGGESKLLGNALEA-KLADMFG--PDWPAHPRKQEIRHAVHERLWAADYGETPDKKR
218767588	*Neisseria meningitidis* Z2491	TA-ERFIWLT-KLNNLRI---------------------------------LEQGSERPLTDTERATLMDEPYR-----K--SK------LT---YAQ----AR-------KLLGLED---TAFFKGLRYGKDN-------------------AEASTLMEMKAY-----------HAISRALEKEGLKDKKSPLNL-----SPELQDE
15602992	*Pasteurella multocida* str. Pm70	SA-ERFVWLT-KLNNLRI---------------------------------LEDGAERALNEEERQALINHPYE-----K--SK------LT---YAQ----VR-------KLLGLSE---QAIFKHLRYSKEN-------------------AEASTFMELKAW-----------HAIRKALENQGLKDTWQDLAK-----KPDLLDE
187736489	*Akkermansia muciniphila* ATCC BAA-835	EF-YEYRMAR-ILCNIRA---------------------------------DGEPLSAEIRRELMNQARQ-----E--GK------LT---KAS----LE-------KAISSRLGKETETNVSNYF-----------------TLHPDSEEALYLNPAVEVLQRSGIGQILSPSVYRI----------------AANRLRR
315605738	*Actinomyces* sp. oral taxon 180 str. F0310	AF-QKFKVVA-TLANMRI---------------------------------REQSAG-ERSLTSEELNRVAFTLN-----HTESES------PT---WDD----VA-------RRLRGSSRAS---------------LETGGGLTYPPVDDT---------TVRVMSAEVDWLADWWDCANDESRGHMIDAISN
117929158	*Acidothermus cellulolyticus* 11B	EF-QEYRIVA-AVANLRI---------------------------------RDGSGSRPLSLEERNAVIEALLA-----QTERS------LT---WSD----IA-------LEILKLPN---ESDLTSVPE-----------EDGPSSLAYSQFAPF-DETSARIAEFIAKNRRKIPTFAQWWQEQ--------DRTSRSD
189440764	*Bifidobacterium longum* DJO10A	AF-QEYRIAN-VITNLRI---------------------------------KDASAELRKLTVDEKSQIYDQLVS-----PSSED------IT---WSD----LC-------DFLGFK---RSQLKGVGS----------LTEDGEERISSRPPR-----LTSVQRIYESDNKIRKPLVAWWKSA-------SDNEHEA
403744858	*Alicyclobacillus hesperidum* URH17-3-68	SF-EERFQLLT-KVVNLRI--YRQQ---EDGGRYPC------------------ELTQTERARVI-DCAY-----EQTK------IT---YGK----LR-------KLLDMK---DTESFAGLTYGLN----------------RSRNKTEDTV------FVEMKFYH-EVRKALQRAGVFIQD------------LSI
407803669	*Alcanivorax* sp. W11-5	AA-QNFRIEK-QMADLRW-----------GMGRRAE-------------------MLNDHQKAVIRELLN-----QQKE------LS---FRK----IY-------KEL--E----RAGCPGPEGKGLNMDRAAL--------GGRDDLSGNTT-----LAAWRKLGLEDRWQELDEVTQIQVINFLA---DLGSPE
423317190	*Bergeyella zoohelcum* ATCC 43767	VF-QEYKVWE-QINKLIV--NTKIEAGTNRKGEKKYKYIDR-------------PIPTALKEWIFEELQN-----KKE------IT---FSA----IFKKL---KAEFDLR----EGIDFLNGMSPKD-----------------------KLKGNET-----KLQLQKSL-----------GELWDVLGL--------DSI
402847315	*Porphyromonas* sp. oral taxon 279 str. F0450	LF-QLCCIYE-AVNNIRL----------TRPNGSPC------------------DITPEERAKIV-AHLQ-----SSAS-------LS---FAA----LKKLL---KEKALIA----DQLTSKSGLKGNSTRVALA---------SQLAPYQPYKH-----LLDMELETRMMTVQLTDEETGEVTEREVAV-VTDSYVR
404487228	*Barnesiella intestinihominis* YIT 11860	LF-QVCRLWE-SINNIVV--K------DRRNEIVF-----------------ISAEQRAALF-DFLN-----THEK------LK---GSD----LLKLL--GLSKTYGYR----LGEQFKTGIQGNKTRVEIE----------RALGNYPDKKR------LLQFNLQEESSSMVNTETGEIIPM------ISLSFEQ
374384763	*Odoribacter laneus* YIT 12061	EF-EEFRAYQ-FINNIIY--------------GKNEH-----------------LTAIQREAVFELMCT-----ESKD------FN---FEK----IPKHL------K-------KFEKFNFDDTTKV--------------------PACTT------ISQLRKLF--------PHPVWEE---------------
384109266	*Treponema* sp. JC4	CS-QKLRILQ-DIGNLAY--YE------GGSKKRVE------------------LNDNQDKVLY-ELLN-----SKDK------VT---FDQ----MRKAL---------CLA----DSNSFNLEENRDF------------------LIGNPT------AVKMRSKN--------RFGKLWDE-------------IPL
402849997	*Rhodovulum* sp. PH10	SV-EARGIYE-RLAHLRI--T-------TGPVSDRG------------------LTRPERDVLASALLA-----GKS------LT---FKA----VRKTL------KIL-----PHALVNFEEAGEK---------------GLDGALTAK------LLSKPDHY----------GAAWHG-----------LSF
Jnet	331001027	HH-HHHHHHH-----HHHHHH---------------------------------HHHHHHHHHHHHH---HHH-----HHHHHHHHHHHH-HHHHH------------------------HHHHHHH-------HHHHHHHHHHHHH---------HHHHHHHHHHHH---------HHHHHHHHHHHHHHHHH---------H
331001027	*Parasutterella excrementihominis* YIT 11859	IL-QRAFDRS-IALDECSIRR---------------------TAEDFENGVVIKNEKLEDVLSGHQLE------EFLEFANRYYQE-TAKAKNG-LW-----------------------FP-ENALLEERADLHPPMKNKILNVIVGQALGV---SPAEGTDFIEEIWNS--KVKGRSTVRSICNAIENERKTGYPFSEDYK
34557932	*Wolinella succinogenes* DSM 1740	LL-HRLLDTS-KEIDIYELR------------------------GKKPNELLVK-------TLGQSDAN------RLYGFAQNYYELIRQKVRAG-IWV----------PVKNKDDSLN--LEDNSNMLKRCNHNPPHKKNQIHNLVAGILGV-KLDEAKFAEFEKELWSA---KVGNKKLSAYCKINEELRKTHGNTFKIDIE
54296138	*Legionella pneumophila* str. Paris	IL-QRYLDIN-KKIDKFKIKKQLSFLG-------------QGKQLPANLIETQKEMETHFNSSLVS-------VLIQIASAYNKE-REDAAQG-LW-----------------------FDNAFSLCELSNINPPRKQKILPLLVGAILSEDFINNKDKWAKFKIFWNT--HKIGRTSLKSKCKEIEEARKNSGNAFKIDYE
319941583	*Sutterella wadsworthensis* 3 1 45B	AL-QRAFDRS-KALDPYALRAL------------------AAGSKSNKLTSARTALENCIGGQNVK------TFLDCARRYYRE-ADDAKVG-LW-----------------------FDNADGLLERSDLHPPMKKKILPLLVANILQT---DETTGQKFLDEIWRK--QIKGRETVASRCARIETVRKSFGGGFNIAYN
254447899	gamma proteobact. HTCC5015	AL-HRIVERS-RLSDPYQLRLLIS--------------------ITDAEKRNDLAGYKRLKLSLGSEVDE------FLLLVKNIVDE-TKEAREG-LW-----------------------FETENKLFFKCGKTPPRKEKLKSTLLSAVLGK-NLSDDEQSSFIEEFWKSGTPKIERRNVRGWCRLASQVQKTYGVYLK-EYG
118497352	*Francisella novicida* U112	ILKQFIFDRV-KASDELLLNEIYFQAKKL---QKASSELEKLESSKKLDEVIANSQLSQILKSQHTNGIFEQGTFLHLVCKYYKQ-RQRARDSRLYIMPEYRYDKKLHKYNNTGR--FDDDNQLLTYCNHKPRQKRYQLLNDLAGVLQVSPNFLKDKIGSDDDLFISKWLVEHIRGFKKACEDSLKIQKDNRGLLNHKIN
Motifs		____
informative positions		**-*******-*******--

```
227494853   Actinomyces coleocanis DSM 15436#            -----------AEK----LTEGTAAEV------EVAEFLQNL--------------SDEDNEK-LDSF---SLPIG------RAAYSVDSLERLTKRMI------ENGE----DLFEAR-------------------------------------------------------------------------------------------------------
328956315   Coriobacterium glomerans PW2                 ----------IILW----NTLF-EDRK------ILSQRLKEEYGSR----LS-----AEQIKT--ICKK----RFTG------WGRLSEKFLTGITVQVD------EDSV----SIMDVL-------------------------------------------------------------------------------------------------------
227824983   Acidaminococcus sp. D21                      ----------IVER----MTYS-DDTK------RVRLWLNNNYGT----LT-----ADDVKH--ISRL----RKHD------FGRLSKMFLTGLKGVHK------ETGE----RASILD--------------------------------------------------------------------------------F-----------------------
303229466   Veillonella atypica ACS-134-V-Col7a          ----------IITD----ITIFGESKK------MLRQTLRNKFGSQ----LN-----DETIKK--LSKL----RYRD------WGRLSKKLLKGIDGCDK-------AGN----GAPKTI------------------------------------------------------------------------------I------------------------
34762592    Fusobacterium nucleatum ATCC 49256           ----------SILW----KCLYGDDKK------IFEKKIKNEYGDI----LN-----KDEIKK--INSF----KFNT------WGRLSEKLLTGIEFINL------ETGE----CYSSVM-------------------------------------------------------------------------------------------------------
374307738   Filifactor alocis ATCC 35896                 ----------IIKW----ITIYGNDKE------MLKRVIRANYSNQ----LT-----EEQMKK--ITGF----QYSG------WGNFSKMFLKGISGSDV------STGE----TFDIIT-------------------------------------------------------------------------------------------------------
320528778   Solobacterium moorei F0204                   ----------IIKG----ILVYSDDKS------MLRRWLKNNIKG----LS-----ENDVKY--LAKL----NYKE------WGRLSKTLLTDIYTINF------EDGE----ACSILD----------------------------------------------------------------------------I-----------------------
291520705   Coprococcus catus GD-7                       ----------IVLN----VVLFGDDKK------LLKQRLSKMYPN----LT-----TGQLKG--ICSL----SYQG------WGRLSKTFLEEITVPAP------GTGE----VWNIMT--------------------------------------------------------------------------A-----------------------
42525843    Treponema denticola ATCC 35405               ----------IIRWAT--IYDEGEKT------LLKTKIKAEYGKY----CS-----DEQIKK--ILNL----KFSG------WGRLSRKFLETVTSEMP------------------GFSEPV-------------------------------------------------------------------------------------------------------
304438954   Peptoniphilus duerdenii ATCC BAA-1640        ----------IIKL----IVLYEDDKT------YLKKKIKSAYKND----FT-----DDEIKK--IAAL----NYKD------WGRLSRKFLTGIEGVDK------TTGE----KGSIIY-------------------------------------------------------------------------------------------------------
224543312   Catenibacterium mitsuokai DSM 15897          ----------IIYD----LTVF-EDKK------IMKRRLKKKYA------LP-----DDKVKQ--ILKL----KYKD------WSRLSKKLLDGIVADNR-------FGS----SVTVLD-------------------------------------------------------------------------------------------------------
116628213   Streptococcus thermophilus LMD-9            ----------IIHT----LTIF-EDRE------MIKQRLSKFEN------IF-----DKSVLKK-LSRR----HYTG------WGKLSAKLINGIRDEKS-----GNTIL----DYLIDD-------------------------------------------------------------------------------------------------------
24379809    Streptococcus mutans UA159                   ----------IVLT----LTLF-EDRE------MIRKRLENYSD------LL-----TKEQVKK-LERR----HYTG------WGRLSAELIHGIRNKES-----RKTIL----DYLIDD-------------------------------------------------------------------------------------------------------
13622193    Streptococcus pyogenes SF370                 ----------IVLT----LTLF-EDRE------MIEERLKTYAH------LF-----DDKVMKQ-LKRR----RYTG------WGRLSRKLINGIRDKQS-----GKTIL----DFLKSD-------------------------------------------------------------------------------------------------------
310286728   Bifidobacterium bifidum S17                  ----------IVEL----QTVF-EDKE------TLLHQLRQLEG------IS-----EADCAL--LVNT----HYTG------WGRLSRKLLTTKAGECKI--SDDFAPR----KHSIIE-------------------------------------------------------------------------------------------------------
366983953   Oenococcus kitaharae DSM 17330               ----------ITEL----QTVF-EDKK------VLRRQLDQLDG------IS-----DHNREK--LSRK----HYTG------WGRISKKLLTTKIVQNAD-KIDNQTFDVPRMNQSIID-------------------------------------------------------------------------------------------------------
422884106   Streptococcus sanguinis SK49                 ----------IIKV----ITVF-DDKK------SIKNYLTKFFG--HLEILD-----EEKINQ--LSKL----RYSG------WGRYSAKLLLDIRDED----------T----GFNLLQ-------------------------------------------------------------------------------------------------------
339625081   Fructobacillus fructosus KCTC 3544           ----------IIEI----QTVF-EDSK------IASREL-SKLP------LD-----DDQVKK--LSQT----HYTG------WGRLSEKLLDSKIIDERG--------Q----KVSILD-------------------------------------------------------------------------------------------------------
306821691   Eubacterium yurii ATCC 43715                 ----------IIFL----KTVYGDEKR------FVKEEIVEKYG----DEID-----KDKIKR--ILGF----KFSN------WGNLSKSFLELEGADVGT--G-----E----VRSIIQ-------------------------------------------------------------------------------------------------------
336394882   Lactobacillus farciminis KCTC 3681           ----------LIEW----LTIF-EDKQ------ILNEKLHSSKY-----SYT-----PDQIKK--ISNM----RYKG------WGRLSKKILMDITTETNT--PQLLQLS----NYSILD-------------------------------------------------------------------------------------------------------
323463801   Staphylococcus pseudintermedius ED99         ----------IIQW----ITIF-EDKK------ILVQKLKECYP-----ELT-----SKQINQ--LKKL----NYSG------WGRLSEKLLTHAY--------------Q----GHSIIE-------------------------------------------------------------------------------------------------------
389815359   Planococcus antarcticus DSM 14505            ----------LIYW----IAVF-EDRE------ILHLKIQEKYP-----SIT-----DVQRQK--LALV----KLPG------WGRFSRLLIDGLPLDE----------Q----GQSVLD-------------------------------------------------------------------------------------------------------
258509199   Lactobacillus rhamnosus GG                   ----------IITW----STVF-EDHT------IFETKLAEIEW------LD-----PKKINE--LSGI----RYRG------WGQFSRKLLDGLKLGN--------------GHTVIQ-------------------------------------------------------------------------------------------------------
169823755   Finegoldia magna ATCC 29328                  ----------LIEK----ITIHTGNKK------LLKKYIEETYPD-----LS-----SSQIQK--IINL----KYKD------WGRLSRKLLDGIKGTKK------ETE----KTDTVI--------------------------------------------------------------------------N------------------------
Jnet        227501312                                    ----------HHHH----HEE----HH------HHHHHHHH----------------HHHHHH--HHH----------------HHHHHHHHHHHHHH-----------H-----HHHHHH-------------------------------------------------------------------------------------------------------
47458868    Mycoplasma mobile 163K                       --------I--SNI----LNKF-S---------TIQDRIRILEGYFEFSNLK-----KDVKSSEIYSEIAKLREFSG------TSSLSFGAYYKFIPNLI------SEGS----KNYSTI-------------------------------------------------------------------------------------------------------
284931710   Mycoplasma gallisepticum str. F              --------VDSANL----KEFS-DSNK------LFERILQKQKDGLFKLFEQ-----TDKDDEK-ILAQ--------------THSLSTKAMLLAITRMT------NL----DNDEDN-------------------------------------------------------------------------------------------------------
71894592    Mycoplasma synoviae 53                       --------LDSIYLAISYSSDLKERNE-----WFKKLLKELYPKIKNNNLEIIENVEDIFE---ITDQEKFESFSK------THSLSREAFNHIIPLLL--------------SNNEGK-------------------------------------------------------------------------------------------------------
363542550   Mycoplasma ovipneumoniae SC01                --------LDAICIILDREKS-RGQDE-----VLKKLTEKNIFEVLKIDREKQLDFVKSIFS---NTKF----NFKK------IGNFSLKAIREFLPKMF---------------EQNK-------------------------------------------------------------------------------------------------------
384393286   Mycoplasma canis PG 14                       --------LDNLYLFLIYQKESNNKDS-----SIDLFIAKNESLNIENLKLKKEFLLGAGN---EFENHN----SK------THSLSKKAIDELIPKLL--------------DNNEGW-------------------------------------------------------------------------------------------------------
238924075   Eubacterium rectale ATCC 33656               ----------IGYI----MTIN-TDKE------AMMEAFQKSWID-----LS-----DDVKQCLINMRKTNGALFNK------WQSFSLKIMNELIPEMY-----AQPKE----QMTLLT-------------------------------------------------------------------------------------------------------
315149830   Enterococcus faecalis TX0012                 ----------LAKV----LTLN-TERE------GIENTLAFE--------LP-----ELSESVK-LLVLDRYKELSQSISTQSWHRFSLKTLHLLIPELM------NAT----SEQNTL-------------------------------------------------------------------------------------------------------
116627542   Streptococcus thermophilus LMD-9            ----------LAYV----LTLN-TERE------GIQEALEHE--------FA-----DGSFSQKQVDELVQFRKANSSIFGKGWHNFSVKLMMELIPELY-----ETSEE----QMTILT-------------------------------------------------------------------------------------------------------
315659848   Staphylococcus lugdunensis M23590            ----------IAEI----LTIY-QDKD------SIKSKLTELDI------LN-----NEEDKEN-IAQL---TGYTG------THRLSLKCIRLVLEEQW---------------YSSRN-------------------------------------------------------------------------------------------------------
160915782   Eubacterium dolichum DSM 3991                ----------IAEI----LTKT-KDIE------GRKKQISE---------LS-----SDLNEES-VHQLAGLTKFTA------YHSLSFKALRLINEEML-----KTELN----QMQSIT-------------------------------------------------------------------------------------------------------
325677756   Ruminococcus albus 8                         ----------IGIV----LSQA-QTPK------RREKLKALNIG------LD-----DGLIN-E-LTKL----KLSG------TANVSYKYMQGSIEAFC-------EGD----LYGKYQ-------------------------------------------------------------------------------------------------------
225377804   Roseburia inulinivorans DSM 16841            ----------IGEI----LTAY-KNDD------SRSSRLKELG-------LS-----GEEIDGL-LDLS-----PAK------YQRVSLKAMRKMQPYLE-------DGL----IYDKAC-------------------------------------------------------------------------------------------------------
336393381   Lactobacillus coryniformis KCTC 3535         ----------IGTA----LTLY-SSDK------RRRRYFAEELN------LP-----AELIE-K-LLPL----NFSK------FGHLSIKSMQNIIPYLE-------MGQ----VYSEAT-------------------------------------------------------------------------------------------------------
310780384   Ilyobacter polytropus DSM 2926               ----------IIEI----LTFN-KSDK------TIESNLKKLE-------LS-----REDIEIL-LSEE-----FSG------TLNLSLKAIKKILPYLE-------KGL----SYNEAC-------------------------------------------------------------------------------------------------------
301311869   Bacteroides sp. 20 3                         ----------LWHI----LYSI-EDKQ-----EEKTLEKALHSFANKNN---LS-----EEFIE-Q-FKNF---PPFKKE------YGSYSAKAIKKLLPLMR-------MGK----YWSIEN-------------------------------------------------------------------------------IDNGTRIRINKIID
385811609   Ignavibacterium album JCM 16511              ----------LWHS----DYSNDYADKE-----KTEKSILSSLGWKNRNGKWEKSKNYDVFNLPLEVAKAIANLPPLKKE---YGSYSALAIRKMLVVMR-------DGKYWQHPDQIAKDQENTSLMLFDKNLIQLTNNQRKVLNKYLLTLAEVQKRSTLIKQKLNEIEHNPYKLELVS------------------------
60683389    Bacteroides fragilis NCTC 9343              QPYYK-----LWHL----LYSF-EGDNTPTGNGRLIQKMTELYGF-----------EKEYATI-LANV----SFQDD-----YGSLSAKAIHKILPHLK-------GGN----RYDVAC-------------------------------------------------------------------------------------------------------
319957206   Nitratifractor salsuginis DSM 16511          ----------LAEI----LQRS-KTPQ------EALDRLRALMAGKG---ID-----TDDRE---LLEL-FKNKRSG------TRELSHRYIIEALPLFL-------EGY----DEKEVQ--------------------------------------------------------------------------R------IL----
187250660   Elusimicrobium minutum Pei191                ----------FLYD----WNSC-PEDK------LLTEKLSNEYH------LS-----EEEIDNA-FNEI---VLSSS------YAPLGKSAMLIILEKIK-------NDL----SYTEAV-------------------------------------------------------------------------------------------------------
325972003   Sphaerochaeta globus str. Buddy              ----------FFSA----WTNT-PDDK------RLSKYLMKHLL------LT-----ENEVVDA-LKTV---SLIGD------YGPIGKTATQLLMKHLE-------DGL----TYTEAL-------------------------------------------------------------------------------------------------------
296446027   Methylosinus trichosporium OB3b              ----------IAEV----ISFR-SDIG------RISEGLAQAGCNAP---LV-----DALTA---AASDGRFDPFTG------AGHISSKAARNILSGLR-------QGM----TYDKAC-------------------------------------------------------------------------------------------------------
347536497   Flavobacterium branchiophilum FL-15          --------KDLWHL----LTVA-TSDV------YLYEFAIDKLG------LD-----EKNAK-A-FSKT---KLKKD------FASLSLSAINKILPYLK-------EGL----LYSHAVFVANIENIV---DENIWKDEKQRDYIKTQISEIIENYTLEKSRFEIINGLLKEYKSENEDGK--RVYYSKEAEQSFENDL
345885718   Prevotella sp. C561                          ------SIEDIWHF----CYDA-EEPE------AVLAFAQETLR------LE-----RKKAE-E-LVRIWS-AMPQG------YAMLSQKAIRNINKILM-------LGL----KYSDAVILAKVPELVDVSDEELLSIAKDYYLVEAQVNY-------DKRINSIVNGLIAKYKSVSEEYRFADHNYEYLLDESDEKDI
282880052   Prevotella timonensis CRIS 5C-B1            ---------DVWNV----LYSF-SSVE------LKEFAHHKLQ-------LD-----EESAE-K-FAKI---KLSHS------FAALSLKAIRKFLPFLR-------KGM----YYTHASFFANIPTIV---GKEIWNKEQNRKYIMENVGE----------------LVFNYQPKHREVQ-----------------
312879015   Aminomonas paucivorans DSM 12260             ----------IADT----LTFY-KNED------EILPRLESLG-------LS-----PENAR-A-LAPL----SFSG------TAHLSLKALRKLLPYLE-------EGK----SYTQAR-------------------------------------------------------------------------------------------------------
294086111   Candidatus Puniceispirillum marinum IMCC1322  ----------FILM----LQDDQKGDD------EVRSILTQQYG------LS-----DDVAEDC-LDVR----LPDG------HGSLSKKAIDRILPVLR-------DQGL----IYYDAV-------------------------------------------------------------------------------------------------------
330822845   Alicycliphilus denitrificans K601            ----------IVWQ----LVTE-EGEG------ALIAWLQTHTG------LS-----EARAQAIVDVSL-----PEG------YGNLSRKALARIVPALR-------AAVI----TYDKAV-------------------------------------------------------------------------------------------------------
344171927   Ralstonia syzygii R24                        ----------LVCL----LLDG-ENDA------VLADALREHYG------LT-----DAQID-T-LLGL---SFEDG------HMRLGRSALLRVLDALESGRDEQGLPL----SYDKAV--------------------------------------------------------------------------V------------------------
159042956   Dinoroseobacter shibae DFL 12                -----------IRR----VQSD-AEHA------ALVDWLTEAHG------LD-----RAHAEATAHAPL-----PDG------YGRLGLTATTRILYQLT-------ADVV----TYADAV-------------------------------------------------------------------------------------------------------
83591793    Rhodospirillum rubrum ATCC 11170            ----------VFSD----LWAARQDRS------ALLALIGDPRGPTR---VT-----EDETAEA-VADAIQIVLPTG------RASLSAKAARAIAQAMA-------PGI----GYDEAV-------------------------------------------------------------------------------------------------------
288957741   Azospirillum sp. B510                        ----------LVEL----LLTE-AEPE------AIRAALTARWA------LD-----EAT-----AAKLAGATLPDF------HGRYGRRAVAELLPVLERETRGDPDGRVRPIRLDEAV-------------------------------------------------------------------------------------------------
427429481   Caenispirillum salinarum AK4                ----------YVRD----VVARGADSA------ALARLLAEGAHGVP-PVA------AAAVPAATAAILESDIMQPG------RYSVCPWAAEAAILDAWANA-----PTE----GFYDVTRGLFGFAPGEIVLEDLRRARGALLA--------------------------------------------------------------------------------
92109262    Nitrobacter hamburgensis X14                 ----------IVAR----LEET-EDEN------ELIAWLEKECA------L------DGAAAAR-VANT---TLPDG------HCRLGLRAIKKIVPIMQ-------DGL----DEDGVA-------------------------------------------------------------------------------------------------------
148255343   Bradyrhizobium sp. BTAi1                     ----------IVAL----LESA-LDEA------AIIASLGTTHS------LD-----EAAAQRA-LSAL----LPDG------YCRLGLRAIKRVLPLME-------AGR----TYAEAA-------------------------------------------------------------------------------------------------------
34557790    Wolinella succinogenes DSM 1740              ----------ATKI----LTYY-KDEG------QKRRELTKLP-------LE-----AEMVE-R-LVKI---GFSD------FLKLSLKAIRDILPAME-------SGA----RYDEAV-------------------------------------------------------------------------------------------------------
218563121   Campylobacter jejuni NCTC 11168              ----------IAKD----ITLI-KDEI------KKKALAKYD--------LN-----QNQID-S-LSKL---EFKD------HLNISFKALKLVTPLML-------EGK----KYDEAC--------------------------------------------------------------------------N------------------------
Jnet        218563121                                    ----------HHHH----HH-----HH------HHHHHHHH----------------HHHHH-H-HH-----------------HHHHHHHHHHHHHHH-------------------HHHHH-------------------------------------------------------------------------------------------------------
291276265   Helicobacter mustelae 12198                  ----------IANI----LGAN-KDWE------AILKELESLQ-------LS-----KEQIQTIKDAKL----NFSK------HINLSLEALYHLLPLMR-------EGK----RYDEGV-------------------------------------------------------------------------------------------------------
222109285   Acidovorax ebreus TPSY                        ----------IATV----LSVY-KDGA------EVVQQLRQLA-------LP-----EPAASIAVLEKI---SFDK------FSSLSLKALRRIVPLMQ-------SGL----RYDEAV-------------------------------------------------------------------------------------------------------
365156657   Bacillus smithii 7 3 47FAA                   ----------FGYA----LTIF-KDDE------DIVAYLQNEYITKNGKRVSNLA--NKVYDKS-LIDELLNLSFSK------FAHLSMKAIRNILPYME-------QGE----IYSKAC-------------------------------------------------------------------------------------------------------
220930482   Clostridium cellulolyticum H10               ----------LFYC----LTVY-KNDN------EIKDYLQANN--------------LDYLIEY-IAKL---PTFNK------FKHLSLVAMKRIIPFME-------KGY----KYSDAC-------------------------------------------------------------------------------------------------------
297182908   uncultured delta proteobact. HF0070 07E19    ----------AARL----ITLI-KDDE------KLSDQLADL--------LP---EASDKAITQ-LCEL----DFTT------AAKISLEAMYRILPHMN-------QGM----GFFDAC-------------------------------------------------------------------------------------------------------
154250555   Parvibaculum lavamentivorans DS-1            ----------VIIL------SE-KDRK------AHREAAANSF-------VA-----DFGITGEQAAQLQALKLPTG------WEPYSIPALNLFLAELE-------KGE----RFGALV-------------------------------------------------------------------------------------------------------
218767588   Neisseria meningitidis Z2491                 ----------IGTA----FSLF-KTDE------DITGRLKD-R-------IQ-----PEILEAL-LKHI---SFDK------FVQISLKALRRIVPLME-------QGK----RYDEAC-------------------------------------------------------------------------------------------------------
15602992    Pasteurella multocida str. Pm70              ----------IGTA----FSLY-KTDE------DIQQYLTN-K-------VP-----NSVINAL-LVSL---NFDK------FIELSLKSLRKILPLME-------QGK----RYDQAC-------------------------------------------------------------------------------------------------------
187736489   Akkermansia muciniphila ATCC BAA-835         --GKSVTPNYLLNL----LKSRGESGE------ALEKKIEKESKK-----KE-----ADYADTP-LKPK----YATG------RAPYARTVLKKVVEEIL-------DGE----DPTRPA-------------------------------------------------------------------------------------------------------
315605738   Actinomyces sp. oral taxon 180 str. F0310   -----------GCG----SEPDDVEDE------EVNELISSA----------------TAEDMLK-LELLAKK-LPSG------RVAYSLKTLREVTAAIL------ETGD----DLSQAI-------------------------------------------------------------------------------------------------------
117929158   Acidothermus cellulolyticus 11B              ----------LVAA----LADNSIAGE------EEQELLVH---------LP-----DAELE-A-LEGL---ALPSG------RVAYSRLTLSGLTRVMR-------DDGV----DVHNAR-------------------------------------------------------------------------------------------------------
189440764   Bifidobacterium longum DJO10A                ----------MIRL----LSNT-VDID------KVREDVAYASAIEFIDGLD-----DDA-----LTKLDSVDLPSG------RAAYSVETLQKLTRQML------TTDD----DLHEAR-------------------------------------------------------------------------------------------------------
403744858   Alicyclobacillus hesperidum URH17-3-68      ETLDQ-----IGWI----LSV---WKS---------DDNRRKKLSTL--GLS-----DNV-----IEELLPL----N------GSKFGHLSLKAIRKILP----FLEDGY----SYDVACE-------LAGYQFQ-----------------------------------------------------------------------------------
407803669   Alcanivorax sp. W11-5                        QLDTD-----DWSC----RFM---GKN---------GRPRNFSDEFVAFM------NELRMTDGFDRLSKMGFEGG------RSSYSIKALKALTEWMIAP--HWRETP----ETHRVDE--------EAAIREC-------------------------------------------------------------------------------------
423317190   Bergeyella zoohelcum ATCC 43767              NRQIE-----LWNI----LYNE-KGNE------YDLTSDRTSKVLEFINKYG-----NNIVDDNAEETAIRISKIKF------ARAYSSLSLKAVERILP----LVRAGK----YFNNDFSQQLQSKILKLLNENVEDPFAKAAQ---------------------------------------------------------------------------
402847315   Porphyromonas sp. oral taxon 279 str. F0450  KPLYR-----LWHI----LYS---IEE---------REAMRRALITQLGMKE-----EDL-DGGLLDQLYRLDFV---------KPGYGNKSAKFICKLLP----QLQQGL----GYSEACA--------AVGYRHS------------------------------------------------------------------------------------
404487228   Barnesiella intestinihominis YIT 11860       EPLYR-----LWHV----LYS---IDD---------REQLQSVLRQKFGIDD-----DEV-----LERLSAIDLV--------KAGFGNKSSKAIRRILP----FLQLGM----NYAEACE--------AAGYNHS------------------------------------------------------------------------------------
374384763   Odoribacter laneus YIT 12061                 -KREE-----IWHC----FYF---YDD--------NTLLFEKLQKDYALQT------NDL-----EKIKKIRL---------SESYGNVSLKAIRRINP----YLKKGY----AYSTAVLLGGIRN-SFGKRFEFKEYEPEIE-----------------------------------------------------------------------------
384109266   Treponema sp. JC4                            EEQDL-----IIET----IIT---ADE---------DDAVYEVIKKYD--LT-----QEQ----RDFIVKNTILQSG-------TSMLCKEVSEKLVKRLE-----EIADL----KYHEAVE--------SLGYKFA------------------------------------------------------------------------------------
402849997   Rhodovulum sp. PH10                          AEKDT-----FVGK----LLD---EAD---------EERLIRRLVT-ENRLS-----EDA-----ARRCASIPLADG------RYGLGRTANTEILAALVEETDETGTVV----TYAEAVR--------RAGERTG------------------------------------------------------------------------------------
Jnet        331001027                                    HHH----HHHH---------HHHHHHH------HHHHHH-----HHHHHHHHHH------------------HHHHHH---H----HHHHHHHHH------------------EEEEE--E---------------------------------------------------------------------------------------------------
331001027   Parasutterella excrementihominis YIT 11859  FVK----TALKEGKTEKELSKKFAAVI------KVLKMV-----SEVVPFIGKELRLS--------------DEAQSK--FNDLYSLAQLYNLIETER-------NGF----SKVSLAAHLENAWRMT---MTDG---------------------------------------------------------------------------------
34557932    Wolinella succinogenes DSM 1740              ELR---------KKDPAELSKEEKAKL------RLTDDVI----LNEWSQKIANFFDID--------------DKHRQR--FNNLFSMAQLHTVIDTPR-------SGF----SSTCKRCTAENRFRSETAFYNDETGE-------------------------------------------------------------------------------
54296138    Legionella pneumophila str. Paris            EAL-----NHPEH-------SNNKALI------KIIQTI------PDIIQAIQSHLGHN--------------DSQALI---YHNPFSLSQLYTILETKR-------DGF----HKNCVAVTCENYWRSQKTEIDPEISY-------------------------------------------------------------------------------
319941583   Sutterella wadsworthensis 3 1 45B            TAQYREVNKLPRN-------AQDKELL------TIRDRV------AETADFIAANLGLS--------------DEQKRK--FANPFSLAQFYTLIETEV-------SGF----SATTLAVHLENAWRMT---IKDAVIN---------------------------------------------------------------------------------
254447899   gamma proteobact. HTCC5015                   LQQ---LHKLEAGKK-----LDDKPLA------LLYKNS------GLIASKIGEALNIE--------------PDEVSR--FASPHSLAQIFNIIEGDV-------AGF----NKTCRACTYENIWRMQEEKVESLLTNQLLSE---------------------------------------------------------------------------
118497352   Francisella novicida U112                    IAR------NTKGKCEKEIFNLICKIE------GSEDKK------GNYKHGLAYELGVLLFGEPNEASKPE-FDRKIKK---FNSIYSFAQIQQIAFAER-------KGN----ANTCAVCSADNAHRMQQIKITEPVED-----------------------------------------------------------------------------

Motifs                                                   __________________________________________________________________________________________________________________________________________________________________________________________
informative positions                                     ---------------------------------------------------------------------------------------------------------------********************---------------------------------------------------------------------------------------------------------------------------------------------------------
```

227494853	*Actinomyces coleocanis* DSM 15436#	---VNEFGVSEDWRPPA---EPIGARVGNPAVDRVLKAVNRYLMAAEAE---WG--APL--SVN-IEHVREG
328956315	*Coriobacterium glomerans* PW2	---REGCPVS---GKRGRAMVMMEILRDEELGFQKKVDDFNRAFF---AENAQALG-VNELPGSPAVRRSLNQSIRIVDEIASI---AGK-APA--NIF-IEVTRDE
227824983	*Acidaminococcus* sp. D21	---MWNTNDNLMQLLSECYT--FSDEITKLQEAYY---AKAQLSLNDF-LDSMYISNAVKRPIYRTLAVVNDIRKA---CGT-APK--RIF-IEMARDG
303229466	*Veillonella atypica* ACS-134-V-Col7a	---ELMRNDSYNLMEILGDKFSFMECIEEENAKLA---QGQVVNPHDI-IDELALSPAVKRAVWQALRIVDEVAHI---KKA-LPS--RIF-VEVARTN
34762592	*Fusobacterium nucleatum* ATCC 49256	---EALRRTNYNLMELLSSKFTLQESIDNENKEMN---EVSYRDL-IEESYVSPSLKRAILQTLKIYEEIKKI---TGR-VPK--KVF-IEMARGG
374307738	*Filifactor alocis* ATCC 35896	---AMWETDNNLMQILSKKFTFMDNVEDFNSGKV---GKIDKITYDST-VKEMFLSPENKRAVWQTIQVAEEIKKV---MGC-EPK--KIF-IEMARGG
320528778	*Solobacterium moorei* F0204	---MWNTNATLMEILSNEKYQ-FKQNIENYKAENY---DEKQNLHEE-LDDMYISPAARRSIWQALRIVDEIVDI---KKS-APK--KIF-IEMAREK
291520705	*Coprococcus catus* GD-7	---LWQTNDNLMQLLSRNYG--FTNEVEEFNTLKK---ETDLSYKT-VDELYVSPAVKRQIWQTLKVVKEIQKV---MGN-APK--RVF-VEMAREK
42525843	*Treponema denticola* ATCC 35405	---NIITAMRETQNNLMELLSSEFTFTENIKKINSGFE---DAEKQFSYDGL-VKPLFLSPSVKKMLWQTLKLVKEISHI---TQA-PPK--KIF-IEMAKGA
304438954	*Peptoniphilus duerdenii* ATCC BAA-1640	---FMREYNLNLMELMSGHYT-FTEEVEKLNPVEN---RELCYEM-VDELYLSPSVKRMLWQSLRVVDEIKRI---IGK-DPK--KIF-IEMARAK
224543312	*Catenibacterium mitsuokai* DSM 15897	---VLEMSRLNLMEIINDKDLGYAQMIEEATSCPE---DGKFTYEE-VERLAGSPALKRGIWQSLQIVEEITKV---MKC-RPK--YIY-IEFERSE
116628213	*Streptococcus thermophilus* LMD-9	---GISNRNFMQLIHDD-----ALSFKKKIQKAQI---IGDEDKGNIKEV-VKSLPGSPAIKKGILQSIKIVDELVKV---MGGRKPE--SIV-VEMAREN
24379809	*Streptococcus mutans* UA159	---GNSNRNFMQLINDD-----ALSFKEEIAKAQV---IGETD--NLNQV-VSDIAGSPAIKKGILQSLKIVDELVKI---MG-HQPE--NIV-VEMAREN
13622193	*Streptococcus pyogenes* SF370	---GFANRNFMQLIHDD-----SLTFKEDIQKAQV---SGQGD--SLHEH-IANLAGSPAIKKGILQTVKVVDELVKV---MGRHKPE--NIV-IEMAREN
310286728	*Bifidobacterium bifidum* S17	---IMRAEDRNLMEIITDKQLGFSDWIEQENLGAE---NGSSLMEV-VDDLRVSPKVKRGIIQSIRLIDDISKA---VGK-RPS--RIF-LELADDI
366983953	*Oenococcus kitaharae* DSM 17330	---TLYNTKMNLMEIINNAEDDFGVRAWIDKQNTT---DGDEQDVYSL-IDELAGPKEIKRGIVQSFRILDDITKA---VGY-APK--RVY-LEFARKT
422884106	*Streptococcus sanguinis* SK49	---FLRNDEENRNLTKLISDNTLSFEPKIKDI-QSKST---IEDDIFDE-IKKLAGSPAIKRGILNSIKIVDELVQI---IGY-PPH--NIV-IEMAREN
339625081	*Fructobacillus fructosus* KCTC 3544	---KLKSTSQNFMSIINNDKYGVQAWITEQ---NTG---SSKLTFDEKVNELTTSPANKRGIKQSFAVLNDIKKA---MKE-EPR--RVY-LEFARED
306821691	*Eubacterium yurii* ATCC 43715	---SLWETNFNLMELLSSRFTYMDELEKRVKKLEKP---LSEWTIED-LDDMYLSSPVKRMIWQSMKIVDEIQTV---IGY-APK--RIF-VEMTRSE
336394882	*Lactobacillus farciminis* KCTC 3681	---LMWATNNNFISIMSNDKYDFKNYIENH-NLNKN---EDQNISDL-VNDIHVSPALKRGITQSIKIVQEIVKF---MGH-APK--HIF-IEVTRET
323463801	*Staphylococcus pseudintermedius* ED99	---LLRHSDENFMEILTNDVYGFQNFIKEE--NQVQ---SNKIQHQD-IANLTTSPALKKGIWSTIKLVRELTSI---FGE--PE--KII-MEFATED
389815359	*Planococcus antarcticus* DSM 14505	---HMEQYSSVFMEVLKNKGFGLEKKIQKMNQHQVDG---TKKIRYED-IEELAGSPALKRGIWRSVKIVEELVSI---FGE--PA--NIV-LEVARED
258509199	*Lactobacillus rhamnosus* GG	---ELMLSNHNLMQILADET--LKETMTELNQDKL---KTDDIEDVINDAYTSPSNKKALRQVLRVVEDIKHA---ANGQDPS--WLF-IETADGT
169823755	*Finegoldia magna* ATCC 29328	---FLRNSSDNLMQIIGSQNYSFNEYIDKLRKKYI---PQEISYEVVENLYVSPSVKKMIWQVIRVTEEITKV---MGY-DPD--KIF-IEMAKSE
Jnet	227501312	---H------HHHHHHHH-----HHHHHHHHHH---HHHHHHHHHHHHHHHHHHH---H-------EEE-EEEEE--
47458868	*Mycoplasma mobile* 163K	---SYEEKALQNQKN-------NFSHSNLFEKTWV---EDLIASPTVKRSLRQTMNLLKEIFKY---SEKNNLEIE--KIV-VEVTRSS
284931710	*Mycoplasma gallisepticum* str. F	---QKNNDKGWNFEAIKNFDQKFIDITKKNNNLSL---KQNKRYLDDRFINDAILSPGVKRILREATKVFNAILKQ---FSE-EYDVTKVV-IELAREL
71894592	*Mycoplasma synoviae* 53	---NYESLKHSNEELKKRTEKAELKAQQN---QKYLKDNFLKEALVPLSVKTSVLQAIKIFNQIIKN---FGK-KYEISQVV-IEMAREL
363542550	*Mycoplasma ovipneumoniae* SC01	---NSEYLKWKDEEIRRKWEEQKSKLGKTDKKT---KYLNPRIFQDEIISPGTKNTFEQAVLVLNQIIKK---YSK-ENIIDAII-IESPREK
384393286	*Mycoplasma canis* PG 14	---NLEAIKNYDEEIKSQIEDNSSLMAKQDK---KYLNDNFLKDAILPPNVKVTFQQAILIFNKIIQK---FSK-DFEIDKVV-IELAREM
238924075	*Eubacterium rectale* ATCC 33656	---EMGVTKGTQEEFAG------LKYIPV---DVVSEDIFNPVVRRSVRISFKILNAVLKK---YK--ALD--TIV-IEMPRDR
315149830	*Enterococcus faecalis* TX0012	---LEQFQLKSDVRKRYSEYKKLPT---KDVLAEIYNPTVNKTVSQAFKVIDALLVK---YGKEQIR--YIT-IEMPRDD
116627542	*Streptococcus thermophilus* LMD-9	---RLGKQK------TTSSSNK---TKYIDEKLLTEEIYNPVVAKSVRQAIKIVNAAIKE---YG--DFD--NIV-IEMARET
315659848	*Staphylococcus lugdunensis* M23590	---QMEIFTHLNIKPKKI----NLTAANKIPKAMI---DEFILSPVVKRTFGQAINLINKIIEK---YG--VPE--DII-IELAREN
160915782	*Eubacterium dolichum* DSM 3991	---LFGLKQNNELSVKG---MKNIQADDTAILSPVAKRAQRETFKVVNRLREI---YG--EFD--SIV-VEMAREK
325677756	*Ruminococcus albus* 8	---AKFNKEIPDIDENAKPQ--KLPPFKN---EDDCEFFKNPVVFRSINETRKLINAIIDK---YG--YPA--AVN-IETADEL
225377804	*Roseburia inulinivorans* DSM 16841	---EAAGYDFRALNDGNKKH--LLKGEEINAIV---NDITNPVVKRSVSQTIKVINAIIQK---YG--SPQ--AVN-IELAREM
336393381	*Lactobacillus coryniformis* KCTC 3535	---TNTGYDFRKKQISK---DTIREEITNPVVRRAVTKTIKIVEQIIRR---YG--KPD--GIN-IELAREL
310780384	*Ilyobacter polytropus* DSM 2926	---EKADYDYKNNGIKFKRGE-LLPVVDK---DLIANPVVLRAISQTRKVVNAIIRK---YG--TPH--TIH-VEVARDL
301311869	*Bacteroides* sp. 20 3	GEYDENIR---ERVRQKAINLTDIT-----HFRALPLWLACYL----VYDRHSEVKDIVKWKTPK------DIDLYLKSFKQHSLRNPIVEQVITETLRTVRDIWQQ---VG--HID--EIH-IELGREM
385811609	*Ignavibacterium album* JCM 16511	---DQDLEKQVLKSFLEKKNESD----YLKGLKTYQAGYL----IYGKHSE-KDVPIVNSPDELGEYIR-----KKLPNNSLRNPIVEQVIRETIFIVRDVWKS---FG--IID--EIH-IELGREL
60683389	*Bacteroides fragilis* NCTC 9343	---VYAGYRHSESSLT------REEIANKVLKDRL---MLLPKNSLHNPVVEKILNQMVNVINVIIDI---YG--KPD--EIR-VELAREL
319957206	*Nitratifractor salsuginis* DSM 16511	---GFDDRE------------DYSRYPKSLRHLH---LREGNLFEKEENPINNHAVKSLASWALGLIADLSWR---YG--PFD--EII-LETTRDA
187250660	*Elusimicrobium minutum* Pei191	---EEALKEGKLTKEKQAIKD-RLPYYGAVLQEST---------QKIIAKGFSPQFKDKGYKTPHTNKYELEYGRIANPVVHQTLNELRKLVNEIIDI---LGK-KPC--EIG-LETAREL
325972003	*Sphaerochaeta globus* str. Buddy	---ERGMETGEFQELSVWEQQSLLPYYGQILTGST------QALMGKYWHSAFKEKRDSEGFFKPNTNSDEEKYGRIANPVVHQTLNELRKLMNELITI---LGA-KPQ--EIT-VELAREL
296446027	*Methylosinus trichosporium* OB3b	---CAADYDHTASRERG-----AFDVGGHGREALK---RILQEERISRELVGSPTARKALIESIKQVKAIVER---YG--VPD--RIH-VELARDV
347536497	*Flavobacterium branchiophilum* FL-15	KKKLVLFYKSN--EIENKEQQETIFNELLPIFIQQLKDYE--FIKIQRLDQKVLIFLKGKNETGQIFCTEEKGTAEEKEKKI---KNRLKKLYHPSDIEKFKKKIIKDEFGNEKIVLGSPLTPSIKNPMAMRALHQLRKVLNALILE---GQID--EKT--IIH-IEMAREL
345885718	*Prevotella* sp. C561	IRQIENSLGARRWSLMDANEQTDILQKVRDRYQDFFRSHERKFVESPKLGESFENYLTKKFPMVE---REQWKKLYHPSQITIYRPVSVGKDRS--VLRLGNPDIGAIKNPTVLRVLNTLRRRVNQLLDD---GVISP-DET--RVV-VETAREL
282880052	*Prevotella timonensis* CRIS 5C-B1	---GTIEMLIKDFLANNFELP---AGATDKLYHPSMIETY-PNAQRNEFG--ILQLGSPRTNAIRNPMAMRSLHILRRVVNQLLKE---SIID--ENT--EVH-VEYAREL
312879015	*Aminomonas paucivorans* DSM 12260	---ADAGYAAPPPDRHP-----KLPPLEE---ADWRNPVVFRALTQTRKVVNALVRR---YG--PPW--CIH-LELAREL
294086111	*Candidatus Puniceispirillum marinum* IMCC1322	---KEAGLGEANLYDPYAALSDKLDYYGKALAGHV---MGASGKFEDSDEKRYGTISNPTVHIALNQVRAVVNELIRL---HG--KPD--EVV-IEIGRDL
330822845	*Alicycliphilus denitrificans* K601	-----QAAGFDHHSQLGFEYDAS---EVEDL---VHPETGEIRSV--FKQLPYYGKALQRHV---AFGSGKPEDPDEKRYGKIANPTVHIGLNQVRMVVNALIRR---YG--RPT--EVV-IELARDL
344171927	*Ralstonia syzygii* R24	---AAGYPAHTADLENGERD--ALPYYGELLWRYT---QDAPTAKNDAERKFGKIANPTVHIGLNQLRKLVNALIQR---YG--KPA--QIV-VELARNL
159042956	*Dinoroseobacter shibae* DFL 12	---KACGWHHSDGRTGECFD--RLPYYGEVLERHV---IPGSYHPDDDDITRFGRITNPTVHIGLNQLRRLVNRIIET---HG--KPH--QIV-VELARDL
83591793	*Rhodospirillum rubrum* ATCC 11170	---TLALGLHHSHRPRQERLA-RLPYYAAALPDVG---LDGDPVGPPPAEDDGAAAEAYYGRIGNISVHIALNETRKIVNALLHR---HG--PIL--RLVMVETTREL
288957741	*Azospirillum* sp. B510	---KLLRGGKDHSDFSREGALLDALPYYGAVLERHV---AFGTGNPADPEEKRVGRVANPTVHIALNQLRHLVNAILAR---HG--RPE--EIV-IELARDL
427429481	*Caenispirillum salinarum* AK4	---HLPRTMAAARTPNRAAQQRGPLPAYESVIPSQL-----------ITSLRRAHKGRAADWSAADPEERNPFLRTWTGNAATDHILNQVRKTANEVITK---YGNRRGWDPLPS--RIT-VELAREA
92109262	*Nitrobacter hamburgensis* X14	---GAGYHI---AAKRAGYDHAKLPTGEQLGRLPYYGQWLQDAV---VGSGDARDQKEKQYGQFPNPTVHIGLGQLRKVVNDLIDK---YG--PPT--EIS-IEFTRAL
148255343	*Bradyrhizobium* sp. BTAi1	---SAAGYDHALLPGGKLSPTGYLPYYGQWLQNDV---VGSDDERDTNERRWGRLPNPTVHIGIGQLRRVVNELIRW---HG--PPA--EIT-VELTRDL
34557790	*Wolinella succinogenes* DSM 1740	---LMLGVPHKEKSAILPPLNK---TDIDILNPTVIRAFAQFRKVANALVRK---YG--AFD--RVH-FELAREI
218563121	*Campylobacter jejuni* NCTC 11168	---ELNLKVAINEDKKD-----FLPAFNE---TYYKDEVTNPVVLRAIKEYRKVLNALLKK---YG--KVH--KIN-IELAREV
Jnet	218563121	---HHHHHHHHHHHH---HHHHHHHHHHHHHHHHHHH---H-------EEE-EEEEE--
291276265	*Helicobacter mustelae* 12198	---EILQERGIFSKPQPKNRQ-LLPPLSELAK---EESYFDIPNPVLRRALSEFRKVVNALLEK---YG--GFH--YFH-IELTRDV
222109285	*Acidovorax ebreus* TPSY	---AQIPEYGHHSQRIEPGAAKHLYLPPFYEAQRKYA---GKGDHIGSMQFRDDADIPRNPVVLRALNQARKVVNALIRE---YG--SPI--AVN-IEMARDL
365156657	*Bacillus smithii* 7 3 47FAA	---ELAGYNFTGPKKKEKAL--LLPVIPNI---ANPVVMRALTQSRKVVNAIIKK---YG--SPV--SIH-IELARDL
220930482	*Clostridium cellulolyticum* H10	---NMAELDFTGSS--------KLEKCNKLTV---EPIIENVTNPVVIRALTQARKVINAIIQK---YG--LPY--MVN-IELAREA
297182908	uncultured delta proteobact. HF0070 07E19	---QQESLPEIGVP--------PAGDRVPP---FDEMYNPVVNRVLSQSRKLINAVIDE---YG--MPA--KIR-VELARDL
154250555	*Parvibaculum lavamentivorans* DS-1	---NGPDWEGWRRTNFPHRNQPTGEILDKLPSPASKEE---RERISQLRNPTVVRTQNELRKVVNNLIGL---YG--KPD--RIR-IEVGRDV
218767588	*Neisseria meningitidis* Z2491	---AEIYGDHY-GKKNTEEKI-YLPPIPA---DEIRNPVVLRALSQARKVINGVVRR---YG--SPA--RIH-IETAREV
15602992	*Pasteurella multocida* str. Pm70	---REIYGHHY-GEANQKTSQ-LLPAIPA---QEIRNPVVLRTLSQARKVINAIIRQ---YG--SPA--RVH-IETGREL
187736489	*Akkermansia muciniphila* ATCC BAA-835	---RGEAHPDGELKAHDGCLYCLLDTDSSVNQHQK---ERRLDTMTNNHLVRHRMLILDRLLKDLIQD---FADGQKDRISRVCVEVGKEL
315605738	*Actinomyces* sp. oral taxon 180 str. F0310	---TRLYGVDPGWVPTP---APIEAPVGNPSVDRVLKQVARWLKFASKR---WG--VPQ--TVN-IEHTREG
117929158	*Acidothermus cellulolyticus* 11B	---KTCFGVDDNWRPPL---PALHEATGHPVVDRNLAILRKFLSSATMR---WG--PPQ--SIV-VELARGA
189440764	*Bifidobacterium longum* DJO10A	---KTLFNVTDSWRPPA---DPIGEPLGNPSVDRVLKNVNRYLMNCQQR---WG--NPV--SVN-IEHVRSS
403744858	*Alicyclobacillus hesperidum* URH17-3-68	---GKTEYVKQRLLP---PLGEGE------VTNPVVRRALSQAIKVVNAVIRK---HG---SPE-SIH-IELAREL
407803669	*Alcanivorax* sp. W11-5	---YP---ESLATPAQGGRQSKLE---PPPL--------TGNEVVDVALRQVRHTINMMIDD---LG--SVPA-QIV-VEMAREM
423317190	*Bergeyella zoohelcum* ATCC 43767	---TYLDN---NQSVLSEGGVGNSIAT--ILVYDKHTAKE---YSHDELYKSYKEINLLKQGDLRNPLVEQIINEALVLIRDIWKN---YG--IKPN-EIR-VELARDL
402847315	*Porphyromonas* sp. oral taxon 279 str. F0450	---NS---PTSEEITERTLLEKIP---LLQRNE------LRQPLVEKILNQMINLVNALKAE---YG----ID-EVR-VELAREL
404487228	*Barnesiella intestinihominis* YIT 11860	---NN---YTKAENEARALLDRLP---AIKKNE------LRQPVVEKILNQMVNVVNALMEK---YG---RFD-EIR-VELAREL
374384763	*Odoribacter laneus* YIT 12061	---KAVCR---ILKEKNAEGEVIRKIKDYLVHNRFGFAKNDRAFQKLY---HHSQAITTQAQKERLPETGNLRNPIVQQGLNELRRTVNKLLATCREK---YGPSFKFD-HIH-VEMGREL
384109266	*Treponema* sp. JC4	---DQTVEKYDLLP-----YYGKVLPG---STMEIDLSAPETNPEKHYGKISNPTVHVALNQTRVVVNALIKE---YG---KPS-QIA-IELSRDL
402849997	*Rhodovulum* sp. PH10	---RN---WHHSDERDGVILDRLP-----YYGEILQR---HVVPGSGEPEEKNEAARWGRLANPTVHIGLNQLRKVVNRLIAA---HG---RPD-QIV-VELAREL
Jnet	331001027	---EE---------HHHHHHHHHHHHHHHHHHHHHHHHHHH---EEE--EEE-EE----
331001027	*Parasutterella excrementihominis* YIT 11859	---SAQCCRLPADCVRPFDGFIRKAIDRNSWEVAKRIAEEVKKSVDFT---NG-TVKI--PVA-IEANSFN
34557932	*Wolinella succinogenes* DSM 1740	---FHKKATATCQRLPADTQRPFSGKIERYIDKLGYELAKIKAKELEGMEA---K-EIKV--PII-LEQNAFE
54296138	*Legionella pneumophila* str. Paris	---ASRLPADSVRPFDGVLARMMQRLAYEIAMAKWEQIKHIPD---NS-SLLI--PIY-LEQNRFE
319941583	*Sutterella wadsworthensis* 3 1 45B	---GETVRAAQCSRLPAETARPFDGLVRRLVDRQAWEIAKRVSTDIQSKVDFS---NG-IVDV--SIF-VEENKFE
254447899	gamma proteobact. HTCC5015	---IHGERKVPLKSAMCTRLSADSTRPFDGQMASIIEHIARKIAQHKIAQINDVPK---EF-SIDI--PII-IESNQFS
118497352	*Francisella novicida* U112	---NKDKIILSAKAQRLPAIPTRIVDGAVKKMATILAKNIVDDNWQNIKQVLS-A---KH-QLHI--PII-TESNAFE
Motifs		RuvC_II E
informative positions		---**********************---***********

227494853	*Actinomyces coleocanis* DSM 15436#	F-------ISKRQAVEIDRENQKRY--QRNQAVRSQIADHINATS-------------------------GVRGSDV---TRY-LAIQR------QNGE----------CLY-----------------------CGTAITFVN---------SEMDHIVPRA
328956315	*Coriobacterium glomerans* PW2	DPK-KKGRRTKRRYNDLKDALEAFK--KEDPELWRELC------------------------------ETAPNDM--DERL-SLYFM------QRGK----------CLY-----------------------SGRAIDIHQLSNAGI---YEVDHIIPRT
227824983	*Acidaminococcus* sp. D21	E---SKKKRSVTRREQIKNLYRSIR--KDFQQEVDFLEKILENKSDG----------------------QLQSDAL---YLYFA------QLGR----------DMY-----------------------TGDPIKLEHIKDQSF---YNIDHIYPQS
303229466	*Veillonella atypica* ACS-134-V-Col7a	K---SEKKKKDSRQKRLSDLYSAI---KKDDVLQSGLQDKEFGALKSG----------------L-----ANYDDAALR---SKKL-YLYYT------QMGR----------CAY-----------------------TGNIIDLNQLNTDN----YDIDHIYPRS
34762592	*Fusobacterium nucleatum* ATCC 49256	DES-MKNKKIPARQEQLKKLYDSCG--NDIANFSIDIKEMKNSLS-------------------------SYDNNSLRQ---KKL-YLYYL------QFGK----------CMY-----------------------TGREIDLDRLLQNNDT--YDIDHIYPRS
374307738	*Filifactor alocis* ATCC 35896	E---KVKKRTKSRKAQLLELYAACE--EDCRELIKEIE--------------------------------DRDERDFNS---MKL-FLYYT------QFGK----------CMY-----------------------SGDDIDINELIRGNSK--WDRDHIYPQS
320528778	*Solobacterium moorei* F0204	KSA-MKKKRTESRKDTLLELYKSCK--SQADGFYDEELFEKLSNESNS----------------------RLRRDQL---YLYYT------QMGR----------SMY-----------------------TGKRIDFDKLINDKNT--YDIDHIYPRS
291520705	*Coprococcus catus* GD-7	Q----EGKRSDSRKKQLVELYRACK--NEERDWITELN--------------------------------AQSDQQLRS---DKL-FLYYI------QKGR----------CMY-----------------------SGETIQLDELWDNTK---YDIDHIYPQS
42525843	*Treponema denticola* ATCC 35405	E---LEPARTKTRLKILQDLYNNCK--NDADAFSSEIKDLSGKIE-------------------------NEDNLRLRS---DKL-YLYYT------QLGK----------CMY-----------------------CGKPIEIGHVFDTSN---YDIDHIYPQS
304438954	*Peptoniphilus duerdenii* ATCC BAA-1640	E---AKNSRKESRKNKLLEFYKFGKKAFINEIGEERYNYLLNEIN-------------------------SEEESKFRW---DNL-YLYYT------QLGR----------CMY-----------------------SLEPIDLADLKSNNI---YDQDHIYPKS
224543312	*Catenibacterium mitsuokai* DSM 15897	E----AKERTESKIKKLENVYKDLD-EQTKKEYKSVLEELKGFDNTK-----------------------KISSDSL---FLYFT------QLGK----------CMY-----------------------SGKKLDIDSLDK------YQIDHIVPQS
116628213	*Streptococcus thermophilus* LMD-9	Q-------YT-------NQGKSNSQ--QRLKRLEKSLKELGSKILKENIPAKLS-----------------KIDNNALQN---DRL-YLYYL------QNGK----------DMY-----------------------TGDDLDIDRLSN------YDIDHIIPQA
24379809	*Streptococcus mutans* UA159	Q-------FT-------NQGRRNSQ--QRLKGLTDSIKEFGSQILKEH-----------------------PVENSQLQN---DRL-FLYYL------QNGR----------DMY-----------------------TGEELDIDYLSQ------YDIDHIIPQA
13622193	*Streptococcus pyogenes* SF370	Q-------TT-------QKGQKNSR--ERMKRIEEGIKELGSQILKEH-----------------------PVENTQLQN---EKL-YLYYL------QNGR----------DMY-----------------------VDQELDINRLSD------YDVDHIVPQS
310286728	*Bifidobacterium bifidum* S17	Q----PSGRTISRKSRLQDLYRNANLGKEFKGIADELN--------------------------------ACSDKDLQD---DRL-FLYYT------QLGK----------DMY-----------------------TGEELDLDRLSSA-----YDIDHIIPQA
366983953	*Oenococcus kitaharae* DSM 17330	Q----ESHLTNSRKNQLSTLLKNA--GLSELVTQVSQY---------------------------------DAAALQN---DRL-YLYFL------QQGK----------DMY-----------------------SGEKLNLDNLSN------YDIDHIIPQA
422884106	*Streptococcus sanguinis* SK49	MTTEEGQKKAKTRKTKLESAL--KNIEN--SLLENGKV--------------------------------PHSDEQLQS---EKL-YLYYL------QNGK----------DMY-----------------TLDKTGSPAPLYLDQLDQ------YEVDHIIPYS
339625081	*Fructobacillus fructosus* KCTC 3544	Q----TSVRSVPRYNQLKEKYQSKSLSEEAKVLKKTLD--------------------------------G-NKNKMSD---DRY-FLYFQ------QQGK----------DMY-----------------------TGRPINFERLSQD-----YDIDHIIPQA
306821691	*Eubacterium yurii* ATCC 43715	G----EKVRTKSRKDRLKELY--NGIKEDSKQWVKELD--------------------------------SKDESYFRS---KKM-YLYYL------QKGR----------CMY-----------------------SGEVIELDKLMDDN---LYDIDHIYPRS
336394882	*Lactobacillus farciminis* KCTC 3681	K----KSEITTSREKRIKRLQS-KLLNKANDFKPQLRE-YLVPNKKIQE---------------------ELKKHKNDLSS---ERI-MLYFL------QNGK----------SLY-----------------------SEESLNINKLSD------YQVDHILPRT
323463801	*Staphylococcus pseudintermedius* ED99	Q---QKGKKQKSRKQLWDDNIKKNKLKSVDEYKYIIDV-------------------------------ANKLNNEQLQQ---EKL-WLYLS------QNGK----------CMY-----------------------SGQSIDLDALLSPNATKHYEVDHIFPRS
389815359	*Planococcus antarcticus* DSM 14505	G----EKKRTKSRKDQWEELTK-TTLKNDPDLKSFIGE--------------------------------IKSQGDQRFNE---QRF-WLYVT------QQGK----------CLY-----------------------TGKALDIQNLSM------YEVDHILPQN
258509199	*Lactobacillus rhamnosus* GG	G---TAGKRTQSRQKQIQTVYANAAQELIDSAVRGELEDKI-----------------------------ADKASFT---DRL-VLYFM------QGGR----------DIY-----------------------TGAPLNIDQLSH------YDIDHILPQS
169823755	*Finegoldia magna* ATCC 29328	E----EKKTTISRKNKLLDLYKAIKKDERDSQYEKLLTGLN-----------------------------KLDDSDLRS---RKL-YLYYT------QMGR----------DMY-----------------------TGEKIDLDKLFDSTH---YDKDHIIPQS
Jnet	227501312	-------HHHHHHHHHHHHHHHHHHHHHHHH---HHHH--------------------------------------HHHH---HHH-HHHHH------H---EEEEEE---
47458868	*Mycoplasma mobile* 163K	N--------NKHERKKIEGINKYRK--EKYEELKKVYDLP------------------------------NENTTLL---KKL-WLLRQ------QQGY----------DAY-----------------------SLRKIEANDVINKPWN--YDIDHIVPRS
284931710	*Mycoplasma gallisepticum* str. F	S--------EEKELENTKNYKKLIK--KNGDKISEGLKALGISEDEIK----------------------DILKSPTKS---YKF-LLWLQ------QDHI----------DPY-----------------------SLKEIAFDDIFTKTEK--FEIDHIIPYS
71894592	*Mycoplasma synoviae* 53	TKP-NLEKLLNNATNSNIKILKEKL--DQTEKFDDFTKKKFID---------------------------KIENSVVFR---NKL-FLWFE------QDRK----------DPY-----------------------TQLDIKINEIEDE-----TEIDHVIPYS
363542550	*Mycoplasma ovipneumoniae* SC01	NDK-KTIEEIKK---RNKKGKGKTL--EKLFQILNLENKGYKLS--------------------------DLETKPAKLL---DRL-RFYHQ------QDGI----------DLY-----------------------TLDKINIDQLINGSQK--YEIEHIIPYS
384393286	*Mycoplasma canis* PG 14	TQD-QENDALKG-IAKAQKSKKSLV--EERLEANNIDKSVFN----------------------------DKYEKLI---YKI-FLWIS------QDFK----------DPY-----------------------TGAQISVNEIVN--NK--VEIDHIIPYS
238924075	*Eubacterium rectale* ATCC 33656	N--------SEEQKKRINDSQKLNE--KEMEYIEKKLAVTYGIKLSPS----------------------DFSSQKQLS---LKL-KLWNE------QDGI----------CLY-----------------------SGKTIDPNDIINNPQL--FEIDHIIPRS
315149830	*Enterococcus faecalis* TX0012	N--------EEDEKKRIKELHAKNS--QRKNDSQSYFMQKSGWSQEKF----------------QTTI--QKNRRFL---AKL-LYYYE------QDGI----------CAY-----------------------TGLPISPELLVSDS---TEIDHIIPIS
116627542	*Streptococcus thermophilus* LMD-9	N--------EDDEKKAIQKIQKANK--DEKDAAMLKAANQYNGKAELPHSVF------------------HGHKQLA---TKI-RLWHQ------QGER----------CLY-----------------------TGKTISIHDLINNSNQ--FEVDHILPLS
315659848	*Staphylococcus lugdunensis* M23590	N--------SKDKQKFINEMQKKNE--NTRKRINEIIGKYGN----------------------------QNAKRLV---EKI-RLHDE------QEGK----------CLY-----------------------SLESIPLEDLLNNPNH--YEVDHIIPRS
160915782	*Eubacterium dolichum* DSM 3991	N--------SEEQRKAIRERQKFFE--MRNKQVADIIGDDR-----------------------------KINAKLR---EKL-VLYQE------QDGK----------TAY-----------------------SLEPIDLKLLIDDPNA--YEVDHIIPIS
325677756	*Ruminococcus albus* 8	N--------KTFEDRAIDTKRNNDNQ--KENDRIVKEIIECIKCDE------------------------VHARHLI---EKY-KLWEA------QEGK----------CLY-----------------------SGETITKEDMLRDKDKL-FEVDHIVPYS
225377804	*Roseburia inulinivorans* DSM 16841	S--------KNFQDRTNLEKEMKKRQ--QENERAKQQIIELGKQ--------------------------NPTGQDI---LKY-RLWND------QGGY----------CLY-----------------------SGKKIPLEELFDGG---YDIDHILPYS
336393381	*Lactobacillus coryniformis* KCTC 3535	G--------RNFKERGDIQKRQDKNR--QTNDKIAAELTELGI---------------------------PVNGQNI---IRY-KLHKE------QNGV----------DPY-----------------------TGDQIPFERAFSEG---YEVDHIIPYS
310780384	*Ilyobacter polytropus* DSM 2926	A--------KSYDDRQTIIKENKKRE--LENEKTKKFISEEFGIK-------------------------NVKGKLL---LKY-RLYQE------QEGR----------CAY-----------------------SRKELSLSEVILDESM--TDIDHIIPYS
301311869	*Bacteroides* sp. 20 3	K--------NPADKRARMSQQMIKNE--NTNLRIKALLTEFLNPEFGIENVRPY-SPSQQDLLRIYEEGVLNSILELPEDIGIILGKFNQTDTLKRPTRSEI----------LRY-KLWLE------QKYR----------SPY-----------------------TGEMIPLSKLFTPA---YEIEHIIPQS
385811609	*Ignavibacterium album* JCM 16511	K--------NNSEERKKTSESQEKNF--QEKERARKLLKELLNS----SNFEHY-DENGN---KIFSSFTVNPNPDSPLDIEKFRIWKNQSGLTDEELNKKLKDEKIPTEIEV-----KKY-ILWLT------QKCR----------SPY-----------------------TGKIIPLSKLFDSNV---YEIEHIIPRS
60683389	*Bacteroides fragilis* NCTC 9343	K--------KNAKEREELTKSIAQTT--KAHEEYKTLLQTEFGLT-------------------------NVSRTDI---LRY-KLYKE---LESCGYK----------TLY-----------------------SNTYISREKLFSKE---FDIEHIIPQA
319957206	*Nitratifractor salsuginis* DSM 16511	L--------PEKIRKEIDKAMRERE--KALDKIIGKYKKEF-----------------------------PSIDKRL---ARKI-QLWER------QKGL----------DLY-----------------------SGKVINLSQLLDGS---ADIEHIVPQS
187250660	*Elusimicrobium minutum* Pei191	K--------KSAEDRSKLSREQNDNE--SNRNRIYEIYIRPQQQVIITR----------------R-----ENPRNYI---LKF-ELLEE------QKSQ----------CPF-----------------------CGGQISPNDIINNQ---ADIEHLFPIA
325972003	*Sphaerochaeta globus* str. Buddy	K--------VGAEKREDIIKQQTKQE--KEAVLAYSKYCEPN----------------------------NLDKRYI---ERF-RLLED------QAFV----------CPY-----------------------CLEHISVADIAAGR---ADVDHIFPRD
296446027	*Methylosinus trichosporium* OB3b	G--------KSIEEREEITRGIEKRN--RQKDKLRGLFEKEVGRPPQDG---------------------ARGKEEL---LRF-ELWSE------QMGR----------CLY-----------------------TDDYISPSQLVATDDA--VQVDHILPWS
347536497	*Flavobacterium branchiophilum* FL-15	N--------DANKRKGIQDYQNDNK--KFREDAIKEIKKLYFEDCKKEVEP--------------------TEDDI---LRY-QLWME------QNRS----------EIY----------------------EEGKNISICDIIGSNPA--YDIEHTIPRS
345885718	*Prevotella* sp. C561	N--------DANRKWALDTYNRIRH--DENEKIKKILEEFYPKRDGISTDDIDKARYVIDQREVDYFTGSKTYNKDI---------------------------------------KKY-KFWLE------QGGQ----------CMY-----------------------TGRTINLSNLFDPN-A--FDIEHTIPES
282880052	*Prevotella timonensis* CRIS 5C-B1	N--------DANKRRAIADRQKEQD--KQHKKYGDEIRKLYKEETGKDIEP--------------------TQTDV---LKF-QLWEE------QNHH----------CLY-----------------------TGEQIGITDFIGSNPK--FDIEHTIPQS
312879015	*Aminomonas paucivorans* DSM 12260	S--------QPAKVRRRIETEQQANE--KKKQQAEREFLDIVGT--------------------------APGPGDL---LKM-RLWRE------QGGF----------CPY-----------------------CEEYLNPTRLAEPGY--AEMDHILPYS
294086111	*Candidatus Puniceispirillum marinum* IMCC1322	P--------MGADGKRELERFQKEGR--AKNERARDELKKLGH---------------------------IDSRESR---QKF-QLWEQLAKEP-VDRC----------CPF-----------------------TGKMMSISDLFSDK---VEIEHLLPFS
330822845	*Alicycliphilus denitrificans* K601	K--------QSREQKVEAQRRQADNQ--RRNARIRRSIAEVLGIGEE-----------------------RVRGSDI---QKW-ICWEELSFDA-ADRR----------CPY-----------------------SGVQISAAMLLSDE---VEVEHILPFS
344171927	*Ralstonia syzygii* R24	K--------AGLEEKERIKKQQTANL--ERNERIRQKLQDAGV---------------------------PDNRENR---LRM-RLFEELGQ---GNGLGTP--------CIY-----------------------SGRQISLQRLFSND---VQVDHILPFS
159042956	*Dinoroseobacter shibae* DFL 12	K--------KSEEQKRADIKRIRDTT--EAAKKRSEKLEELEI---------------------------EDNGRNR---MLL-RLWEDLNPDDAMRRF-----------CPY-----------------------TGTRISAAMIFDGS---CDVDHILPYS
83591793	*Rhodospirillum rubrum* ATCC 11170	K--------AGADERKRMIAEQAERE--RENAEIDVELRKSDRWM-------------------------ANARERR---QRV-RLARR------QNNL----------CPY-----------------------TSTPIGHADLLGDA---YDIDHVIPLA
288957741	*Azospirillum* sp. B510	K--------RSAEDRRREDKRQADNQ--KRNEERKRLILSLGE---------------------------RPTPRNL---LKL-RLWEEQGPV--ENRR----------CPY-----------------------SGETISMRMLLSEQ---VDIDHILPFS
427429481	*Caenispirillum salinarum* AK4	K-----HGVIRRNEIAKENRENEGR--RKKESAALDTFCQDNTVSWQAGG--------------------LPKERAA---LRL-RLAQRQEFF--------------CPY--------------------CAERPKLRATDLFSPAE---TEIDHVIERR
92109262	*Nitrobacter hamburgensis* X14	K--------LSEQQKAERQREQRRNQ--DKNKARAEELAKFGR---------------------------PANPRNL---LKM-RLWEELAHDP-LDRK----------CVY-----------------------TGEQISIERLLSDE---VDIDHILPVA
148255343	*Bradyrhizobium* sp. BTAi1	K--------LSPRRLAELEREQAENQ--RKNDKRTSLLRKLGL---------------------------PASTHNL---LKL-RLWDE------QGDVASE-------CPY-----------------------TGEAIGLERLVSDD---VDIDHLIPFS
34557790	*Wolinella succinogenes* DSM 1740	N--------TKGEIEDIKESQRKNE--KERKEAADWIAETSFQV--------------------------PLTRKNI---LKK-RLYIQ------QDGR----------CAY-----------------------TGDVIELERLFDEGY--CEIDHILPRS
218563121	*Campylobacter jejuni* NCTC 11168	G--------KNHSQRAKIEKEQNENY--KAKKDAELECEKLGL---------------------------KINSKNI---LKL-RLFKE------QKEF----------CAY-----------------------SGEKIKISDLQDEKM---LEIDHIYPYS
Jnet	218563121	-----------HHHHHHHHHHHHHHH--HHHHHHHHHHHH---------------------------------HHH---HHH-HHHHH---EEEEEEEEE-
291276265	*Helicobacter mustelae* 12198	C--------KAKSARMQLEKINKKNK--SENDAASQLLEVLGL---------------------------PNTYNNR---LKC-KLWKQ------QEEY----------CLY-----------------------SGEKITIDHLKDQRA---LQIDHAFPLS
222109285	*Acidovorax ebreus* TPSY	S--------RPLDERNKVKRAQEEFR--DRNDRARSEFERDFGY--------------------------KPKAAAF---EKW-MLYRE------QLGQ----------CAY-----------------------SQQPLDIQRVLDDHNY--AQVDHALPYS
365156657	*Bacillus smithii* 7 3 47FAA	S--------HSFDERKKIQKDQTENR--KKNETAIKQLIEYELTK-------------------------NPTGLDI---VKF-KLWSE------QQGR----------CMY-----------------------SLKPIELERLLEPGY--VEVDHILPYS
220930482	*Clostridium cellulolyticum* H10	G--------MTRQDRDNLKKEHENNR--KAREKISDLIRQNGR---------------------------VASGLDI---LKW-RLWED------QGGR----------CAY-----------------------SGKPIPVCDLLNDSL--TQIDHIYPYS
297182908	uncultured delta proteobact. HF0070 07E19	G--------KGRELRERIKLDQL------DKSKQNDQRAEDFRAEFQQ---------------------APRGDQS---LRY-RLWKE------QNCT----------CPY-----------------------SGRMIPVNSVLSED---TQIDHILPIS
154250555	*Parvibaculum lavamentivorans* DS-1	G--------KSKREREEIQSGIRRNE--KQRKKATEDLIKNGIA--------------------------NPSRDDV---EKW-ILWKE------GQER----------CPY-----------------------TGDQIGFNALFREGR--YEVEHIWPRS
218767588	*Neisseria meningitidis* Z2491	G--------KSFKDRKEIEKRQEENR--KDREKAAAKFREYFPNFVG-----------------------EPKSKDI---LKL-RLYEQ------QHGK----------CLY-----------------------SGKEINLGRLNEKGY--VEIDHALPFS
15602992	*Pasteurella multocida* str. Pm70	G--------KSFKERREIQKQQEDNR--TKRESAVQKFKELFSDFSS-----------------------EPKSKDI---LKF-RLYEQ------QHGK----------CLY-----------------------SGKEINIHRLNEKGY--VEIDHALPFS
187736489	*Akkermansia muciniphila* ATCC BAA-835	T-------TFSAMDSKKIQRELTLRQ--KSHTDAVNRLKRKLPGK-------------------------ALSANLI---RKC-RIAMD------MNWT----------CPF-----------------------TGATYGDHELEN------LELEHIVPHS
315605738	*Actinomyces* sp. oral taxon 180 str. F0310	L--------KSA-SLLEEERERWERF-EARREIRQKEMYKRLGISG------------------------PFRRSDQ---VRY-EILDL------QDCA----------CLY-----------------------CGNEINFQT---------FEVDHIIPRV
117929158	*Acidothermus cellulolyticus* 11B	S--------ESRERQAEEEAARRAHR--KANDRIRAELRASGLS--------------------------DPSPADL---VRA-RLLEL------YDCH----------CMY-----------------------CGAPISWEN---------SELDHIVPRT
189440764	*Bifidobacterium longum* DJO10A	F--------SSVAFARKDKREYEKNN--EKRSIFRSSLSEQLRADEQME---------------------KVRESDL---RRL-EAIQR------QNGQ----------CLY-----------------------CGRTITFRT---------CEMDHIVPRK
403744858	*Alicyclobacillus hesperidum* URH17-3-68	S--------KNLDERRKIEKAQKENQ--KN----NEQIKDEIRE-ILGS--------------------AHVTGRDI---VKY-KLFKQ------QQEF----------CMY-----------------------SGEKLDVTRLFEP---GYAEVDHIIPYG
407803669	*Alcanivorax* sp. W11-5	K--------GGVTRRNDIEKQNKRFA--SE----RKKAAQSIEE--NGK---------------------TPTPARI---LRY-QLWIE------QGHQ----------CPY-----------------------CESNISLEQALSG---AYTNFEHILPRT
423317190	*Bergeyella zoohelcum* ATCC 43767	K--------NSAKERATIHKRNKDNQ--TI----NNKIKETLVK--NKK---------------------ELSLANI---EKV-KLWEA------QRHL----------SPY-----------------------TGQPIPLSDLFDK---EKYDVDHIIPIS
402847315	*Porphyromonas* sp. oral taxon 279 str. F0450	K--------MSREERERMARNNKDRE--ER----NKGVAAKIRE--CGL---------------------YPTKPRI---QKY-MLWKE------AGRQ----------CLY-----------------------CGRSIEEEQCLRE---GGMEVEHIIPKS
404487228	*Barnesiella intestinihominis* YIT 11860	K--------QSKEERSNTYKSINKNQ--RE----NEQIAKRIVE--YGV----------------------PTRSRI---QKY-KMWEE------SKHC----------CIY-----------------------CGQPVDVGDFLRG---FDVEVEHIIPKS
374384763	*Odoribacter laneus* YIT 12061	R--------SSKTEREKQSRQIRENE--KK----NEAAKVKLAE--YGL---------------------KAYRDNI---QKY-LLYKEIE----EKGGTVC-------CPY-----------------------TGKTLNISHTLGSD--NSVQIEHIIPYS
384109266	*Treponema* sp. JC4	K--------NNVEKKAEIARKQNQRA--KENIAINDTISALYHTAFPGK--------------------SFYPNRNDR---MKY-RLWSELG----LGNK----------CIY-----------------------CGKGISGAELFTK----EIEIEHILPFS
402849997	*Rhodovulum* sp. PH10	K--------LNREQKERLDRENRKNR--EE----NERRTAILAE--HGQ---------------------RDTAENK---IRL-RLFEE------QARANAGIAL----CPY-----------------------TGRAIGIAELFTS----EVEIDHILPVS
Jnet	331001027	---------------HHHHHH------------------HHHHHHH------------------HHHHH----HHHHH---HHH-HHH--------H---EEE---
331001027	*Parasutterella excrementihominis* YIT 11859	F--------TASLTDLKYIQLK--------------EQKLKKKLEDI-----------------QRNEENQEKRWLSKE---ERI-RAD--------SHGI----------CAY-----------------------TGRPLDDV----------GEIDHIIPRS
34557932	*Wolinella succinogenes* DSM 1740	Y--------EESLRKSKTGSND-----------RVINSKKDRDGKKL-----------------AKAKENAEDRLKDKD---KRI-KAF--------SSGI----------CPY-----------------------CGDTIGDD----------GEIDHILPRS
54296138	*Legionella pneumophila* str. Paris	F--------EESFKKIK-GSSS--------------DKTLEQ----------------------AIEKQNIQ--WEEKF---QRI-INA--------SMNI----------CPY-----------------------KGASIGGQ----------GEIDHIYPRS
319941583	*Sutterella wadsworthensis* 3 1 45B	F--------SASVADLK----K--------------NKRVKDKM--------------------LSEAEKLETRWLIKN---ERI-KKA--------SRGT----------CPY-----------------------TGDRLAEG----------GEIDHILPRS
254447899	gamma proteobact. HTCC5015	F--------TAELEEIKRGRGS--------------AKAKKA----------------------KELGEKSKAGWVSKT---ERI-KTS--------SEGI----------CPY-----------------------TGAPLGGS----------GEIDHIIPRS
118497352	*Francisella novicida* U112	F--------EPALADVKGKSLK--------------DRRKK-----------------------ALERISPENIFKDKN---NRI-KEF--------AKGI----------SAY-----------------------SGANLTDGDFDGAK----EELDHIIPRS
Motifs		____{____H____
informative positions		*---****------------***---------------------------***************-*********

```
227494853   Actinomyces coleocanis DSM 15436#      GLGST------NTRDNLVATCERCNK-SKSN-KPFAV-------WAAECGIP-GVSVAEALK-------RVDF----------------WIADG-FASSKEHREL-QKGVKDRLKRKV-----------------------SDPEIDNRSMESVAWMARELAHRVQYYFDEKHTGT---------------KVRVFRGSL
328956315   Coriobacterium glomerans PW2           Y-VKD------DSLENKALVYREENQ-RKTDMLLIDP--------------EIRRRMSGYWR-------MLHE----------------AKLIG-------------DKKFRNLLRSR---------------------IDDKALKGFIARQLVETGQMVKLVRSLLEARYPET-------------------NIISVKASI
227824983   Acidaminococcus sp. D21                M-VKD------DSLDNKVLVQSEING-EKSSRYPLDA--------------AIRNKMKPLWD-------AYYN----------------HGLIS-------------LKKYQRLTRST---------------------PFTDDEKWDFINRQLVETRQSTKALAILLKRKFPDT-------------------EIVYSKAGL
303229466   Veillonella atypica ACS-134-V-Col7a     L-TKD------DSFDNLVLCERTANA-KKSDIYPIDN--------------RIQTKQKPFWA-------FLKH----------------QGLIS-------------ERKYERLTRIA---------------------PLTADDLSGFIARQLVETNQSVKATTTLLRRLYPDI-------------------DVVFVKAEN
34762592    Fusobacterium nucleatum ATCC 49256     KVIKD------DSFDNLVLVLKNENA-EKSNEYPVKK--------------EIQEKMKSFWR-------FLKE----------------KNFIS-------------DEKYKRLTGKD---------------------DFELRGFMARQLVNVRQTTKEVGKILQQIEPEI-------------------KIVYSKAEI
374307738   Filifactor alocis ATCC 35896            K-IKD------DSIDNLVLVNKTYNA-KKSNELLSED--------------IQKKMHSFWL-------SLLN----------------KKLIT-------------KSKYDRLTRKG---------------------DFTDEELSGFIARQLVETRQSTKAIADIFKQIYSS-------------------EVVYVKSSL
320528778   Solobacterium moorei F0204             K-IKD------DSITNRVLVEKDING-EKTDIYPISE--------------DIRQKMQPFWK-------ILKE----------------KGLIN-------------EEKYKRLTRNY---------------------ELTDEELSSFVARQLVETQQSTKALATLLKKEYPSA-------------------KIVYSKAGN
291520705   Coprococcus catus GD-7                 K-TMD------DSLNNRVLVKKNYNA-IKSDTYPLSL--------------DIQKKMMSFWK-------MLQQ----------------QGFIT-------------KEKYVRLVRSD---------------------ELSADELAGFIERQIVETRQSTKAVATILKEALPDT-------------------EIVYVKAGN
42525843    Treponema denticola ATCC 35405         K-IKD------DSISNRVLVCSSCNK-NKEDKYPLKS--------------EIQSKQRGFWN-------FLQR----------------NNFIS-------------LEKLNRLTRAT---------------------PISDDETAKFIARQLVETRQATKVAAKVLEKMFPET-------------------KIVYSKAET
304438954   Peptoniphilus duerdenii ATCC BAA-1640  K-IYD------DSLENRVLVKKNLNH-EKGNQYPIPE--------------KVLNKNAYGFWK-------ILFD----------------KGLIG-------------QKKYTRLTRRT---------------------PFEERELAEFIERQIVETRQATKETANLLKNICQDS-------------------EIVYSKAEN
224543312   Catenibacterium mitsuokai DSM 15897    L-VKD------DSFDNRVLVVPSENQ-RKLDDLVVPF--------------DIRDKMYRFWK-------LLFD----------------HELIS-------------PKKFYSLIKTE---------------------YTERDEERFINRQLVETRQITKNVTQIIEDHYSTT-------------------KVAAIRANL
116628213   Streptococcus thermophilus LMD-9       F-LKD------NSIDNKVLVSSASNR-GKSDDVPSLE--------------VVKKRKTFWY-------QLLK----------------SKLIS-------------QRKFDNLTKAE-----------------RGGLSPEDKAGFIQRQLVETRQITKHVARLLDEKFNNKKDENNRAVRTV--------KIITLKSTL
24379809    Streptococcus mutans UA159             F-IKD------NSIDNRVLTSSKENR-GKSDDVPSKD--------------VVRKMKSYWS-------KLLS----------------AKLIT-------------QRKFDNLTKAE-----------------RGGLTDDDKAGFIKRQLVETRQITKHVARILDERFNTETDENNKKIRQV--------KIVTLKSNL
13622193    Streptococcus pyogenes SF370           F-LKD------DSIDNKVLTRSDKNR-GKSDNVPSEE--------------VVKKMKNYWR-------QLLN----------------AKLIT-------------QRKFDNLTKAE-----------------RGGLSELDKAGFIKRQLVETRQITKHVAQILDSRMNTKYDENDKLIREV--------KVITLKSKL
310286728   Bifidobacterium bifidum S17            V-TQN------DSIDNRVLVARAENA-RKTDSFTYMP--------------QIADRMRNFWQ-------ILLD----------------NGLIS-------------RVKFERLTRQN---------------------EFSEREKERFVQRSLVETRQIMKNVATLMRQRYGNSA------------------AVIGLNAEL
366983953   Oenococcus kitaharae DSM 17330         Y-TKD------NSLDNRVLVSNITNR-RKSDSSNYLP--------------ALIDKMRPFWS-------VLSK----------------QGLLS-------------KHKFANLTRTR---------------------DFDDMEKERFIARSLVETRQIIKNVASLIDSHFGGET------------------KAVAIRSSL
422884106   Streptococcus sanguinis SK49           F-LPI------DSIDNKVLTHRENNQ-QKLNNIP-DK--------------ETVANMKPFWE-------KLYN----------------AKLIS-------------QTKYQRLTTSERTPDGV--------------LTESMKAGFIERQLVETRQIIKHVARILDNRF--SDT-----------------KIITLKSQL
339625081   Fructobacillus fructosus KCTC 3544      F-TKD------DSLDNRVLVSRPENA-RKSDSFAYTD--------------EVQKQDGSLWT-------SLLK----------------SGFIN-------------RKKYERLTKAGK--------------------YLDGQKTGFIARQLVETRQIIKNVASLIEGEY--ENS-----------------KAVAIRSEI
306821691   Eubacterium yurii ATCC 43715           F-VKD------DSLDNLVLVKKEINN-RKQND-PITP--------------QIQASCQGFWK-------ILHD----------------QGFMS-------------NEKYSRLTRKTQE-------------------FSDEEKLSFINRQIVETGQATKCMAQILQKSM-GEDV-----------------DVVFSKARL
336394882   Lactobacillus farciminis KCTC 3681     Y-IPD------DSLENKALVLAKENQ-RKADDLLLNS--------------NVIDRNLERWT-------YMLN----------------NNMIG-------------LKKFKNLTRRV---------------------ITDKDKLGFIHRQLVQTSQMVKGVANILDNMYKNQGT-----------------TCIQARANL
323463801   Staphylococcus pseudintermedius ED99    F-IKD------DSIDNKVLVIKKMNQ-TKGDQVPLQFI-------------QQPYERIAYWK-------SLNK----------------AGLIS-------------DSKLHKLMKPE---------------------FTAMDKEGFIQRQLVETRQISVHVRDFLKEEY--PNT-----------------KVIPMKAKM
389815359   Planococcus antarcticus DSM 14505      F-VKD------DSLDNLALVMPEANQ-RKNQVGQNKM-------PLEIIEANQQYAMRTLWE-------RLHE----------------LKLIS-------------SGKLGRLKKPS---------------------FDEVDKDKFIARQLVETRQIIKHVRDLLDERF--SKS-----------------DIHLVKAGI
258509199   Lactobacillus rhamnosus GG             L-IKD------DSLDNRVLVNATINR-EKNNVFASTL--------------FAGKMKATWR-------KWHE----------------AGLIS-------------GRKLRNLMLRP---------------------DEIDKFAKGFVARQLVETRQIIKLTEQIAAAQYPNT-------------------KIIAVKAGL
169823755   Finegoldia magna ATCC 29328            MKKDD------SIINNLVLVNKNANQTTKGNIYPVPS-------------SIRNNPKIYNYWK-------YLME----------------KEFIS-------------KEKYNRLIRNT---------------------PLTNEELGGFINRQLVETRQSTKAIKELFEKFYQKS-------------------KIIPVKASL
Jnet        227501312                              ----------------HHHHHHHHH-HH-------HH------------H-----HHHHHHHH-------HHHH--------------------------------------HHHHHHH---------------------------HHHHHHHHHHH----HHHHHHHHHHHHHHHHH-------------------------EEEEEEEEE
47458868    Mycoplasma mobile 163K                 I-SFD------DSFSNLVIVNKLDNA-KKSNDLSAKQ------FIEKIYGIEKLKEAKENWG-------NWYL----------------RNANG----------KAFNDKGKFIKLYTID-------------------NLDEFDNSDFINRNLSDTSYITNALVNHLTFSNSKYKY-----------------SVVSVNGKQ
284931710   Mycoplasma gallisepticum str. F        I-SFD------DSSSNKLLVLAESNQ-AKSNQTPYEF--------------ISSGNAGIKWE-------DYEA----------------YCRKFKDGDSSLLDSTQRSKKFAKMMKTD-------------------TSSKYDIGFLARNLNDTRYATIVFRDALEDYANNHLVEDKPMF-----------KVVCINGSV
71894592    Mycoplasma synoviae 53                 K-SAD------DSWFNKLLVKKSTNQ-LKKNKTVWEY--------------YQNESDPEAKWN-------KFVA----------------WAKRIYLVQKSDKESKDNSEKNSIFKNKK-------PNLKFKNITKKLFDPYKDLGFLARNLNDTRYATKVFRDQLNNYSKHHSKDDENKLF-----------KVVCMNGSI
363542550   Mycoplasma ovipneumoniae SC01          M-SYD------NSQANKILTEKAENL-KKGKLIASEY--------------IKRNGDE--FYN-------KYYE----------------KAKELFI-----NKYKKNKKLDSYVDLDEDS-----AKNRFRFLTLQDYDEF-QVEFLARNLNDTRYSTKLFYHALVEHFENNEFFTYIDENSSKHKV----KISTIKGHV
384393286   Mycoplasma canis PG 14                 L-CFD------DSSANKVLVHKQSNQ-EKSNSLPYEY--------------IK-QGHSGWNWD-------EFTK----------------YVKRVFV-----------NNVDSI--LSKKE-----RLKKSENLLTASYDGYDKLGFLARNLNDTRYATILFRDQLNNYAEHHLIDNK-KMF----------KVIAMNGAV
238924075   Eubacterium rectale ATCC 33656         I-SFD------DARSNKVLVYRSENQ-KKGNQTPYYY---------------LTHSHSEWS-----FEQYKA-----------------TVMNL----SKKKEYAISRKKIQNLLYSE--------------------DITKMDVLKGFINRNINDTSYASRLVLNTIQNFFMANEADT----------------KVKVIKGSY
315149830   Enterococcus faecalis TX0012           I-SLD------DSINNKVLVLSKANQ-VKGQQTPYDA--------WMDGSFKKINGKFSNWD-------DYQK----------------WVESR-------------HFSHKKENNLLETR-------------------NIFDSEQVEKFLARNLNDTRYASRLVLNTLQSFFTNQET-----------------KVRVVNGSF
116627542   Streptococcus thermophilus LMD-9       I-TFD------DSLANKVLVYATANQ-EKGQRTPYQA--------------LDSMDDAWS-----FRELKA-----------------FVRES-------KTLSNKKKEYLLTEE---------------------DISKFDVRKKFIERNLVDTRYASRVVLNALQEHFRAHKIDT-----------------KVSVVRGQF
315659848   Staphylococcus lugdunensis M23590      V-SFD------NSYHNKVLVKQSENS-KKSNLTPYQY--------------FNSGKSKLSYN-------QFKQ----------------HILNL-----SKSQDRISKKKKEYLLEER---------------------DINKFEVQKEFINRNLVDTRYATRELTNYLKAYFSANNMNV-----------------KVKTINGSF
160915782   Eubacterium dolichum DSM 3991          I-SLD------DSITNKVLVTHRENQ-EKGNLTPISA--------------FVKGRFTKGSLA-------QYKA----------------YCLKL-KEKNIKTNKGYRKKVEQYLLNEN---------------------DIYKYDIQKEFINRNLVDTSYASRVVLNTLTTYFKQNEIPT-----------------KVFTVKGSL
325677756   Ruminococcus albus 8                   L-ILD------NTINNKALVYAEENQ-KKGQRTPLMY--------------MNEAQAADYR-------VRVN----------------TMFKS---------KKCSKKKYQYLMLPD--------------------LNDQELLGGWRSRNLNDTRYICKYLVNYLRKNLRFDRSYESSDEDDLKIRDHY--RVFPVKSRF
225377804   Roseburia inulinivorans DSM 16841      I-TFD------DSYRNKVLVTAQENR-QKGNRTPYEY--------------FGADEKRWE-------DYEA----------------SVRLL---------VRDYKKQQKLLKKN--------------------FTEEERKEFKERNLNDTKYITRVVYNMIRQNLELEPFNHPEKKK-----------QVWAVNGAV
336393381   Lactobacillus coryniformis KCTC 3535   I-SWD------DSYTNKVLTSAKCNR-EKGNRIPMVY--------------LANNEQRLNALT-------NIAD----------------NIIRN-------------SRKRQKLLKQK--------------------LSDEELKDWKQRNINDTRFITRVLYNYFRQAIEFNPELEKKQ--------------RVLPLNGEV
310780384   Ilyobacter polytropus DSM 2926          R-SMD------DSYSNKVLVLSGENR-KKSNLLPKEY--------------FDRQGRDWD-------TFVL----------------NVKAM-----------KIHPRKKSNLLKEK--------------------FTREDNKDWKSRALNDTRYISRFVANYLENALEYRDDSPKK-------------RVFMIPGQL
301311869   Bacteroides sp. 20 3                   R-YFD------DSLSNKVICESEINK-LKDRSLGYEF-----------IKNHHGEKVELAFD-------KPVE----------------VLSVEAYEKLVHESYSHNRSKMKKLLMED---------------------IPDQFIERQLNDSRYISKVVKSLLSNIVREENEQEAISK-----------NVIPCTGGI
385811609   Ignavibacterium album JCM 16511        K-MKN------DSTNNLVICELGVNK-AKGDRLAANF-----------ISESNG-KCKFG-E-------VEYT----------------LLKYGDYLQYCKDTFKYQKAKYKNLLATE---------------------PPEDFIERQINDTRYIGRKLAELLTPVVKD-------SK-----------NIIFTIGSI
60683389    Bacteroides fragilis NCTC 9343         R-LFD------DSFSNKTLEARSVNI-EKGNKTAYDF----------VKEKFGESGADNSLE-------HYLN----------------NIEDL------FKSGKISKTKYNKLKMAE-------------------QDIPDGFIERDLRNTQYIAKKALSMLNEISH------------------RVVATSGSV
319957206   Nitratifractor salsuginis DSM 16511    L-GGL------STDYNTIVTLKSVNA-AKGNRLPGDW--------------LAGNPDYRERIG-------MLSE----------------KGLID-------------WKKRKNLLAQS---------------------LDEIYTENTHSKGIRATSYLEALVAQVLKRYYPFPDPELRKNGI-----------GVRMIPGKV
187250660   Elusimicrobium minutum Pei191          E-SED------NGRNNLVISHSACNA-DKAKRSPWAA--------------FASAAKDSKYD--------YN----------------RILSNVKENIPHKAWRFNQGAFEKFIENK-----------------------PMAARFKTDNSYISKVAHKYLACLFEKP-------------NIICVKGSL
325972003   Sphaerochaeta globus str. Buddy        D-TAD------NSYGNKVVAHRQCND-IKGKRTPYAA-----------------FSNTSAWG-------PIMH----------------YLDETPGMWRKRRKFETNEEEYAKYLQSK-------------------------GFVSRFESDNSYIAKAAKEYLRCLFNPNNVT-----------------AVGSLKGME
296446027   Methylosinus trichosporium OB3b        R-FAD------DSYANKTLCMAKANQ-DKKGRTPYEW--------------FKAEKTDTEWD-------AFIV----------------RVEAL---------ADMKGFKKRNYKLRNA-----------------------EEAAAKFRNRNLNDTRWACRLLAEALKQLYPKGEKDKDGKERR---------RVFSRPGAL
347536497   Flavobacterium branchiophilum FL-15     R-SQD------NSQMNKTLCSQRFNREVKKQSMPIELNNHLE------------ILPRIAHWKEEADNLTREIEIISRSIKAAATKEI--KDKKIRRRHYLTLKRDYLQGKYDRFIWEE------------------------PKVGFKNSQIPDTGIITKYAQAYLKSYFKKVE-----------------------SVKGGM
345885718   Prevotella sp. C561                    L-SFD------SSDMNLTLCDAHYNRFIKKNHIPTDMPNYDKAITIDGKEYPAITSQLQRWVERVERLNRNVEYWKGQARRAQNKDR--KDQCMREMHLWKMELEYWKKKLERFTVTE--------------------------VTDGFKNSQLVDTRVITRHAVLYLKSIFPHVD------------------------VQRGDV
282880052   Prevotella timonensis CRIS 5C-B1        V-GGD------STQMNLTLCDNRFNREVKKAKLPTELANHEE------------ILTRIEPWKNKYEQLVKERD--KQRTFAGMDKAV--KDIRIQKRHKLQMEIDYWRGKYERFTMTE-----------------------VPEGFSRRQGTGIGLISRYAGLYLKSLFHQADSRNKSNVY-----------------VVKGVA
312879015   Aminomonas paucivorans DSM 12260       R-SLD------NGWHNRVLVHGKDNR-DKGNRTPFEA--------------FGGDTARWD-------RLVA----------------WVQAS------------HLSAPKKRNLLRED---------------------FGEEAERELKDRNLTDTRFITKTAATLLRDRLTFHPEAPKD--------------PVMTLNGRL
294086111   Candidatus Puniceispirillum marinum IMCC1322  L-TLD------DSMANKTVCFRQANR-DKGNRAPFDA--------------FGNSPAGYDWQ-------EILG----------------RSQNL-------------PYAKRWRFLPDA--------------------MKRFEADGGFLERQLNDTRYISRYTTEYISTIIPKN-------------------KIWVVTGRL
330822845   Alicycliphilus denitrificans K601      K-TLD------DSLNNRTVAMRQANR-IKRNRTPWDA--------------RAEFEAQGWSYE-------DILQ----------------RAERM----PLRKRYRFAPDGYERWLGDD------------------------KDFLARALNDTRYLSRVAAEYLRLVCP-------------------GTRVIPGQL
344171927   Ralstonia syzygii R24                  K-TLD------DSFANKVLAQHDANR-YKGNRGPFEA--------------FGANRDGYAWD-------DIRA----------------RAAVL--------------PRNKRNRFAETA-------------------MQDWLHNETDFLARQLTDTAYLSRVARQYLTAICSKD------------------DVYVSPGRL
159042956   Dinoroseobacter shibae DFL 12           R-TLD------DSFPNRTLCLREANR-QKRNQTPWQA--------------WGDTPHWH-------AIAA----------------NLKNL--------------PENKRWRFAPDA--------------------MTRFEGENGFLDRALKDTQYLARISRSYLDTLFTKGG-----------------HVWVVPGRF
83591793    Rhodospirillum rubrum ATCC 11170       R-GGR------DSLDNMVLCQSDANK-TKGDKTPWEA--------------FHDKPGWI------AQRDD-------------------FLARLDPQTAKALAWRFADDAGERVARKS---------------------AEDEDQGFLPRQLTDTGYIARVALRYLSLVTNEPN-----------------AVVATNGRL
288957741   Azospirillum sp. B510                  V-SLD------DSAANKVVCLREANR-IKRNRSPWEA--------------FGHDSERWA-------GILA----------------RAEAL--------------PKNKRWRFAPDA--------------------LEKLEGEGGLRARHLNDTRHLSRLAVEYLRCVCP-----------------------KVRVSPGRL
427429481   Caenispirillum salinarum AK4           M-G-G------DGPDNLVLAHKDCNN-AKGKKTPHEH----------AGDLLDSPALAALWQ-------GWRKENADRLKGKGHKARTPREDKD------------FMDRVGWRFEEDA-----------------RAKAEENQERRGRRMLHDTARATRLARLYLAAAVMPEDPAEIGAPPVETPPSPEDPTGYTAIYRT
92109262    Nitrobacter hamburgensis X14           M-TLD------DSPANKIICMRYANR-HKRKQTPSEA--------FGSSPTLQGHRYNWD-------DIAA----------------RATGL--------------PRNKRWRFDANA--------------------REEFDKRGGFLARQLNETGWLARLAKQYLGAVTDPN-----------------QIWVVPGRL
148255343   Bradyrhizobium sp. BTAi1               I-SWD------DSAANKVVCMRYANR-EKGNRTPFEA--------------FGHRQGRPYDWA-------DIAE----------------RAARL--------------PRGKRWRFGPGA--------------------RAQFEELGDFQARLLNETSWLARVAKQYLAAVTHPH-----------------RIHVLPGRL
34557790    Wolinella succinogenes DSM 1740         R-SAD------DSFANKVLCLARANQ-QKTDRTPYEW--------------FGHDAARWN-------AFET----------------RTSAP------SNRVRTGKGKIDRLLKKN--------------------FDENSEMAFKDRNLNDTRYMARAIKTYCEQYWVFKNSHTKA----------------PVQVRSGKL
218563121   Campylobacter jejuni NCTC 11168        R-SFD------DSYMNKVLVFTKQNQ-EKLNQTPFEA--------------FGNDSAKWQ------------------------------KIEVL---------AKNLPTKKQKRILDKN--------------------YKDKEQKNFKDRNLNDTRYIARLVLNYTKDYLDFLPLSDDENTKLNDTQKGSKVHVEAKSGML
Jnet        218563121                              ----------------EEHHHHHHHH-H-----HHHH----------------HH---HHHH-------------------------------HHHHH---------H----HHHHHHHH-------------------HHHHH-HHH----HHHHHHHHHHHHHHHHHH------------------EEEEEEE---
291276265   Helicobacter mustelae 12198            R-SLD------DSQSNKVLCLTSSNQ-EKSNKTPYEW--------------LGSDEKKWD-------MYVG----------------RVYSS----------NFSPSKKRKLTQKN--------------------FKERNEEDFLARNLVDTGYIGRVTKEYIKHSLSFLPLPDGKKE-----------HIRIISGSM
222109285   Acidovorax ebreus TPSY                 R-SYD------DSKNNKVLVLTHENQ-NKGNRTAFEY----------LTSFPDGEDGERWR-------TFVA----------------WVQGN-----------KAYRMAKRNLLRKN--------------------YGVDESKGFIDRNLNDTRYICKFFKNYVEEHLQLAARADGDTAR-----------RCVVVNGQL
365156657   Bacillus smithii 7 3 47FAA             R-SLD------DSYANKVLVLTKENR-EKGNHTPVEY--------------LGLGSERWK-------KFEK----------------FVLAN---------KQFSKKKKQNLLRLR--------------------YEETEEKEFKERNLNDTRYISKFFANFIKEHLKFADGDGGQ-----------KVYTINGKI
220930482   Clostridium cellulolyticum H10         R-SMD------DSYMNKVLVLTDENQ-NKRSYTPYEV--------------WGSTEKWE-------DFEA----------------RIYSM------------HLPQSKEKRLLNRN--------------------FITKDLDSFISRNLNDTRYISRFLKNYIESYLQFSNDSPKS-----------CVVCVNGQC
297182908   uncultured delta proteobact. HF0070 07E19  Q-SFD------NSLSNKVLCFTEENA-QKSNRTPFEY--------------------LDAA-------DFQRL----------------EAISG-------------NWPEAKRNKLLHKS--------------------FGKVAEEWKSRALNDTRYLTSALADHLRHHLPDS-------------------KIQTVNGRI
154250555   Parvibaculum lavamentivorans DS-1      R-SFD------NSPRNKTLCRKDVNI-EKGNRMPFEA--------------FGHDEDRWS-------AIQI----------------RLQGM---VSAKGGTGMSPGKVKRFLAKT-----------------------MPEDFAARQLNDTRYAAKQILAQLKRLWPDMGPEAPV---------------KVEAVTGQV
218767588   Neisseria meningitidis Z2491           R-TWD------DSFNNKVLVLGSENQ-NKGNQTPYEY--------------FNGKDNSREWQ-------EFKA----------------RVETS-------------RFPRSKKQRILLQK--------------------FDED--GFKERNLNDTRYVNRFLCQFVADRMRLTGKGK--------------RVFASNGQI
15602992    Pasteurella multocida str. Pm70        R-TWD------DSFNNKVLVLASENQ-NKGNQTPYEW--------------LQGKINSERWK-------NFVA----------------LVLGS-------------QCSAAKKQRLLTQV--------------------IDDN---KFIDRNLNDTRYIARFLSNYIQENLLLVGKNKK--------------NVFTPNGQI
187736489   Akkermansia muciniphila ATCC BAA-835    F-RQS------NALSSLVLTWPGVNR-MKGQRTGYDF----------VEQEQENPVPDKPNLHICSLNNYRE----------------LVEKLDDKKGHEDDRRRKKKRKALLMVRG----------LSHKHQSQNHEAMKEIGMTEGMMTQSSHLMKLACKSIKTSLPDA--------------------HIDMIPGAV
315605738   Actinomyces sp. oral taxon 180 str. F0310  DASSD------SRRTNLAAVCHSCNS-AKGG-LAFGQ-------WVKRGDCPSGVSLENAIK-------RVRS----------------WSKDR-LGLTEKAMGKRKSEVISRLKTEM-----------------------PYEEFDGRSMESVAMAEIKRKIEGYFNSDRPEGCAAV---------------QVNAYSGRL
117929158   Acidothermus cellulolyticus 11B        D-GGS------NRHENLAITCGACNK-EKGR-RPFAS--------------WAETSNRVQLR-------DVID----------------RVQKL----KYSGNMYWTRDEFSRYKKSV--------------------VARLKRRTSDPEVIQSIESTGYAAVALRDRLLSYGEKNGVA-----------------QVAVFRGGV
189440764   Bifidobacterium longum DJO10A          GVGST------NTRTNFAAVCAECNR-MKSN-TPFAI----------WARSEDAQTRGVSLA-------EAKK----------------RVTMFTFNPKSYAPREVKAFKQAVIARLQ--------------------QTEDDAAIDNRSIESVAWMADELHRRIDWYFNAKQYVNSASIDDAEAETMKT-TVSVFQGRV
403744858   Alicyclobacillus hesperidum URH17-3-68  I-SFD------DSYDNKVLVKTEQNR-QKGNRTPLEY----------LRDKPE-------QKA------KFIA----------------LVES--IPLSQKKK-----NHLLMDKRAI-----------------DLEQ----EGFRERNLSDTRYITRALMNHIQAWLLFDETASTRS-------------KRVVCVNGAV
407803669   Alcanivorax sp. W11-5                  LTQIG------RKRSELVLAHRECND-EKGNRTPYQA----------FGHDDR-------RWR-------IVEQ----------------RANA--LP-KKSSRK----TRLLLLKDFE----------------GEALTDESIDEFADRQLHESSWLAKVTTQWLSSLG--------------------SDVYVSRGSL
423317190   Bergeyella zoohelcum ATCC 43767         R-YFD------DSFTNKVISEKSVNQ-EKANRTAMEY----------FEVGSL-------KYS------IFTK----------------EQFI--AHVNEY-------FSGVKRKNLL----------------ATSIP----EDPVQRQIKDTQYIAIRVKEELNKIVGN-------------------ENVKTTTGSI
402847315   Porphyromonas sp. oral taxon 279 str. F0450  V-LYD------DSYGNKTCACRRCNK-EKGNRTALEY----------IRAKGR--------EA-------EYMK----------------RIND--LLKEKKIS-----YSKHQRLRWL----------------KEDIP----SDFLERQLRLTQYISRQAMAILQQGI---------------------RRVSASEGGV
404487228   Barnesiella intestinihominis YIT 11860  L-YFD------DSFANKVCSCRSCNK-EKNNRTAYDY----------MKSKGE-------KALS------DYVE----------------RVNT--MYTNNQISK----TKWQNLLTPV----------------DKIS-----IDFIDRQLRESQYIARKAKEILTSIC----------------------YNVTATSGSV
374384763   Odoribacter laneus YIT 12061           I-SLD------DSLANKTLCDATFNR-EKGELTPYDF----------YQKDPSPEKWGASSWE-------EIED----------------RAFR--LLPYAK------AQRFIRRKPQ----------------ES------NEFISRQLNDTRYISKKAVEYLSAIC----------------------SDVKAFPGQL
384109266   Treponema sp. JC4                      R-TLL------DAESNLTVAHSSCNA-FKAERSPFEA----------FGTNPS------GYSWQ------EIIQ----------------RANQ--LKNTSKKNK----FSPNAMDSFE----------------KD------SSFIARQLSDNQYIAKAALRYLKCLVENP-------------------SDVWTTNGSM
402849997   Rhodovulum sp. PH10                    L-TLD------DSLANRVLCRREANR-EKRRQTPFQA----------FGATPA--------WN-------DIVA----------------RAAK--LPPNKRWR----FDPAALERFE----------------RE------GGFLGRQLNETKYLSRLAKIYLGKICDP-------------------DRVYVTPGTL
Jnet        331001027                              ------EEEEE----EEEEEE-----------EEEE------------------HHHH-------HHHHHHHHHHHH-------HHH-H-----------------------------------------------------EEEE---HHHHHHHHHHHH-----HHHHHHHHHHH------------------------EEE----H
331001027   Parasutterella excrementihominis YIT 11859  L-TLKKSESIYNSEVNLIFVSAQGNQ-EKKNNIYLLS-------------NLAKNYLAAVFGTSDLSQITNEIESTV-------LQL-KAAGRL--------------------------------------------GYFDLLSEKERACARHALFLNSDSEARRAVIDVLGSR------------------RKASVNGTQ
34557932    Wolinella succinogenes DSM 1740         H-TLKIYGTVFNPEGNLIYVHQKCNQ-AKADSIYKLS-------------DIKA-----------GVSAQWIEEQV-------ANI-KGY---------------------------------------------------KTFSVLSAEQQKAFRYALFLQNDNEAYKKVVDWLRTD-------------------QSARVNGTQ
54296138    Legionella pneumophila str. Paris      L-SKKHFGVIFNSEVNLIYCSSQGNR-EKKEEHYLLE-------------HLSPLYLKHQFGTDNVSDIKNFISQNV-------ANI-KKY---------------------------------------------------ISFHLLTPEQQKAARHALFLDYDDEAFKTITKFLMSQ-------------------QKARVNGTQ
319941583   Sutterella wadsworthensis 3 1 45B      L-IKDARGIVFNAEPNLIYASSRGNQ-LKKNQRYSLS-------------DLKANYRNEIFKTSNIAAITAEIEDVV-------TKL-QQTHRL--------------------------------------------KFFDLLNEHEQDCVRHALFLDDGSEARDAVLELLATQ------------------RRTRVNGTQ
254447899   gamma proteobact. HTCC5015             L-TGRTKKTVFNSEANLIYCSSKGNH-DKGNRVYVIE-------------QLNDKYLKKQFSTSDVNLIKKKIKTTI-------QRFTEGGEKL--------------------------------------------RSFSELSREDQKAFRHALFV---PELKSEVTSLLAVK-------------------NITRVNGTQ
118497352   Francisella novicida U112              ----HKKYGTLNDEANLICVTRGDNK-NKGNRIFCLR-------------DLADNYKLKQFETTDDLEIEKKIADTIWDANKKDFKF-GNY-----------------------------------------------------RSFINLTPQEQKAFRHALFLADENPIKQAVIRAINNR-------------------NRTFVNGTQ

Motifs                                             *_***__________N__________N_______________HNH___}_______________________________________________________________________________________________________________[_
informative positions                              ***********************************------------------------------------------------------------------------------*************************---------------------*********
```

227494853	*Actinomyces coleocanis* DSM 15436#	TSAARKAS--------------------GFE-----------------------------SR--
328956315	*Coriobacterium glomerans* PW2	SHDLRTAA--------------------ELV-----------------------------KC--
227824983	*Acidaminococcus* sp. D21	SSDFRHEF--------------------GLV-----------------------------KS--
303229466	*Veillonella atypica* ACS-134-V-Col7a	VSDFRHNN--------------------NFI-----------------------------KV--
34762592	*Fusobacterium nucleatum* ATCC 49256	ASSFREMF--------------------DFI-----------------------------KV--
374307738	*Filifactor alocis* ATCC 35896	VSDFRKKPL-------------------NYL-----------------------------KS--
320528778	*Solobacterium moorei* F0204	VSEFRNRKD------------------KELP-----------------------------KF--
291520705	*Coprococcus catus* GD-7	VSNFRQTY--------------------ELL-----------------------------KV--
42525843	*Treponema denticola* ATCC 35405	VSMFRNKF--------------------DIV-----------------------------KC--
304438954	*Peptoniphilus duerdenii* ATCC BAA-1640	ASRFRQEF--------------------DII-----------------------------KC--
224543312	*Catenibacterium mitsuokai* DSM 15897	SHEFRVKN--------------------HIY-----------------------------KN--
116628213	*Streptococcus thermophilus* LMD-9	VSQFRKDF--------------------ELY-----------------------------KV--
24379809	*Streptococcus mutans* UA159	VSNFRKEF--------------------ELY-----------------------------KV--
13622193	*Streptococcus pyogenes* SF370	VSDFRKDF--------------------QFY-----------------------------KV--
310286728	*Bifidobacterium bifidum* S17	TKEMHRYL--------------------GFS-----------------------------HK--
366983953	*Oenococcus kitaharae* DSM 17330	TADMRRYV--------------------DIP-----------------------------KN--
422884106	*Streptococcus sanguinis* SK49	ITNFRNTF--------------------HIA------------------------------K--
339625081	*Fructobacillus fructosus* KCTC 3544	TADMRLLV--------------------GIK------------------------------K--
306821691	*Eubacterium yurii* ATCC 43715	VSEFRHKF--------------------ELF------------------------------K--
336394882	*Lactobacillus farciminis* KCTC 3681	STAFRKALSGQDDTYHFKHP--------ELV------------------------------K--
323463801	*Staphylococcus pseudintermedius* ED99	VSEFRKKF--------------------DIP------------------------------K--
389815359	*Planococcus antarcticus* DSM 14505	VSKFRRFS--------------------EIP------------------------------K--
258509199	*Lactobacillus rhamnosus* GG	SHQLREEL--------------------DFP-----------------------------KN--
169823755	*Finegoldia magna* ATCC 29328	ASDLRKDM--------------------NTL-----------------------------KS--
Jnet	227501312	HHHHHHH---
47458868	*Mycoplasma mobile* 163K	TSNLRNQIAFVGIKNNKETEREW-KRPEGFK-----------------------------SI--
284931710	*Mycoplasma gallisepticum* str. F	TSFLRKNFD-----------------DSSYA-----------------------------KK--
71894592	*Mycoplasma synoviae* 53	TSFLRKSMW-------------------RKN-----------------------------EE--
363542550	*Mycoplasma ovipneumoniae* SC01	TKYFRAKPVQKNNGPNEN-------------------LNNNK------------------PE--
384393286	*Mycoplasma canis* PG 14	TSFIRKNM--------------------SYDN----------------------------KL--
238924075	*Eubacterium rectale* ATCC 33656	THQMRCNL--------------------KLD-----------------------------KN--
315149830	*Enterococcus faecalis* TX0012	THTLRKKW--------------------GAD-----------------------------LD--
116627542	*Streptococcus thermophilus* LMD-9	TSQLRRHW--------------------GIE-----------------------------KT--
315659848	*Staphylococcus lugdunensis* M23590	TDYLRKVW--------------------KFK-----------------------------KE--
160915782	*Eubacterium dolichum* DSM 3991	TNAFRRKI--------------------NLK-----------------------------KD--
325677756	*Ruminococcus albus* 8	TSMFRRWW---------------------LN-----------------------------EK--
225377804	*Roseburia inulinivorans* DSM 16841	TSYLRKRW--------------------GLM-----------------------------QK--
336393381	*Lactobacillus coryniformis* KCTC 3535	TSKIRSRW--------------------GFL-----------------------------KV--
310780384	*Ilyobacter polytropus* DSM 2926	TAQLRARW--------------------RLN-----------------------------KV--
301311869	*Bacteroides* sp. 20 3	TDRLKKDW--------------------GIN-----------------------------DVWNKIVLPRFIRL--
385811609	*Ignavibacterium album* JCM 16511	TSELKITW--------------------GLN-----------------------------GVWKDILRPRFKRL--
60683389	*Bacteroides fragilis* NCTC 9343	TDKLREDW--------------------QLI-----------------------------DV-----------M--
319957206	*Nitratifractor salsuginis* DSM 16511	TSKTRSLL--------------------GIK-----------------------------SK--
187250660	*Elusimicrobium minutum* Pei191	TAQLRMAW--------------------GLQ-----------------------------GL--
325972003	*Sphaerochaeta globus* str. Buddy	TSILRKAW--------------------NLQ-----------------------------GI--
296446027	*Methylosinus trichosporium* OB3b	TDRLRRAW--------------------GLQ-----------------------------WM--
347536497	*Flavobacterium branchiophilum* FL-15	VAEFRKIW--------------------GIQ-----------------------------ES--
345885718	*Prevotella* sp. C561	TAKFRKIL--------------------GIQ-----------------------------SV--
282880052	*Prevotella timonensis* CRIS 5C-B1	TAEFRKMW--------------------GLQ-----------------------------SE--
312879015	*Aminomonas paucivorans* DSM 12260	TAFLRKQW--------------------GLH-----------------------------KN--
294086111	*Candidatus Puniceispirillum marinum* IMCC1322	TSLLRGFW--------------------GLN-----------------------------SI--
330822845	*Alicycliphilus denitrificans* K601	TALLRGKF--------------------GLN-----------------------------DV--
344171927	*Ralstonia syzygii* R24	TAMLRAKW--------------------GLN-----------------------------RV--
159042956	*Dinoroseobacter shibae* DFL 12	TEMLRRHW--------------------GLN-----------------------------SL--
83591793	*Rhodospirillum rubrum* ATCC 11170	TGLLRLAW--------------------DITPGPAPRDLLPTPRDALRDDTAARRFLDGLTPPPLAKAVEGAVQ--
288957741	*Azospirillum* sp. B510	TALLRRRW--------------------GID-----------------------------AI--
427429481	*Caenispirillum salinarum* AK4	ISRVQPVN--------------------GSVTHMLRQRLLQRDKNRDYQTHHAEDACLLLLAGPA---
92109262	*Nitrobacter hamburgensis* X14	TSMLRGKW--------------------GLN-----------------------------GL--
148255343	*Bradyrhizobium* sp. BTAi1	TALLRATW--------------------ELN-----------------------------DL--
34557790	*Wolinella succinogenes* DSM 1740	TSVLRYQW--------------------GLE-----------------------------SK--
218563121	*Campylobacter jejuni* NCTC 11168	TSALRHTW--------------------GFS-----------------------------AK--
Jnet	218563121	HHHHHHH---
291276265	*Helicobacter mustelae* 12198	TSTMRSFW--------------------GVQ-----------------------------EK--
222109285	*Acidovorax ebreus* TPSY	TAFLRARW--------------------GLT-----------------------------KV--
365156657	*Bacillus smithii* 7 3 47FAA	TAHLRSRW--------------------DFN-----------------------------KN--
220930482	*Clostridium cellulolyticum* H10	TAQLRSRW--------------------GLN-----------------------------KN--
297182908	uncultured delta proteobact. HF0070 07E19	TGYLRKQW--------------------GLE---
154250555	*Parvibaculum lavamentivorans* DS-1	TAQLRKLW--------------------TLN-----------------------------NI--
218767588	*Neisseria meningitidis* Z2491	TNLLRGFW--------------------GLR-----------------------------KV--
15602992	*Pasteurella multocida* str. Pm70	TALLRSRW--------------------GLI-----------------------------KA--
187736489	*Akkermansia muciniphila* ATCC BAA-835	TAEVRKAW--------------------DVF-----------------------------GV--
315605738	*Actinomyces* sp. oral taxon 180 str. F0310	TACARRAA--------------------HVD-----------------------------KR--
117929158	*Acidothermus cellulolyticus* 11B	TAEARRWL--------------------DISIE---------------------------RL--
189440764	*Bifidobacterium longum* DJO10A	TASARRAA--------------------GIE-----------------------------GK--
403744858	*Alicyclobacillus hesperidum* URH17-3-68	TAYMRARW--------------------GLT-----------------------------KD--
407803669	*Alcanivorax* sp. W11-5	TAELRRRW--------------------GLD-----------------------------TVIPQVRFESG-MPVVDEEGAEI------------------------------TPEEFEKFRLQWEGHRVTREMRTDRR---
423317190	*Bergeyella zoohelcum* ATCC 43767	TDYLRNHW--------------------GLT-----------------------------DKFKLLLKERY-EALLESEKFLEAEYDNYKKDFDSRKKEYEEKEVLFEEQELTREEFIKEYKENYIRYKKNKLIIKGWS---
402847315	*Porphyromonas* sp. oral taxon 279 str. F0450	TARLRSLW--------------------GYG-----------------------------KILHTLNLDRY-DSMGETERV--SREGEATEELHITNWS---
404487228	*Barnesiella intestinihominis* YIT 11860	TSFLRHVW--------------------GWD-----------------------------TVLHDLNFDRY-KKVGLTEVIEV--NHRGSVIRREQIKDWS---
374384763	*Odoribacter laneus* YIT 12061	TAELRHLW--------------------GLN-----------------------------NILQSAPDITFPLPVSATENHREYYVITNEQNEVIRLFPKQGETPRTEKGELLLTGEVERKVFRCKGMQEFQTDVSDGKYWRRIKLSSSVTWSPLFAPKPISADGQIVLKGRIEKGVFVCNQLKQKLKTGLPDGSYWISL
384109266	*Treponema* sp. JC4	TKLLRDKW--------------------EMD-----------------------------SIL---CRKFTEKEVALLGLK---
402849997	*Rhodovulum* sp. PH10	TGLLRARW--------------------GLN-----------------------------SIL--SDSN---
Jnet	331001027	HHHHHHHH--HHHHHHHHHHHHH----EEEEEE-------------------------HHHHHHHHHHHHH--HEE--
331001027	*Parasutterella excrementihominis* YIT 11859	AWFVRSIF--SKVRQALAAWTQETGNELIFDAISVPA---------------------ADSSEMRKRFAEYRPEFRK---
34557932	*Wolinella succinogenes* DSM 1740	KYLAKKIQ--EKLTKML------PNKHLSFEFILADA---------------------TEVSELRRQYARQNPLLAK---
54296138	*Legionella pneumophila* str. Paris	KFLGKQIM--EFLSTLA----DSKQLQLEFSIKQITA---------------------EEVHDHRELLSKQEPKLVK---
319941583	*Sutterella wadsworthensis* 3 1 45B	IWMIKNLA--NKIREELQNWCKTTNNRLHFQAAATNV---------------------SDAKNLRLKLAQNQPDFEK---
254447899	gamma proteobact. HTCC5015	AWLAKKIA--SLLAEHL----DKQGRDYTLSAHQIDP---------------------WSVSKQRKMLASAEPIWAK---
118497352	*Francisella novicida* U112	RYFAEVLA--NNIYLRA-KKENLNTDKISFDYFGIPTIGNG-----------------RGIAEIRQLYEKVDSDIQAYAK--
Motifs		____R____
informative positions		********--

227494853	*Actinomyces coleocanis* DSM 15436#	---VNFIGGNGKTRLDRRHHAMDAATVAMLRNSVA----KTLVLRG--------
328956315	*Coriobacterium glomerans* PW2	--REANDFHHAHDAFLACRVGLFIQ----KR--HPC--------
227824983	*Acidaminococcus* sp. D21	--RNINDLHHAKDAFLAIVTGNVYH----ER--FNR--------
303229466	*Veillonella atypica* ACS-134-V-Col7a	--RSLNHHHHAKDAYLNIVVGNVYH----EK--FTR--------
34762592	*Fusobacterium nucleatum* ATCC 49256	--RELNDTHHAKDAYLNIVAGNVYN----TK--FTE--------
374307738	*Filifactor alocis* ATCC 35896	--RRVNDYHHAKDAYLNIVVGNVYN----KK--FTS--------
320528778	*Solobacterium moorei* F0204	--REINDLHHAKDAYLNIVVGNVYD----TK--FTE--------
291520705	*Coprococcus catus* GD-7	--REMNDLHHAKDAYLNIVVGNAYF----VK--FTK--------
42525843	*Treponema denticola* ATCC 35405	--REINDFHHAHDAYLNIVVGNVYN----TK--FTN--------
304438954	*Peptoniphilus duerdenii* ATCC BAA-1640	--RTVNDLHHMHDAYLNIVVGNVYN----TK--FTK--------
224543312	*Catenibacterium mitsuokai* DSM 15897	--RDINDYHHAHDAYIVALIGGFMR----DR--YPN--------
116628213	*Streptococcus thermophilus* LMD-9	--REINDFHHAHDAYLNAVVASALL----KK--YPK--------
24379809	*Streptococcus mutans* UA159	--REINDYHHAHDAYLNAVIGKALL----GV--YPQ--------
13622193	*Streptococcus pyogenes* SF370	--REINNYHHAHDAYLNAVVGTALI----KK--YPK--------
310286728	*Bifidobacterium bifidum* S17	---NRDINDYHHAQDALCVGIAGQFAA----NRGFFAD------GE
366983953	*Oenococcus kitaharae* DSM 17330	--RDINDYHHAFDALLFSTVGQYTE----NSGLMKK------GQ
422884106	*Streptococcus sanguinis* SK49	---IRELNDYHHAHDAYLAVVVGQTLL----KVYPKLA------PE
339625081	*Fructobacillus fructosus* KCTC 3544	---HREINSFHHAFDALLITAAGQYMQ----NRYPDRD--------
306821691	*Eubacterium yurii* ATCC 43715	---SRLINDFHHANDAYLNIVVGNSY--------------------
336394882	*Lactobacillus farciminis* KCTC 3681	---NRNVNDFHHAQDAYLASFLGTYRL----RRFPTNE------ML
323463801	*Staphylococcus pseudintermedius* ED99	---IRQMNDAHHAIDAYLNGVVYHGAQ----LAYPNVD--------
389815359	*Planococcus antarcticus* DSM 14505	---IRDYNNKHHAMDALFAAALIQSIL----GKYGKN---------
258509199	*Lactobacillus rhamnosus* GG	--RDVNHYHHAFDAFLAARIGTYLL----KR--YPK--------
169823755	*Finegoldia magna* ATCC 29328	--REVNDLHHAHDAFLNIVAGDVWN----RE--FTS--------
Jnet	227501312	---HHHHHHHHHHHHH-HHHH----HH--H----------
47458868	*Mycoplasma mobile* 163K	--NSNDFLIREEGKNDVKDDVLIKDRSFNGHHAEDAYFITIISQYFR----SFKRIER--------
284931710	*Mycoplasma gallisepticum* str. F	--DRDKNIHHAVDASIISIFSNETK----TL--FNQ--------
71894592	*Mycoplasma synoviae* 53	--QVYRFNFWKKDRDQFFHHAVDASIIAIFSLLTK----TLYNKLR--------
363542550	*Mycoplasma ovipneumoniae* SC01	--KIE------KNRENNEHHAVDAAIVAIIGNKNP----QIANLLT--------
384393286	*Mycoplasma canis* PG 14	--RL-------KDRSDFSHHAYDAAIIALFSNKTK----TLYNLID--------
238924075	*Eubacterium rectale* ATCC 33656	--RDESYSHHAVDAMLIGYSELGYE----AYHKLQG--------
315149830	*Enterococcus faecalis* TX0012	---KTRETHHHHAVDATLCAVTSFVKV----SRYHYAV------KE
116627542	*Streptococcus thermophilus* LMD-9	---RDTYHHHAVDALIIAASSQLNL--------WKK--------
315659848	*Staphylococcus lugdunensis* M23590	--RNHGYKHHAEDALIIANADFLFK----ENKKLKA--------
160915782	*Eubacterium dolichum* DSM 3991	--RDEDYGHHAIDALIIASMPKMRL--------LST--------
325677756	*Ruminococcus albus* 8	---TWGRYDKA-ELKKLTYLDHAADAIIIANCRPEYVVLAGEKLKLNK--------
225377804	*Roseburia inulinivorans* DSM 16841	--DRSTDRHHAMDAVVIACCTDGMI----HK--ISR--------
336393381	*Lactobacillus coryniformis* KCTC 3535	--REDGDLHHAIDATVIAAITPKFI----QQ--VTK--------
310780384	*Ilyobacter polytropus* DSM 2926	--RENGDLHHALDAAVVAVTDQKAI----NN--ISN--------
301311869	*Bacteroides* sp. 20 3	--NELTESTRFTSINTNNTMIPSMPLELQKGFNK----KRIDHRHHAMDAIIIACANRNIV----NY--LNN--------
385811609	*Ignavibacterium album* JCM 16511	--ESII-NKKLIFQDEDDPNKYHFDLSINPQLDKEGL-KRLDHRHHALDATIIAATTREHV----RY--LNS--------
60683389	*Bacteroides fragilis* NCTC 9343	--KELNWEKYKALGLVEYFEDRDGRQIGRIKDWTKRNDHRHHAMDALTVAFTKDVFI----QY--FNN--------
319957206	*Nitratifractor salsuginis* DSM 16511	--SRETNFHHAEDALILSTLTRGWQ----NR--LHR--------
187250660	*Elusimicrobium minutum* Pei191	--MIPFAKQLITEKESESFNKDVNSNKKIRLDNRHHALDAIVIAYASRGYG----NL--LNK--------
325972003	*Sphaerochaeta globus* str. Buddy	---DDLLGSRHWSKDADTSPTMRKNRDDNRHHGLDAIVALYCSRSLV----QM--INT--------
296446027	*Methylosinus trichosporium* OB3b	---KKSTKGDRIPDDRHHALDAIVIAATTESLL----QR-------------
347536497	*Flavobacterium branchiophilum* FL-15	--FIDENGMKHYK-VKDRSKHTHHTIDAITIACMTKEKY----DV--LAH--------
345885718	*Prevotella* sp. C561	---DE-KKDRSLHSHHAIDATTLTIIPVSAKRD--RM--LEL--------
282880052	*Prevotella timonensis* CRIS 5C-B1	---YE-KKCRDNHSHHCMDAITIACIGKREY----DL--MAE--------
312879015	*Aminomonas paucivorans* DSM 12260	--RKNGDLHHALDAAVLAVASRSFV----YR--LSS--------
294086111	*Candidatus Puniceispirillum marinum* IMCC1322	--LRGHNTDDGTPAKKSRDDHRHHAIDAIVVGMTSRGLL----QK--VSK--------
330822845	*Alicycliphilus denitrificans* K601	---LGLDGEKNRNDHRHHAVDACVIGVTDQGLM----QR--FAT--------
344171927	*Ralstonia syzygii* R24	--LDGVMEEQGRPAVKNRDDHRHHAIDAVVIGATDRAML----QQ--VAT--------
159042956	*Dinoroseobacter shibae* DFL 12	--LSDAGRGAVKAKNRTDHRHHAIDAAVIAATDPGLL----NR--ISR--------
83591793	*Rhodospirillum rubrum* ATCC 11170	--ARLAALGRSRVADAGLADALGLTLASLGGGGKNRADHRHHFIDAAMIAVTTRGLI----NQ--INQ--------
288957741	*Azospirillum* sp. B510	---LAEADGPPPEVPAETLDPSPAEKNRADHRHHALDAVVIGCIDRSMV----QRVQLAA--------
427429481	*Caenispirillum salinarum* AK4	---VVQAFNTEAAQHGADAPDDRPVDLMPTSDAYHQQRRARALGRVPLATV----DAAL-----------
92109262	*Nitrobacter hamburgensis* X14	---LPSDNYAGVQDKAEEFLASTDDMEFSGVKNRADHRHHAIDGLVTALTDRSLL-------------------
148255343	*Bradyrhizobium* sp. BTAi1	---LPGSDDRA-AKSRKDHRHHAIDALVAALTDQALL----RR-------------
34557790	*Wolinella succinogenes* DSM 1740	--DRESHTHHAVDAIIIAFSTQGMV----QK--LSE--------
218563121	*Campylobacter jejuni* NCTC 11168	--DRNNHLHHAIDAVIIAYANNSIV----KA--FSD--------
Jnet	218563121	---HHHHHHHHHHH-HHHH----HH--HHH--------
291276265	*Helicobacter mustelae* 12198	--NRDHHLHHAQDAIIIACIEPSMI----QK--YTT--------
222109285	*Acidovorax ebreus* TPSY	--RGDSDRHHALDAAVVAACTHGMV----KA--LAD--------
365156657	*Bacillus smithii* 7 3 47FAA	--REESDLHHAVDAVIVACATQGMI----KK--ITE--------
220930482	*Clostridium cellulolyticum* H10	--REESDLHHALDAAVIACADRKII----KE--ITN--------
297182908	uncultured delta proteobact. HF0070 07E19	---KDRDKHTHHAVDAIVVACTTPAIV----QQ--VTL--------
154250555	*Parvibaculum lavamentivorans* DS-1	---LADDGEKTRADHRHHAIDALTVACTHPGMT----NK--LSR--------
218767588	*Neisseria meningitidis* Z2491	--RAENDRHHALDAVVVACSTVAMQ----QK--ITR--------
15602992	*Pasteurella multocida* str. Pm70	--RENNNRHHALDAIVVACATPSMQ----QK--ITR--------
187736489	*Akkermansia muciniphila* ATCC BAA-835	---FKELCPEAADPDSGKILKENLRSLTHLHHALDACVLGLIPYIIP----AH-------------
315605738	*Actinomyces* sp. oral taxon 180 str. F0310	--VRLIRLKGDDGHHKNRFDRRNHAMDALVIALMTPAIA----RTIAVRE--------
117929158	*Acidothermus cellulolyticus* 11B	---FSRVAIFAQSTSTKRLDRRHHAVDAVVLTTLTPGVA----KT--LAD--------
189440764	*Bifidobacterium longum* DJO10A	---IHFIGQQSKTRLDRRHHAVDASVIAMMNTAAA----QT--LME--------
403744858	*Alicyclobacillus hesperidum* URH17-3-68	--RDAGDKHHAADAVVVACIGDSLI----QR--VTK--------
407803669	*Alcanivorax* sp. W11-5	--PDKRIDHRHHLVDAIVTALTSRSLY----QQ--YAK--------
423317190	*Bergeyella zoohelcum* ATCC 43767	--KRIDHRHHAIDALIVACTEPAHI----KR--LND--------
402847315	*Porphyromonas* sp. oral taxon 279 str. F0450	--KRMDHRHHAIDALVVACTRQSYI----QR--LNR--------
404487228	*Barnesiella intestinihominis* YIT 11860	--KRFDHRHHAIDALTIACTKQAYI----QR--LNN--------
374384763	*Odoribacter laneus* YIT 12061	PVISQTFKEGESVNNSKLTSQQVQLFGRVREGIFRCHNYQCPASGADGNFWCTLDTDTAQPAFTPIKNAPPGVGGGQIILTGDVDDKGIFHADDDLHYELPASLPKGKYYGIFTVESCDPTLIPIELSAPKTSKGENLIEGNIWVDEHTGEVRFDPKKNREDQRHHAIDAIVIALSSQSLF----QR--LST--------
384109266	*Treponema* sp. JC4	---PEQIGNYKKNRFDHRHHAIDAVVIGLTDRSMV----QK--LAT--------
402849997	*Rhodovulum* sp. PH10	--FKNRSDHRHHAVDAVVIGVLTRGMI----QR--IAH--------
Jnet	331001027	---HHHHHHHHHHHHH----------------------
331001027	*Parasutterella excrementihominis* YIT 11859	---PKVQPVASHSIDAMCIYLAACSDPFK-----------------
34557932	*Wolinella succinogenes* DSM 1740	---AEKQAPSSHAIDAVMAFVARYQKVFKDGTPP------------
54296138	*Legionella pneumophila* str. Paris	---SRQQSFPSHAIDATLTMSIGL----------------------
319941583	*Sutterella wadsworthensis* 3 1 45B	---PDIQPIASHSIDALCSFAVGSAD--------------------
254447899	gamma proteobact. HTCC5015	---KDPQPAASHVVDAVCTFLEALE---------------------
118497352	*Francisella novicida* U112	--GDKPQASYSHLIDAMLAFCIAADEHRNDGSIGLEID--------
Motifs		____H__D_RuvC_III______________________
informative positions		--********************----------------------

227494853	*Actinomyces coleocanis* DSM 15436#	--------NIRASERAIG----------------------AAETWKSFRGENVADRQI----------------FESW---SENMRVLVEKFNL-------------------------------------ALYNDEVSI---FSSLRLQL-------------GNGKAHDDTI---------------------------
328956315	*Coriobacterium glomerans* PW2	-------VYENPIGLSQVVRNYVRQ---------------QADIFKRCRTIPGSSGFIVNSFMTSGFDKETGEIFKDDW---DAEAEVEGIRRSL--------------------------------------NFRQCF---ISRMPFED-------------HGVFWDATIYS-------------------------
227824983	*Acidaminococcus* sp. D21	---------RWFMVNQPY----------------------SVKTKTLFTHSIKNGN-------------------FVAW---NGEEDLGRIV-KM----------------------------------LKQNKNTIH--FTRFSFDR-------------KEGLFDIQPL-------------------------
303229466	*Veillonella atypica* ACS-134-V-Col7a	-------NFRLFFKKNGANR--------------------TYNLAKMFNYDVICTNAQD----------------GKAW---DVKTSMNTVK-KM-------------------------------------MASNDVR---VTRRLLEQ-------------SGALADATIYK--------------------------A
34762592	*Fusobacterium nucleatum* ATCC 49256	-------KYRYLQEIKE-----------------------NYDVKKIYNYDI-----------------------KNVW---DKENSLEIVKKNM------------------------------------EKNTVN---ITRFIKEE-------------KGELFNLNP--------------------------IK
374307738	*Filifactor alocis* ATCC 35896	-------NPIQMWKKNRDT---------------------NYSLNKVFEHDVVING-------------------EVIW---EKCYHEDTN-TY-----------------------DGGTLDRIRKIVERDNIL---YTEYAYCE-------------KGELFNATI---------------------------
320528778	*Solobacterium moorei* F0204	-------KFFNNIRNE------------------------NYSLKRVFDFSV-----------------------PGAW---DAKGSTFNTIKKY------------------------------------AKEMNPI---IAFAPYEV-------------KGELFDQQI---------------------------
291520705	*Coprococcus catus* GD-7	-------NAAWFIRNNFGR---------------------SYNLKRMFEFDIERSG-------------------EIAW---KAGNKGSIVTVKK------------------------------------VMQKNNIL---VTRKAYEV-------------KGGLFDQQI---------------------------
42525843	*Treponema denticola* ATCC 35405	-------NPWNFIKEKRDNPKIAD----------------TYNYYKVFDYDVKRNN-------------------ITAW---EKGKTIITVK-DM-------------------------------------LKRNTPI---YTRQAACK-------------KGELFNQTI---------------------------
304438954	*Peptoniphilus duerdenii* ATCC BAA-1640	-------NPLNFIKDKDNVR--------------------SYNLENMFKYDVVRGS-------------------YTAWIADDSEGNVKAATIKK-----------------------------VKRELEGKNYR--FTRMSYIG-------------TGGLYDQNL---------------------------
224543312	*Catenibacterium mitsuokai* DSM 15897	------------MHDSKAV---------------------YSEYMKMFRKNKNDQKRWKDGFVINSMNYPYEVDGKLIWN--PDLINEIKKC------------------------------------FYYKDCY---CTTKLDQK-------------SGQLFNLTV--------------------------L
116628213	*Streptococcus thermophilus* LMD-9	-----LEPEFVYGDYPKYN-------SFRERKSATEKVYFYSNIMNIFKKSISLADGRVIERPLIEVNEETG---ESVW---NKESDLATVR-RV-----------------------------------LSYPQVN---VVKKVEEQ-----NHGLDRGKPKGLFNANLSS----------------------
24379809	*Streptococcus mutans* UA159	-----LEPEFVYGDYPHFH-------GHKE-NKATAKKFFYSNIMNFFKKD------DVR--------TDKNG---EIIW---KKDEHISNIK-KV-----------------------------------LSYPQVN---IVKKVEEQ-------------TGGFSKESI---------------------------
13622193	*Streptococcus pyogenes* SF370	-----LESEFVYGDYKVYDVRKMIAKSEQEIGKATAKYFFYSNIMNFFKTEITLANGEIRKRPLIETNGETG---EIVW---DKGRDFATVR-KV-----------------------------------LSMPQVN---IVKKTEVQ-------------TGGFSKESI---------------------------
310286728	*Bifidobacterium bifidum* S17	VSDGAQNSYNQYLRDYLRGYREKLSAE-------------DRKQGRAFGFIVGSMRSQDEQKRVNPRTG------EVVW---SEEDKDYLRK-VM-------------------------------------NYRKML---VTQKVGDD-------------FGALYDETRYA-------------------------
366983953	*Oenococcus kitaharae* DSM 17330	LSDSAGNQYNRYIKEWIHAARLNA-----------------QSQRVNPFGFVVGSMRNAAPGK-LNPETG-------EITP----EENADWSIA-DL-------------------------------------DYLHKV---MNFRKITVTRRLKDQ--------KGQLYDESRYP-------------------------
422884106	*Streptococcus sanguinis* SK49	LIYGHHAHFNRHEENKATLRKHLYSN-----------------------IMRFFNNPDSK------VS------KDIWD--CNRDLPIIKD-VI------------------------------------YNSQIN---FVKRTMIK-------------KGAFYNQNPVG-------------------------
339625081	*Fructobacillus fructosus* KCTC 3544	-STNVYNEFDRYTNDYLKNLRQLSSRD--------------EVRRLKSFGFVVGTMRKGNED-------------------W---SEENTSYLRK-VM-------------------------------------MFKNIL---TTKKTEKD-------------RGPLNKETIFS-------------------------
306821691	*Eubacterium yurii* ATCC 43715	-----FVKFTRNPANFIKDARKNPDN----------------------PVYKYHMDRFFERDVK-----SKSEVAWIGQSEG---NSGTIVIVKK-TM-------------------------------------AKNSPL---ITKKVEEG-------------HGSITKETIVGVKEIKFGRNKVEK-------------
336394882	*Lactobacillus farciminis* KCTC 3681	LMNGEYNKFYGQVKELYSKKKKLPDS----------------------RKNGFIISPLVNGTTQ--YDRNTG------EIIW---NVGFRDKILK-IF-------------------------------------NYHQCN---VTRKTEIK-------------TGQFYDQTIYS-------------------------
323463801	*Staphylococcus pseudintermedius* ED99	-----LFDFNFKWEKVREKWKALGEFN-------------TKQKSREL-FFFKKLEKMEVS----------------Q---GERLISKIKL-DM-------------------------------------NHFKIN---YSRKLANI-------------PQQFYNQTAVS-------------------------
389815359	*Planococcus antarcticus* DSM 14505	-----FLAFDLSKKDRQKQWRSVKGSN-------------------KEF-FLFKNFGNLRLQ---SPVTG------EEV-----SGVEYMKH-VY-------------------------------------FELPWQ---TTKMTQTG-------------DGMFYKESIFS-------------------------
258509199	*Lactobacillus rhamnosus* GG	-----LAPFFTYGEFAKV----------------------DVKKFREFNFIGALTHAKKNIIAKDTG--------EIVW---DKERDIRELD-RI-------------------------------------YNFKRML---ITHEVYFE-------------TADLFKQTIYA-------------------------
169823755	*Finegoldia magna* ATCC 29328	-------NPINYVKENREGDKV------------------KYSLSKDFTRPRKSKG-------------------KVIWTPEKGRKLIVDTL--NKPSVL---ISNESHVK-------------KGELFNATIAG-------------------------
Jnet	227501312	---------HHHH---EE--------HHHHH--E---EEEEEEE---
47458868	*Mycoplasma mobile* 163K	-------LNVNYRKE-------------------------TRELDDLEKNNIKFKE-------------------KASF---DNFLLINALD-EL--------------------------------------NEKLNQMR---FSRMVITK-------------KNTQLFNETLYS-----------------------GKY
284931710	*Mycoplasma gallisepticum* str. F	--------LTQFADYKLFKN--------------------TDGSWKKIDPKTGVVTEVT---------------DENW---KQIRVRNQVS-EI------------------------AKVIEKYIQDSNIERKAR---YSRKIENK-------------TNISLFNDTVYSAKKVGYEDQIKRKNLKTLDIHESAK
71894592	*Mycoplasma synoviae* 53	-------VYESYDVQRREDG--------------------VYLINKETGEVKKAD-----------------------KDYWKDQHNFLKIRENAIEI-----------------------------KNVLNNVDFQNQVR---YSRKANTK-------------LNTQLFNETLYG------------------------VKEF
363542550	*Mycoplasma ovipneumoniae* SC01	-------LADNKTDKKFLLHDENYKE-----------------NIETGELVKIP--------------------KFE---VDKLAKVEDLKKII---------------------------------QEKYEEAKKHTAIK---FSRKTRTI-------------LNGGLSDETLYG----------------FKYDEKEDK
384393286	*Mycoplasma canis* PG 14	-------PSLNG--IISKRSEGYWVIE-----------------DRYTGEIKELK-----------------------KEDWTSIKNNVQARKIAKEI---------------------------EEYLIDLD--DEVF---FSRKTRKK-------------TNRQLYNETIYG-----------------IATKTDEDG
238924075	*Eubacterium rectale* ATCC 33656	-------EFIDFETGEIL--------------------RKDMWDENMSDEVYADYLY------------------GKKW---ANIRNEVVKA------------------------------------EKNVK---YWHYVMRK-------------SNRGLCNQTI----------------------RGTRE
315149830	*Enterococcus faecalis* TX0012	ETGEKVMREIDFETGEIVNEMS-------------------YWEFKKSKKYERKTY-----------------QVKW---PNF-----RE-QL-------------------------------KPVNLHPRIK---FSHQVDRK-------------ANRKLSDATIYS---------VREKTEVKTLKSGK
116627542	*Streptococcus thermophilus* LMD-9	---QKNTLVSYSEDQ-------------------------LLDIETGELISDDEYKESVF--------------KAPY---QHFVDTLKSK-EF-------------------------------------EDSIL---FSYQVDSK-------------FNRKISDATI---------------------YATRQAKVG
315659848	*Staphylococcus lugdunensis* M23590	-------VNSVLEKPEIE----------------------SKQLDIQVDSEDNYS----------------------EMFIIPKQVQ-DI-------------------------------------KDFRNFK---YSRHVPDK-------------PNRQLINDTL--------------------------Y
160915782	*Eubacterium dolichum* DSM 3991	-------IFSRYKIEDIY----------------------DESTGEVFSSGDDSMYY-----------------DDRY---FAFIASLKAI-------------------------------------KVRK---FSHKIDTK-------------PNRSVADETI---------------------------
325677756	*Ruminococcus albus* 8	---------MYHQAGKRITPEYEQSKKACIDNLYKLFRMDRRTAEKLLSGHGRLTPIIPNLSEEVDKRLWDKNIYEQFW---KDDKDKKSCE-EL-----------------YRENVASLYKGDPKFASSLSMPV---ISLKPDHK-------------YRGTITGEEA--------------------------
325377804	*Roseburia inulinivorans* DSM 16841	--------YMQGRELAYSRNFKFPDEETGEI-------LNRDNFTREQWDEKFGVKV-----------------PLPW---NSFRDELDIRLLNEDPKNFLLTHADVQRELDYPGWMYGEEESPIEEGRYINYIRPLF---VSRMPNHK-------------VTGSAHDATI-------------------------
336393381	*Lactobacillus coryniformis* KCTC 3535	--------YSQHQEVKN-----------------------NQALWHDAEIKDAEYAAEAQRMDADLFNKIFNGF-PLPW---PEFLDELLARISD------------NPVEMMKSRSWNTYTPIEIAKLKPVF---VVRLANHK-------------ISGPAHLDTI--------------------RSAKLF
310780384	*Ilyobacter polytropus* DSM 2926	--------ISRYKELKNCKDVIPSIEYHA-----------DEETGEVYFEEVKDTRF-----------------PMPW---SGFDLELQKRLESENPREEFYN---L--LSDKRYLGWFNYEE-----GFIEKLRPVF---VSRMPNRG-------------VKGQAHQETI------------------------
301311869	*Bacteroides* sp. 20 3	--------VSASKNTKIT----------------------RRDLQTLLCHKDKTDNNGNYKWVI-----------DKPW---ETFTQDTLTALQK-----------------------ITVSFKQNLRVINKTTNH--YQHYENGKKIVSNQSKGDSWAIRKSMHKETV--------------HGEVNLRMIKT
385811609	*Ignavibacterium album* JCM 16511	--------LNAADNDE-E----------------------KREYFLSLCNHKIRD--------F-----------KLPW---ENFTSEVKSKLLS-----------CVVSYKESKPILSDPFNKYLKWEYKNGKWQKVFA---IQIKNDRW-------------KAVRRSMFKEPI-GTVWIKKIKEVSLKEAIKIQAIWEEV
60683389	*Bacteroides fragilis* NCTC 9343	--------KNASLDPNAN----------------------EHAIKNKYFQNGRAIA-------------------PMPL---REFRAEAKKHLEN-------------------------TLISIKAKNKVITGNINKTRKKGG---VNKNMQQT-------------PRGQLHLETI----------YGSGKQYLTKEEKVNASF
319957206	*Nitratifractor salsuginis* DSM 16511	------MLRDNYGKSEAE----------------------LKELWKKYMPHIEGLTLADYI--------------DEAF---RRFMSKGEES-LF------------------------------YRDMFDTIRS---ISYWVDKK-------------PLSASSHKETV--------------------YSSRHEVPT
187250660	*Elusimicrobium minutum* Pei191	--MAGKDYKINYSERNWLSKILLPPN-----------NIVW---ENIDADLESF-ES------------------------------------SVKTALKNAF---ISVKHDHS-------------DNGELVKGTM-----------------------YKIFY
325972003	*Sphaerochaeta globus* str. Buddy	--MSEQGKRAVEIEA----------------------MIPI---PGYASEPNLSFEA-------------------------QRELFRKKILEFMDLHAF---VSMKTDND-------------ANGALLKDTV--------------------------Y
296446027	*Methylosinus trichosporium* OB3b	--ATREVQEIEDKGLHYDLVKNV--------------TPPW---PGFREQAVEA-------------------------------------VEKVF---VARAERRR-------------ARGKAHDATI-------------------------
347536497	*Flavobacterium branchiophilum* FL-15	--AWTLEDQQNK---KEARSIIEA-------------SKPW---KTFKEDLLK-------------------------------------IEEEILV---SHYTPDNVK-------KQAKKIVRVRGKKQFVAEVERDVNGKA---VPKKASGKTIYK
345885718	*Prevotella* sp. C561	--FAKIEEINKMLSFSGSEDRTGL-------------IQEL---EGLKNKLQMEVKVCRIGHNVSEIGTF------------------------INDNIIV---NHHIKNQA----------LTPVRRRLRKKGY--------------------VGGVDN--
282880052	*Prevotella timonensis* CRIS 5C-B1	--YYRMEETFK----QGRGSKPKF-------------SKPW---ATFTEDVLN-------------------------------------IYKNLLV---VHDTPNNM-----------PKHTKKYVQT-------------------SIGK----
312879015	*Aminomonas paucivorans* DSM 12260	--HNAAWGELPRGREAENGF-----------------SLPY---PAFRSEVLARLCP----------------TREEILLRLDQGGVGYDEAFRNGLRPVF---VSRAPSRR-------------LRGKAHMETL-------------------------
294086111	*Candidatus Puniceispirillum marinum* IMCC1322	--AARRSEDLDLTRLFEGR------------------IDPW---DGFRDEVKKH-------------------------------------IDAII---VSHRPRKK-------------SQGALHNDTA-------------------------
330822845	*Alicycliphilus denitrificans* K601	--ASAQARGDGLTRLVDGM------------------PMPW---PTYRDHVERA-------------------------------------VRHIW---VSHRPDHG-------------FEGAMMEETS-------------------------
344171927	*Ralstonia syzygii* R24	--LAARAREQDAERLIGDM------------------PTPW---PNFLEDVRAA-------------------------------------VARCV---VSHKPDHG-------------PEGGLHNDTA-------------------YGIVAGPF
159042956	*Dinoroseobacter shibae* DFL 12	--AAGQGEAAGQSAELIARDT----------------PPPW---EGFRDDLRRK-------------------------------------LDRII---VSHRADHGRIDHAARKQGRDSTAGQLHQETA--------------------------
83591793	*Rhodospirillum rubrum* ATCC 11170	--ASGAGRILDLRKWPRTNF-----------------EPPY---PTFRAEVMKQ-------------------------------------WDHIH---PSIRPAHR-------------DGGSLHAATV-------------------------
288957741	*Azospirillum* sp. B510	--ASAEREAAAREDNIRRVLEGFK-------------EEPW---DGFRAELERR-------------------------------------ARTIV---VSHRPEHG-------------IGGALHKETA-------------------------
427429481	*Caenispirillum salinarum* AK4	--ADIVMPESDRQDPETG-------------------RVHW---RLTRAGRGLKRRIDDLTRNC---------------------------VILSR---PRRPSETG-------------TPGALHNATH-------------YGRREITVDGRTDT
92109262	*Nitrobacter hamburgensis* X14	--WKMANAYDEEHEKFVI-------------------EPPW---PTMRDDLKAA-------------------------------------LEKMV---VSHKPDHG-------------IEGKLHEDSA-------------------------
148255343	*Bradyrhizobium* sp. BTAi1	--MANAHDDTRRKIEV---------------------LLPW---PTFRIDLETR-------------------------------------LKAML---VSHKPDHG-------------LQARLHEDTA-------------------------
34557790	*Wolinella succinogenes* DSM 1740	--YYRFKETHREKERPKL-------------------AVPL---ANFRDAVEEATRI----------------------ENTETVEKGVEVKRLL---ISRPPRAR-------------VTGQAHEQTA-------------------------
218563121	*Campylobacter jejuni* NCTC 11168	--------FKKEQESNSAEL--------------------YAKKISELDYKNKRKF-------------------FEPF---SGFRQKVLDK-------------------------------------IDEIF---VSKPERKK-------------PSGALHEETF-------------------------
Jnet	218563121	--------HHHHH--H--EE---EEE-------------------HHHHHHHH--------------------------
291276265	*Helicobacter mustelae* 12198	--------YLKDKETHRLK---------------------SHQKAQILREGDHKL--------------------SLRW-PMSNFKDKIQES-------------------------------------IQNII---PSHHVSHK-------------VTGELHQETV-------------------------
222109285	*Acidovorax ebreus* TPSY	--------YSRKKEISFLQEGFP-----------------DPETGEILNPAAFDRARQHF---------------PEPW---THFAHELKARLFT-------------DDLAALREDMQRLGSYTTEDLGRLRTLF---VSRAPQRR-------------SGGAVHKETI-------------------------
365156657	*Bacillus smithii* 7 3 47FAA	------------------------------FYKAREQNKESAKKKEPIF--------------------------PQPW---PHFADELKARLSK-----------------FPQESIEAFALGNYDRKKLESLRPVF---VSRMPKRS-------------VTGAAHQETL--------------------RRC
220930482	*Clostridium cellulolyticum* H10	--YYNERENHNYKVKY---------------------PLPW---HSFRQDLMET-------------------------------------LAGVF---ISRAPRRK-------------ITGPAHDETI-------------------------
297182908	uncultured delta proteobact. HF0070 07E19	--YHQDIRRYKKLGEKR--------------------PTPW---PETFRQDVLDV-------------------------------------EEEIF---ITRQPKKV-------------SGGIQTKDTL-------------------------
154250555	*Parvibaculum lavamentivorans* DS-1	--YWQLRDDPRAEKPAL--------------------TPPW---DTIRADAEKA-------------------------------------VSEIV---VSHRVRKK-------------VSGPLHKETT-------------------------
218767588	*Neisseria meningitidis* Z2491	--FVRYKEMNAFDGKT----IDKETGEVLHQKTHF--PQPW---EFFAQEVMIRVFGKPDGKPEFEEADTPEKL----RTLLAEKLSSRPEAVHEYVTPLF---VSHRPNRK-------------MSGQGHMETV-------------------------
15602992	*Pasteurella multocida* str. Pm70	--FIRFKEVHPYKIENRYEMVDQESGEIISP--HF--PEPW---AYFRQEVNIRVFD---NHPD-------------TVLKEMLPDRPQANHQFVQPLF---VSRAPTRK-------------MSGQGHMETI-------------------------
187736489	*Akkermansia muciniphila* ATCC BAA-835	--HNGLLRRVLAMRRI---------------------PEKL---IPQVRPVAN---QRHYVLND--------------DGRMMLRDL------------------------
315605738	*Actinomyces* sp. oral taxon 180 str. F0310	--------DRREAQQLTR----------------------AFESWKNFLGSEERMQDR-----------------WESW---IGDVEYACDRLNE--------------------------------LIDADKIPV---TENLRLR-------------NSGKLHADQP-------------------ESLKKARRGSK
117929158	*Acidothermus cellulolyticus* 11B	------------ARSRRV----------------------SAEFWRRPSDVNRHSTEEPQSPA------------YRQWKESCSGLGDLLIS-------------------------------------TAARDSIA---VAAPLRLR-------------PTGALHEETL------------------------
189440764	*Bifidobacterium longum* DJO10A	--------RESLRESQRLIGLMP-----------------GERSWKEYPYEGTSRYES-----------------FHLWL--DNMDVLLELLNDA-----------------------------------LDNDRIAV---MQSQRYVL-------------GNSIAHDATIHPL------------------------E
403744858	*Alicyclobacillus hesperidum* URH17-3-68	--------YDKFKRNALADRNRYVQQVSKSEG--ITQYVDKETGEVFTWESFDERKFL-------------PNEPLEPWP---FFRDELLARLSDDPSKNIRAIGLLTYSE---TEQ--------------IDPIFVSR--MPTRKV---------------------TGAAHKETIRSP----------RI
407803669	*Alcanivorax* sp. W11-5	--------AWKVADEKQRHGR-----------------------------------VDVKVEL----------------PMPIL---TIRDIA--------------------LEA---------------VRSVRISH--KPDRYP------------------------------DGRFFEATAYGI----------AQ
423317190	*Bergeyella zoohelcum* ATCC 43767	--------LNKVLQDWLVEHKSEFMPNFEGSN--SELLEEILSLPENERTEIFTQIEK-----------FRAIEMPWK---GFPEQV----------------------EQK----------------LKEIISH--KPKDKLLQLYNKAGDRQIKL---------------RGQLHEGTLYGI----------SQ
402847315	*Porphyromonas* sp. oral taxon 279 str. F0450	--------LSSEFGREDKKKE------------DQEAQEQQATETGRLSNLERWLT-------------QRPHF---SVRTV-----------------------SDK----------------VAEILISY--RPGQRVVTRGRNIYRKKMADGREVSCVQRGVLVPRGELMEASFYGK----------IL
404487228	*Barnesiella intestinihominis* YIT 11860	--------LRAEEGPDFNKMS---------------------------------LERYIQ--------------SQPHF---SVAQV----------------------REA----------------VDRILVSF--RAGKRAVTPGKRYIRKNRKRISVQSVLIP-----RGALSEESVYGV----------IH
374384763	*Odoribacter laneus* YIT 12061	--------YNARRENKKRGLD---------------------------------STEHF---------------PSPWP---GFAQDV-----------------------RQS----------------VVPLLVSY--KQNPKTLCKISKTLYKDGKKIHSCGNAV------RGQLHKETVYGQ----------RT
384109266	*Treponema* sp. JC4	--------KNSHKGNRIEI--PEF---PILRSDL------------------------IEK-----------------VKNIVVSF--KPDHGA--------------------------EGKLSKETLLGK----------IK
402849997	*Rhodovulum* sp. PH10	--------DAARAEDQDLDRV-----------------------------------FRDV---------------PVPFE---DFRDHV-----------------------RER------------------VSTITVAV--KPEHGK--------------------------GGALHEDTSYGL----------VP
Jnet	331001027	---------------------------EEEEE---------------------------EEE--EEEE-----EEEE-------------------------------------EE-----
331001027	*Parasutterella excrementihominis* YIT 11859	-------------------TKRMGSQLAIYEPINFDN----------LFTGSCQVIQNTPRNFSDKTNIANS---PIFKETIY---AERFLDIIVSRG--EIFIGYP--SNMPFEEK-------------------PNRISIGGKD
34557932	*Wolinella succinogenes* DSM 1740	-------------------NADEVAKLAMLDSWNPASNEPLT---------KGLSTNQKIEKMIKSGDYGQKNMREVFGK---SIFGENAI---GERYKPIVVQEG--GYYIGYPATVKKGYELK----------------NCKVVTSKND
54296138	*Legionella pneumophila* str. Paris	-------------------KEFPQFSQELDNSWFINH--------------LMPDEVHLNPVRSKEKYNKRIGSST---PLFKDSLY---AERFIPVWVKGE--TFAIGFS--EKDLFEIK-------------------PSNKEK
319941583	*Sutterella wadsworthensis* 3 1 45B	-------------------AERDQNGFDYLDGKTVLG--------------LYPQSCEVIHLQAKPQEEKSHFDSV--AIFKEGIY---AEQFLPIFTLNE--KIWIGYE--TLNAKGER-------------------CGAIEVSGKQ
254447899	gamma proteobact. HTCC5015	-------------------QPHTASRLKTISSTSFEKTGWRS---------ALIPDLIKVDALDRRPKYRRYNIGST--SLFKDGIY---AENFLPILIDEN--GLMAGYD--IDNSLKAK-------------------GADVVFESLS
118497352	*Francisella novicida* U112	-------------------KNYSLYPLDKNTGEVFTKDIFSQ---------IKITDNEFSDKKLVRKKAIEGFNTHR---QMTRDGIY---AENYLPILIHKELNEVRKGYT--WKNSEEIKIFKGKKYDIQQLNNLVYCLKFVDKPIS
Motifs		______________]____H_________________________
informative positions		--

227494853	*Actinomyces coleocanis* DSM 15436#	-TKLQMHKVGDAWSLTEIDRAST-----PALWCALTR-QPDFTWKDGL--------------------------PANEDRTIIVNGTHYGPLDKVGIF--GKAAASLLVRGG-----------SVDIGSA
328956315	*Coriobacterium glomerans* PW2	-------PRAKKTAALPLKQGLN-----PSRYGSFSREQFAYFFIYKARNPRK---------------------EQTLFEFAQVPVRLSAQIRQDENA---LERYARELAKD-------------QGLEFIR
227824983	*Acidaminococcus* sp. D21	---------KASTGLVPRKAGLD-----VVKYGGYDKSTAAYYLLVRFTLED----------------------KKTQHKLMMI---PVEGLYKARID--HDKEFLTDYA-----------QTTISEI
303229466	*Veillonella atypica* ACS-134-V-Col7a	SVAAKAKDGAYIGMKTKYSVFAD-----VTKYGGMTKIKNAYSIIVQYTGKK----------------------GEEIKEIVPLPIYLINRNATDIEL---IDYVKSVIPKAKDISIKYR-----------KLCINQL
34762592	*Fusobacterium nucleatum* ATCC 49256	KGETSNEIISIKPKLYDGKDNKL-----NEKYGYYTSLKAAYFIYVEHEKKNK---------------------KVKTFERITRIDSTLIKNEKNLIK---YLVSQKKLLNPKIIKKI-----------YKEQTLI
374307738	*Filifactor alocis* ATCC 35896	-------QNKNGNSTVSLKKGLD-----VKKYGGYFSANTSYFSLIEF--------------------------EDKKGDRARH---IIGVPIYIANM---LEHSPSAFL-----------EYCEQKG
320528778	*Solobacterium moorei* F0204	--------VPKGKGQFPIKQGKD-----IEKYGGYNKLSSAFLFAVEYKGKKAR--------------------ERSLETVYIK---DVELYLQDPIK---YCESV-----------LGLKEPQ
291520705	*Coprococcus catus* GD-7	----MKKGKGQVPIKGNDERLAD-----IEKYGGYNKAAGTYFMLVKSLDKKGK--------------------EIRTIEFVPL---YLKNQIEINHE--SAIQYLAQERGLNS-----------PEILLSK
42525843	*Treponema denticola* ATCC 35405	-----MKKGLGQHPLKKEGPFSN-----ISKYGGYNKVSAAYYTLIEYEEK-----------------------GNKIRSLETIPLYLVKDIQKDQDV---LKSYLTDLLGKKE-----------FKILVPK
304438954	*Peptoniphilus duerdenii* ATCC BAA-1640	-----MRKGKGQIPQKENTNKSN-----IEKYGGYNKASSAYFALIESDGKAG---------------------RERTLETIPIMVYNQEKYGNTEAV--DKYLKDNLELQD-----------PKILKDK
224543312	*Catenibacterium mitsuokai* DSM 15897	SNDAHADKGVTKAVVPVNKNRSD-----VHKYGGFSGLQYTIVAIEGQKKKGK---------------------KTELVKKISGVPLHLKAASINEKI--NYIEEKEGLSDVRI-----------IKDNIPV
116628213	*Streptococcus thermophilus* LMD-9	------KPKPNSNENLVGAKEYLD----PKKYGGYAGISNSFTVLVKGTIEKG---------------------AKKKITNVLEFQGISILDRINYRK--DKLNFLLEKGYK-----------DI--ELI
24379809	*Streptococcus mutans* UA159	------LPKGNSDKLIPRKTKKFYWD--TKKYGGFDSPIVAYSILVIADIEKG---------------------KSKKLKTVKALVGVTIMEKMTFER--DPVAFLERKGYR-----------NVQEENI
13622193	*Streptococcus pyogenes* SF370	------LPKRNSDKLIARKKDWD-----PKKYGGFDSPTVAYSVLVVAKVEKG---------------------KSKKLKSVKELLGITIMERSSFEK--NPIDFLEAKGYK-----------EVKKDLI
310286728	*Bifidobacterium bifidum* S17	-----ATDPKGIKGIPFDGAKQD-----TSLYGGFSSAKPAYAVLIESKG------------------------KTRLVNVTMQ---EYSLLGDRPSD---DELRKVLAKKKSE-----------YAKANIL
366983953	*Oenococcus kitaharae* DSM 17330	---SVLHDAKSKASINFDKHKP------VDLYGGFSSAKPAYAALIKFKN------------------------KFRLVNVLRQ--WTYSDKNSEDYI---LEQIRGK-----------YPKAEMV
422884106	*Streptococcus sanguinis* SK49	---KFNKQLAANNRYPLKTKALCLD---TSIYGGYGPMNSALSIIIIAERFNE---------------------KKGKIETVKEFH-DIFIIDYEKFN---NNPFQFLNDTSENGFLKKNNIN---RVLGFY
339625081	*Fructobacillus fructosus* KCTC 3544	-----PKS--GKKLIPLNSKRSD-----TALYGGYSNVYSAYMTLVRANG------------------------KNLLIKIPISIA-NQIEVGNLKIN---D----YIVNNPAIKKFEK------ILISKL-
306821691	*Eubacterium yurii* ATCC 43715	-ADKTPKKPNLQAYRPIKTS-DERLCN-ILRYGGRTSISISGYCLVEYVK------------------------KRKTIRSLEAIP-VYLGRKDSLSE---EKLLNYFRYNLNDGGKDS------VSDIRLC
336394882	*Lactobacillus farciminis* KCTC 3681	-----PKNPKYKKLIAQKKD-MD-----PNIYGGFSGDNKSSITIVKIDN------------------------NKIK---PVAIP-IRLI-NDLKDK---KTLQNWLEENVKHKKSIQ------IIKNNV-
323463801	*Staphylococcus pseudintermedius* ED99	-----PKTAELKYESNKSN---------EVVYKGLTPYQTYVVAIKSVNK------------------------KGKEKMEYQMID-HYVFDFYKFQN---GNEKELALYLAQRENKDE------VLDAQI-
389815359	*Planococcus antarcticus* DSM 14505	-----PKVKQAKYVSPKTE---------KFVHDEVKNHSICLVEFTFMK-------------------------KEKEVQETKFID-LKVIEHHQFLK---EPESQLAKFLAEKETNSP------IIHARI-
258509199	*Lactobacillus rhamnosus* GG	--AKDSKERGGSKQLIPKKQGYP-----TQVYGGYTQESGSYNALVRVAEA-----------------------DTTAYQVIKISAQNASKIASANLK--SREKGKQLLNEIVVKQLAKR-----------RKNWKPS
169823755	*Finegoldia magna* ATCC 29328	--KKDYKKGKIYLPLKKDDRLQD-----VSKYGGYKAINGAFFFLVEHTKS-----------------------KKRIRSIELFPLHLLSKFYEDKNT---VLDYAINVLQ--------------LQDPKII
Jnet	227501312	------------------------------EEEE-----EEEEEEEE-EE----------------------------EEEEEEE----HHH---HHHHHHHHH----------------------
47458868	*Mycoplasma mobile* 163K	DKGKNTIKKVEKLNLLDNRTDKI-----KKIEEFFDEDKLKENELTKLHIFNH---------------------DKNLYETLKIIWNEVKIEIKNKNL--NEKNYFKYFVNKKLQEGKISFN------EWVPILDNDFKI
284931710	*Mycoplasma gallisepticum* str. F	ENKNSKVKRQFVYRKLVNVSLLN-----NDKLADLFAEKEDILMYRANPWVINLAEQIFNEYTE----------NKKIKSQNVFEKYMLDLTKEFPEK--FSEFLVKSMLRNKTAIIYDD-----------KKNIVHR
71894592	*Mycoplasma synoviae* 53	ENNFYKLEKVNLFSRKDLRKFIL-----EDLNEESEKNKKNENGSRKRILT-----------------------EKYIVDEILQILENEEFKDSKSDI---NALNKYMDSLPSKFS-----------EFFSQDF
363542550	*Mycoplasma ovipneumoniae* SC01	---YFKIIKKKLVTSK---------------NEELKKYFENPFGKKADGKSEYTVLMAQSHLSEFNKLKEIFE----KYNGFSNKTG--NAFVEYMNDLALKEP-----------T-LKAEI
384393286	*Mycoplasma canis* PG 14	ITNYYKKEKFSILDDKDIYLRLL-----RE-REKFVINQSNPE-------VIDQIIEIIESYGKENNIPSRDEAINIKYTKNKINYN--LYLKQYMRSLTKSLD-----------Q-FSEEF
238924075	*Eubacterium rectale* ATCC 33656	YDGKQYKINKLDIRTKEGIKVFA-----KLAFSKKDSDRERLLVYLN---------------------------DRRTFDDLCKIYEDYSDAANPFVQ---YEKETGDIIRKYS-----------KKHNGPR
315149830	*Enterococcus faecalis* TX0012	QKITTDEYTIGKIKDIYTLDGWE------AFKKKQDKLLMKDL-------------------------------DEKTYERLLSIAETTPDFQEVEEK--NGKVKRVKRSPFAVYCEENDIPAIQKYA-----------KKNNGPL
116627542	*Streptococcus thermophilus* LMD-9	KDKADETYVLGKIKDIYTQDGYD------AFMKIYKKDKSKFLMYRH---------------------------DPQTFEKVIEPILENYPNKQINEK---GKEVPCNPFLKYKEEHGYIRKYS-----------KKGNGPE
315659848	*Staphylococcus lugdunensis* M23590	STRKKDNSTYIVQTIKDIYAKDN-----TTLKKQFDKSPEKFLMYQH---------------------------DPRTFEKLEV---IMKQYANEKNP--LAKYHEETGEYLTKYS-----------KKNNGPI
160915782	*Eubacterium dolichum* DSM 3991	---YSTRVIDGKEKVVKKYKDIY-----DPKFTALAEDILNNAYQEKYLMALH---------------------DPQTFDQIVKVVNYYFEEMSKSEK--YFTKDKKGRIKISGMNPLSLYRDEHGMLKKYS-----------KKGDGPA
325677756	*Ruminococcus albus* 8	IRVKEIDGKLIKLKRKSISEITA-----ESINSIYTD-------------------------------------DKILIDSLKT---IFEQADYKDVG---DYLKKTNQHFF-----------TTSSGKR
225377804	*Roseburia inulinivorans* DSM 16841	----------RSARDYETRGVVI----TKVPLTDLKLNKDNEIEGYYDKDS-----------------------DRLLYQALVR---QLLLHGNDGKK---AFAEDFHKPKA-----------DGTEGPV
336393381	*Lactobacillus coryniformis* KCTC 3535	DEKGIVLSRVSITKLKINKKGQV-----ATGDGIYDPENSNNG-------------------------------DKVVYSAIRQALEAHNGSGELAFP---DGYLEYV-----------DHGTKKL
310780384	*Ilyobacter polytropus* DSM 2926	----------RSSKKISNQIAVS-----KKPLNSIKLKDLEKMQGRDT--------------------------DRKLYEALKN---RLEEYDDKPEK---AFAEPFYKPTN-----------SGKRGPL
301311869	*Bacteroides* sp. 20 3	VSFNEALKKPQAIVEMDLKKKIL-----AMLELGYDTKRIKNYFEENKDTWQDINPS-----------------KIKVYYFTKETKDRYFAVRKPIDTSFDKKKIKESIT--DTGIQQIMLRHLETKDNDPTLAFS-----------PDGIDEM
385811609	*Ignavibacterium album* JCM 16511	KNDP-VRKKKEKYIYDDYAQKVI---AKIVQELGLSSSMRKQDDEKLNKFINEAKVSAGVNKNLNTTNKTI---YNLEGRFYEKIKVAEYVLYKAKRMPLNKKEYIEKLSL-------QKMFNDLPNFILEKSILDNYPEILKELESDNKYIIEPHKKNNPVNRLLLEHILEYHNNPKEAFS-----------TEGLEKL
60683389	*Bacteroides fragilis* NCTC 9343	DMRKIGTVSKSAYRDALLKRLYENDNDPKKAFAGKNSLDKQPIWLDKEQMRKV---------------------PEKVKIVTLEAIYTIRKEISPDLK---VDKVIDVGVRKILIDRLNEYGNDAKKAFSNLDKNPIWL--NKEKGIS
319957206	*Nitratifractor salsuginis* DSM 16511	LRKNILEAFDSLNVIKDRHKLTT-----EEFMKRYDKEIRQKLWLHRIGNT-----------------------NDESYRAVEERATQIAQILTRYQL--MDAQNDKEIDEKFQQALKELITSP-----------IEVTGKL
187250660	*Elusimicrobium minutum* Pei191	SERGYTLTTYKKLSALKLTDPQK-----KKTPKDFLETALLKFKGRESEMKNEKIKSAIEN-------------NKRLFDVIQDNLEKAKKLLEEENE---KSKAEGKKEKNINDASIYQ-----------KAISLSG
325972003	*Sphaerochaeta globus* str. Buddy	SILGADTQGEDLVFVVKKKIKDI-----GVKIGDYEEVASAIRGRIT---------------------------DKQPKWYPMEMKDKIEQLQSKNEA--ALQKYKESLVQAAAVLEESNRKLIESGKKPIQ
296446027	*Methylosinus trichosporium* OB3b	----RHIAVREGEQRVYERRKVA-----ELKLADLDRVKDAER-------------------------------NARLIEKLRNWIEAGSPKDDPPLS---PKGDP
347536497	*Flavobacterium branchiophilum* FL-15	LDGEGKKLPRLQQGDTIRGSLHQ-----DSIYGAI-KNPLNTDE----------------IKYVIRKDL--------ESI-KGSDVES-IVDEVVKEKIKEAIANKVLL---LSSNAQQKNKLVGTVWM--NEE------------KRIA
345885718	*Prevotella* sp. C561	--------PRWQTGDALRGEIHK-----ASYYGAI--TQFAKDDEGKVLMKEGRPQVNPTIKFVIRRELKYKKSAADSGFASWDDLGKAIVDKELFALMKGQFPAETSF--KDACEQGIYMIKKGKNGMPD------IKLHH
282880052	*Prevotella timonensis* CRIS 5C-B1	---------VLAQGDTARGSLHL-----DTYYGAI-------ERDGE-------------IRYVVRRPL--------SSFTKPEELEN-IVDETVKRTIKEAIADKNF---KQAIAEPIYM--NEE------------KGIL
312879015	*Aminomonas paucivorans* DSM 12260	-------RSPKWKDHPEGPRTAS-----RIPLKDLNLEKLERMVGKDR--------------------------DRKLYEALRE---RLAAFGGNGKK---AFVAPFRKPC------------RSGEGPL
294086111	*Candidatus Puniceispirillum marinum* IMCC1322	----YGIVEHAENGASTVVHRVP-----ITSLGKQSDIEKVR--------------------------------DPLIKSALLNETAGLSGKSFENAV---Q-----------KWCADNS
330822845	*Alicycliphilus denitrificans* K601	----------------YGIRKDG-----SIKQRRKADGSAGR---------------------------------EISNLIRIH---EATQPLRHGVS---ADGQPLA
344171927	*Ralstonia syzygii* R24	EDGRYRVRHRVSLFDLKPGDLSN-----VRCDAPLQAELEPIFEQD----------------------------DARAREVALTALAERYRQRKVWLE--ELMSVLPIRP-----------RGEDGKT
159042956	*Dinoroseobacter shibae* DFL 12	------YSIVDDIHVASRTDLLS-----LKPAQLLDEPGRSGQVR-----------------------------DPQLRKALRV---ATGGKTGKDFE--NALRYFASKPGPYQ-----------------A
83591793	*Rhodospirillum rubrum* ATCC 11170	FGVRNRPDARVLVQRKPVEKLFL-----DANAKPLPADKIAEIIDGFA--------------------------SPRMAKRFKALLARYQAAHPEVPP--ALAALAVARDPA-----------FGPRGMT
288957741	*Azospirillum* sp. B510	--------YGPVDPPEEGFNLVV-----RKPIDGLSKDEINSVR------------------------------DPRLRRALIDRLAIRRRDANDPAT--ALAKAAEDLA-----------AQPASRG
427429481	*Caenispirillum salinarum* AK4	VVTQRMNARDLVALLDNAKIVPAARLDAAAPGDTILKEICTEIADRHDRVVDPEGTHARRWISARLAALVPAHAEAVARDIAELADLDALADADRTPEQEAR--RSALRQSPYLGRAISAKKADGRARAREQEILTRALLDPHWGPRGLRHL-I
92109262	*Nitrobacter hamburgensis* X14	-YGFVKPLDATGLKEEEAGNLVY-----RKAIESLNENEVDRIR------------------------------DIQLRTIVRD---HVNVEKTKGVA--LADALRQLQAPSDDYP-------------QFKHG
148255343	*Bradyrhizobium* sp. BTAi1	------YGTVEHPETEDGANLVY-----RKTFVDISEKEIDRIR------------------------------DRRLRDLVRA---HVAGERQQGKT---LKAAVLSFAQRRD-----------IAGHPNG
34557790	*Wolinella succinogenes* DSM 1740	-----------------------------KPYPRIKQVKNKKKWRLAPI-------------------------DEEKFESFKA---DRVASAN
218563121	*Campylobacter jejuni* NCTC 11168	-----------------RKEEEF-----YQSYGG--KEGVLKAL-----ELGKIRKVNGK--
Jnet	218563121	--EEEE--
291276265	*Helicobacter mustelae* 12198	-----------------RTKEFY-----YQAFGG--EEGVKKAL-----KFGKIREINQG--
222109285	*Acidovorax ebreus* TPSY	AQPESLKQQGGVIEKILLTSLKL-----QDFDKLLNPESNDHFVEPHR--------------------------NERLYAAIRQ---RLEQFGGRADK--AFGPDNLFHKPDKN-----------NQPTGPV
365156657	*Bacillus smithii* 7 3 47FAA	VGIDEQSGKIQTAVKTKLSDIK------LDKDGHFPMYQKES--------------------------------DPRTYEAIRQ---RLLEHNNDPKK---AFQEPLYKPKK-----------NGEPGPV
220930482	*Clostridium cellulolyticum* H10	-----RSPKHFNKGLTSVKIPLT-----TVTLEKLETMVKNTKGGIS---------------------------DKAVYNVLKN---RLIEHNNKPLK---AFAEKIYKPLK-----------NGTNGAI
297182908	uncultured delta proteobact. HF0070 07E19	-------RKHRSKP-DRQRVALT-----KVKLADLERLVEKDA---S---------------------------NRNLYEHLKQ---CLEESGDQPTK---AFKAPFYMP----------------SGPE
154250555	*Parvibaculum lavamentivorans* DS-1	---YGDTGTDIKTKSGTYRQFVT-----RKKIESLSKGELDEIR------------------------------DPRIKEIVAA---HVAGRGGDPKK--AFPPYPC----------VSPGGPE
218767588	*Neisseria meningitidis* Z2491	----------KSAKRLDEGVSVL-----RVPLTQLKLKDLEKMVN-RER-------------------------EPKLYEALKA---RLEAHKDDPAK---AFAEPFYKYDK-----------AGNRTQQ
15602992	*Pasteurella multocida* str. Pm70	----------KSAKRLAEGISVL-----RIPLTQLKPNLLENMVN-KER-------------------------EPALYAGLKA---RLAEFNQDPAK---AFATPFYK--Q-----------GG---QQ
187736489	*Akkermansia muciniphila* ATCC BAA-835	-SASLKENIREQLMEQRVIQHVP-----ADMGGALLKETMQRVLSV----------------------------DGSGEDAMVSLSKKKDGKKEKNQV--KASKLVGVFP-----------EGPSK--
315605738	*Actinomyces* sp. oral taxon 180 str. F0310	RPRPQRYVLGDALPADVINRVTD-----PGLWTALVR-APGFDSQLGL--------------------------PADLNRGLKLRGKRISADFPIDYF--PTDSPALAVQGGYV-------------GLE
117929158	*Acidothermus cellulolyticus* 11B	-RAFSEHTVGAAWKGAELRRIVE-----PEVYAAFLALTDPGGRFLKVSPS-----------------------EDVLPADENRHIVLSDRVLGPRDR---VKLFPDDRGSIRVRG-----------GAAYIAS
189440764	*Bifidobacterium longum* DJO10A	KVPLGSAMSADLIRRASTPALWCA----LTRLPDYDEKEGL---------------------------------PEDSHREIRVHDTRYSADDEMGFF--ASQAAQIAVQEG-----------SADIGSA
403744858	*Alicyclobacillus hesperidum* URH17-3-68	VKVDDNKGTEIQ--VVVSKVALTE----LKLTKDGEI----------------KDYFRP------------EDDPRLYNTLRERL-VQFGGDAKAAF--KEPVYKISKD-------------GSVRTP
407803669	*Alcanivorax* sp. W11-5	RLDERSGEKVD---WLVSRKSLTD----LAPEKKSID----------------VDKVRA------------NISRIVGEAIRLHISNIFEKRVSKGMT--PQQALREPIEF---------------QGNI
423317190	*Bergeyella zoohelcum* ATCC 43767	GKEAY-------------RIPLTK----FGGSKFAT-----------------EKNIQK------------IVSPFLSGFIANHL-KEYNNKKEEAFS--AEGIMDLNNKLAQYRNEK----GELKPHTP
402847315	*Porphyromonas* sp. oral taxon 279 str. F0450	SQGRV---------RIVKRYPLHD----LK---------------------------GE------------VVDPHLRELITTYN-QELKSREKGAP--IPPLCLDKDK----------------KQE
404487228	*Barnesiella intestinihominis* YIT 11860	VWEKDEQGHVIQKQRAVMKYPITS----INRE---------------------MLDKEK------------VVDKRIHRILSGRL-AQYNDNPKEAF--AKPVYIDKEC----------------RIP
374384763	*Odoribacter laneus* YIT 12061	APGATEK-------SYHIRKDIRE----LKT----------------------SKHIGK------------VVDITIRQMLLKHLQENYHIDITQEFN--IPSNAFFKEGV-YRIFLP----NKHGEPVP
384109266	*Treponema* sp. JC4	LHGKE---------TFVCRENIVS----LS-----------------------EKNLDD------------IVDEKIKSKVKDYV-AKHKGQKIEAV--LSDF--------------SKENG
402849997	*Rhodovulum* sp. PH10	DTDPNAALG-----NLVVRKPIRS----LT-----------------------AGEVDR------------VRDRALRARLGALA-APFRDESGRVRD--AKGLAQALEAF--------------GAENG
Jnet	331001027	HHHHHHHHHHHHH--------EEEEEE---------HHHHHHHHHHH------------HHHHHH----HHHHH-EEEEEEEEEEEEHHH-------HHHHH--HHH-HHHHHHHHHH---EEE---EEEEEE---HHHHHHHHHHH-------
331001027	*Parasutterella excrementihominis* YIT 11859	PFSILSVLGAYLDKAPSSEKEKLTIYRVV------KNKAFELFSKVAG----SKFTAEEDKAAKI---LEALHFVTVKQDVAATVSDLIKSKKELSKDSIEN--LAKQKGCLKKVEYSSKEFKFKGSLIIPAAVEWGKVLWNVFKENTAEELKD
34557932	*Wolinella succinogenes* DSM 1740	IAKLEKII-------KNQDLISLKENQYIKIFSINKQTISELSNRYFNMNYKNLVERDKEIVGLLEFIVENCRYYTKKVDVKFA----PKYIHETKYPFYDD--WRRFDEAWRYLQENQNKTSSKDRFVIDKSS--LNEYYQPDKNEYKLD-VD
54296138	*Legionella pneumophila* str. Paris	LFTLLKTYSTKNPGESLQELQAKSKAKWL-YFPINKTLALEFLHHYFH---KEIVTPDDTTVCHF---INSLRYYTKKESITVKI-------------LKE---PMPVLSVKFESSKKNVLGSFKHTIALPATKDWERLFNHPNFLALKANPAP
319941583	*Sutterella wadsworthensis* 3 1 45B	PKELLEMLAPFFNK-PVGDLSAHATYRIL------KKPAYEFLAKAAL----QPLSAEEKRLAAL---LDALRYCTSRKSLMSLFMA-ANGKSLKKREDVLK--PKLFQLKVELKGEKS--FKLNGSLTLPVKQDWLRICDSPELADAFGKPCS
254447899	gamma proteobact. HTCC5015	PFLLFK--GEEVGAQSLSDWQERIDGRYL-YMSIDKVKAFDYLQEKVG---------EKDIAAEL---LNSIHFTQRKTELRAKFSD-DSGKKMKTLDAIRK--SLKLTVTVNEIGKRKEKCGFSGTIGIPAKSAWENLLDEPLLETYWGTKMP
118497352	*Francisella novicida* U112	IDIQISTLEELRNILTTNNIAATAEYYYI---NLKTQKLHEYYIENYN---TALGYKKYSKEMEF---LRSLAYRSERVKIKS--IDDVKQVLDKDSNF--IIGKITLPFKKEWQRLYREWQNTTIKDD---
Motifs		____
informative positions		--

227494853	*Actinomyces coleocanis* DSM 15436#	IHH------ARIY------------------RIAGKKP------------TYGMVRVFAPDLLR----------------YRNEDLFNVELPP-------------------------QSVSMRYAEPKVREAIR----------------------EGKAEYLGWLVVGDELLLDLSSETSGQIA----ELQQDFPGTT
328956315	*Coriobacterium glomerans* PW2	IER------SKILKNQLIEIDGDRLC------ITGKEEVRNACEL------AFAQDEMRVIRMLVSEK-----------------PVSRECVIS--------------------------LFNRILLHGDQASRRL-------------------------SKQLKLALLSEAFSEASDNVQRN----------VVLGLIAIFN
227824983	*Acidaminococcus* sp. D21	LQK----DKQKVINI-----------------MFPMGTR------------HIKLNSMISIDGFYLSI---GGKSSKGKSVLCHAMVPLIVPHK----------------IECYIKAMESFARKFKENNKLRIVEKFDKITV-------------EDNLNL---YELFLQKLQHNPYNKFFS-------TQFDVLTNGR
303229466	*Veillonella atypica* ACS-134-V-Col7a	VKV---NGFYYYLGG-----------------KTNDKIYIDN---------AIELVVPHDIATYIKLL---DK---------YDLLRKENKTLKA----------------SSITTSIYNINTSTVVSLSNKVGIDVFDYF------------------MSKLRTPLYMKMKGNKVDELSST----------GRSKFIKMTL
34762592	*Fusobacterium nucleatum* ATCC 49256	IDS------YPYTFTGVDSNKKVELKNKKQ--LYLEKKYEQ----------ILKNALKFVEDNQGETE-------------ENYKFIYLKRNN----------------NEKNETIDAVKERYNIEFN---------------------EMYDKFL---EKLSSKDYKNYINN-----------KLYTNFLNSK
374307738	*Filifactor alocis* ATCC 35896	YQN------VRILVE-----------------KIKKNSL------------LIINGYPLRIRGENEVD---TS------FKRAIQLKLDQKN-----------------YELVRNIEKFLEKYVEKKGNYPIDENRDHITH--------------EKMNQL---YEVLLSKMKKFNKKGMADPS-----DRIEKSKPKF
320528778	*Solobacterium moorei* F0204	IIK------PKILMGSLFSINNKKLVVT----------------------GRSGKQYVCHHIYQLSI--------------NDEDSQYLKNIAK----------------YLQEEPDGNIERQNILNITSV--------------------NNIKLFDVLCTKFNSNTYEIILN----------SLKNDVNEGR
291520705	*Coprococcus catus* GD-7	IKI------DTLFKV----DGFKM--------WLSGRTGNQL---------IFKGANQLILSHQEAAI-------------LKGVVKYVNRKNE----------------------NKDAKLSERDGMTEEKL------------------LQLYDTF--LDKLSNTVYSIRLS----------AQIKTLTEKR
42525843	*Treponema denticola* ATCC 35405	IKI------NSLLKI----NGFPC--------HITGKTND-----------SFLLRPAVQFCCSNNEV-------------LYFKKIIRFSEIR----------------SQREKIGKTISPYEDLSFRSYIKENLWKKTKNDEIGE---------KEFYDLLQKKNLEIYDMLLTKHKDTIYKKRPNSATIDILVKGK
304438954	*Peptoniphilus duerdenii* ATCC BAA-1640	IKI------NSLIKL----DGFLY--------NIKGKTGD-----------SLSIAGSVQLIVNKEEQ-------------KLIKKMDKFLVKK----------------KDNKDIKVTSFDN----------------------------IKEEELIKLYKTLSDKLNNGIYSNKRN------NQAKNISEAL
224543312	*Catenibacterium mitsuokai* DSM 15897	NQM------IEMDGGEYLLTSPTE--------YVNARQLV-----------LNEKQCALIADIYN------------------------AIYKQDYDN----------------------------LDDILMIQLYI----------------------ELTNKMKVLYPAYRGIAEKFE--------------SMNENYVVIS
116628213	*Streptococcus thermophilus* LMD-9	IEL------PKYSLF-----------------ELSDGSRRMLASIL---STNNKRGEIHKGNQIFLSQ-------------KFVKLLYHAKRIS----------------NT-----INENHRKYVENHK------------------------KEFEELFYYILEFNENYVGAKKNG------------KLLNSAFQSW
24379809	*Streptococcus mutans* UA159	IKL------PKYSLF-----------------KLENGRKRLLASA----------RELQKGNEIVLPN-------------HLGTLLYHAKNIH----------------KV-----DEPKHLDYVDKHK------------------------DEFKELLDVVSNFSKKYTLAEGNL----------EKIKELYAQN
13622193	*Streptococcus pyogenes* SF370	IKL------PKYSLF-----------------ELENGRKRMLASA----------GELQKGNELALPS-------------KYVNFLYLASHYE----------------KLKGSPEDNEQKQLFVEQHK---------------------HYLDEIIEQISEFSKRVILADANL----------DKVLSAYNKH
310286728	*Bifidobacterium bifidum* S17	LRHVPKMQLIRYGGGLMVIKSAGELNN-----------------------AQQLWLPYEEYCYFDDL---S----------QGKGSLEKDDLKK------------------------------LLDSILGSVQC---LYPWHRFTE---------------EELADLHVAFDKLPEDEKKNVIT----------GIVSALHADA
366983953	*Oenococcus kitaharae* DSM 17330	LSHIPYGQLVKKDGALVTISSATELHN-----------------------FEQLWLPLADYKLINTL---L----------KTKEDNLVDILHNRLDLPEMTIESAFY-------------KAFDSILSFAFN---RYALHQNAL------------VKLQAHRDDFNALNYEDKQQTLE----------ILLDALHASP
422884106	*Streptococcus sanguinis* SK49	RIP-KYSLMQKIDGTRMLFESKSN------LHK-----------------ATQFKLTKTQNELFFHM---K----------RLLTKSNLMDL-KSKSAIKESQNFIL-------------KHKEEFDNISNQLSA--------FSQKMLGN------TTSLKNLIKGYNERKIKEIDIRDETIKYFY----DNFIKMFSFV
339625081	*Fructobacillus fructosus* KCTC 3544	----PLGQLVNEDGNLIYLASNEY------RHN-----------------AKQLWLSTTDADKIASI---S----------ENSSDEELLEA----------------------------YDILTSENVKNRFP-------FFKKD----------IDKLSQVRDEFLDS---------DKRIAVIQ----TILRGLQIDA
306821691	*Eubacterium yurii* ATCC 43715	LPFISTNSLVKIDGYLYYLGGKNDD--RIQLYN-----------------AYQLKMKKEEVEYIRKI---E----------KAVSMSKFDEIDREKNPVLTEEKNIE-------------LYNKIQDKFENTVFSKRMSLVKYNKKDLSF------GDFLKNKKSKFEEIDL------EKQCKVLY----NIIFNLSNLK
336394882	*Lactobacillus farciminis* KCTC 3681	---PIGQIIYSKKVGLLSLNSDRE------VAN-----------------RQQLILPPEHSALLRLL---Q------------IPDEDLDQI----LAFYDKNILVE-------------ILQELITK-MKKFYP-------FYKGE---------REFLIANIENFNQATT-------SEKVNSLE----ELITLLHANS
323463801	*Staphylococcus pseudintermedius* ED99	VYSLNKGDLLYINNHPCYFVSRKE------VIN-----------------AKQFELTVEQQLSLYNV---M----------NNK-ETNVEKL-------------LI-------------EYDFIAEK-VINEYH-------HYLNSKLK------EKRVRTFFSESNQTH--------EDFIKALD----ELFKVVTASA
389815359	*Planococcus antarcticus* DSM 14505	IRTIPKYQKIWIEHFPYYFISTRE------LHN-----------------ARQFEISYELMEKVKQL---S----------ERSSVEELKIV-----------------------------FGLLIDQ-MNDNYP--------IYTKS----------SIQDRVQKFVDTQLYDF---KSFEIGFE----ELKKAVAANA
258509199	*Lactobacillus rhamnosus* GG	ANS------FKIVIP-----------------------RFGMGTLFQN---AKYGLFMVNSDTYYRN--------------YQELWLSRENQKL-------------------------LKKLFSIKYEKTQMNHDALQVYK---------------AIIDQVEKFFKLYDINQFRAKLS----------DAIERFEKLP
169823755	*Finegoldia magna* ATCC 29328	IDK------INYRTEIIIDNFSYLIS------------------------TKSNDGSITVKPNEQMY--------------WRVDEISNLKKIE------------------------NKYKKDAILTEEDRKIME------------------SYIDKIYQQFKAGKYKNRRTTDT----------IIEKYEIIDL
Jnet	227501312	--------------EEEE---EEEEE-------------------------E------EEEE---EEE------------E--HHHHHHHHHH------------------------HHHHH-----HHHHHH------------------------H-HHHHHHHH---------HHHH-----------HHHHHHHHHH
47458868	*Mycoplasma mobile* 163K	IRK------IRYIKFSSEEKETDEIIFSQSNFL-----KIDQRQNFS----FHNTLYWVQIWVYKNQK---DQ--------YCFISIDARNSKF--------------------------EKDEIKINYEKLKTQKEKLQI----------------INEEPI---LKINKGDLFENE------------EKELFYIVGR
284931710	*Mycoplasma gallisepticum* str. F	IKR------LKMLSSELKENKLSNVII-----------RSKNQSGTKLS--YQDTINSLALMIMRSID---PTAKKQ------YIRVPLNTLNLHL----------------------GDHDFDLHNMDAYLKKPKFVKYLKAN------------EIGDEYKPWRVLTSGTLLIHKKD----------KKLMYISSFQ
71894592	*Mycoplasma synoviae* 53	INKCKKENSLILTFDAIKHNDPKKVIKIKNLKFFREDATLKNKQ-------AVHKDSKNQIKSFYESY-------------KCVGFIWLKNKND-------------LEESIFVPINSRVIHFGDKDKDIDFDS---------------------YNKEKLLNEINLKRPENKKFNSI----------NEIEFVKFVK
363542550	*Mycoplasma ovipneumoniae* SC01	ESAKSVEKLLYYNFKPSDQFTYHDNINNKSFKRFYKNIRIIEYKSIPIK--FKILSKHDGGKSFKDTL-------------FSLYSLVYK-VYENG----------------KESYKSIPVTSQMRNFGIDEFDFLDEN-----------------LYNKEKL----------DIYKSDFAKPIP-------VNCKPVFVLK
384393286	*Mycoplasma canis* PG 14	INQMIANKTFVL-YNPTKNTTR----KIK-FLRLVNDVKINDIRKNQVI--NKFNGKNNEPKAFYENI-------------NSLGAIVFK----NS---------------ANNFKTLSINTQIAIFGDKNWDIEDFK----------------TYNMEKI----------EKYKEIYGID------KTYNFHSFIF
238924075	*Eubacterium rectale* ATCC 33656	IDK------LKYKDG----EVGACIDISHKY-------GFEKGSKKV----ILESLVPYRMDVYYKEE---NHS-------YYLVGVKQSDIKF------------------EKGRNVIDEEAYARILVNEKMIQPGQSRAD---------------LENLGFKFKLSFYKNDIIEY------------EKDGKIYTER
315149830	*Enterococcus faecalis* TX0012	IRS------LKYYDG----KLNKHINITKDSQGRPVEKTKNGRKV------TLQSLKPYRYDIGYDLE---TKA-------YYTVQLYYSDLKF----------------------------VEGKYGITKEYMKVAEQTK---------GQVVRFC--FSLQKNDGLEIEWK----------DSQRYDVRFY
116627542	*Streptococcus thermophilus* LMD-9	IKS------LKYYDS---K-LGNHIDI----------TPKDSNNKV-----VLQSVSPWRADVYFNKT---TGK-------YEILGLKYADLQF------------------EKGTGTYKISQEKYNDIKKKEGV----------------DSDSEFK--FTLYKNDLLLVKDTET----------KEQQLFRFLS
315659848	*Staphylococcus lugdunensis* M23590	VKS------LKYIGN----KLGSHLDVTHQF-------KSSTKKL------VKLSIKPYRFDVYLTDK---G---------YKFITISYLDVKK----------------------------KDNYYYIPEQKYDKLKLGKAI--------------DKNAKFI--ASFYKNDLIKLD------------GEIYKIIGVN
160915782	*Eubacterium dolichum* DSM 3991	ITQ------MKYFDG----VLGNHIDISAHY-------QVRDKKV------VLQQISPYRTDFYYSKE---NG--------YKFVTIRYKDVRW------------------SEKKKKYVIDQQDYAMKKAEKKI---------------DDTYEFQ--FSMHRDELIGITKAEG----------EALIYPDETW
325677756	*Ruminococcus albus* 8	VNK------VTVIEK----VPSRWLRK----------EIDDNNFS------LLNDSSYYCIELYKDSK---GD--------NNLQGIAMSDVH-------------------------DRKTKKLYLKPDFNYP-----------------DDYYTHV--MYIFPGDYLRIKSTSKSEGQL--KFEGYFISVK
225377804	*Roseburia inulinivorans* DSM 16841	VRK------VKIEKK----QTSGV-------------MVRGG------TGIAANGEMVRIDVFRENG---K----------YYFVPVYTADVVR--------------------------KVLPNRAATHTKP----YSEWRVM------------------DDANFV--FSLYSRDLIHV-------------KSKKDIKTN
336393381	*Lactobacillus coryniformis* KCTC 3535	VRK------VRVAKKVSLPVRLKNKA-------------------------AADNGSMVRIDVFNTGK--K----------FVFVPIYIKDTVE--------------------------QVLPNKAIARGKS------LWYQI------------------TESDQFC--FSLYPGDMVHIESKT----------GIKPKYSNKE
310780384	*Ilyobacter polytropus* DSM 2926	VRG------IKVEEK----QNVGV-------------YVNGG---------QASNGSMVRIDVFRKNG---K----------FYTVPIYVHQTLL--------------------------KELPNRAINGKPY----KDWDLI------------------DGSFEFL--YSFYPNDLIEIEF-----------GKSKSIKNDN
301311869	*Bacteroides* sp. 20 3	NRN------ILILNKGKKHQPIYKVRVYEKAFEEVTVGQKGNKRTKFV---EAAKGTNLFFAIYETEEIDKDTKKVIRKRSYSTIPLNVVIERQ------------------KQGLSSAPEDENGNLPKYILSPNDLVYVPTQ-----------------EEINKGEVVMPIDRDRIYKMVDSS----------GITANFIPAS
385811609	*Ignavibacterium album* JCM 16511	NKK------AI----NKIGKPIKYITRLDG----DINEEEIFRGAVF----ETDKGSNVYFVMYE----NNQTKDREFLKPNPSISVLKAIEHK------------------NKIDFFAP--NRLGFSRIILSPGDLVYVPTNDQYVLIKDNSSNETIINWDDNEFI----SNRIYQVKKFT---------GNSCYFLKND
60683389	*Bacteroides fragilis* NCTC 9343	IKR------VTISGINSNAQSLHVKKDKDGK--------PILDENGRNIPVDFVNTGNNHHVAVYYRPV---IDKRGQLVVDEAGNPKYELEEVV-----------------VSFFEAVTRANLGLPIIDKDYKTTEGWQFL-------------FSMKQNE--YFVFPNEKTGFPNKEIDLL-------DVENYGLISP
319957206	*Nitratifractor salsuginis* DSM 16511	LRK------MRFVYD----KLNAM-------------QIDRGL------VETDKNMLGIHISKGPN---EK----------LIFRRMDVNNAHE---------------------------LQKERSGILCYLN-------------------------EML--FIFNKKGLIHY-------------GCLRSYLEKG
187250660	*Elusimicrobium minutum* Pei191	DKY------VQLSKK----EPGKFFAISK----------PTPTTTGY----GYDTGDSLCVDLYYDNK---GK--------LCGEIIRKIDAQQ-----------------------------------KNPLKYK------------------EQGFTLF--ERIYGGDILEVDF------------DIHSDKNSFR
325972003	*Sphaerochaeta globus* str. Buddy	LSE------KTISKKALELVGGYYYLISNNKRTKTFVVKEPSNEVKGF---AFDTGSNLCLDFYHDAQ---GK--------LCGEIIRKIQAMN------------------PSYKPAYM---------------------------------KQGYSLY--VRLYQGDVCELRASDLTE---------AESNLAKTTH
296446027	*Methylosinus trichosporium* OB3b	IFK------VRLVTKSKVNIALDT-------------GNPKRPG------TVDRGEMARVDVFRKAS---KKGKYE-----YYLVPIYPHDIAT-------------------------------MKTPPIRAVQAYKPEDEWPEM------------------DSSYEFC--WSLVPMTYLQVISSKGE--------IFEGYYRGMN
347536497	*Flavobacterium branchiophilum* FL-15	INK------VRIYANSVKNPLHIKEHSLL---------SKSKHVHKQKV--YGQNDENYAMAIYE---LDGK------------RDFELIN-------------------------IFNLAKLIKQGQGFYPLHKKKEIK-------GKIVFVPIEKRNKRDVV--LKRGQQVVFYDKEVENPKDI--SEI--VDFKG
345885718	*Prevotella* sp. C561	IRH------VRC--EAPQSGLKIKEQT----------YKSEKEYKRYF--YAAVGDLYAMCCYT----NGK------------IREFRIYS---------------------------LYDVSCHRKSDIEDIP-EFITDKK---------------GNRLMLDYK--LRTGDMILLYK---DNPAEL--YDLDNVLNS
282880052	*Prevotella timonensis* CRIS 5C-B1	IKK------VRCFAKSVKQPINIRQHR-----------DLSKKEYKQQY--HVMNENNYLLAIYEGL-VKNKV-----------VREFEIVS--------------------------YIEAAKYYKRSQDRNIFSSIVPTH------------------STKYGLPLKTKLLMGQLVLMFE---ENPDEI--QVDNTKDLVK
312879015	*Aminomonas paucivorans* DSM 12260	VRS------LRIFDSGYSGVE---------------LRDGGEVYA------VKLDHESMVRVDVYAKKN---K----------FYLVPVYVADVAR--------------------------GIVKNRAIVAHKS----EEEWDLV------------------DGSFDFR--FSLFPGDLVEIEKKDG---------AYLGYYKSCH
294086111	*Candidatus Puniceispirillum marinum* IMCC1322	IKS------LRIVETVSII-----------------PITDKEGVAYK--GYKGDGNAYMDIYQDPT---SSK---------WKGEIVSRFDANQ-----------------------------KGFIPSWQSQ-----------------------FPTARLI--MRLRINDLLKLQDG---------EIEEIYRVQR
330822845	*Alicycliphilus denitrificans* K601	YK---GYVGGSNYCIEITVNDK---GK---------WEGEVISTFRAYG------------------------VRAGGMGRLRNPHEG------------------QNGRKLI--MRLVIGDSVRLEVD------------GAERTMRIVK
344171927	*Ralstonia syzygii* R24	LPD----------------SAPYK----------------------------AYKGDSNYCYELFINER---GR--------WDGELISTFRANQ----------------------AAYRRFRNDPARFRRYT---------------------AGGRPLL--MRLCINDYIAVGTA----------AERTIFRVVK
159042956	*Dinoroseobacter shibae* DFL 12	IRR------VRIIKPLQAQARVPV------------PAQDPIK-------AYQGGSNHLFEIWRLPD---GE---------IEAQVITSFEAKR-----------------------------LEGEKRPHPAAKRL-------------------------LRVHKGDMVALERDGR----------RVVGHVQKMD
83591793	*Rhodospirillum rubrum* ATCC 11170	ANT------VIAGRSDGDGEDAGLIT------------PFRANPKAAVRTMGNAVYEVWEIQVKGRPR-------------WTHRVLTRFDRTQ--------------------------PAPPPP---------------------------PENARLV--MRLRRGDLVYWPLESG----------DRLFLVKKMA
288957741	*Azospirillum* sp. B510	IRR------VRVLKKESNPIRVEHG------------NPSGPRSGGPFHKLLLAGEVHVDVFAL---GRR------------WVGHWVTLFEAHG-------------------------GRGADGAAAPPRL-----------------------GDGERFL--MRLHKGDCLKLEHK-----------GRVRMVQVVK
427429481	*Caenispirillum salinarum* AK4	MREARAPSLVRIRANKTDA-FGRPVPDAAVWVKTDGNAVSQLWRLTSVVTDDGRRIPLPKPIEKRIEISNLEYARLNGLDEGAGVTGNNAPPRPLRQDIDRLTPLWRDHGTAPGGYLGTAVGELEDKARSALRGKAMRQTLTDAG ITAEAGWRLDSEGA---
92109262	*Nitrobacter hamburgensis* X14	LRH------VRILKK----EKGDYLV-----------PIANRASGVAYK--AYSAGENFCVEVFETAG---GK--------WDGEAVRRFDANK------------------------KNAGPKIAHAPQWRDA------------------NEGAKLV--MRIHKGDLIRLDHE----------GRARIMVVHR
148255343	*Bradyrhizobium* sp. BTAi1	IRH------VRLTKS----IKPDYLV-----------PIRDKAGRIYK--SYNAGENAFVDILQAES---GR--------WIARATTVFQANQ--------------------------ANESHDAPAAQPI------------------------------MRVFKGDMLRIDHA----------GAEKFVKIVR
34557790	*Wolinella succinogenes* DSM 1740	QKN---FYETSTIPRVDVFHKKG---K----------FHLVPIYTHDIAY-------------------------NELPNLSLTNPE----------------------AMDENFFK-FSIFKDDLISIQTQGTPKKPAK--IIMGYFKNMH
218563121	*Campylobacter jejuni* NCTC 11168	---IVKNGDMFRVDIFKHKK---TNK-------FYAVPIYTMDFAL--------------------------KVLPNKAVARSKK-GEIKDWILM----------------DENYEFC--FSLYKDSLILIQTKDMQE------PEFVYYNAFT
Jnet	218563121	---EEEEEEEE--------E-------EEEEE----HHH---------------------------EEE--EE-EEE-----EEEEEE--------------EEEEEEE---
291276265	*Helicobacter mustelae* 12198	---IVDNGAMVRVDIFKSKD---KGK-------FYAVPIYTYDFAI--------------------------GKLPNKAIVQGKKNGIIKDWLEM----------------DENYEFC--FSLFKNDCIKIQTK----------EMQEAVLAIY
222109285	*Acidovorax ebreus* TPSY	VRS------IKLVRG----KQTGI-------------PIRGG---------LAKNDSMLRVDIFTHAG---K----------FHLVPVYVHRVT--------------------------GLPNRAIVAFKD---EDEWTLI------------------DESFAFL--FSVYPNDYVKVTLKKE---------QQSGYYSGAD
365156657	*Bacillus smithii* 7 3 47FAA	IRT------VKIIDT----KNKVV-------------HLDGSKT-------VAYNSNIVRTDVFEKDG---K----------YYCVPVYTMDIMK--------------------------GTLPNKAIEANKP----YSEWKEM------------------TEEYTFQ--FSLFPNDLVRIV-------------LPREKTIKT-
220930482	*Clostridium cellulolyticum* H10	IRS------IRVETP----SYTGV-------------FNEGKG--------ISDNSLMVRVDVFKKKD---K----------YYLVPIYVHDIAI--------------------------KELPSKAIVPLKP---ESQWELI------------------DSTHEFL--FSLYQNDYLVIKTKKG---------ITEGYYRSCH
297182908	uncultured delta proteobact. HF0070 07E19	AKQ-------RPILSKVTLLREKPEPP---------KQLTELSGG-----RRYDSMAQGRLDIYRYKP---GGKRKD---EYRVVLQRMIDLMR-------------------------GEENVHVFQKGVP---YDQGPEI------------------EQNYTFL--FSLYFDDLVEFQRSADSE------VIRGYYRTFN
154250555	*Parvibaculum lavamentivorans* DS-1	IRK------VRLTSK----QQLNL-------------MAQTGNG-------YADLGSNHHIAIYRLPD---GK--------ADFEIVSLFDASR-------------------------RLAQRNPIVQRTR------------------------ADGASFV--MSLAAGEAIMIPEG----------SKKGIWIVQG
218767588	*Neisseria meningitidis* Z2491	VKA------VRVEQV----QKTGV-------------WVRNHNG-------IADNATMVRVDVFEKGD---K----------YYLVPIYSWQVAK--------------------------GILPDRAVVQGKD---EEDWQLI------------------DDSFNFK--FSLHPNDLVEVITKKA---------RMFGYFASCH
15602992	*Pasteurella multocida* str. Pm70	VKA------IRVEQV----QKSGV-------------LVRENNG-------VADNASIVRTDVFIKNN---K----------FFLVPIYTWQVAK--------------------------GILPNKAIVAHKN---EDEWEEM------------------DEGAKFK--FSLFPNDLVELKTKKE---------YFFGYYIGLD
187736489	*Akkermansia muciniphila* ATCC BAA-835	---------LKALKA--------------------------------------AIEIDGNYGVALDPKPV-------------VIRHIKVFK--RIMALKEQNGGKPVRILKK----------GMLIHLTSSK
315605738	*Actinomyces* sp. oral taxon 180 str. F0310	FHH------ARLYRIIGPK--------------------EKV--------KYALLRVCAIDLCG--------------IDCDDLFEVELKP-----------------------SSISMRTADAKLKEAMG---------------------NGSAKQIGWLVLGDEIQIDPTKFPKQSIG----KFLKECGPVS
117929158	*Acidothermus cellulolyticus* 11B	FHH------ARVFRWGSSHSPSFALL-------------RVSLADL-----AVAGLLRDGVDVFTAELPPWTPA-------WRYASIALVKAVE-----------------------SGDAKQVGWLVPGDELDFGPEGVTTAA--------------GDLSMFLKYFPERHWVVTGFEDD----------KRINLKPAFL
189440764	*Bifidobacterium longum* DJO10A	IHH------ARVYRC------------------------WKTNAKGVRKY--FYGMIRVFQTDLLRACH---DDLFTVPLPPQSISMRYGEPRV------------------VQALQSGNAQYLGSLVVGDEIEMDFSSLDVD--------------VVDGFFKQFQFSFSGGNLAWKHW-----------VVDGFFKQ
403744858	*Alicyclobacillus hesperidum* URH17-3-68	VRK------VKIQEKLTL-------------------------------GV--PVHGGRGIAENG-GMVR----IDVF---AKGGK------YYFVPIY--------------VADVLKRELPNRLATAHKP----------------YSEWRVVDDSYQF---KFSLYPNDAV-------MIKPSREVD-ITYKDRKEPVGC
407803669	*Alcanivorax* sp. W11-5	LRK------VRCFYSKADDCV--------------------------------RI--EHSSRRGHHYKMLLNDG----FAYMEVPCKEGI------LYGVPNL---------------V------KSGVAIGIKRA--------------------PESGDF---I-RFYKGDTV--------KNIKTGRVY-TIK------
423317190	*Bergeyella zoohelcum* ATCC 43767	IST------VKIYYKDPSKNKKKKDEEDLSLQKL-------------DRE--KAFNEKLYVKTG-DNYL----FAVLEGEIKTKKTSQIKRLYDIISF------------FDATNFLKEEFRNAPDKKT---------FDKDLLFRQYFEERNKAKL---LFTLKQGDFVYLPNENEEVILDKESP-LYNQYWGDLKER
402847315	*Porphyromonas* sp. oral taxon 279 str. F0450	VRS------VRCYAKTLSLDKAIPMC------------------FD--EKGEPTAFVKSA-SNHH----LALY--RTPKGK------LVESIVT------------------FWDAVDRARYGIPLVITHPREVMEQVLQRGDIPEQVLSLLPPSDWVF---VDSLQQDEMV---------VIGLSDEEL-QRALEAQNYRKI
404487228	*Barnesiella intestinihominis* YIT 11860	IRT------VRCFAKPAINTLVPLK-----------------------KD--DKGNPVAWVNPG-SNHH----VAIY--RDEDGK------YKERTVT------------------FWEAVDRCRVGIPAIVTQPDTIWDNILQRNDISENVLESLPDVKWQF---VLSLQQNEMF-------ILGMNEEDY-RYAMDQQDYALL
374384763	*Odoribacter laneus* YIT 12061	IKK------IRMKEELGNA-----------------------------ER--LKDNINQYVNPR-NNHH----VMIY--QDADGN------LKEEIVS------------------FWSVIERQNQGQPI-------------------------YQLPREGRNI---VSILQINDTF-------LIGLKEEEP-EV--YRNDLSTL
384109266	*Treponema* sp. JC4	IKK------VRCVNRVQTPI----------------------------EI--TSGKISRYLSPE-DYFA----AVIWEIPGEKKT---FKAQYIRRNE--------------VEKNSKGLNVVKPAVLENG-------------------KPHPAAKQ---VCLLHKDDYL-------EFSDKGKMY-FCRIAGYAATNN
402849997	*Rhodovulum* sp. PH10	IRR------VRILKPDASVVTIA-------------------------DR--RTGVPYRAVAPG-ENHH----VDIV--QMRDGS------WRGFAAS------------------VFEVNRP------GW--------------------RPEWEVKKLGGKL---VMRLHKGDMV-------ELSDKDGQR-RVKVVQQIEISA
Jnet	331001027	-HHHHHHHHHH---HHHHHHH-----EEE----E-------------------------EEEEEE------EEEEEE--HHHHH-------------HHE-----------EEEEE------
331001027	*Parasutterella excrementihominis* YIT 11859	ENALRKALEAAWPSSFGTR--------------------------------NLHSKAKRVFSLPVVATQSGA---------------------VRIRRKTAFGDFVYQSQDTNN-LYSSFPVKNGKLDWSSPIIHPALQNRNLTA--------IYGRFVDHDRSIS-MS--EFREVYNKDD-----LMRIELAQGTS
34557932	*Wolinella succinogenes* DSM 1740	TQPIWDDFCRWYFLDRYKTAND-----------------------------KKSIRIKARKTFSLLAESGVQGK---------------------VFRAKRKIPTG-YAYQALPMDNNVIAG---DYANILLEANSKTLSLVPKSGISIEKQLDKKLDVIKKTDVRGLA-IDNNSFFNADFDTHGIRLIVENTSVKVGNF
54296138	*Legionella pneumophila* str. Paris	NPKEFNEFIRKYFLSDNNPNSDIPN-------------------------NGHNIKPQKHKAVRKVFSLPVIPGNAGT---------------------MMRIRRKDNKGQPLYQLQTIDDTPSMGIQHAEDRL-VKQEVLMDAYKTRNLST----------IDGINNSEGQAYATFD--NWLTLPVSTF--KPEIIKLEMKPHSK
319941583	*Sutterella wadsworthensis* 3 1 45B	ADELTSKLARIWKRPVMRD--------------------------------LAHAPVRREFSLPAIDNPSGG---------------------FRIRRTNLFGNELYQVHAINAKKYRGFASAGSNVDWSKGILFNELQHENLTE---------CGGRFITSADVTP-MS--EWRKVVAEDN------LSIWIAPGTE
254447899	gamma proteobact. HTCC5015	PQEIWEKVYRHFPRNIPN---------------------------------QAHRKVRKDFSLPVVDSVSGG---------------------FRVKRKTPNG-YNYQLLAIDGYSAVGFKKEGDNVDFKSPALVPQIAESKSVT------PISSELVHLDKNEIVY-FD--EWRKIDISDSDLKQFVSSLELAPGSQ
118497352	*Francisella novicida* U112	-----YEFLKSFFNVKSIT-------------------------------KLHKKVRKDFSLP-ISTNEGK----------------------FLVKRKTWDNNFIYQILNDSDSRADGTKPFIPAFDISKNEIVEAIIDSFTSKNIFWLPKNIELQKVDNKNIFA-IDTSKWFEVETPSDLRDIGIATIQYKIDNN

Motifs

informative positions

227494853	*Actinomyces coleocanis* DSM 15436#	HWTVAGFFSPSR--------------LRLRPVYLAQEGLGEDVSEGSKSIIAG-------------------QGWRPAVNKVFGSAMPEVIRRDGLGR-KRRFSYSGLPVSWQG------------
328956315	*Coriobacterium glomerans* PW2	G-------STNM--------------VNLSDIGGSKFAGNVRIKYKKELASPK-------------------------------VNVHLI-DQSVTGM-FERRTKIGL------------------
227824983	*Acidaminococcus* sp. D21	STFTKLSPEEQVQTL-----------LNILSIFKTCRSSGCDLKSINGSAQAA----------------RIMISADLTGLSKKYSDIRLV-EQSASGL-FVSKSQNLLEYL--------------
303229466	*Veillonella atypica* ACS-134-V-Col7a	--------EEQS--------------IYLLEVLNLLTNSKTTFDVKPLGITGS--------------------RSTIGVKIHNLDEFKII-NESITGL-YSNEVTIV-------------------
34762592	*Fusobacterium nucleatum* ATCC 49256	EKFKKLKLWEKS--------------LILREFLKIFNKNTYGKYEIKDSQTKE------------KLFSFPEDTGRIRLGQSSLGNNKELLEESVTGL-FVKKIKL--------------------
374307738	*Filifactor alocis* ATCC 35896	IKLEDLIDKINV--------------INKMLNLLRCDNDTKADLSLIELPKNA-------------------GSFVVKKNTIGKSKIILV-NQSVTGL-YENRREL--------------------
320528778	*Solobacterium moorei* F0204	EKFSELDILEQC--------------NILLQLLKAFKCNRESSNLEKLNNKKQ-------------------AGVIVIPHLFTKCSVFKVIHQSITGL-FEKEMDLLK------------------
291520705	*Coprococcus catus* GD-7	AKFIGLSNEDQC--------------IVLNEILHMFQCQSGSANLKLIGGPGS-------------------AGILVMNNNITACKQISVINQSPTGI-YEKEIDLIKL-----------------
42525843	*Treponema denticola* ATCC 35405	EKFKSLIIENQF--------------EVILEILKLFSATRNVSDLQHIGGSKY-------------------SGVAKIGNKISSLDNCILIYQSITGI-FEKRIDLLKV-----------------
304438954	*Peptoniphilus duerdenii* ATCC BAA-1640	DKFKEISIEEKI--------------DVLNQIILLFQSYNNGCNLKSIGLSAK-------------------TGVVFIP-KKLNYKECKLINQSITGL-FENEVDLLNL----------------
224543312	*Catenibacterium mitsuokai* DSM 15897	KEEKANIIKQML--------------IVMHRGPQNGNIVYDDFKISDRIGRLK--------------------------TKNHNLNNIVFISQSPTGI-YTKKYKL-------------------
116628213	*Streptococcus thermophilus* LMD-9	QNHSIDELCSSFIGPTGSERK------GLFELTSRGSAADFEFLGVKIPRYRDY-------------------------TPSSLLKDATLIHQSVTGL-YETRIDLAKLGEG--------------
24379809	*Streptococcus mutans* UA159	NGEDLKELASSF--------------INLLTFTAIGAPATFKFFDKNIDRKRY--------------------------TSTTEILNATLIHQSITGL-YETRIDLNKLGGD--------------
13622193	*Streptococcus pyogenes* SF370	RDKPIREQAENI--------------IHLFTLTNLGAPAAFKYFDTTIDRKRY--------------------------TSTKEVLDATLIHQSITGL-YETRIDLSQLGGD--------------
310286728	*Bifidobacterium bifidum* S17	KTANLSIVGMTG---SWRRMNNKSGYTFSDEDEFIFQSPSGL-FEKRVTVGELKRKAKKEVNSKYRTNEK
366983953	*Oenococcus kitaharae* DSM 17330	ASSDLKKINLSS---GFGRLFSPSHFTLADTDEFIFQSVTGL-FSTQKTVAQLYQETK------------
422884106	*Streptococcus sanguinis* SK49	KSGAPKDINDFFDNKCTV---ARMRPKPDKKL-LNATLIHQSITGL-YETRIDLSKLGED--------------
339625081	*Fructobacillus fructosus* KCTC 3544	AYQAPVKIISK-KVSDW--HKLQQSGGIKLSDNSEMIYQSATGI-FETRVKISDLL----------------
306821691	*Eubacterium yurii* ATCC 43715	E----VDLSDIGGSKST--GKCRCKKNITNYKEFKLIQQSITGL-YSCEKDLMTI-----------------
336394882	*Lactobacillus farciminis* KCTC 3681	T-SAHLIFNNI-EKKAFG---RK---THGLTL-NNTDFIYQSVTGL-YETRIHIE-------------------
323463801	*Staphylococcus pseudintermedius* ED99	TRSD--------KIGSRK---NSMTHRAFLGKGKDVKIAYTSISGLKTTKPKSLFKLAESRNEL----------
389815359	*Planococcus antarcticus* DSM 14505	Q-----------RSDTFG---SRISKKP---KPEEVAIGYESITGLKYRKPRSVVGTKR---------------
258509199	*Lactobacillus rhamnosus* GG	INTDGNKIGKTE--------------TLRQILIGLQANGTRSNV-KNLGIKTD-------------------LGLLQVGSGIKLDKDTQIVYQSPSGL-FKRRIPLADL----------------
169823755	*Finegoldia magna* ATCC 29328	DTLDNKQLYQLL--------------VAFISLSYKTSNNAVDFTVIGLGTECG-----------------------KPRITNLPDNTYLVYKSITGI-YEKRIRIK------------------
Jnet	227501312	-------HHHHH--------------HHHHHH-H--------EEEE------E-----------------------EEEE------EEEEE--------EEEEEE--------------------
47458868	*Mycoplasma mobile* 163K	DE------KPQK--------------LEIKYILGKKIKDQKQIQKPVKKYFPN---------------------------------WKKV-NLTYMGE-IFKK----------------------
284931710	*Mycoplasma gallisepticum* str. F	N-------LNDV--------------IEIKNLIETEYKENDDSDSKKKKKANR-------------------FLMTLSTILNDYILLDAKDNFDILGL-SKNRIDEILNSKLGLDKIVK-------
71894592	*Mycoplasma synoviae* 53	PGALLLNFENQQ--------------IYYISTLESSSLRAKIKLLNKMDKGKA----------------------VSMKKITNPDEYKIIEHVNPLGI-NLNWTKKLENNN--------------
363542550	*Mycoplasma ovipneumoniae* SC01	KGSILKKKSLDIDDFKETKETEEGN-YYFISTISKRFNRDTAYGLKPLKLSVVKPV-------------------AEPSTNPIFKEYIPI-HLDELGN-EYPVKIKEHTDDEKLMCTIK-------
384393286	*Mycoplasma canis* PG 14	PGTILLDKQNKE--------------FYYISSIQTVRDIIEIKFLNKIEFKDENKN----------QDTSKTPKRLMFGIKSIMNNYEQV-DISPFGI-NKKIFE--------------------
238924075	*Eubacterium rectale* ATCC 33656	LVSRTMPKQRNY--------------IETKPIDKAKFEKQNLV-----------------------------------GLGKTKFIKKY-RYDILGN-KYSCSEEKFTSFC--------------
315149830	*Enterococcus faecalis* TX0012	NFQ-----SANS--------------INFKGLEQEMMPAENQFKQKPYNNGAI--------------------NLNIAKYGKEGKKLRKF-NTDILGK-KHYLFYEKEPKNIIK------------
116627542	*Streptococcus thermophilus* LMD-9	RTMPK---QKHY--------------VELKPYDKQKFEGGEALIKVLGNVANS--------------------GQCKKGLGKSNISIYKV-RTDVLGN-QHIIKNEGDKPKLDF------------
315659848	*Staphylococcus lugdunensis* M23590	SD------TRNM--------------IELDLPDIRYKEYCELNNIKGEPRIKK--------------------------TIGKKVNSIEKL-TTDVLGN-VFTNTQYTKPQLLFKRGN---------
160915782	*Eubacterium dolichum* DSM 3991	HNFNFFFHAGET--------------PEILKFTATNNDKSNKIEVKPIHCYCK--------------------MRLMPTISKKIVRIDKY-ATDVVGN-LYKVKKNTLKFEFD-------------
325677756	*Ruminococcus albus* 8	NV------NENS--------------FRFISDNKPCAKDKRVSITKKDIVIKL--------------------AVDLMGKVQGENNGKGISCGEPLSL-LKEKN---------------------
225377804	*Roseburia inulinivorans* DSM 16841	LVNGGLLLQ-----------------KEIFAYYTGADIATASIAGFANDSNFKF----------------------RGLGIQSLEIFEKC-QVDILGN-ISVVRHENRQEFH--------------
336393381	*Lactobacillus coryniformis* KCTC 3535	NNTSVVPIKNFY--------------GYFDGADIATASILVRAHDSSYTARSI--------------------------GIAGLLKFEKY-QVDYFGR-YHKVHEKKRQLFVKRDE----------
310780384	*Ilyobacter polytropus* DSM 2926	KLTKTEIPEVNL--------------SEVLGYYRGMDTSTGAATIDTQDGKIQ----------------------MRIGIKTVKNIKKY-QVDVLGN-VYKVKREKRQTF--------------
301311869	*Bacteroides* sp. 20 3	TANLIFALPKAT--------------AEIYCNGENCIQNEYGIGSPQSKNQKA-----------------------ITGEMVKEICFPI-KVDRLGN-IIQVGSCILTN----------------
385811609	*Ignavibacterium album* JCM 16511	IASLILSYSASN--------------GVGEFGSQNI--SEYSVDDPPIR---------------------------------IKDVCIKI-RVDRLGN-VRPL----------------------
60683389	*Bacteroides fragilis* NCTC 9343	NLFRVQKFSLKN--------------YVFRHHLETTIKDTSSILRGITWIDFR--------------------------SSKGLDTIVKV-RVNHIGQ-IVSVGEY-------------------
319957206	*Nitratifractor salsuginis* DSM 16511	QGSKYIALFNPR--------------FPANPKAQPSKFTSDSKIKQVGIGSAT-------------------GIIKAHLDLDGHVRSYEVFGTLPEGS-IEWFKEESGYGRVEDDPHH--------
187250660	*Elusimicrobium minutum* Pei191	NNTGSAPENRVF--------------IKVGTFTEITNNNIQIWFGNIIKSTGG-------------------QDDSFTINSMQQYNPRKL-ILSSCGF-IKYRSPILKNKEG-------------
325972003	*Sphaerochaeta globus* str. Buddy	VRLPNAKPGRTF--------------VIIITFTEMGSGYQIYFSNLAKSKKGQ--------------------DTSFTLTTIKNYDVRKV-QLSSAGL-VRYVSPLLVDKIEKDEVALCGE-----
296446027	*Methylosinus trichosporium* OB3b	R-------SVGA--------------IQLSAHSNSSDVVQGI--------------------------------------GARTLTEFKKF-NVDRFGR-KHEVERELRTWRGETWRGKAYI-----
347536497	*Flavobacterium branchiophilum* FL-15	RIYIIEGLSIQRIV-RPSGKVDEYGVIMLRYFKEARKAD-DIKQDNFKPDGVFKLG-----------------------ENKPTRKMNHN-QFTAFVEGIDFKVLPSGK--FEKI-----------
345885718	*Prevotella* sp. C561	RLYKINRFESQ-------------SNLVLMTHHLSTSKE---RGRSLGKTVDYQNL-----------------------PESIRSSVKSL-NFLIMGENRDFVIKN-GKIIFNHR-----------
282880052	*Prevotella timonensis* CRIS 5C-B1	RLYKVVGIEKD-------------GRIKFKYHQEARKEGLPIFSTPYKNNDDY--------------------------APIFRQSINNI-NILVDGI--DFTIDILGKVTLKE------------
312879015	*Aminomonas paucivorans* DSM 12260	R-------GDGR--------------LLLDRHDRMPRESDCGTFYVS---------------------------------TRKDVLSMSKY-QVDPLGE-IRLVGSEKPPFVL--------------
294086111	*Candidatus Puniceispirillum marinum* IMCC1322	L-------SGSK--------------ILMAPHTEANVDARDRDKNDTFKLTSK------------------------SPGKLQSASARKV-HISPTGL-IREG----------------------
330822845	*Alicycliphilus denitrificans* K601	IS-----GSNGQ--------------IFMAPIHEANVDARNTDKQDAFTYTSKYA-----------------------GSLQKAKTRRV-TISPIGE-VRDPGFKG------------------
344171927	*Ralstonia syzygii* R24	M-------SENK--------------ITLAEHFEGGTLKQRDADKDDPFKYLT----------------------KSPGALRDLGARRI-FVDLIGR-VLDPGIKGD-----------------
159042956	*Dinoroseobacter shibae* DFL 12	IANG----------------------LFIVPHNEANADTRNNDKSDPFKWIQI------------------------GARPAIASGIRRV-SVDEIGR-LRDGGTRPI-----------------
83591793	*Rhodospirillum rubrum* ATCC 11170	VDGRLALWPARL--------------ATGKATALYAQLSCPNINLNGDQGYCV----------------------QSAEGIRKEKIRTT-SCTALGR-LRLSKKAT------------------
288957741	*Azospirillum* sp. B510	LEP-----SSNS--------------VVVVEPHQVKTDRSKHVKISC---------------------------------DQLRARGARRV-TVDPLGR-VRVHAPGARVGIGGDAGRTAMEPAEDI
427429481	*Caenispirillum salinarum* AK4	---VCDLEVAKGD-TVKKDGKTYKVGV--ITQGIFGMPVDAAGSAPRTP
92109262	*Nitrobacter hamburgensis* X14	LDA-----AAGR--------------FKLADHNETGNLDKRHATNNDIDPFRW---------------------LMASYNTLKKLAAVPV-RVDELGR-VWRVMPN-------------------
148255343	*Bradyrhizobium* sp. BTAi1	LSP-----SNNL--------------LYLVEHHQAGVFQTRHDDPEDSF----------------------RWLFASFDKLREWNAELV-RIDTLGQ-PWRRKRGLETGSEDATRIGWTRPKKWP
34557790	*Wolinella succinogenes* DSM 1740	GANMVLSSINNSPCEGFTCT-----PVSMDKKHKDKCKLCPEENRIAGRCLQGFLDYWSQ-----EGLRPPRKEFECDQGVKFALDVKKY-QIDPLGY-YYEVKQEKRLGTIPQMRSAKKLVKK--
218563121	*Campylobacter jejuni* NCTC 11168	SSTVSLIVSKHD--------------NKFETLSKNQKILFKNANEKEVIAKSI----------------------GIQNLKVFEKY-IVSALGE-VTKAEFRQREDFKK------------
Jnet	218563121	---EEEEEEE----------------------HHHHH---------EEEEE--------------------------------EEEEEE-EEE-----EEE----------------------
291276265	*Helicobacter mustelae* 12198	KSTNSAKATIEL--------------EHLSKYALKNEDEEKMFTDTDKEKNKT--------------------MTRESCGIQGLKVFQKV-KLSVLGE-VLEHKPRNRQNIALKTTPKHV------
222109285	*Acidovorax ebreus* TPSY	R-------STGA--------------MNLWAHDRAASVGKDGLIRGI---------------------------------GVKTALSVEKF-NVDVLGR-IYLAPPETRSGLA-------------
365156657	*Bacillus smithii* 7 3 47FAA	-S------TNEE--------------IIIKDIFAYYKTIDSATGGLELISHDR-------------------NFSLRGVGSKTLKRFEKY-QVDVLGN-IHKVKGEKRVGLAAPTNQKKGKTVDSL
220930482	*Clostridium cellulolyticum* H10	R-------GTGS--------------LSLMPHFANNKNVKIDI-------------------------------------GVRTAISIEKY-NVDILGN-KSIVKGEPRRGMEKYNSFKSN------
297182908	uncultured delta proteobact. HF0070 07E19	I-------ANGQ--------------LKISTYLEGRQDFDFF--------------------------------------GANRLAHFAKV-QVNLLGK-VIK-----------------------
154250555	*Parvibaculum lavamentivorans* DS-1	VW------ASGQ--------------VVLERDTDADHSTTTRPMP-----------------------------------NPILKDDAKKV-SIDPIGR-VRPSND-------------------
218767588	*Neisseria meningitidis* Z2491	R-------GTGN--------------INIRIHDLDHKIGKNGILEGI---------------------------------GVKTALSFQKY-QIDELGK-EIRPCRLKKRPPVR------------
15602992	*Pasteurella multocida* str. Pm70	R-------ATGN--------------ISLKEHDGEISKGKDGVYR-V---------------------------------GVKLALSFEKY-QVDELGK-NRQICRPQQRQPVR------------
187736489	*Akkermansia muciniphila* ATCC BAA-835	DP------KHAG--------------VWRIESIQDSKGGVKLDLQRAHCAVPK--------------------------NKTHECNWREVDLISLLKK-YQMKRYPTSYTGTPR------------
315605738	*Actinomyces* sp. oral taxon 180 str. F0310	SWRVSALDTPSK--------------ITLKPRLLSNEPLLKTSRVGGHESDLVVAECVEKIMKK--------TGWVVEINALCQSGLIRVIRRNALGE-VRTSPKSGLPISLNLR-----------
117929158	*Acidothermus cellulolyticus* 11B	SAEQAEVLRTER--------------SDRPDTLTEAGEILAQFFPRCWRATVA--------------------------KVLCHPGLTVI-RRTALGQ-PRWRRGHLPYSWRPWSADPWSGGTP--
189440764	*Bifidobacterium longum* DJO10A	LRIRPRYLAAEG--------------LAKAFSDDVVPDGVQKIVTKQGWLPPV--------------------------NTASKTAVRIV-RRNAFGE-PRLSSAHHMPCSWQWRHE---------
403744858	*Alicyclobacillus hesperidum* URH17-3-68	RIM-----------------YFVSANIASASIS----------LRTHDNSGELEGL-----------------------GIQGLEVFEKY-VVGPLGD-THPVYKERRMPFRVERKMN--------
407803669	*Alcanivorax* sp. W11-5	--------------------QILGDGGGKLILTP---VTETKPADL-LSAKWGR-LKVGGRNIHLLRLCAE-----------
423317190	*Bergeyella zoohelcum* ATCC 43767	GKNIYVVQKFSKKQ-----IYFIKHTIADIIKK----------DVEFGSQNCYETV--------------------EGRSIK--ENCFKL-EIDRLGN-IVKVIKR------------------
402847315	*Porphyromonas* sp. oral taxon 279 str. F0450	SEHLYRVQKMSSSY------YVFRYHLETSVAD---DKNTSGRIPKFHRVQSLKAY-----------------------EER---NIRKV-RVDLLGR-ISLL----------------------
404487228	*Barnesiella intestinihominis* YIT 11860	NKYLYRVQKLSKSD------YSFRYHTETSVED---KYDGKPNLKLSMQMGKLKRV-------------------SIKSLLG--LNPHKV-HISVLGE-IKEIS---------------------
374384763	*Odoribacter laneus* YIT 12061	SKHLYRVQKLSGMY------YTFRHHLASTLNN----------EREEFRIQSLEAW-----------------------KRA---NPVKV-QIDEIGR-ITFLNGPLC-----------------
384109266	*Treponema* sp. JC4	KLDIRPV-------------YAVSYCADWINSTN--ETMLTGYWKPTPTQNWVSVN-----------------------VLFDKQKARLV-TVSPIGR-VFRK----------------------
402849997	*Rhodovulum* sp. PH10	NRV-----------------RLSPHNDGGKLQD----RHADADDPFRWDLATIPLL-----------------------KDR---GCVAV-RVDPIGV-VTLRRSNV-----------------
Jnet	331001027	--EEEEEE--HHHHHHHH------------EE-------------------EEHHHHHH------------------------------EE-EEEEE----EEEEEEEE--HHHHHHHHHHHH----
331001027	*Parasutterella excrementihominis* YIT 11859	SRRYLRVEMPGEKFLAWFGE--------NSISLGSSFKFSVSEVFD--NKIYTENAEFTKFLPKPREDNKHN---------------GTI-FFELVG--PRVIFNYIVGGAASSLKEIFSEAGKER
34557932	*Wolinella succinogenes* DSM 1740	PISAIDKSAKRMIFRALFEKEKGKRKKKTTISFKESGPVQDYLKVF-LKKI-------------------------------------VKI-QLRTDGSISNIVVRKNAADFTLSFRSEHIQKLLK-
54296138	*Legionella pneumophila* str. Paris	TRRYIRITQSLADFIKTIDE---------ALMIKPSDSIDDPLNMP--NEIVCKNKLFGNELKPRD--------------------GKM-KIVSTG--KIVTYEFESDSTPQWIQTLYVTQLKKQ
319941583	*Sutterella wadsworthensis* 3 1 45B	GRRYVRVETTFIQASHWFEQSVENWAITSPLSLPASFKVDKPAEFQ--KAV---GTELSELLGQPR---------------------SEI-FIENVGNAKHIRFWYIVVSSNKKMNESYNNVSKS-
254447899	gamma proteobact. HTCC5015	NRFYIRFTVDEDQFERHF---------------KSALRVNGIQDLDTVNKTFDWNREIPSLLIPPR---------------------SNL-FLLETG--QKITFEYIANGANAEVKKAYSLRRA--
118497352	*Francisella novicida* U112	SRPKVRVKLDYVIDDDS--------------------KINYFMNHSLLKSRY------------PD---------------------KVL-EILK----QSTIIEFESSGFNKTIKEMLGMKLAGI

Motifs ____G______________________________

informative positions --***************------------------------

```
16130662   Escherichia coli str. K-12 substr. MG1655          ---------------------------------------MTWLPLNPIPLKDRVSMIFLQYGQIDVIDGAFVLIDKT-------------------GIRTHIPVGSVACIMLE-PGTRVSHAAVRLAAQVGTLLVWVGEAGVRVYASGQPGG-ARSDKLLYQAKLALDEDLRLKV-VRKMFELRFGEPAP----------
11500011   Archaeoglobus fulgidus DSM 4304                    ---------------------------------------MRK---------KNYYLVSDG-KLRRHENTIYFENE-------------------DGKRPIPINSIYAIYALG-SLSITSKAISLLAKEGVCIHFFNRYGYYIGSFYPRESLVSGEVVLRQAEHHLDSEKRLHL-ARAFVEGAILNMARVLKK--AEQD
18977490   Pyrococcus furiosus DSM 3638                       ---------------------------------------MRK---------KSLTIFSDG-TLLRRENTLYFENV-------------------NGRKPLAIEGIYDIYIYG-HVNITSQALHYIAQKGILIHFFNHYGYYDGTFYPRETLLSGDLIIKQAEHYLDKNKRLFL-AKSFVVGGAKNMEKNLKN--WGIS
46447796   Desulfovibrio vulgaris str. Hildenborough          ------------------------------------MKKLL---------NTLYVTTQGTYLAKEGECIVVRVGD-------------------EVRLRVPVHSLGGVVCFG-QVSCSPFLMGFAAERGLGFSFLTEHGRFLARVQGPVS-GNVLLRREQYRRADSPEASAEV-ARSIVSAKVVNARGVLQR--AMRD
54296139   Legionella pneumophila str. Paris                  ------------------------------------------M---------KHLTLSEQGSFCKLQGERILVFNKD-------------------QMVFEGTLNRLKTISIVKQGVTLTSNLLVACAMRGIQIFILDYSGKAVCSLQG--G-LHRTAKVRESQFKYIYTKEASNMAARFILAKIKKQSAVLKY--FAKA
331001028  Parasutterella excrementihominis YIT 11859         ------------------------------------------M---------RHLTVSGFGDFLGVHGNRLIVRNGD-------------------NVILETALSRLRTIRID-KSVLSSNLILACAARGIRIYFLDWKGVGVVESGMH--RHAVVGVRKSQFECLKSLQAQLLAKEIIFSKIRNQRAVLLY--FSKY
319941582  Sutterella wadsworthensis 3 1 45B                  ------------------------------------------M---------RYLTVSSFGTALGLSGERLVVHEAD-------------------GRTWETALSRLRTIRIEKKGISVSTNLMLACAARGIGIFLLVDWRGVGVVALSGLHQ-HAVVSIREAQFLAIHSSQGRTI-AAEMIIAKIRNQRTVLLY--FWKY
118497353  Francisella novicida U112                          ------------------------------------------M---------KHLIISEYGIYLGLESGRLVVKNK-------------------DDKKYFPLNRLATLSIAKKGVSFSSDLVEQFSLRGIKLFFLDFRGVAHSMLVGANQ-HAVVQARI-NQYRYIDRNALTL-SIKLIAAKIKNQRATLNY--FNKH
34557933   Wolinella succinogenes DSM 1740                    -----------------------------------------MT---------KHLYIDEYGSFVGKTSERLTVSQGG-------------------ELKAEIPLSRLKSVNILKGGISLSSDIEACSLRGIKIFFLDFRNVAHTAITSTAN-HAVANVRKAQFLTIESNHGREI-ARTLIVGKLKNQRATLQYFYKYLK
187736488  Akkermansia muciniphila ATCC BAA-835               ---------------------------------------MSY---------HILSIDAYTCHLSCDKGQLRCADGE-------------------NSPRTIPLEDVGAVVLSSFKATLTSNLLIELARKRIGFVLCESYR-PAVLLLPAD--RSTDTGLLRHLADMPARLRNRL-WQKTLDAKCGNQTALAQA--WNPH
407803668  Alcanivorax sp. W11-5                              --------------------------------------MSEQ---------HILLVESPC-ALNIHLECLKVSRKG-------------------MEPLRLLPSDIAVLCLHHHTVQLSVHVLRKLTEAGAAILVTDDRHQPAGLMLQDYG-RPNAVVRLRQQIALEQSGQDRLVWQQLVSWKLRTQSRLLGK--LERR
325972002  Sphaerochaeta globus str. Buddy                    ----------------------------------------MW---------RVIDLGGTGYYLHTVHNKLIVEKD-------------------GEESLIPFDDIHSIICHGIGFRYSDKFFKSCLHHKVPVTFCDEKHIPLGMLLPMLQ-HKDTSQRMDIQLKASLPRRKQA-WQQIITEKLKNQRDFLQH--LQIP
187250661  Elusimicrobium minutum Pei191                      ----------------------------------------MW---------RVLDIPGDGYHLCVKNNNFSAVKDR-------------------EEKLHCLFDDINSIILYGNNITISNTCIQKCLEHKVPVIFCDKTYNPAGMLLSSFT-TNIYGRRLQLQINASKPQIKQA-WQQIITSKLNNQAEVLKR--FDTL
47458867   Mycoplasma mobile 163K                             ---------------------------------------MGW---------KIVEINTDE-YVHLYLNNLYIKRK-------------------NEKILLNIRDIDTILFNNQYSTISIRLLTFLAKNNVNIIFMNEKNEPNSYLIPIEG-NHNSLKVVAEQVKWTKEYKAIL-WKDIIKNKIHNQKNLLIK--NKLF
363542551  Mycoplasma ovipneumoniae SC01                      -----------------------------------------MK---------KIIEISESE-YVYLFLNNIVIKKD-------------------SEKFVFPIDTVDVLIFENDRATISIPVINELVEKKVNIIICKNHL-PQSLIIPYSG-YYNN-KIFQEQIKWDIPYKTKT-WQEIIKLKIQRSISVLKS--IQKI
71894593   Mycoplasma synoviae 53                             --------------------------------------MMAK---------KVVEIKETE-YLFFHLNSLIVKKE-------------------NNKIVLPLSDIDTILISNPYCTISVPLINAIVSNNINLIICNKDFEPNVQLLSISG-YYSN-KNFLSQINWTQEFKDKT-WEKIIKLKTTNYVNLIYF--FGLL
384393287  Mycoplasma canis PG 14                             ----------------------------------------MSK---------KVLEISQSE-YISYFLNSLIVKSN-------------------EKRIIVPISQIDTVLVSNPRCNISVPLLNELVAQNVNIIICDSKFKPTVQISPIQG-YYSN-KNFLNQINWSKDFKGFV-WREIIKTKIVNYTKLIYY--LDIT
284931711  Mycoplasma gallisepticum str. F                    ---------------------MQLPHPPEHLCSGFLFIMSK---------KVLEVSETE-FISLHLDTLIVRKH-------------------GARVTLPLKTLDTILITNPYCNISVPLLNKIVENNINLIVCNTKFQPTMQILPISS-YYSN-KNYLWQIRWTDEQKNQI-WKQLVKLKIINYARLINH--LNIT
117929157  Acidothermus cellulolyticus 11B                    -------------------------------------MTGPW---------RVVDLSELSGEVHAAQGALLVGDE-----------------------RVPLVDVA-MMLTGPYVSLHGSVIDRAAAFGVGVVHCDWRGVPVAATLPWST-HNRVAARHRAQAELSLPRQKNA-WMNIVKTKIRNQAAVLRA--LRRD
317482065  Bifidobacterium sp. 12 1 47BFAA                    -------------------------------------MQNQW---------RVIDCTDLDGSISYERGRMRVVKNDT-----------------GECVDVPLAQIAVVLL-GLHVRCSAAVLHEMARYGVSVMLCDWRGVPDAALHAWTDMPTTVTTRQLAQSNMSLPRKKSA-WARIVQMKIRGQAACLDS--SKLE
315605739  Actinomyces sp. oral taxon 180 str. F0310          -------------------------------------MNSQW---------RVIDLCGFEGTLRSTRGGVEVCAQE-------------------GLPTTIPVAELAVVLV-GMKVNLSAAVLHRLAEADVAVLFCDWRGIPEGGCYSWSD-HGRVAARHRAQAEVTLPRKKNA-WARLIRAKIEGQASVLEN--IKIR
294086112  Candidatus Puniceispirillum marinum IMCC1322       --------------------------------------------------MIEISGQPRHISVRRGAIIVSDNE-------------------TELGQIDLDGVLSVIVTSRGATVTTPMITEAAARNIPIIICNDRFQPVSLSLPLVQ-HSDQTRRFETQALAKKGIKNRI-WQKIIKGKVKNQATLLVL--YQSA
282880053  Prevotella timonensis CRIS 5C-B1                   ---------------------------------------MLK---------RSVVFSHPA-SLSLRYNQLVITMKDLP-----------------DERRTIPIEDIGFLMVDNPMVSISVPLINALTENNVSVVFCNQKGMPTSMLFNLDN-NNTQGETLRNQLSASQSFNKQL-WKQIIECKIRNQAQLLEK--LGKE
374384762  Odoribacter laneus YIT 12061                       ---------------------------------------MLK---------RTLFFGKPS-RLSVKDKQLVIEIKET-----------------GELHKAPIEDIGFVVIENQQISVSLPLLQEFMSNNVAVIFCDASHMPNSMLLNLDG-HHLQSEMFRFQINASEPLKKNL-WKQTVEAKIRNQAALLDK--SGKN
423317188  Bergeyella zoohelcum ATCC 43767                    ---------------------------------------MLY---------RSIYIGNPA-YLKLKDQQMKIVCPETK-----------------AEKGSVPVEDLGLLMLDHFQITISHQLIQWLMGNNVVIISCDAHHLPHGQMLPLHG-NAIYSQRIKDQIEASEPLKKQL-WKQTIECKIANQAAVLER--LGTF
385811610  Ignavibacterium album JCM 16511                    ---------------------------------------MIK---------RTLYFGNPA-YLSLKNMQMLIKLPISGNDNEATDEDKNEKYKDRNNQNTFPIEDIGIVILDNQQITITQGLLSSLLDNNSAIVTCDSRHHPAGLFLPLES-NLEQSQRFQEQIEASVPLKKQL-WQQTISSKIINQALLLNK--IGVK
402847305  Porphyromonas sp. oral taxon 279 str. F0450        ---------------------------------------MMK---------RTIYIGNPA-YLSLRLKQMEVRQPLDDRE---------------TTVRTIPIEDIGVVLLDHPQITITHALLGALLENNCAVIVCSATHMPSGLLLPLDG-HTLQTERFRAQLDASLPLKKQL-WQQTVQAKILNQAHALHT--VLGE
345885719  Prevotella sp. C561                                ---------------------------------------MIK---------KTLYFGNPA-YLSLRNGQLVIRLPEVEKAE-LPDIIK------QETIRTVPIEDIGVVVLDNKQITITQGALAELVANSAAVITCDSKCMPTGMLLPLSG-NTLHQERFRNQIEAAIPLRKQL-WQQTVKAKIENQAYCLQK--NTSK
404487227  Barnesiella intestinihominis YIT 11860             ---------------------------------------MIK---------RTLYFGNPS-YLSLRNAQLVLRLPEVSNNDTLPDTFK------KEAERTIPIEDIGVVVLDNKQITITQGLLEALLENNCAIITCDKSRLPVGLILPLCG-NTTQNERFRDQLNASLPLKKQL-WQQTVQQKIANQAAILES--QGII
301311870  Bacteroides sp. 20 3                               ---------------------------------------MIK---------KTFYFGNPA-YLSLRNQQLVIKLPEIVKNDTVPESFK------RQSEITKPIEDIAVIVLDNKQITITQGVLEALLENNCAVITCDGRSMPVGLMLPLYG-NTTQNERFRDQLDASLPLKKQL-WQQTIQAKINNQASVLCS--CKNE
60683388   Bacteroides fragilis NCTC 9343                     ---------------------------------------MIK---------KTLYFGNPV-YLSLRNAQLVIKLPDVEKATVLPETLK------KQAEVTKPIEDIAVIVLDNKQITITSGVLEALLENNCSVITCDSRSMPVGLMLPLYG-NTTQNERFRNQLDTSLPLKKQL-WQQTIQAKINNQASVLKD--CMDE
402849996  Rhodovulum sp. PH10                                ---------------------------------------MLG---------RIIEVEGEGRRLCLERGFLRITGPA-------------------GLLGKIPLDDIEAVILTHPAASLTTQAVAALAARGTPLVVSGPDFRPVAFLLPIDG-HHGQGHRLEAQAEASRPTRKRV-WAEIVRAKVTAQADMVEH--VGGA
154250554  Parvibaculum lavamentivorans DS-1                  ---------------------------------------MIR---------KTVEFATPGTRLSVANKQLVIERPD-------------------LPKATLPIEDLGVVIVDDLRATYTQAVFIELLEAGATVMVTGRDHLPAGMMLPLDA-HHIQTERHRAQVEASEPTKKRA-WQALIRSKIAQQGIVLAH--FTGE
159042957  Dinoroseobacter shibae DFL 12                      -----------------------------------------MD---------QIVDIATDGRHLSRDRGFLKVSEGA-------------------REIGRIPLDQIAGVIVHAHGTTWTTSLLTELADRGAPVVLCGANHAPRSVLMPLDG-HHAQGARLRAQWQARAPLVKQA-WKQTVIAKIAMQAAALEA--MGEP
288957740  Azospirillum sp. B510                              ---------------------------------------MTG---------RIVEIAEDGRHLSLSRGFLVVTAEG-------------------RELGRVPLDDIAVVMANAHGLTYSNALLMALMERGAVVVMCGPNHLPAAFVWPVDA-HHVQSRRMRAQVDAPAPLAKRL-WQALVRAKIRQQAAALEA--RGLP
330822846  Alicycliphilus denitrificans K601                  ---------------------------------------MIG---------RIVEIADDRRHLFVNRGFLVVRDTEGER----------------KELGQVPLDDIAAVIANAHGLTYTNNLTLVALAERGAPFVLCGPNHNAMGMLLPIDG-HHVQAKRIEAQIAASLPVHKRL-WAAVVKSKLEQQAAALEA--AAAP
344171926  Ralstonia syzygii R24                              ---------------------------------------MIG---------RIVEIATNNRYLGLDRGFMVVKSAD-------------------EEVGRVPLDDLAAVIVAHANHGITHSSNLLVALAERGVPFVLTAANMQPVAMLWSVDG-NYQQAGRMEAQLGASLPQNKRL-WQQVVRSKLQMQAVVLES--VGVP
319957207  Nitratifractor salsuginis DSM 16511                --------------------------------------MAAW---------KTIRITKPC-RLSIENGNLVVEDE-------------------KVRFKLSLSDTDSIIFEGDRFTLSAKVLAALSRHKVATLFCDEYYMPTAILHPYHQ-SSLATETLKAQLSLPDDLRDRL-WQEIIRVKILNQSAVLSH--FAKK
315149831  Enterococcus faecalis TX0012                       -------------------------------------MLLGF---------RTVFVKNGE-KLKVKLDNLEVIKE-------------------GNTYVIPLTDIESVILEGDQTVITTRLLAKFAQHHIDTVICDNTFMPVGVFLGIGQ-YHRSAKRAIWQSNLKEEQKQVA-WCEIVTQKIQNQIAVAKY--LGTD
116627543  Streptococcus thermophilus LMD-9                   ---------------------------------------MTW---------RVVHVSQSE-KMRLKLDNLLVQKM-------------------GQEFTVPLSDISIIVAEGGDTVVTLRLLSALSKYNIALVVCDNEHLPTGIYHSQNG-HFRAYKRLKEQLDWSQKQKDKA-WQIVTYYKINNQEDVLAM--FEKS
238924076  Eubacterium rectale ATCC 33656                     ---------------------------------------MAF---------RVILIENEV-TIKVKLNNLIITKC-------------------GEDIWIPLDDISMIVLDNLASNLSTRLMCQLAEQGIGLMLYNQQHLPTGFYSAYDN-HSRASKVIGFQIDKEQDYYGKL-WQQIVKIKIENQAKAYQI--MTRD
218563120  Campylobacter jejuni NCTC 11168                    ----------------------------------MSYDEAF---------KTLLISSNA-KLNLELNHLVIKQD-------------------ENIAKLFLKDINIIVLESLQISISSALFNAFAKYKIILLTCDETHSINGVFTPFLG-HFQSAIKAKEQMNVSAQKKAIL-WQKIIKNKILNQAFILKK--HNKI
291276264  Helicobacter mustelae 12198                        ------------------------------------MFDEAF---------RTLFMSKPA-KLSHKNNNLIIHGK-------------------EDIG-IPLRDHTIVIDTPQITLTSSLMKELAQYKILLVFCDDSHLPSGVFSPFLA-HYRSSAILDLQIHFSKQNKSIL-WQKIIQAKIQNQTNLLMQ--ENKT
325677757  Ruminococcus albus 8                               ----------------------------------------MW---------RTVIVTKGE-KLTLRDNWLVVYSD-------------------NNEQCVPLGDIYSVVIDNRAALVSVSLMAALAEANIHVIFCDSKHTPTALMLPMNT-HYKPFGVIKRQLGLSDEFKAVL-WQRIVKQKLTNQIICLKL--AGIN
296446028  Methylosinus trichosporium OB3b                   ---------------------------------------MAW---------KGLHLSRPA-RLNFSDNQIVVAQD-------------------DGEARLPLEDVAYIILDAPHATLTSTLISACMEAGVVIVSVDARHTPNGLALPFHS-HHRQAGVAAKQVLLSEPFKKRC-WQGVVVAKIDNQAAHLES--LGRN
34557789   Wolinella succinogenes DSM 1740                    --------------------------------------MAAW---------RTVVIATSC-HLRVRDRQLVIESRE-------------------GERTQLPLADIGVVVENPQVTLSAALLSALAQEGTALMSCDSSHLPDGVFAPFGT-HSRHTRAARVQAVWSEPFKKRC-WQKIVRAKVSSQAEVLRR--VGAE
222109284  Acidovorax ebreus TPSY                              ---------------------------------------MVW---------RSIVINRPA-KLKREHFALVVEQE-------------------Q-SARVPFEDIAVIVLNHREITLTHPVLSACGEYGIGLYSTGDNHQPNGVFMPFLQ-HSRATRMQRLQLDLDKPSAKRA-WAHIVQVKIGNQARCMEL--LGTV
336393380  Lactobacillus coryniformis KCTC 3535               ---------------------------------------MAF---------RNLYIQNPA-RLSISRSQLVIQQE-------------------ERYTVPVEDISSIMIEHKQCTITAPTLSFLAEHQVAVFTCDQKHLPCAVVLPLGQ-YYRKLEALEAQLHVRTKTKARL-WQKLMKQKIENQAKVLEL--NHCK
218767587  Neisseria meningitidis Z2491                       ---------------------------------------MTW---------RSLLIQNGG-KLSLQRRQLLIQQN-------------------GESHTVPLEDIAVIIIENREITLTAPLLSALAEGATLLTCDEQFLPCGQWLPYAQ-YHRQLKILKLQLNISEPLKKQL-WQHIVRQKILNQAFVADE--TGND
15602991   Pasteurella multocida Pm70                         MMSSVKIDKFADLSKDNLCVKLYHPDFLYIGNRDFLGVIMSW---------RSILISQGG-KLSLQRKQMLIQQS-------------------NNSFTVPLEDIAVIVIESKETLITAPLLSALAQYGVTLLTCDEQFLPCGQWLPFGQ-YYRQLKTLKLQLETTLPQKKQL-WQKIVQQKIRNQGFVLEQ--VGYF
310780383  Ilyobacter polytropus DSM 2926                     ---------------------------------------MGW---------RNIFITSST-KISTRDSGLLITKE-------------------GGEVNIPLEDISSIILDNNSVTITSRVLEEIASRDIVLLTSDEKHLPNGIFQGYGR-HSRQLAVAKAQINMSVPRKKRL-WQEIVTKKLENQGRVLEL--MRCE
312879014  Aminomonas paucivorans DSM 12260                   ---------------------------------------MGW---------RSVFIANPA-RLSTGGGRLQVEQE-------------------GEAASVPLEDLGALVLESPQVSLTVPLLRELAENRVPLFVCDRRHLPCGVLLPFHA-HCRQEKVLRAQMGLSRPFAKNC-WQRVVRRKILNQAACLRF--LDLP
403744859  Alicyclobacillus hesperidum URH17-3-68             ---------------------------------------MSH---------RHVMVTRSA-KLSCRNNQLVVRQEDESSGE--------------TCEYTIPLEDIRTLMLEDRQSIITSALLARLAEFRIAVFTCDEKHLPNGVLLPYCQ-HSRGPKIMQSQLEMKRPLVKRL-WQAVVRRKIENQAACLTL--CGCS
421874296  Brevibacillus laterosporus GI-9                    -------------------------------------------------------------MSIRLNQLVVKQ-------------------EEEVTIPLEDIASIVVEENQVVLTAALLSKCSEYGIGLYTCDEKHLPNGVLLPFQS-HSRQLRVLETQYNLSKPFKKRV-WQKIITTKIMNQALCLSY--LDKT
220930481  Clostridium cellulolyticum H10                     ---------------------------------------MGW---------RNIIVSNPT-KLKKQNNLWVIEQ-------------------SDGFSIPIDDINTIVLDSADVTITSALLSKLAEEDIALYSCDGKHTPNGVLLPFSC-HSRQYKIVKTQINLSAPFKKRC-WQRVVQQKIENQAFCLNI--LELK
225377803  Roseburia inulinivorans DSM 16841                  ---------------------------------------MGF---------RNIMISSNM-KLRIQDQQLVVTA-------------------GDEVHIPLEDINCILIENQSVTLSAYLLQKVADSGIALYVCDEKHLPNAVLLPMVR-HSRHFKILKYQMETGKPLQKRL-WQQVVIQKIRNQALCLQL--LELD
315659847  Staphylococcus lugdunensis M23590                  ---------------------------------------MSW---------RIVYVSDVN-HMSLNLNSLKIKKG-------------------DLETKIPLADIFALVIEDLTVTITSRLLVELSNYNILVIFCNQKHLPECSLQPISG-HFNQYIQMKEQLKWDEARKNEL-WSAIVRKKIRHNQIECMKY--LDID
160915783  Eubacterium dolichum DSM 3991                      ---------------------------------------MGF---------RVLYIENQY-HLQLYLDNLKVETS-------------------QGDIQFPISDIQLVIDHYRSTLTVPLVNKLTENNVCVIICGIDHLPKSYILPMNG-HFAQSGNIMKQITWSNEIKQIL-HQQIVKAKIFNQIEILKT--NQCK
323463802  Staphylococcus pseudintermedius ED99               ---------------------------------------MSF---------RTVIITKES-KLSLRMNQLIVKSE-------------------NLSRIPLNEILCLVIEHPNVSMTGHLMNALSDQKIVTILCNTKHLPNTLLLPYG-HHRQARLIQNQMNWNKERKGQL-WQLLVKHKILNQKQVIQE--CFKE
389815358  Planococcus antarcticus DSM 14505                  ---------------------------------------MSW---------RTVILAKEA-KLTLRMGHLIVSGE-------------------TVNRVPLEEISFLLIENPNIQLTGHLINALSHHHIVTLICDANMKPSSVVNSVYG-HHRQSRIIAQQATWTEERKGML-WQEITKQKIENQRQLLIH--LKKE
328956316  Coriobacterium glomerans PW2                       ---------------------------------------MAF---------RNVLIESAC-KCTYQGGYLVVRKE-------------------DDAAKIHLSEISSVVLQSTQVFVSAYLLSELAKAKITFVISDEKRNPVGQYLPIYG-AHNTSKRIGEQISWEEPIKKRV-WQRVVRDKITNQARLLEI--RGRS
422884107  Streptococcus sanguinis SK49                       ---------------------------------------MGW---------RTVVVNTHS-KLSYKNNHLIFKDA-------------------SRTELIHLSEVDILLLETTDIVLSTMLIKRLVDENILVIFCDDKRLPTAYLMPYYG-RHDSSLQLSRQITWNEETKAQV-WTTIIAQKILNQAFYLGS--CGFL
116628212  Streptococcus thermophilus LMD-9                   --------------------------------------MAGW---------RTVVVNIHS-KLSYKNNHLIFRNS-------------------YKTEMIHLSEIDILLLETTDIVLTTMLVKRLVDENILVIFCDDKRLPTAFLTPYYA-RHDSSLQIARQIAWKENVKCEV-WTAIIAQKILNQSYYLGE--CSFF
24379808   Streptococcus mutans UA159                         ---------------------------------------MGW---------RTVVVNTHS-KLSYKNNHLIFKDA-------------------YQTEMIHLSEIDILLLETTDIVLSTMLIKRLVDENILVIFCDDKRLPTAMLMPYYA-RHDSSLQLSHQISWTEEVKCDV-WTTIIAQKILNQSCYLGE--CFYF
13622194   Streptococcus pyogenes SF370                       --------------------------------------MAGW---------RTVVVNTHS-KLSYKNNHLIFKDA-------------------YKTELIHLSEIDILLLETTDIVLSTMLVKRLVDENVLVIFCDDKRLPTAMLMPFYG-RHDSSLQLGKQMSWSETVKSQV-WTTIIAQKILNQSCYLGA--CSYF
227824982  Acidaminococcus sp. D21                            ---------------------------------------MSY---------RTIVITQRC-KLDFCMNYVEVQTA-------------------VSKKRIFIDEIKTLIIENTGVAITAYLLSELMNRKVNLIFCDKAHNPQSSLLPLHA-RFDSIRKIKQQMIWSQEIKDEV-WDCIVKAKIRQQALFLDE--LEKK
169823756  Finegoldia magna ATCC 29328                        ---------------------------------------MGW---------RTVLISGRA-KLDLKLNNLVIRK-------------------EEIVKIHIPEIAVLIIDSTMVSFTTALIEKLAEKVKIIFCGKDHNTIGEIVNYYN-KHNSPLMIRKQISWQNNSKQKT-WREITKEKINRQALLLKF--IGNE
320528779  Solobacterium moorei F0204                         ---------------------------------------MGW---------RTVIISNTA-KLDYKMDYLVVRSS-------------------NQVSRVHLSEISLIIVETPSVSLTAYLLCELAANKIGIIFCDEKRYPNGQYIPFYG-SHDTSEKIRKQYNWNLNSKMII-WKEVVIRKIAGQMGVLNRYNHVKS
303229394  Veillonella atypica ACS-134-V-Col7a                ---------------------------------------MSW---------RTVVISSRS-KLDFKMNYMIIRKD-------------------FETTKIYIDEIYMLVIESTAVSLTAVLLNELIKKVAIVFCDEKRNPASEVLPLHG-SHNSSKRCREQADWSKDVQAYI-WTEIVRRKIMNQAELLKY--KELK
304438953  Peptoniphilus duerdenii ATCC BAA-1640              ---------------------------------------MTW---------RVVVIDNRA-KLELKLSHLVVRQDG-------------------NKVQKIYLKDISHIIVNTTEASITFALLNEIVKNKIKLIFCDETRNPTAEVSPLYS-SYNTSEKIRLQIKWDEYTKGEV-WQQIVKQKIKNQRNLLEA--AGKP
374307737  Filifactor alocis ATCC 35896                       ---------------------------------------MSW---------REVIITGHA-KLDYKMDYLVVRKQ-------------------DGTDRVYIPDIGFLMIESTRVSLTTTLISELVKNKVKIIFCDANHNPQSELLPYHG-SHDTSLKVREQVSWSDEIKQLV-WTEIVKEKIRNQMELLEE--YQNF
256845020  Fusobacterium sp. 3 1 36A2                         --------------------------------------MSGW---------RVIVITGRS-KLDLRYNSISIRRD-------------------NGTDFIHIGEVNTLILETTTISITAALMCELIKQKVKVIFCDEKSNPHFELLPFYG-SHDCSAKIKEQIAWTDFLKESL-WTIIVTEKIENQMKLLKK--LNKE
306821690  Eubacterium yurii ATCC 43715                       ---------------------------------------MSW---------RTIEISNSA-KLDYQMGYMVVRQ-------------------ESTTKIHLSEIHTLILATTAISITCALLSELTKQKVKIIFCDEKRNPSSELIPYYG-SHDSSAKVRTQIQWDNEMKLEV-WTQIVKEKIRQQALHLKS--RKKS
42525844   Treponema denticola ATCC 35405                     ---------------------------------------MSW---------RTVVISNRA-KLDLHLNHLVVRG-------------------EKTQKVFIEEISVLIIETTAVSITAALLNELIKQKVKVIFCDEKRNPASELIGYYG-SHDTSEKIRLQIKWDKNIKQLV-WTEVVTEKIRQQKYLLEK--LNLP
291520706  Coprococcus catus GD-7                             ---------------------------------------MSW---------RIIVISKRA-KLDLQLGKMVVRSD-------------------EITK-IVLSEISTIIIESTAVSITASLLSELIKKRKIKVIFCDEKRNPSCELVNYYG-SHDTSNKIRKQIAWKQQTKEAI-WTEIVTDKIRKQKDLLEL--LRRK
258509198  Lactobacillus rhamnosus GG                         ---------------------------------------MGW---------RSFIITQHC-KVTTKQHSLVVQTN-------------------GEVYTMPIDDLNQVIFTTTRALISADAIAALADANAKVIFSGRDGQPVTETKNLYS-DRRRAEQVRLQVNWPLDLIETV-WTKIVAAKISNQAQVAKL--CGHD
336394883  Lactobacillus farciminis KCTC 3681                 ---------------------------------------MGW---------RTIYITQKA-KLSYKANHLLIQTN-------------------MDIKQIPFHQINCIIIATTQSVITGYLISKLVTENIKVIFCAEDHNPCAELNGYYN-NIQRNQNIENQVSWSLPTKQDL-WTRVVKNKLQNQRSVLVR--ADLS
339625080  Fructobacillus fructosus KCTC 3544                 ---------------------------------------MAW---------RTVFVNEHA-KINYKMNDINIQTK-------------------EGGTRIPIDDIRVLILGNTRTVITSYAVNELQKKNVKIINCDEKGMPVGEFLSYCD-NTKQNINIWKQFDWAENRKNLL-WQKIVQTKIFNQHQALGE--KVIA
310286727  Bifidobacterium bifidum S17                        ---------------------------------------MAW---------RNVVVTRHC-KISTKMRLLVIQTD-------------------DDVCQLPMDDIGMLMIETTRAVITTCAIADLLKRDVKIVFCDDKHLPIGETSPYET-ESSRRAYIIQQIRWDEARKDLL-WQHIIREKIAHQVSVLEK-NCDSL
366983954  Oenococcus kitaharae DSM 17330                     ---------------------------------------MAW---------RNILINQHC-KISYKMNMCVVQTE-------------------TDVHQIPVEDIRLLVIATTRAVITTYAVMELLKRQVGILFTDDKAFPIGEINNYLG-GGNRNKNIQQQVDWPIERKQNL-WAEIVKSKILNQSKCLDF--FSEN

informative positions                                         ____________________________________________________________**************___________________*******************************************************_*********************_*****************_____
```

Supplementary Figure S3. Multiple sequence alignment of representative Cas1 sequences (see Supplementary Table S2 and Materials and Methods). Informative positions chosen for the Cas1 tree reconstruction are marked with asterisks at the bottom of the alignment.

16130662 *Escherichia coli* str. K-12 substr. MG1655 ------------------ARRSVEQLRGIE-------------GS-RVRA----------TYALLAKQYGVTWNG-----RRYDP---KDWEKG------DTINQCISAATSCLYGVTEAAILAAGYAPAIGFVHTGK--PLSFVYDIADIIKF---DTVVPKAFEIARRNPG------E----PDREVRLACRDIFRS
11500011 *Archaeoglobus fulgidus* DSM 4304 D------------SD--VIASLQHLSSAKS-------------IV-ELMG----------AEAAARNAYYTK-FDEILKNFEFGKRSRMPPE--------NEVNAMISFGNSLLYSAVLSEIYHTQLNPAISYLHEPSERRFSLALDIAELFKPVIVDRLIFYLVNNGIVTESDFDSRLGGILLSE-EGKKKFVRHFNE
18977490 *Pyrococcus furiosus* DSM 3638 S------------DF---SNHLKELQGAKK-------------VT-EIMN----------VEGRIRQEYYARWDESLPGEFRIGKRTRRPPK--------NEMNALISFLNSRLYATMISEIYNTQLAPTISYLHEPSERRFSLALDLSEIFKPIIADRIANRLVKKGIIKKDHFREDLNGVLLTD-EGMKIVTKAYNQ
46447796 *Desulfovibrio vulgaris* str. Hildenborough H-GDKVDGVALEAEVLHLRACLMRLQQPAG-------------LD-AVRG----------IEGEAAKGYFSVFDN-----LILTREAAFRFEGRSRRPPLDRVNCLLSFIYTLLGHDVRSALEGVGLDSAVGFLHRDRPGRHGLALDVMEEFRAVVADRLALSLINLGKLKKSDFEIQETGAVRMTDDARKALLVAYQK
54296139 *Legionella pneumophila* str. Paris K----NRPENEKIILHAASQKMAGIVANIT-------------ET-NWIKLQDWRFTLLGFEGSAARIYWETVKEVNLVCSDFEG---RRPRVNK-----DITNSMLNYAYSILSSWVWQAITNAGLELYAGILHTSKPGKPSLVLDLMEEFRPWCADRIIFKLHARAQKQNE-L----TPALKK--TLIGEMANLYAK
331001028 *Parasutterella excrementihominis* YIT 11859 LMKRETAYGLLLRNAGTRLEEISNLLNTRA-------------SGPDWKN------TVMGLEGSASLIYWNALKEARLLPETFQE---REGRGST-----EITNAALNYGYAILHSYIQNALGNAGLEIYAGLLHADRAGKPSLVLDCIEEYRAWVVDRNVISLRCKLGSEQNFL----D----T--ALKKQLVELIDK
319941582 *Sutterella wadsworthensis* 3 1 45B LNKSAPEKAEKLREA---ADTIAKIVTKTKAEIENLQTWNEGIWST-ILMG----------YEGAAATLYWQALRTAELVPETFLT---REGRGSV-----EIVNAALNYGYAILQSYIWSALDNAGFELYAGFLHRRRPGKPSLVLDVMEEYRAWVVDRNIIKLRSMLKNSKRGL----T----Q--EIKSAIVNAIDE
118497353 *Francisella novicida* U112 H---------KSINLLNAIEELKRVAQLIKNAKT----------LN-DVLG----------YEGYAANIYFSSLAK-----DKFLS---ASFANREGRGSQEIANSMLNFGYAILSSYILNAITNAGLEPYLGFLHQKRPGKMSLVLDLMEEYRAWVVDRVVIKLREQYKNKQY-I----D----T--KLKSILISEIQA
34557933 *Wolinella succinogenes* DSM 1740 N------TDKQSKKLESAIEKIKETIESLK-------------NI-ELSNNKHWSQIIMGKEGGMAAIYFDAIGAELLEECDFTT---RIGRGAE-----DIMNSALNYGYAILTSFVWQSIINAGLEAYAGVLHTNRPGKPSLVLDVMEEYRAWVVDRNIIKLRSSLAGKKE-L----D----A--ELKKKIITEINA
187736488 *Akkermansia muciniphila* ATCC BAA-835 H-----------------PAIAELKRMA--------------VT-EKTA----------REAECARLFW-SVFADTWANSDFRR---GRHE--------EGFNNLFNYAYAILLSCILQYLFALGLDPCFGIFHQSREHAAPLAYDLMEPFRPAF-DANVARWIHLCLREGKTE----ERAGEITREFRQHITATLQA
407803668 *Alcanivorax* sp. W11-5 S-----------------TARLARLAEAVE-------------PG-DAGH----------LEGQGARLYWRDLFD-----VPFKR---QKRGAE------DPINTRLNFGYALLRSLVARELAFAALRPEIGVGHRSTENPFNLADDFLEPYRFLV-DEIVVSYPPDENSEFDAQ------------SRKRLISDLFEQ
325972002 *Sphaerochaeta globus* str. Buddy G-----------------VKRLDVLSQTVL-------------SG-DSSN----------NEAQGARVYFESIYG-----TSFFR---SDED--------NAINGKLDYGYSILRSAVARAVVDTGLLPSCGVFHSSRINPFCLVDDLMEPFRPMV-DSLVLAMCSKYGVIED-----------LESREKKELMRITAR
187250661 *Elusimicrobium minutum* Pei191 K------------AA----ETIFNMAREVR-------------SG-DATF----------KEGVGAKVYFENLF------NDFHRNTDDK----------DIINSALNYGYAIVRSSIARAVVSAGLNPAIGIFHSKNHNPFCLIDDLIEPLRPLI-DFMVKNKLDVLTQEESLS---------P--SAKKYMASVIES
47458867 *Mycoplasma mobile* 163K N------------NSEFGIEYFDDLINNVN-------------LF-DISN----------REGHAAKVYWNMTYS-----KEFIR---NNSATKLKFDKFEIVNAILNYGYSILRSSAIQSIIKKGLDPRFSIFHKSFSNFFALASDLIEIFRPLV-DEIAYLHKDETFFDV---------------KIKDELINVLNK
363542551 *Mycoplasma ovipneumoniae* SC01 N------------SED--EAKMWDYFHDVQ-------------PY-DTNN----------REGHAAKLYFKLLFG-----KGFIR---DQNGD-------DNINILLNYGYIVLLSYVARIICGKGLDNRLGIYHKSFNNNFPLACDLMEPYRYWV-DQIVYNHINAEFLNFQ-------------DFKEALFKSFSN
71894593 *Mycoplasma synoviae* 53 N------------KED--VEKFNFYYKKII-------------PG-DKNS----------MEGHIAKLTFKNLYG-----STFNR---TDKE--------NEINKFLNYGYTILMTYVSRNLVKKGYDNRIGVFHKSFNNHFALATDLMEPFRFLI-DKLVYELLIIEKNYDF-I------------NFKKKVFLIFEE
384393287 *Mycoplasma canis* PG 14 S------------EEE--IDKLIKYYESVL-------------IN-DRSN----------CEGHAAKLSFKILYG-----ANFSR---DD-E--------NIINTFLDYGYTVLMTYVSRSIIKNGLDNRIGIFHKSFNNHFALSCDLMEPLRALI-DKLVYTHIYIYGKKDF-I------------LFKKDLFCMFEE
284931711 *Mycoplasma gallisepticum* str. F T------------DDN--IEKLTNYYTNVV-------------DG-DLNN----------CEGHAAKLSFKLLYG-----EDFSR---SQKD--------DIINKYLDYGYIVLMTYVSRTLVKNGLDNRIGIFHKSFNNHFALSCDIMEPFRPII-DYLTFSYLIKNQNDDFK-------------SYKKDLFISFEN
117929157 *Acidothermus cellulolyticus* 11B G-----------------VAQLERLAAQVR-------------SG-DASN----------AEGAAARVYWARLFQD----KHFRRVPRAR----------DVVNGLLDYGYAILRGCCLRAVVGAGLAPSLGLWHRRHDNPFTLVDDLIEPFRPAV-DKTVIEIVTAGASG---L----D----R--PTKRLLVAVLDH
317482065 *Bifidobacterium* sp. 12 1 47BFAA G-----------------GGLLRGIAASVR-------------SG-DPSN----------CEGHAAREYWRRMFPQD---EKFRR---IP-GQG------YGRNAQLDYAYMVLRGFAVKAVIAAGLIPSLGVNHHGRGNYFCLADDLLEVYRPAV-DYRVSLLNDEDSLQEK-------------AVKKHLVDAVNQ
315605739 *Actinomyces* sp. oral taxon 180 str. F0310 G-----------------SGELLALADQVR-------------SG-DPGN----------IEAQAARLYWSRTLG-----KGVGRQPAAGQL--------IGANACLDYGYSVLRGHLMRAVLAAGLAPALGVFHRGRGNAFALADDLIEPFRPAI-DEVALQLPPTASPSDR-------------SVKQLLVAAASQ
294086112 *Candidatus Puniceispirillum marinum* IMCC1322 S-----------------VERLNRLHTLIK-------------SG-DPQN----------IEAQAAQVYWPALFG-----RDFRR---DRHK--------GDVNALLNYGYAIIRSAMVSAVLSAGLHPTFGMFHKNKNNPLCLVDDLMEPYRPLV-DQLVKRLVERGHSN---V----T----T--EVKRCLASIVTS
282880053 *Prevotella timonensis* CRIS 5C-B1 G------------------KCLFPYYNNVK-------------SG-DSTN----------REGAAAKLYWTQLFG-----KDFIR---DRTQ--------SGTNALLNYGYTILRAAVTRAIMSAGLFPALGIFHHNRSNAFPLADDMMEPYRPFV-DEIVYHLTQNGQTT---L----T----T--EVKGILIKLLYC
374384762 *Odoribacter laneus* YIT 12061 G------------------NILKPFYCNVK-------------SG-DTDN----------REGAAARVYWTEMFG-----TSFIR---DRYG--------ECPNNLLNYAYIVLRAAVARAITGSGLLPTLGLFHHNRYNAFCLADDLMEPYRPYA-DEVVSGLFQKGEKE---L----T----T--AVKAEILKILAN
423317188 *Bergeyella zoohelcum* ATCC 43767 S------------------SPMYEYLNQVK-------------SG-DTSN----------MEGIAAQHYWKYLIH-----PDFLR---ERYG--------DSPNQFFNFGYSVLRSIVARAIVETGLLPVLGIFHKNKYNAYCLADDLMEPYRPFV-DLLVIRWLEKNPEAEE-L----T----K--EAKAYFLQIATK
385811610 *Ignavibacterium album* JCM 16511 S------------------DNMKKWARDVK-------------SG-DPDN----------LEGRAAAYYWKNLFPEE---LQFQR---DRYG--------KPPNNLLNYGYAILRAVVARSLVASGLLPTLGIHHHNKYNAFCLADDIMEPYRPFV-DDIIVTLVRDGEDFTE-L----T----P--SIKKQLLTIPVI
402847305 *Porphyromonas* sp. oral taxon 279 str. F0450 P-----------------AKNMLAWSRQVK-------------SG-DADN----------LEARAVAFYWRNLFISL---PGFVW---EREE--------AAPNSLLNYGYAILRAIIARALVGCGLLPTLGIHHHNKYNAYCLADDIMEPYRPYV-DLLVVEICEEEGFPTE-L----T----K--EIKARLLALPTL
345885719 *Prevotella* sp. C561 S-----------------FVPLHVLARKVR-------------SD-DADN----------HEAQAAAYYWKNLFS-----DGFTR---DKDG--------IPPNNLLNYGYAILRAVIARALVGSGLLPVYGIHHHNRYNAYCLADDIMEPYRPFV-DDLVLSTMKKMEISDD-L----T----V--DLKREMLSIPVL
404487227 *Barnesiella intestinihominis* YIT 11860 A------------------KNMRAWVGEVR-------------SG-DIDN----------MEARAAAYYWSSIFPHL---DSFRR---DRDG--------DPPNNLLNYGYAILRAVVARSLVISGLLPTLGIHHHNRYNAYCLADDVMEPYRPYV-DGLVCEIMESGKDYSM-L----T----T--ELKAKLLSLPVV
301311870 *Bacteroides* sp. 20 3 E-----------------IKCMRIWANDVR-------------SG-DPDN----------LEGRAAAYYWKYLFGHI---EGFTR---DREG--------IPPNNLLNYGYAILRAIVARGLVTSGMLPTLGIHHHNRYNAYCLADDIMEPYRPYV-DELVFGLIRTKGISPEIL----T----K--EWKASLLSIPTL
60683388 *Bacteroides fragilis* NCTC 9343 E-----------------VKCMRIWATNVR-------------SG-DPDN----------LEARAAAYYWKSLFSDV---DSFTR---EREG--------IPPNNLLNYGYAILRAVVARGLVISGLLPTLGIHHHNRYNAYCLADDIMEPYRPYV-DELVFSLIQEYGKNAE-L----T----K--EMKTRLLTVPTL
402849996 *Rhodovulum* sp. PH10 S------------------GAIRALLPRVR-------------SG-DPDN----------VEALASQRYFPAFFG-----TGFRR---DRNA--------AGANALLNYGYTVLRAATARAIVAAGLHPSLALHHRSRGDALRLADDVMEPFRPTV-DLAVKMLLDEGRED---L----D----Q--GAKRRLAGVLHA
154250554 *Parvibaculum lavamentivorans* DS-1 H------------------GGLLPMARRVR-------------SG-DPDN----------LEAQAAQRYWPRLFG-----KDFRR---DRDL--------EGVNALLNYGYAVVRAATARATVAAGLIPSLGVFHRNRANPFCLADDLLEPYRPYV-DWRVRLLANQMGEEAPSL---------DDRDTRAALLSIFNE
159042957 *Dinoroseobacter shibae* DFL 12 H------------------APVGMLARKVT-------------SG-DATN----------VEAQAARLYWPRMMG-----TEFRR---DRTA--------PDLNALLNYGYTVLRAATARAVVAAGLHPTIGLHHSNRGNAFALADDLMEPFRPLV-DCCVRGLAARNGPQVD----------P--AAKQSLARLIAL
288957740 *Azospirillum* sp. B510 A------------------GGLVEMARRVR-------------SG-DPDN----------LEAQAARRYWPALFG-----REFRR---DQDG--------DGANALLNYGYAILRSATARAVTAAGLHPSIGLHHANRGNPLCLVDDLMEPFRPFV-DLAVLRLAEAGHRE---V----T----R--ETKRYLALVPSL
330822846 *Alicycliphilus denitrificans* K601 T------------------APLLALVSKVK-------------SG-DPEN----------IEGQGARRYWGLLFG-----NTFRR---DQNG--------DGVNALLNYGYTILRSATARAVVAAGLHPSIGLHHSNDSNAMRLVDDLMEPFRPVV-DLKVWQLLAQGEAH---V----T----P--ETKRALVRVLYD
344171926 *Ralstonia syzygii* R24 S------------------APLSALVSKVR-------------SG-DPEN----------YEAQGARRYWVLLFG-----DTFRR---DRQQ--------EGINTLLNYGYTVLRAATARAVIAAGLHPSVGLHHSNTQNAMRLVDDLMEPFRPLI-DLQVHACWRAGDIALDR-------------ETKKRLADVMYL
319957207 *Nitratifractor salsuginis* DSM 16511 N------------------PKLERYAEQIR-------------PG-DPYR----------TEAKSARLYWNKLF------DGLKR---EA-DSL------DVRNQALNYAYAILRSLITRDLSAAGFLPALGLWHDNRYNAFNLSDDLMEPFRPIV-DAAVYLLLPLCDEEYLTP------------EFKKAIISIFDS
315149831 *Enterococcus faecalis* TX0012 S------------ER---VEVLEKLSEGIL-------------PG-DTTN----------REGHVAKVYFHSLYG-----VGFTR---EE-E--------CLPNACMNYGYAVIRAQMARCVVALGLLPMLGIFHKNEYNSFNLVDDLMEPFRPLM-DWYIHQTILKKNEKYLTY------------HSRLTLVEFLHQ
116627543 *Streptococcus thermophilus* LMD-9 L------------DN---IRLLSDYKEQIE-------------PG-DRTN----------REGHAAKVYFNELFG-----KQFVRVT-QKEA--------DVINAGLNYGYAIMRAQMARIVAGYGLNGLLGIFHKNEYNQFNLVDDLMEPFRQIV-DVWVYDNLRDQEFLKY-------------EYRLGLTDLLNA
238924076 *Eubacterium rectale* ATCC 33656 S------------DG---PEKIIEFSKNIL-------------IG-DKSN----------REAHAAKVYFNLLMG-----TSFSR---GNED--------ILLNSGLDYGYAVIRGYIARACVGYGLNTQIGIHHKSEYNRFNLVDDLMEPLRPIV-DIVAYNSMKNDKYFTP-------------EHRRKLVSILNM
218563120 *Campylobacter jejuni NCTC 11168* E----------------QSNELINLAKKVS-------------LN-DSKN----------IEAVAAALYFKTLFG-----TSFSR---DE-L--------CFENSALNYGYAIIRACIIRAVCISGLLPWLGIKHDNIYNSFALCDDLIEVFRASV-DDCVLKLKGESEFLSK-------------DDKRALIGNLQS
291276264 *Helicobacter mustelae* 12198 S------------TA----TKLQALQKAVK-------------LA-DGTN----------NEAKASALYFPSLFG-----RGFSR---KD-F--------SPINAALNYGYAIIRGCIARNLVGSGLIPSLGIFHSNQFNPFNLADDLIEPYRPFV-DSRVLALRIENELTL--------------SHRVELVNLLNT
325677757 *Ruminococcus albus* 8 T------------DE---IKPIEEMISEVL-------------PN-DKTN----------REAAAAKRYFIALFG-----NSFRR---IK-D--------DVTNAALNYGYAIIRSAVAKTLVAYGYNCVLGIHHINEQNAYNLADDMMEPLRPLV-DLWVDNSRDDLFEELT----------K--TNRRDLVDLVNV
296446028 *Methylosinus trichosporium* OB3b A------------------EALRAMAKLVG-------------SG-DPDN----------VEARAARDYWRALY------KDFTR---DDAC--------DLRNKMMNYGYAVVRACVARALVAYGLLPALGLHHVSVTNAFNLADDILEPFRPFV-DAIAVARAEKRKKTDDLT---------I--DDRRAMAGALHV
34557789 *Wolinella succinogenes* DSM 1740 D----------------AARRLENLVGKVT-------------SG-DTTG----------VEAQAAQVYWRSLF------VNFKR---WDGGL-------DARNGGLNYGYAVLRGALGRAIAGAGLIPAFGLHHAGELNSFNLADDLIEPFRPFV-DLLVWGLFLERTGDDP-L----T----R--EERMRIAQILGE
222109284 *Acidovorax ebreus* TPSY G-----------------TDRLASYARRVR-------------SG-DGGN----------LEAQASAYYFPQVFG-----RSFHR---SQ-T--------GWSNAALDYGYAVMRGACARALVAHGMLPSLGLFHRSEQNAFNLADDLIEPYRPVV-DLHVAQHRPADEDAELQP------------SDKVALVGLLNV
336393380 *Lactobacillus coryniformis* KCTC 3535 N-----------------VAELNFLATQVT-------------EG-DKTF----------KESRAARLYFMGLFG-----DQFNR---RN-E--------NPMNGALNYGYAIIRGIIARDICAYGFEPALGVFHKNELNPFNLADDLIEPFRAYV-DLWAYEHQTDLKAG---L----T----P--TNKQALFQLLFT
218767587 *Neisseria meningitidis* Z2491 L----------------AAKRLRTLASEVR-------------SG-DTGN----------REAQAAALYFQALFG-----EKFTR---ND-N--------NAVNAALNYTYAVLRAAVARALTLYGWLPALGLFHRSELNPFNLADDFIEPLRPLA-DLTVIHLYEQGRLKTE-L----T----L--GIKQHLIKILYY
15602991 *Pasteurella multocida* Pm70 N----------------ETKRLNRMANQVK-------------SG-DKEN----------VEAQAALIYFQTLFG-----KTFKR---SE-E--------NNINAHLNYAYTVLRSAIARALVLYGWLPQLGIFHRSEVNPFNLADDFVEPFRPLV-DLMVWQLWHTNQLTSG-L----S----P--NTKQKLISLLHH
310780383 *Ilyobacter polytropus* DSM 2926 G-----------------SKYLYDLSKRVK-------------SG-DDGN----------LEALGAKYYFQKLFG-----DDFTR---RD-H--------DIKNFALNYGYAVIRGRIARSLTSYGYICSLGIQHKNEYNAFNLADDIIEPYRSVV-DLWVANNIDKLSDELTT-------------DNKVSLVEILNA
312879014 *Aminomonas paucivorans* DSM 12260 G-----------------AEELHALASQVR-------------SG-DPDQ----------REGVAARLYFSRLM------PGRTR---DD-P--------TSRNAALDYGYSVLRGALARSLAAHGFLPALGIQHHNELNPFNLADDLLELFRPQV-DLWVFRNVSDDDPFSP-------------EHRASLVGLLHR
403744859 *Alicyclobacillus hesperidum* URH17-3-68 G-----------------DEALLALAGRVA-------------SG-DKGS----------IESQAARMYFKHLFG-----KAFKR---SS-D--------DFINSAMNYGYALIRGQVARSLCAFGFQPSIGLQHHSELNNFNLADDLMEPYRPIV-DYVVASELDVDSPILSKL-------------HKRRLMQILTQ
421874296 *Brevibacillus laterosporus* GI-9 G-----------------ESELLAIRNTVD-------------SG-DSTN----------REAYAAKVYFQHLFG-----KRFIR---RE-D--------HIINHSLNYGYSILRGNVARVLVQYGFFPCLGIDHHNENNAFNLADDFMEVFRPLV-DLFVVKHFHEEMEFTP-------------KWRAGLYNVVNH
220930481 *Clostridium cellulolyticum* H10 G-----------------RDELINLSKSVL-------------SG-DSTN----------VEAHAAKYYFSVLF------TNFKR---GM-Q--------DNTNYALNYGYSILRGAVARTIASYGFIPSIGIHHRSELNNFNLADDFIEPFRPIV-DMWVKQNINEDTLLTP-------------KHKLNLISLLGY
225377803 *Roseburia inulinivorans* DSM 16841 G-----------------HEELLRMCKEVQ-------------SG-DRTH----------VEAKAAAYYFRCMYG-----LGFSR---GN-D--------HIINSALNYGYAIIRGMIARSIICYGLEPSIGIFHHSELNNYNLADDMIEPFRPLV-DLYVSSHFDISEIDST-L----T----P--EMKKGLFGIINF
315659847 *Staphylococcus lugdunensis* M23590 Q------------LR---INKMTDLYHSVA-------------LF-DRNN----------IEGQAAKYYFNSFF------KDFIR---DNDE--------LIENAVLNYGYTIFNAAIARTIVAKGLIPAIGIHHIGGRNHFNLASDLIEPFRPIV-DLFLLKYPPEDFLTK--------------DYRVKLINLLHA
160915783 *Eubacterium dolichum* DSM 3991 Y------------EV---IKKLYEFYDTVD-------------LG-DATN----------REGLAAKMYFREMFG-----NDFIR---FE-D--------DVINAGLNYGYSIFRSLISSIIVGKGYLPNLGIFHKGKTNMFNLSDDIIEVFRPIV-DKYVYDNLKEELLFKS-------------KHREELIQLTVK
323463802 *Staphylococcus pseudintermedius* ED99 R----------------DSELFDAFANNVE-------------LD-DSTN----------REGHAAKVYFNIVLD-----PGYAR---GD-D--------TAQNAAMDYGYQVLLAIVARTIVSKGYLTELGIKHHNEFNIYNLASDFMEIFRPLI-DYLVLKNVEQQFET---------------EQKRAILDILNR
389815358 *Planococcus antarcticus* DSM 14505 G-----------------IDELSMFRDQVE-------------VF-DTTN----------REGHAAKVYFNRLYG-----QDFTR---SI-D--------EPRNWALNYGYVVIHSLIARQIVSRGYLTELGIFHANEFNQMNLASDFVEVFRPLV-DYIVHEHITDTFGK--------------DEKRKVLKMLEY
328956316 *Coriobacterium glomerans* PW2 E----------------EAGRIGAIVSDVR-------------SG-DTTN----------REAYAARCYFQSLFG-----DSFSR---DD-D--------LAINAALNYGYAIILSAVNREIVSRGYLTQLGVCHRNEYNQFNLSCDLMEPFRPIV-DRIVFDNVQDDFTR---------------NDRRLLVDLMNG
422884107 *Streptococcus sanguinis* SK49 E----------------KSQSVIDLYHGLD-------------LF-DPSN----------REGHAARIYFNALFG-----NDFSR---EQ-D--------NDVNAALDYGYTLILSMFAREIVLSGCMTQFGLKHANQFNQFNLASDIMEPFRPII-DKIVYENQNSSFV----------------KIKRELFTIFSD
116628212 *Streptococcus thermophilus* LMD-9 E----------------KSQSIMELYHGLE-------------RF-DPSN----------REGHSARIYFNTLFG-----NDFTR---ES-D--------NDINAALDYGYTLLLSMFAREVVVCGCMTQIGLKHANQFNQFNLASDIMEPFRPII-DRIVYQNRHNNFV----------------KIKKELFSIFSE
24379808 *Streptococcus mutans* UA159 E----------------KSQSIMDLYHDLE-------------PF-DPSN----------REGHSARIYFNTLFG-----NVFSR---EQ-D--------NDINAGLDYGYTLLLSMFAREVVVSGCMTQFGLKHANQFNQFNFASDIMEPFRPIV-DRIVYENRNNSFI----------------KIKRELFSMFSD
13622194 *Streptococcus pyogenes* SF370 E----------------KSQSIMDLYHGLE-------------NF-DPSN----------REGHAARIYFNTLFG-----NDFSR---DL-E--------HPINAGLDYGYTLLLSMFAREVVVSGCMTQFGLKHANQFNQFNFASDIMEPFRPLV-DKIVYENRNQPFP----------------KIKRELFTLFSD
227824982 *Acidaminococcus* sp. D21 E----------------QSKMLMSYLDDVV-------------SA-DAHN----------REGHAAKVYFNALFG-----NSFTR---DL-D--------SPINAGLNYGYSLLLSLFNREIVSAGYLTQLGIFHENTYNPYNFSCDLMEPFRILV-DRYVYNMNPTTFAK---------------DEKREIINLFQE
169823756 *Finegoldia magna* ATCC 29328 N-----------------ANLLLEYKSQVQ-------------DG-DVSN----------REGHAAKVYFNSLFG-----KDFSR---SQ-D--------NPTNAMLNFGYSILLSIFTREITSSGYLTQLGIFHENQFNYYNLSSDLMEPLRPMI-DNIVYFYEPEKFDK---------------EEKIATLKIMDE
320528779 *Solobacterium moorei* F0204 G------------------EKLKQYSYDVE-------------IG-DTTN----------REGHAAKVYFNSLFG-----KDFSR---DDKD-------NIINAQLNYGYSILLSAVCREIVKNGYITQIGIHHDNIFNSYNLACDLMEPLRPFI-DLLILKLEHIVLSA---------------DIKQSILTFLNR
303229394 *Veillonella atypica* ACS-134-V-Col7a E-----------------ADLLYQYLDELT-------------LN-DETQ----------REGHAAKVYFNALFG-----KSFSR---EQ-D--------NPVNAALNYGYAILLSTVNREILSLGYITQLGLNHCNQFNPYNLGSDLMEPLRGFI-DEIVINLMPTEFNR---------------DIKLSLIELLSK
304438953 *Peptoniphilus duerdenii* ATCC BAA-1640 E-----------------YALLEKYIEEVE-------------LK-DATN----------REGHAAKVYFNALFG-----KSFSR---SD-D--------SNINAALNYGYAILLASINRQVVSNGYLTQIGIFHDNMFNQFNFSSDLIEPWRVLV-DNTVYIDDIKIFGK---------------EQKIELINLINE
374307737 *Filifactor alocis* ATCC 35896 D----------------RAELLKTYIEEME-------------PG-DKNN----------REGHAAKVYFNTVFG-----IDFTR---ND-E--------NTINAALNYGYNILLSCFNREIVSNGYLTQIGLWHSSRFNSFNLASDLMEPFRIIV-DRKVLTLNLENFDS---------------HNKYELVSILNG
256845020 *Fusobacterium* sp. 3 1 36A2 E-----------------YKILQEYASQIE-------------HN-DNTN----------REGHSAKIYFSALFG-----NNFSR---NK-E--------NSLNAFLNYGYQLLLSTFNKEIVANGYLTQIGIFHKNMFNYYNLSSDLMEPFRVIV-DELAYKENPQKFEK---------------DEKRKLQNILNL
306821690 *Eubacterium yurii* ATCC 43715 Q-----------------YEMLEDYVRQVQ-------------FA-DSTN----------REGHAAKVYFNALFG-----LDFSR---RA-D--------IVVNSALNYGYAIIASMFSREITSCGYMTQLGLFHDNMFNHFNLASDLMEPFRVLV-DRKVCSMEMEEFTS---------------EEKLKLVNIMQD
42525844 *Treponema denticola* ATCC 35405 Q-----------------ASLLAEYITDID-------------IN-DKTN----------KEAHAAKAYFAALFG-----AGFSR---SL-D--------IPINAALNYGYSILLSAFNREIIANGYITQLGIFHDNMFNPFNLGSDLMEPFRPLV-DAEVFKLNPQKFEH---------------EEKLKIVSVINK
291520706 *Coprococcus catus* GD-7 E-----------------SALLDTYLKKIT-------------WN-DGTN----------REGHAAKVYFNSLFG-----LDFTR---TE-D--------SLINAALNYGYSVLMSAFAREIVASGYLTQLGIFHDNMFNQFNLASDLMEPFRILI-DREVLMMTLEVFQH---------------SEKMQLVNVLNH
258509198 *Lactobacillus rhamnosus* GG A------------------QGLLDDLDALE-------------IN-DVSN----------REASAARRYFKLMFG-----DDFSR---SDIC---------ATNAALNYGYSVLLSTTNRAIVSTGHLTEVGLHHHSASNPYNLGSDLMEPFRPAV-DYWVAQHRIKELTP---------------DVKYGLIDLLNL
336394883 *Lactobacillus farciminis* KCTC 3681 P------------------SFLDNEYRFIQ-------------EN-DSSN----------REAVIARKYFLALFN-----ENFSR---GK-D--------TKINAMLNYGYTILLSATNQEIIAQGYLTQLGIHHHSLKNCFNLSSDLMEPFRPFV-DDKVFQKSDYNFDE---------------YMKLELVDILNQ
339625080 *Fructobacillus fructosus* KCTC 3544 N-----------------YGKLKEIGLEVK-------------EA-DEDN----------REGQAARLYFPRIFD-----KDFVR---SNDS--------LKANAHLNYGYSILLSAVSREIVAAGFLTQIGVHHQSQTNPFNLASDLMEPFRPYV-DRIVKWQRDSPLTL---------------EHKLNLVSVLNE
310286727 *Bifidobacterium bifidum* S17 D-----------------LEKMKSLVVDVK-------------PG-DSDN----------REAVAAHMYFPRLFT-----YEFVR---SDEE--------NGINGILDYGYAIILSEVARKITEIGYLTEFGIHHDNEKNPFNLACDLMEPFRPFM-DERVFQLGAEHLDS---------------KVKVELMRFMRS
366983954 *Oenococcus kitaharae* DSM 17330 D------------------RSLEQLIIQVN-------------QN-DNSN----------REAVAARMYFTRLFG-----PSFVR---HNDD--------EPINALLNYGYSILLSTVAREISSNGFLTELGIHHNSQENVFNLACDLMEPFRPII-DRAVAQMTDFDLSL---------------QHKIELIELFDD

informative positions __________________***********_____________**_****__________*************___________________________**_************________________************

16130662	*Escherichia coli* str. K-12 substr. MG1655	S-------KTLAKLIPLIEDVLAAGEIQPPAPPEDAQ----PVAIPLPVSLGDAGHRSS----------------
11500011	*Archaeoglobus fulgidus* DSM 4304	RLEKTVKHRKLNRKVSYQRLIRLECYKLVKHFTAVEK----YSPFVMWW--------------------------
18977490	*Pyrococcus furiosus* DSM 3638	ELEKTVKHPKLGKGVTRRRLIRLEAYKIIKHLVGVEE----YSPLVAWF--------------------------
46447796	*Desulfovibrio vulgaris* str. Hildenborough	RKQDEIVHPFLNERIPLGLVFHVQAMLMARWLRGDLD----GYP-PFVWK-------------------------
54296139	*Legionella pneumophila* str. Paris	PL------PYHKRRISLESIIQRQVYHLSAVFSGEGK----YKGYLFKW--------------------------
331001028	*Parasutterella excrementihominis* YIT 11859	TMST--KINYSQKQCKLENALQRQVYKLAGAIVEGKR----YRGIRFKW--------------------------
319941582	*Sutterella wadsworthensis* 3 1 45B	TMAN--AVSWQGKSVRLENALQRQAYRLAGTVVDGKR----YKSIRFRW--------------------------
118497353	*Francisella novicida* U112	TIAKKY--IYNGKKLKLEHIIQRQVYRLSGEFAGEHN----YKPYIFKW--------------------------
34557933	*Wolinella succinogenes* DSM 1740	TMNK--KHLYNGKNLSLQGVMQRQVYRLVGAFMQQNR----YKSYIFKW--------------------------
187736488	*Akkermansia muciniphila* ATCC BAA-835	SV------MYRDKQLPLKAAVEAVCRSFRKAVLAGQS----EPYEPWLMTTIKWAG-------------------
407803668	*Alcanivorax* sp. W11-5	TV------CLNGQDFRLPAAIRKTVASYLRILEAGGG----GRRRKLELPECP----------------------
325972002	*Sphaerochaeta globus* str. Buddy	EV------VFEEQDVEFSYALRLYVLSYLSFLSRNRD----AIQFPKYRYAWAV---------------------
187250661	*Elusimicrobium minutum* Pei191	NL------YFEDGAFNLTAGIQKYIQSYIAFLEEREN----RIIFPAILK-------------------------
47458867	*Mycoplasma mobile* 163K	KV------IYKGKYFYVNDTFDQIADNLVNKRSWEWV----ELWD------------------------------
363542551	*Mycoplasma ovipneumoniae* SC01	HI------EYKGKHIKFSKYMEFEIMDILNLKENYKE----ITTESDQS--------------------------
71894593	*Mycoplasma synoviae* 53	KI------LLNKSPISVNEYICKLIENFINKDFNFES----LEIDWNKK--------------------------
384393287	*Mycoplasma canis* PG 14	KI------AIKNTKLSVNEYIDKIVKSVINNNLICED----FIIEWS----------------------------
284931711	*Mycoplasma gallisepticum* str. F	FI------FPNGKN--LNQVIDMLIKAIINNKINERD----FNIDWS----------------------------
117929157	*Acidothermus cellulolyticus* 11B	QF------DASGATVG--TAVERFAQQVGRYVEGEIR----SLRPPAMELSHA----------------------
317482065	*Bifidobacterium* sp. 12 1 47BFAA	Q--------FNGSGLTIPSSLNDFAQQFGLYCEGKID----RLQVPEYVGES-----------------------
315605739	*Actinomyces* sp. oral taxon 180 str. F0310	--------RFDGDGHGIPAVAEALAQSFGRYVEGDID----RLCVLAWQGPSSVAVED-----------------
294086112	*Candidatus Puniceispirillum marinum* IMCC1322	DQ------AVVGATSPLFQHMSQLSYSLWENLDGQKT----TMPMPQLIPELEIEAMVSEC--------------
282880053	*Prevotella timonensis* CRIS 5C-B1	DT------YFINVTRPLSVGLTMTMASLVKYYAKEVK----KISLPLLK--------------------------
374384762	*Odoribacter laneus* YIT 12061	DT------YFENTTRPLMVGLSQTTASLARCFSGEQK----KMVYPEFK--------------------------
423317188	*Bergeyella zoohelcum* ATCC 43767	DV------KIDEQVRPLLVAVKSTVSSLYKCYTGEKR----QISYPKML--------------------------
385811610	*Ignavibacterium album* JCM 16511	DV------FIEEQKRPLMIAVQRTTSSLAKCFSGNQR----KIIYPVFRL-------------------------
402847305	*Porphyromonas* sp. oral taxon 279 str. F0450	DV------EIEGQRRPLMLAATQTATSLLRCFTGEAR----RLAYPLLSCEPSA---------------------
345885719	*Prevotella* sp. C561	DV------VIGGKRSPLMIAAGLTTASLAKCYNGEAR----EISYPSFL--------------------------
404487227	*Barnesiella intestinihominis* YIT 11860	EV------GIQGRRSPLMVAVAQTTASLYKCFNGELR----KIIYPEL---------------------------
301311870	*Bacteroides* sp. 20 3	EV------KIGGKRSPLMIAVAQTTASLYKCFSGEQR----RIVYPER---------------------------
60683388	*Bacteroides fragilis* NCTC 9343	EV------IIGGKRSPLMVAVGQTTASLYKCFNGELR----RISYPER---------------------------
402849996	*Rhodovulum* sp. PH10	DF------ATSDGRTPLSNVLARLAVSLAQVFTRERR----GLAFPETLVPLPTAEDDGSANETAEHAVGVSDLV
154250554	*Parvibaculum lavamentivorans* DS-1	TV------LVGGRRMPLLLALHASAASLCRALTGGEA----ALALPEGMPLAPDLLDNDGEG-------------
159042957	*Dinoroseobacter shibae* DFL 12	DL------PLGDSLTPVSVALGKLAISLGQSFESGTL----DLALPAPPDALTLAGLGA----------------
288957740	*Azospirillum* sp. B510	DM------LTAQGTTPIGTVLIRLAGSLAQVVEEGKG----DLDLPALDRPWAPMPLEWPAAPPAGGETGC----
330822846	*Alicycliphilus denitrificans* K601	DM------QTSVGVTPVMVCMQKLAISLAQVYLGERT----KLDLPLQGLPLALAGSSESD--------------
344171926	*Ralstonia syzygii* R24	DM------PSPQGITPVMGAVQRLCTSLAQLYLGERK----ELELPLCPTPIDLAAMFVPLA-------------
319957207	*Nitratifractor salsuginis* DSM 16511	EYL-----IYDAGRSTLRTVATRYIQGFKAVVRSRKI----ENLHFPTIDFGALRECF-----------------
315149831	*Enterococcus faecalis* TX0012	KI------KVKNKKIYMNQAMSEYVAAFVKAMETGDF----ENVQKIHLVNMLECEGK-----------------
116627543	*Streptococcus thermophilus* LMD-9	KI------KYGKETCSVTVAMDKYVKGFIKYISEKDS----SKFHCPVVSSLEWRK-------------------
238924076	*Eubacterium rectale* ATCC 33656	KI------MYREKKMYMCNMIENYIEQYASLIMKRTD----NIVFPDVYNLIGEEYG------------------
218563120	*Campylobacter jejuni NCTC 11168*	KI------NFDGQNYPLNRAINHYVANFKNALLYEDE----LKIVKFDD--------------------------
291276264	*Helicobacter mustelae* 12198	KV------LLQGKFYPFHRSITRMVWSISNFICDRGK----MIELPILDKESNGREVYESFSDV-----------
325677757	*Ruminococcus albus* 8	PI------KMGKKKTRVRYAIDKYISSLTSAIEKNDP----GLLQLPTLIQIDEMFEDDEDG-------------
296446028	*Methylosinus trichosporium* OB3b	NA------RVGKEIVSLLVASEKAAEGLVQAMESGSA----AILKLPFFVTPKGSSP------------------
34557789	*Wolinella succinogenes* DSM 1740	SC------IMTERYETLLSATQIIARSLVSAGEQKES----RRLI-LPWMGEDDVQGEVYEADGDV---------
222109284	*Acidovorax ebreus* TPSY	DV------AMPRGQMSVLASIEQAAESLARLYDGGSE----QVLELPRLIGLRQHVFEI----------------
336393380	*Lactobacillus coryniformis* KCTC 3535	EV------RIDGEFHSLSNAVHKLVSSYTTCCRNNSA----AGLKLPELIPLVAHEYE-----------------
218767587	*Neisseria meningitidis* Z2491	QT------SIERQHFSTLAAIDKMISSFQAGVTDKNA----KQLKLPEILPLKEYQYE-----------------
15602991	*Pasteurella multocida* Pm70	QM------SFQEETFSVLAAIDRTVGSFQAALTTPNA----NLLKLPEILPLKEHQYE-----------------
310780383	*Ilyobacter polytropus* DSM 2926	DV------KLTGKKYSVNISIDEVIKRYTKCIRGKGG----NLILPSIIPIQEHRYV------------------
312879014	*Aminomonas paucivorans* DSM 12260	RM------EIQGERHTILRGTELCAEGFAAACREKNP----DLLALPALISPDG---------------------
403744859	*Alicyclobacillus hesperidum* URH17-3-68	MV------WLENERYDLTASIEAMVSSFSNAISTEDP----SALQLPRLTEVQMGVYE-----------------
421874296	*Brevibacillus laterosporus* GI-9	QV------ELEGERYSCNRAIEEMIKSYVTACRTSMY----ERLRTPTLLPLKIHSYE-----------------
220930481	*Clostridium cellulolyticum* H10	EC------VFEGKIISIRSAIEKVISSFSSSCAKNDY----SLLKLPEIIPLEVHANE-----------------
225377803	*Roseburia inulinivorans* DSM 16841	DM------SVKGDMRIVSNCIDMLIASYSSALQGNRT----ELDLPELIQLQVHSYE------------------
315659847	*Staphylococcus lugdunensis* M23590	RI------EIDGKMQTIIRAIEIMIQSIIEYFKNGHL----DIVKFPNLRKYQFYEL------------------
160915783	*Eubacterium dolichum* DSM 3991	KI------EIDGKLQTVPNAVNEYVESILKVIETGKI----TDFKFPSPHVVDYDL-------------------
323463802	*Staphylococcus pseudintermedius* ED99	KL------KIDNKKYYFLNAVEIYVDSLFKYLSNGNR----EVIKIPTWTY------------------------
389815358	*Planococcus antarcticus* DSM 14505	KV------QIRNSKQFLQAAVQVYLDSCLQYLNDGHK----EKLLFPILDFPKAR--------------------
328956316	*Coriobacterium glomerans* PW2	CI------AYRDGTYKTSSVISLYVQDCLNALNKRLA---IDEIDPFDIV-------------------------
422884107	*Streptococcus sanguinis* SK49	TF------HYNGKDMYLSNIVSDYTKKVIQALNQPEK----GVPEFRI---------------------------
116628212	*Streptococcus thermophilus* LMD-9	TY------LYNGKEMYLSNIVSDYTKKVIKALNQLGE----EIPEFRI---------------------------
24379808	*Streptococcus mutans* UA159	TY------LYNNKEMYLTNIVSDYTKKVIKALNNDGK----GVPEFRI---------------------------
13622194	*Streptococcus pyogenes* SF370	TF------SYNGKEMYLTNIISDYTKKVVKALNNEGK----GVPEFRI---------------------------
227824982	*Acidaminococcus* sp. D21	IL------YIDDSRQFLANAINIYANSIFQALEKCDT----SKIKIYEI--------------------------
169823756	*Finegoldia magna* ATCC 29328	EV------FIGKTRYTLLNAVNEYCKSVFLALEKGEE----QLIKFIDYEL------------------------
320528779	*Solobacterium moorei* F0204	KV------SIMGREQYMLNAIEIYVKSILDAISSENP----SLIKFVEYEFTCYESDSVL---------------
303229394	*Veillonella atypica* ACS-134-V-Col7a	DV------KINDTIQTFSAAVRIYCKSVFDALRVQAA----NEIRFIEYEL------------------------
304438953	*Peptoniphilus duerdenii* ATCC BAA-1640	FV------TIDNKYCRINQALEIYIRSVFDALNNRDI----SLLRFPSYEL------------------------
374307737	*Filifactor alocis* ATCC 35896	EV------RIDSKVSYVNNAIKLYCKSIFEALHYEDT----SYIKFYTNEL------------------------
256845020	*Fusobacterium* sp. 3 1 36A2	KF------RINNVNHYLSDIIKIYTKSIFDALSANDL----SLVRFFEDEL------------------------
306821690	*Eubacterium yurii* ATCC 43715	SV------IINGRTEVLTNAVKIYCKSIFDALEQKDI----SLIRFYENEF------------------------
42525844	*Treponema denticola* ATCC 35405	KV------LINNKEHYLNKAIEIFVHSIFDALNEKDI----SQINFYRNEL------------------------
291520706	*Coprococcus catus* GD-7	EV------RIDGKVQYVNNAIKIYCKSVFDALSEDDS----ALIRFYKIEL------------------------
258509198	*Lactobacillus rhamnosus* GG	EI------TFNGKKALLQNAVADHVENCLAFLDGKAKVCRIEVKFPDEVSSHAINDHV-----------------
336394883	*Lactobacillus farciminis* KCTC 3681	EI------SYGSKTFILKNALSQYIRDCISYLDSNQE----VEIKKVGFKDEE----------------------
339625080	*Fructobacillus fructosus* KCTC 3544	EI------TMNSKRYELAQAVQKHVQECLKYLCSDDS----ELPEMDFEL-------------------------
310286727	*Bifidobacterium bifidum* S17	D--------VPELGTTITQLISVFVRDALRYLSGEEC----LPELGFVRCGTELCE-------------------
366983954	*Oenococcus kitaharae* DSM 17330	EV------GYLQHQATVNSAITEYVRNALDYLCGKRP----EVAVSVAI--------------------------

informative positions *_______*****************************______________________________________

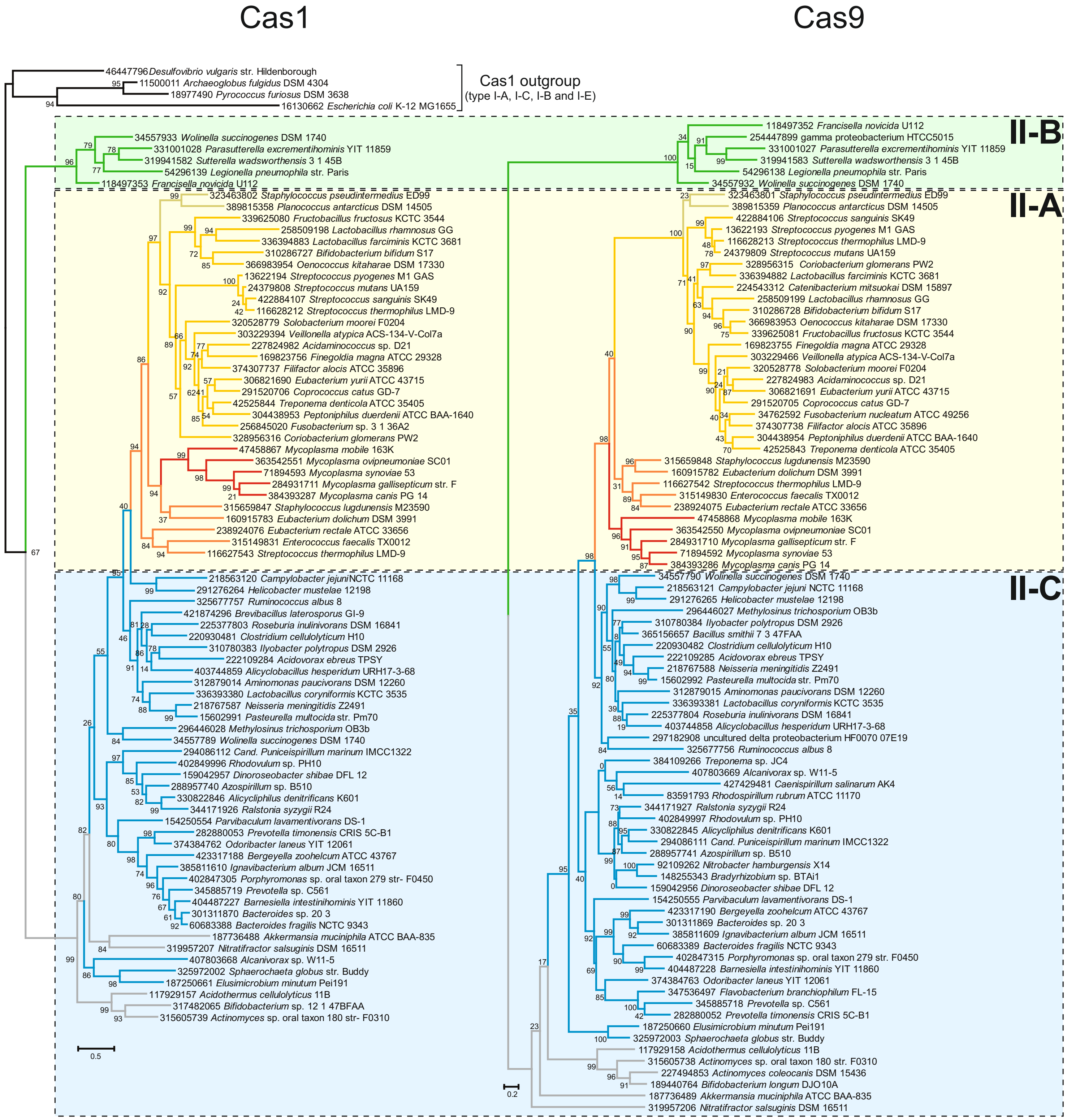
Cas1
Cas9
46447796 Desulfovibrio vulgaris str. Hildenborough
11500011 Archaeoglobus fulgidus DSM 4304
18977490 Pyrococcus furiosus DSM 3638
16130662 Escherichia coli K-12 MG1655
Cas1 outgroup
(type I-A, I-C, I-B and I-E)
II-B
II-A
II-C
34557933 Wolinella succinogenes DSM 1740
331001028 Parasutterella excrementihominis YIT 11859
319941582 Sutterella wadsworthensis 3 1 45B
54296139 Legionella pneumophila str. Paris
118497353 Francisella novicida U112
323463802 Staphylococcus pseudintermedius ED99
389815358 Planococcus antarcticus DSM 14505
339625080 Fructobacillus fructosus KCTC 3544
258509198 Lactobacillus rhamnosus GG
336394883 Lactobacillus farciminis KCTC 3681
310286727 Bifidobacterium bifidum S17
366983954 Oenococcus kitaharae DSM 17330
13622194 Streptococcus pyogenes M1 GAS
24379808 Streptococcus mutans UA159
422884107 Streptococcus sanguinis SK49
116628212 Streptococcus thermophilus LMD-9
320528779 Solobacterium moorei F0204
303229394 Veillonella atypica ACS-134-V-Col7a
227824982 Acidaminococcus sp. D21
169823756 Finegoldia magna ATCC 29328
374307737 Filifactor alocis ATCC 35896
306821690 Eubacterium yurii ATCC 43715
291520706 Coprococcus catus GD-7
42525844 Treponema denticola ATCC 35405
304438953 Peptoniphilus duerdenii ATCC BAA-1640
256845020 Fusobacterium sp. 3 1 36A2
328956316 Coriobacterium glomerans PW2
47458867 Mycoplasma mobile 163K
363542551 Mycoplasma ovipneumoniae SC01
71894593 Mycoplasma synoviae 53
284931711 Mycoplasma gallisepticum str. F
384393287 Mycoplasma canis PG 14
315659847 Staphylococcus lugdunensis M23590
160915783 Eubacterium dolichum DSM 3991
238924076 Eubacterium rectale ATCC 33656
315149831 Enterococcus faecalis TX0012
116627543 Streptococcus thermophilus LMD-9
218563120 Campylobacter jejuni NCTC 11168
291276264 Helicobacter mustelae 12198
325677757 Ruminococcus albus 8
421874296 Brevibacillus laterosporus GI-9
225377803 Roseburia inulinivorans DSM 16841
220930481 Clostridium cellulolyticum H10
310780383 Ilyobacter polytropus DSM 2926
222109284 Acidovorax ebreus TPSY
403744859 Alicyclobacillus hesperidum URH17-3-68
312879014 Aminomonas paucivorans DSM 12260
336393380 Lactobacillus coryniformis KCTC 3535
218767587 Neisseria meningitidis Z2491
15602991 Pasteurella multocida str. Pm70
296446028 Methylosinus trichosporium OB3b
34557789 Wolinella succinogenes DSM 1740
294086112 Cand. Puniceispirillum marinum IMCC1322
402849996 Rhodovulum sp. PH10
159042957 Dinoroseobacter shibae DFL 12
288957740 Azospirillum sp. B510
330822846 Alicycliphilus denitrificans K601
344171926 Ralstonia syzygii R24
154250554 Parvibaculum lavamentivorans DS-1
282880053 Prevotella timonensis CRIS 5C-B1
374384762 Odoribacter laneus YIT 12061
423317188 Bergeyella zoohelcum ATCC 43767
385811610 Ignavibacterium album JCM 16511
402847305 Porphyromonas sp. oral taxon 279 str- F0450
345885719 Prevotella sp. C561
404487227 Barnesiella intestinihominis YIT 11860
301311870 Bacteroides sp. 20 3
60683388 Bacteroides fragilis NCTC 9343
187736488 Akkermansia muciniphila ATCC BAA-835
319957207 Nitratifractor salsuginis DSM 16511
407803668 Alcanivorax sp. W11-5
325972002 Sphaerochaeta globus str. Buddy
187250661 Elusimicrobium minutum Pei191
117929157 Acidothermus cellulolyticus 11B
317482065 Bifidobacterium sp. 12 1 47BFAA
315605739 Actinomyces sp. oral taxon 180 str- F0310
0.5
118497352 Francisella novicida U112
254447899 gamma proteobacterium HTCC5015
331001027 Parasutterella excrementihominis YIT 11859
319941583 Sutterella wadsworthensis 3 1 45B
54296138 Legionella pneumophila str. Paris
34557932 Wolinella succinogenes DSM 1740
323463801 Staphylococcus pseudintermedius ED99
389815359 Planococcus antarcticus DSM 14505
422884106 Streptococcus sanguinis SK49
13622193 Streptococcus pyogenes M1 GAS
116628213 Streptococcus thermophilus LMD-9
24379809 Streptococcus mutans UA159
328956315 Coriobacterium glomerans PW2
336394882 Lactobacillus farciminis KCTC 3681
224543312 Catenibacterium mitsuokai DSM 15897
258509199 Lactobacillus rhamnosus GG
310286728 Bifidobacterium bifidum S17
366983953 Oenococcus kitaharae DSM 17330
339625081 Fructobacillus fructosus KCTC 3544
169823755 Finegoldia magna ATCC 29328
303229466 Veillonella atypica ACS-134-V-Col7a
320528778 Solobacterium moorei F0204
227824983 Acidaminococcus sp. D21
306821691 Eubacterium yurii ATCC 43715
291520705 Coprococcus catus GD-7
34762592 Fusobacterium nucleatum ATCC 49256
374307738 Filifactor alocis ATCC 35896
304438954 Peptoniphilus duerdenii ATCC BAA-1640
42525843 Treponema denticola ATCC 35405
315659848 Staphylococcus lugdunensis M23590
160915782 Eubacterium dolichum DSM 3991
116627542 Streptococcus thermophilus LMD-9
315149830 Enterococcus faecalis TX0012
238924075 Eubacterium rectale ATCC 33656
47458868 Mycoplasma mobile 163K
363542550 Mycoplasma ovipneumoniae SC01
284931710 Mycoplasma gallisepticum str. F
71894592 Mycoplasma synoviae 53
384393286 Mycoplasma canis PG 14
34557790 Wolinella succinogenes DSM 1740
218563121 Campylobacter jejuni NCTC 11168
291276265 Helicobacter mustelae 12198
296446027 Methylosinus trichosporium OB3b
310780384 Ilyobacter polytropus DSM 2926
365156657 Bacillus smithii 7 3 47FAA
220930482 Clostridium cellulolyticum H10
222109285 Acidovorax ebreus TPSY
218767588 Neisseria meningitidis Z2491
15602992 Pasteurella multocida str. Pm70
312879015 Aminomonas paucivorans DSM 12260
336393381 Lactobacillus coryniformis KCTC 3535
225377804 Roseburia inulinivorans DSM 16841
403744858 Alicyclobacillus hesperidum URH17-3-68
297182908 uncultured delta proteobacterium HF0070 07E19
325677756 Ruminococcus albus 8
384109266 Treponema sp. JC4
407803669 Alcanivorax sp. W11-5
427429481 Caenispirillum salinarum AK4
83591793 Rhodospirillum rubrum ATCC 11170
344171927 Ralstonia syzygii R24
402849997 Rhodovulum sp. PH10
330822845 Alicycliphilus denitrificans K601
294086111 Cand. Puniceispirillum marinum IMCC1322
288957741 Azospirillum sp. B510
92109262 Nitrobacter hamburgensis X14
148255343 Bradyrhizobium sp. BTAi1
159042956 Dinoroseobacter shibae DFL 12
154250555 Parvibaculum lavamentivorans DS-1
423317190 Bergeyella zoohelcum ATCC 43767
301311869 Bacteroides sp. 20 3
385811609 Ignavibacterium album JCM 16511
60683389 Bacteroides fragilis NCTC 9343
402847315 Porphyromonas sp. oral taxon 279 str. F0450
404487228 Barnesiella intestinihominis YIT 11860
374384763 Odoribacter laneus YIT 12061
347536497 Flavobacterium branchiophilum FL-15
345885718 Prevotella sp. C561
282880052 Prevotella timonensis CRIS 5C-B1
187250660 Elusimicrobium minutum Pei191
325972003 Sphaerochaeta globus str. Buddy
117929158 Acidothermus cellulolyticus 11B
315605738 Actinomyces sp. oral taxon 180 str. F0310
227494853 Actinomyces coleocanis DSM 15436
189440764 Bifidobacterium longum DJO10A
187736489 Akkermansia muciniphila ATCC BAA-835
319957206 Nitratifractor salsuginis DSM 16511
0.2

Supplementary Figure S4. Phylogenetic analysis of representative Cas9 and Cas1 sequences. Phylogenetic trees of Cas1 (left) and Cas9 (right) reconstructed from selected, informative positions of Cas1 and Cas9 multiple sequence alignments are shown (see Figure 1 and Supplementary Figures S2 and S3). The Cas1 tree is rooted to the outgroup of selected Cas1 orthologs of type I CRISPR-Cas systems. The Cas1 and Cas9 orthologs of the types classified as II-A, II-B and II-C are highlighted with shaded boxes. The same branch colors were used for each bacterial strain on both trees. Each protein is represented by the GenInfo (GI) identifier followed by the bacterial strain name. The bootstrap values are given for each node (see Materials and Methods). The scale bars for the branch length are given as the estimated number of amino acid substitution per site. Note the similarity of the trees topology and monophyletic clusters of subtypes II-A and II-B on both trees supported by high bootstrap values.

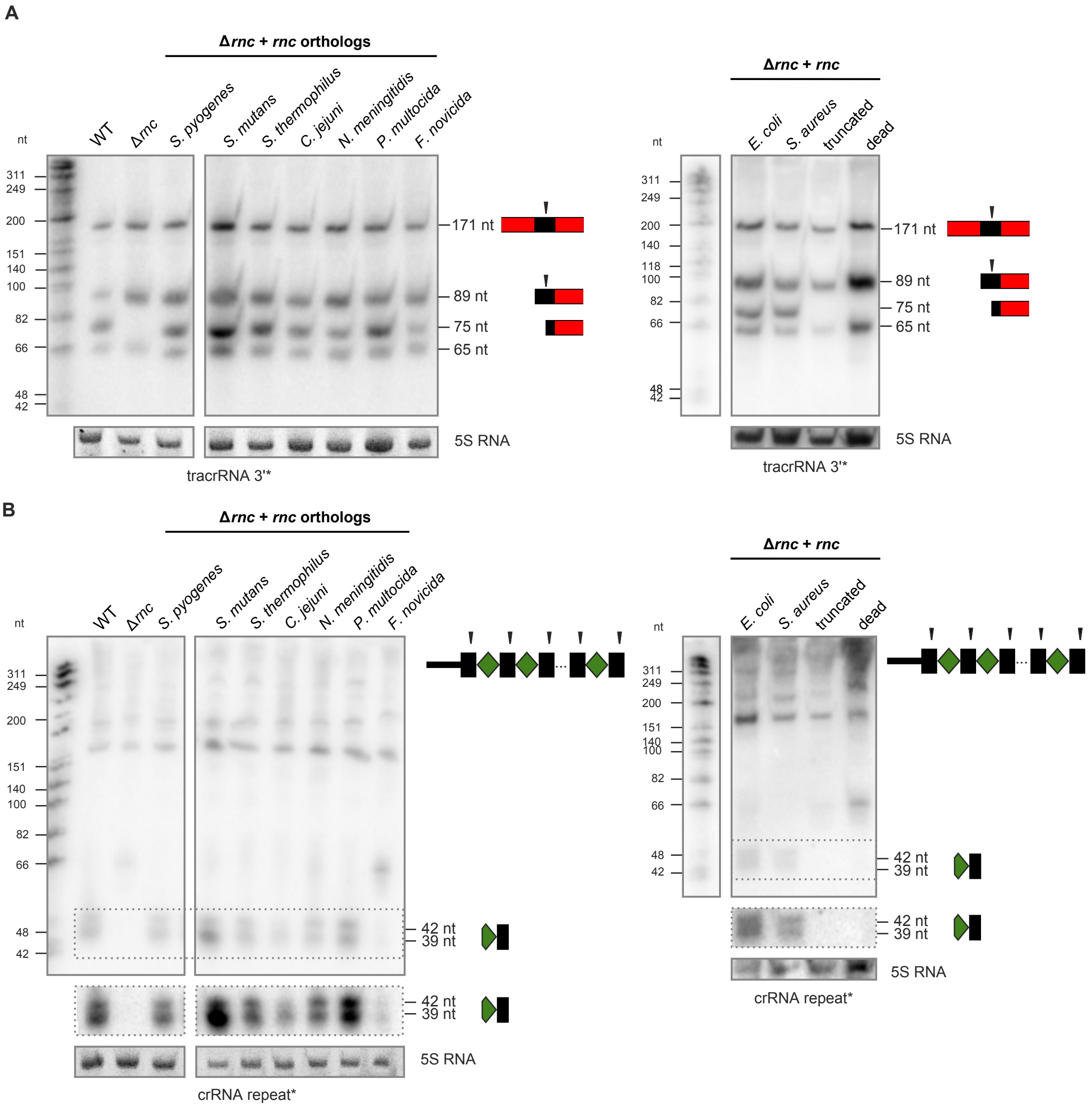

Supplementary Figure S5. RNase III is a general executioner of tracrRNA:pre-crRNA processing in type II CRISPR-Cas. Northern blot analysis of total RNA from *S. pyogenes* WT, Δ*rnc* and Δ*rnc* complemented with *rnc* orthologs or *rnc* mutants probed with (**A**) tracrRNA and (**B**) crRNA repeat (Supplementary Table S1). The dashed-line boxes represented below the Northern blots in (**B**) show the area of the blots with enhanced exposure. All RNAse III orthologs can co-process *S. pyogenes* tracrRNA and pre-crRNA. No mature forms of tracrRNA and crRNAs could be observed in Δ*rnc* complemented with the truncated version or catalytically inactive (dead) mutant of RNase III.

```
                                                                                   *
15674631  Streptococcus pyogenes SF370        ------MKQLEELLSTSF-------DIQFNDLTLLETAFTHTSYANEHRLLNVSHNERLEFLGDAVLQLIISEYLFAKYPKKTEGDMSKLRSMIVREESLAGFSRFCSFDAYIKLGKGEEKSGGRRRDTILGDLFEAFLGALLLDKGIDAVR
24379904  Streptococcus mutans UA159          ------MKTLEKKLAEDF-------KIVFSDKELLETAFTHTSYANEHRLLNISHNERLEFLGDAVLQLTISHYLFDKYPQKAEGDLSKMRSMIVREESLAGFSRNCHFDRYIKLGKGEEKSGGRQRDTILGDLFEAFLGALLLDAGLKAVE
116628032 Streptococcus thermophilus LMD-9    ------MNQLEQKLEQDF-------GIVFSQKDLLETAFTHTSYANEHRLLNISHNERLEFLGDAALQLVISVYLYNRYPNKPEGEMSKMRSTIVREESLAGFTKACGFEQYIRLGKGEEKSGGRERATILGDLWEAFLGALYLDQGLPAVE
57651802  Staphylococcus aureus COL           -MSKQKKSEIVNRFRKRFDTKMTELGFTYQNIDLYQQAFSHSSFINDFNMNRLDHNERLEFLGDAVLELTVSRYLFDKHPNLPEGNLTKMRATIVCEPSLVIFANKIGLNEMILLGKGEEKTGGRTRPSLISDAFEAFIGALYLDQGLDIVW
118498035 Francisella novicida U112           ------MVPEYSRFYNIL-------GYNFKDYTLLIRALTHRSKTKK-------NYERLEFLGDSVLSFVIAEVLYKQFTDLAEGKLSQLRSKLVKGTTLAQLASSLKMDEYIILGASEQ--GGHKREKILEDVFEAVIGAIYLDSDFATVK
218767809 Neisseria meningitidis Z2491        MKDDVLKQQAHAAIQKKL-------GYAFRDISLLRQALTHRSHHAK-------HNERFEFVGDSILNYTVARMLFDAFPKLTEGELSRLRASLVNEGVLAEMAAEMNVGDGLYLGAGELKSGGFRRPSILADAMEAMFAAVSFDADFNTAE
15601926  Pasteurella multocida Pm70          ------MTQNLERLQRQI-------GYQFNQPALLKQALTHRSAAVK-------HNERLEFLGDAILNFIIAEALYHQFPKCNEGELSRMRATLVREPTLASLARQFELGDYLSLGPGELKSGGFRRESILADCVEAIIGAISLDSDLATTT
218563224 Campylobacter jejuni NCTC 11168     -------MKNIEKLEQSL-------TYEFKDKNLLIHALTHKSFKKSY------NNERLEFLGDAVLDLVVGEYLFHKFAKDAEGDLSKLRAALVNEKSFAKIANSLNLGDFILMSVAEENNGGKEKPSILSDALEAIIGAIHLEAGFEFAK
16130492  Escherichia coli K-12               -----MNPIVINRLQRKL-------GYTFNHQELLQQALTHRSASSK-------HNERLEFLGDSILSYVIANALYHRFPRVDEGDMSRMRATLVRGNTLAELAREFELGECLRLGPGELKSGGFRRESILADTVEALIGGVFLDSDIQTVE

Motifs                                            [____________________________________________________________Cutting domain____________________________________________________________________]


                                                       ↓
15674631  Streptococcus pyogenes SF370        RFLKQVMIPQVEKGNFE--RVKDYKTCLQEFLQTKGDVAIDYQVISEKGPAHAKQFEVSIVVNG-AVLSKGLGKSKKLAEQDAAKNALAQLSEV--------
24379904  Streptococcus mutans UA159          AFLNQVVIPKVENNNYE--RVTDYKTALQELLQVDGDVLIDYEVLKESGPAHAKCFEVAVSMNH-EKLSSGTGKSKKLAEQEAAKNALEKLQRGS-------
116628032 Streptococcus thermophilus LMD-9    KFLNQVMIPQVEKGNFD--RVIDYKTALQERLQVNGKVDITYTVIDESGPAHAKEFTMQVAVDG-KELSTGFGKSKKLAEQAAAKSALEQLGN---------
57651802  Staphylococcus aureus COL           KFAEKVIFPHVEQNELL--GVVDFKTQFQEYVHQQNKGDVTYNLIKEEGPAHHRLFTSEVILQG-EAIAEGKGKTKKESEQRAAESAYKQLKQIK-------
118498035 Francisella novicida U112           KVILKWYQPIISSINLDTIKVKDSKSKLQEILLQNALSLPEYSIETIDGKDHEQQFTVVAMSKDLNLRVKAQGTSRKKAEQKAAEKMIEMLSQQGLHEKK--
218767809 Neisseria meningitidis Z2491        KVVRHLFAERVRRVDFQN-QAKDGKTALQEALQARRFALPKYRIEEQIGHANDSMFVISCDLGELGFVCRAKGTSRKAAEQEAAKEALKWLEEKLPLKKKKK
15601926  Pasteurella multocida Pm70          KIVQHWYQAQLKQIQPGD-NQKDPKTRLQEYLQGKRLPLPTYNVVEIKGEAHCQTFTVECYVKNIDRTFMGSGASRRKAEQAAAEKILQLLEMK--------
218563224 Campylobacter jejuni NCTC 11168     TIALRLIEKNFPQIDAKI-LIKDYKTKLQEITQGKIGQTPQYETVRAFGPDHLKQFEIALMLDG-KELARAIAGSKKEAQQMAAKIALEKLGAL--------
16130492  Escherichia coli K-12               KLILNWYQTRLDEISPGD-KQKDPKTRLQEYLQGRHLPLPTYLVVQVRGEAHDQEFTIHCQVSGLSEPVVGTGSSRRKAEQAAAEQALKKLELE--------

Motifs                                                    [__________________________dsRNA binding domain__________________________]
```

Supplementary Figure S6. Multiple sequence alignment of bacterial endoribonucleases III used in the study. Domains indicated below the alignment are according to the domains identified in RNase III from *E. coli* (58, 59). The conserved catalytic aspartate residue mutated in the catalytically inactive “*rnc* dead” mutant and the last amino acid of the truncated *rnc* mutant are indicated above the alignment with an asterisk and an arrow, respectively.

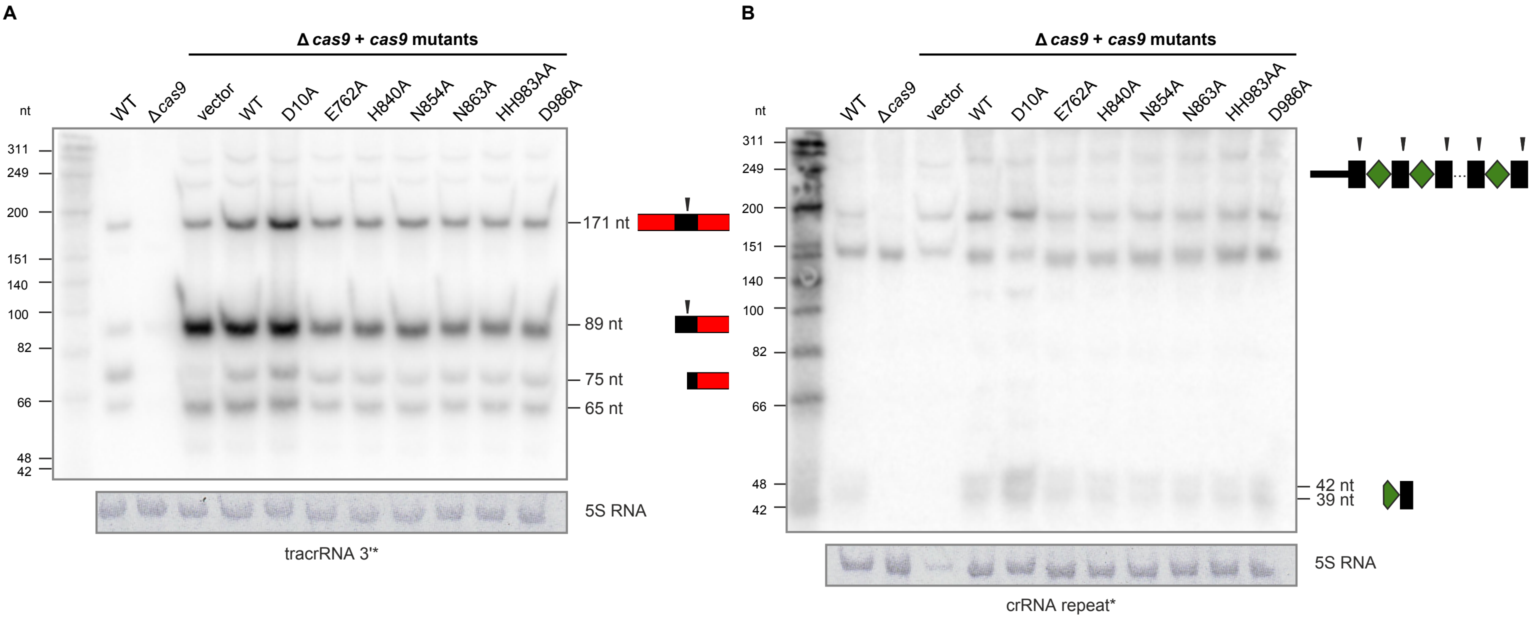

Supplementary Figure S7. Conserved catalytic amino acid residues of Cas9 are not involved in dual-RNA processing by RNase III. Northern blot analysis of total RNA extracted from *S. pyogenes* WT, Δ*cas9* and Δ*cas9* complemented with pEC342 (backbone vector containing tracrRNA-171 nt and the native *cas* operon promoter from *S. pyogenes*) or pEC342-derived plasmids encoding Cas9 WT or mutants, hybridized with (**A**) tracrRNA or (**B**) crRNA repeat probe (Supplementary Table S1). tracrRNA:crRNA co-processing is observed in all strains encoding Cas9 point mutants. Note that in a previous study, we observed low abundance of tracrRNA in the *cas9* deletion mutant (16). For this reason, plasmids used in *cas9* complementation studies were designed to encode tracrRNA in addition to *cas9*.

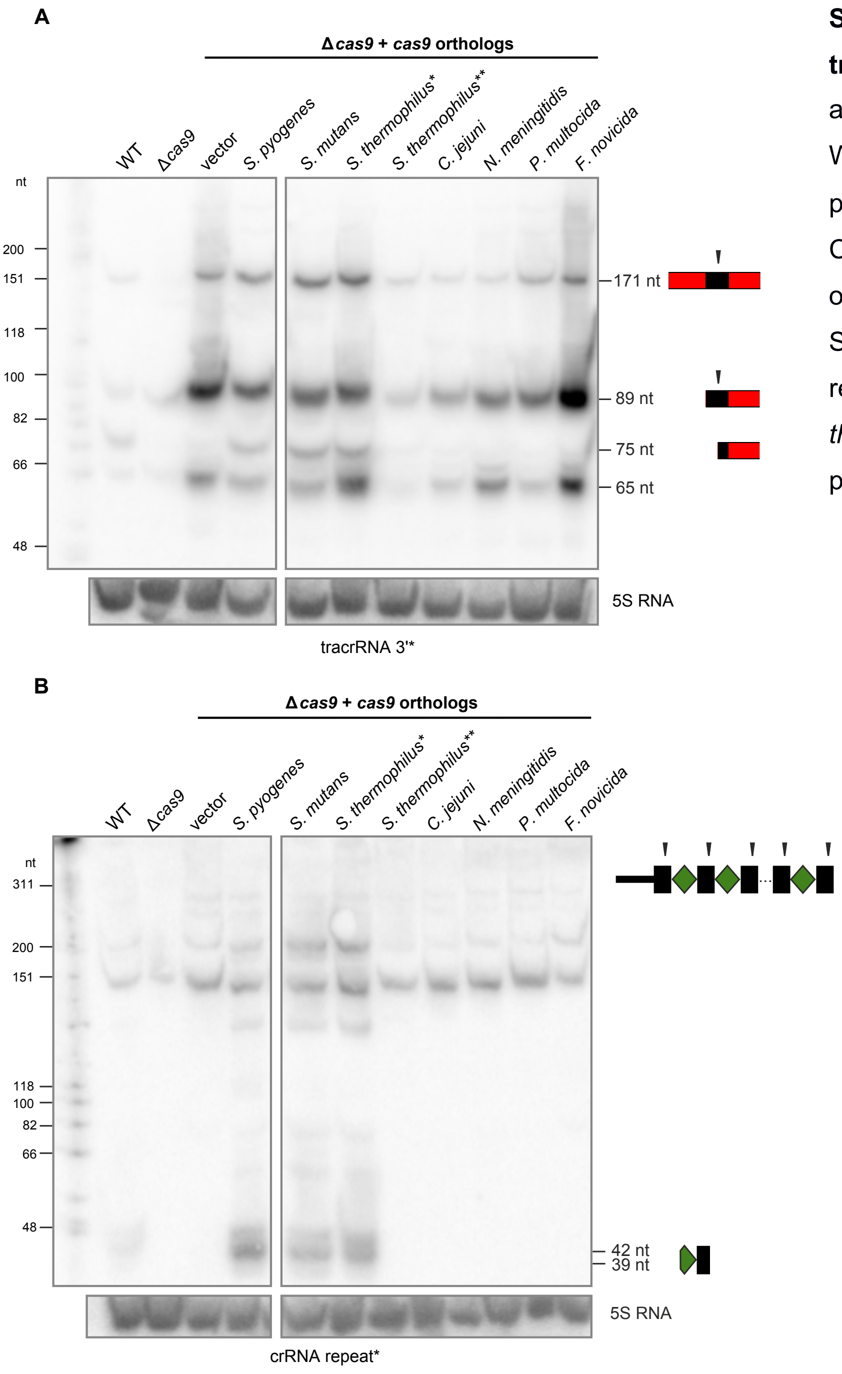

Supplementary Figure S8. Cas9 and tracrRNA:crRNA co-evolved. Northern blot analysis of total RNA extracted from *S. pyogenes* WT, Δ*cas9* and Δ*cas9* complemented with pEC342 or pEC342-derived plasmids encoding Cas9 WT or mutants, hybridized with (**A**) tracrRNA or **(B)** crRNA repeat probe (Supplementary Table S1). Only *S. pyogenes* Cas9 WT and closely related Cas9 orthologs from *S. mutans* and *S. thermophilus** (CRISPR3) can contribute to co-processing of *S. pyogenes* tracrRNA:pre-crRNA**.**

Supplementary Table S3. **Overview of type II CRISPR-Cas spacer sequences from selected bacterial strains with BLAST candidate protospacers and their downstream sequence.**

Strain[a]	Number of spacers	CRISPR Spacer[b]	Spacer sequence	Blast candidate[c]	% Identity[d]	10 bp downstream protospacer[e]
Streptococcus pyogenes SF370 (Accession: NC_002737)	6	1	TGCGCTGGTTGATTTCTTCTTGCGCTTTTT	*S. pyogenes* MGAS1882 (MGAS1882_1116), MGAS8232 (spyM18_0769), MGAS10394 (M6_Spy0995, M6_Spy1349), SSI-1 (SPs0926), ΦP9 **endopeptidase gene**	**100**	TGGCTTTTTC
				S. pyogenes MGAS2096 (MGAS2096_Spy1450), A20 (A20_1472c), M1 476 (M1GAS476_1503), MGAS9429 (MGAS9429_Spy1426), MGAS5005 (M5005_Spy1424) **endopeptidase gene**	**97**	TGGCTTTTTC
				S. pyogenes M1 GAS (SPy_0700), MGAS2096 (MGAS2096_Spy0592) **endopeptidase gene**	**97**	TTGCTTTTTC
				S. pyogenes MGAS6180 (M28_Spy1234); NIH1 (NIH1.1_43), SSI-1 (SPs0647), MGAS315 (SpyM3_0930, SpyM3_1215) **phage related gene**	**100**	TGACTTTTTC
		2	TTATATGAACATAACTCAATTTGTAAAAAA	**gene for pyrogenic exotoxin M** (*speM*) of several Streptococci strains	**100**	GGGTATTGGG
		3	AGGAATATCCGCAATAATTAATTGCGCTCT	*S. pyogenes* MGAS8232 (spyM18_0742), MGAS10750 (MGAS10750_Spy0588), MGAS10270 (MGAS10270_Spy0563) **adenine specific methylase gene**	**100**	TGGTATGTTG
				S. pyogenes Manfredo (SpyM50653) **adenine specific methylase gene**	**97**	TGGTATGTTG
		4	AGTGCCGAGGAAAAATTAGGTGCGCTTGGC	*S. pyogenes* Alab49 (SPYALAB49_001176), MGAS10750 (MGAS10750_Spy1285), MGAS9429 (MGAS9429_Spy0843), MGAS10394 (M6_Spy1203), SSI-1 (SPs0763), MGAS315 (SpyM3_1101), ΦH4489A (hylP) **hyaluronoglucosaminidase gene**	**100**	TGGCGCATTA
				S. pyogenes MGAS8232 (spyM18_1254), NZ131 (Spy49_0785) **hyaluronoglucosaminidase gene**	**97**	TGGCGCATTA

Strain[a]	Number of spacers	CRISPR Spacer[b]	Spacer sequence	Blast candidate[c]	% Identity[d]	10 bp downstream protospacer[e]
		5	TAAATTTGTTTAGCAGGTAAACCGTGCTTT	*S. pyogenes* MGAS10750 (MGAS10750_Spy0839), MGAS10270 (MGAS10270_Spy0546, MGAS10270_Spy0804), SSI-1 (SPs0517, SPs0888), MGAS1882 (MGAS1882_1156), MGAS8232, NZ131(Spy49_1511c), MGAS315 (SpyM3_0965, SpyM3_1347)<br>**phage protein gene or intergenic region**	100	TGGTTATATC
Streptococcus mutans UA159 (Accession: NC_004350)	5	3	CTAACTATGATGACACAACAGCTTTTAGCG	ΦM102 (orf13)<br>**putative tail protein gene**	100	TTGAAATTTC
Streptococcus mutans LJ23 (Accession: NC_017768)	8	2	TGAAGTGCAAGCTTACGTGACTGACTCGCG	ΦM102 (orf15)<br>**putative minor structural protein**	90	AGGTATGCAG
Streptococcus mutans GS-5 (Accession: NC_018089)	21	3	TAATAGCAATCGTGACGGACGTATTGATTT	ΦM102 (orf15)<br>**putative minor structural protein**	97	AGGTGAAATT
		5	GTTGAGTGCAACAGCTAGCTAATAGCTTTT	ΦM102	100	AGGCTGGCAC
		16	AGGCATTTTCTGATTGAGATTTTCGATATT	ΦM102 (orf3)<br>**putative large terminase gene**	93	TGGAAAGATG
		18	TATAGCTAATATGTGTATACTGACAGCGCA	ΦM102 (orf7)<br>**putative DNA packaging protein gene**	100	AGAAAGATTG
Streptococcus mutans NN2025 (Accession: NC_013928)	69	2	GATTGTGCCCGCTAGTAAACCGCCTCGCGC	ΦM102 (orf20)<br>**putative endolysin gene**	93	TGGAGATTTG
		6	GATTGTATCAGTAATCGAACTTCTGCTTAT	ΦM102 (orf38, orf39)<br>**hypothetical protein gene**	93	GGGATTTGAC
		8	TGGTCCAAAGTGCAGAGCCAAAGAAAAACA	ΦM102 (orf11)<br>**putative major tail protein gene**	97	AAGCGGTCTT
		9	ATTGTCAATCGCCGTTCTGCGCTTGCGACG	ΦM102 (orf17)<br>**hypothetical protein gene**	90	CGGTTTTGAA
		17	GCTTGAATATAATTGTGTATCCGCCAATGA	ΦM102 (orf21)<br>**putative replisome organizer gene**	93	CGAATTACGA

Strain[a]	Number of spacers	CRISPR Spacer[b]	Spacer sequence	Blast candidate[c]	% Identity[d]	10 bp downstream protospacer[e]
		**23**	AAAAAGAAACGCCTTTTGATTTGACCAATC	ΦM102 (orf14) **putative receptor-binding protein gene**	**90**	AAGAGCAAGA
		**29**	AGTTATTAATATCTATGACAGTCTCAAAGA	ΦM102 (orf14) **putative receptor-binding protein gene**	**93**	GGGCTACAGA
		**37**	TTCTGGCTGTCTTTCAGAGTGATAAGCGCA	ΦM102 (orf2) **putative small terminase gene**	**100**	GGATTTTTCA
		**40**	TGCAAGTTATCTTGCTATGTGGACGAATTG	ΦM102 (orf9) **hypothetical protein gene**	**93**	GGGAACAATC
		**43**	GCAATTTAGTTTTATTCCGTGGGAGCAGCA	ΦM102 (orf3) **putative large terminase gene**	**93**	AGGCTCCATT
		**48**	AGAGTATAGCCAGTGTTTTCAAGGCCTTTA	ΦM102 (orf12) **putative tape measure protein gene**	**93**	GTGGTGACAA
		**49**	CGCAACAATGACTATTAATATCAACGGTGG	ΦM102 (orf15) **putative minor structural protein gene**	**93**	CGGGAGCAAT
		**56**	AATCGCTTCTTTGCTAACCACAATTTGTGC	ΦM102 (orf26) **putative RecT family single-strand annealing protein gene**	**93**	AGGCGCAGAG
		**60**	AAATGCTCTTGAAGAACCTGATAGATGACA	ΦM102 (orf3) **putative large terminase gene**	**93**	GAGACGAAAA
		**66**	TGCAAAAGATGGCCTCGAGCAATTATCGCA	ΦM102 (orf33) **hypothetical protein gene**	**100**	TGGATTAAGC
Streptococcus thermophilus LMD-9 CASS4 locus (Accession: NC_008532)	**8**	**2**	TCAATGAGTGGTATCCAAGACGAAAACTTA	*Streptococcus thermophilus* plasmid pSt106 **putative resolvase gene**	**100**	TGGCAAGTTT
		**3**	CCTTGTCGTGGCTCTCCATACGCCCATATA	*Streptococcus thermophilus* plasmid pND103	**100**	AGGGGCGGGT
		**4**	TGTTTGGGAAACCGCAGTAGCCATGATTAA	Φ7201 (orf33)	**100**	AGGTGTCGCT

Strain[a]	Number of spacers	CRISPR Spacer[b]	Spacer sequence	Blast candidate[c]	% Identity[d]	10 bp downstream protospacer[e]
		**5**	ACAGAGTACAATATTGTCCTCATTGGAGACAC	Φ TP-J34 (orf11)<br>**hypothetical protein gene**	**94**	TGGGGTAGGA
		**6**	CTCATATTCGTTAGTTGCTTTTGTCATAAA	ΦSfi19 (orf1626)<br>**minor tail protein gene**	**100**	TGGTGCTAAT
				ΦYMC 2011 (Ssal_phage00063)<br>**putative minor tail protein gene**	**90**	GGGTGCTAAC
				Φ7201 (orf33)	**90**	TGGTGCTAGA
Streptococcus thermophilus LMD-9 CASS4a locus (Accession: NC_008532)	**16**	**2**	CTTCACCTCAAATCTTAGAGCTGGACTAAA	Φ7201 (orf39)	**100**	GTAGAATAGA
		**3**	ATGTCTGAAAAATAACCGACCATCATTACT	Φ TP-J34 (orf49), ΦSfi11 (orf669)<br>**putative minor structural protein gene**	**93**	CCAGAATGTC
		**4**	GAAGCTCATCATGTTAAGGCTAAAACCTAT	ΦALQ13.2 (orf35)<br>**helicase gene**	**90**	CTAAAAATTA
				ΦSfi11 (orf443), ΦSFi18 (orf443), ΦSfi21 (orf443), ΦSfi19 (orf443), ΦO1205 (orf10)<br>**putative helicase gene**	**90**	CTCAAAATTA
		**5**	TAGTCTAAATAGATTTCTTGCACCATTGTA	Φ1033, Φ 1042<br>**nonfunctional host specificity protein gene**	**97**	ATAAAATTCA
				ΦDT1.1 (orf18), ΦDT1.2 (orf18), ΦDT1.3 (orf18), ΦDT1.4 (orf18), ΦDT1.5 (orf18), ΦMD4 (orf18)<br>**host specificity protein gene**	**93**	ATAAAATTCA
		**6**	ATTCGTGAAAAAATATCGTGAAATAGGCAA	pSt08 plasmid	**97**	CCGAAAAATA
		**7**	TCTAGGCTCATCTAAAGATAAATCAGTAGC	ΦALQ13.2 (orf25), Φ858 (orf30), ΦST3 (orf253)<br>**endonuclease gene**	**90**	TGAAAAATTA
				ΦJ1 (orf253), ΦS3b (orf253)<br>**endonuclease gene**	**90**	TGAAAGATTA
		**13**	AACTACCAAGCAAATCAGCAATCAATAAGT	ΦSfi11	**100**	TCAAGAATAT

Strain[a]	Number of spacers	CRISPR Spacer[b]	Spacer sequence	Blast candidate[c]	% Identity[d]	10 bp downstream protospacer[e]
				ΦYMC-2011 (Ssal_phage00051) **predicted clp-protease gene**	**93**	TTAAGAACAT
				ΦSfi21 (orf221) **clp-protease gene**	**90**	TCAAGAATAT
				Φ858 (orf22)	**93**	AAAAAAAACT
		**16**	AACAGTTACTATTAATCACGATTCCAACGG	Φ2972 (orf21) **structural protein gene**	**93**	AAAAAAAACT
				ΦAbc2 (orf17) **tail protein gene**	**93**	TAAAAAGACT
Campylobacter jejuni subsp. *jejuni* NCTC 11168 (Accession: NC_002163)	**5**	**1-5**		no significant BLAST hits		
Campylobacter jejuni subsp. *jejuni* CF93-6 (Accession: AANJ00000000)	**5**	**3**	TCATCATCACTTAAAACCTTAAATTTACC	*C. jejuni* RM1221 (CJE1445) **hypothetical protein gene**	**93**	ATAACGCAAG
Campylobacter jejuni subsp. *jejuni* HB93-13c_jejuni_subsp_jejunihb_13_42 (Accession: AANQ00000000)	**9**	**1**	GCATTGCTTTACTACATAGCCAGTCGTGTA	*C. jejuni* subsp. *doylei* 269.97 (JJD26997_1148) **conserved hypothetical protein gene**	**100**	TCACACACGC
Campylobacter jejuni subsp. *jejuni* NW	**5**	**2**	TTATTTTTGTCGCTAATTGCACCTAAAGAC	*C. jejuni* subsp. *doylei* 269.97 (JJD26997_0867) **putative primase gene**	**97**	TCCAACACAT

Strain[a]	Number of spacers	CRISPR Spacer[b]	Spacer sequence	Blast candidate[c]	% Identity[d]	10 bp downstream protospacer[e]
genomic scaffold Mich_State_Univ:Contig3 (Accession: JH376989 REGION: 13521..15062)		5	GGGACACGAGGAATCCTGTCTGAATCCGGG	*C. jejuni* subsp. *jejuni* PT14 (A911_r08426, A911_r08428, A911_r08430), NCTC 11168-BN148 (BN148_r02, BN148_r05, BN148_r08), S3 (CJS3_1811, CJS3_1817, CJS3_1830), ICDCCJ07001 (ICDCCJ07001_29, ICDCCJ07001_396, ICDCCJ07001_718), M1 (CJM1_0031, CJM1_0413, CJM1_0727), IA3902 (CJSA_Cj23SA, CJSA_Cj23SB, CJSA_Cj23SAC), BABS091400, 81116 (C8J_Cj23SA, C8J_Cj23SB, C8J_Cj23SC), 81-176 (CJJ81176_1714, CJJ81176_1727, CJJ81176_1707), NCTC 11168; *C. jejuni* DSM 4688, UNSW091300, strain 100, RP0001, 102-27 (rrlC, rrlB, rrlA), 69-30 (rrlC, rrlB, rrlA), 140-16 (rrlC, rrlB, rrlA), 110-21 (rrlC, rrlB, rrlA), RM1221 (CJE_Cj23SA, CJE_Cj23SB, CJE_Cj23SC), TGH9011_ATCC43431 (rrl); *C. coli* 59-2 (rrlC, rrlB, rrlA); *C. jejuni* subsp. *doylei* 269.97 (JJD26997_0040, JJD26997_1264, JJD26997_1520)<br>**23S rRNA gene**	**100**	TCGACCACGA
Campylobacter jejuni subsp. *doylei* 269.97 (Accession: NC_009707)	**5**	**2**	CTAAGCAATCTTATTTTACCATCTTTTTTA	*C. jejuni* strain TGH 9011 (Tgh093)	**97**	TAAAACACTT
				C. jejuni RM1221 (CJE1099)<br>**hypothetical protein gene**	**93**	TAAAACACTT
Campylobacter jejuni subsp. *jejuni* 1336 (Accession: NZ_CM000854 NZ_ADGL01000000)	**2**	**1**	TTACTGATATTAAAATTAACTCCATAATTT	*C. jejuni* 00-3477 (cje0227), *C. jejuni* subsp. *jejuni* S3 (CJS3_0723), ΦCGC-2007<br>**prophage related genes**	**100**	GCTGCCATTA
				C. jejuni NCTC 13255 (putative CJIE1-2-like prophage), 99-7046 (putative CJIE1-3-like prophage), 00-2425 (putative CJIE1 prophage), RM1221 (CJE0227)*C. jejuni* subsp. *jejuni* ICDCCJ07001 (ICDCCJ07001_691)<br>**major tail sheath protein**	**93**	GCTGCCATTA
		**2**	ATAAAGCTAATGCAAAAGTTGAAAACAAA	*C. jejuni* NCTC 13255 (putative CJIE1-2-like prophage), 99-7046 (putative CJIE1-3-like prophage), 00-3477 (putative CJIE1-4 Mu-like prophage), 00-2425 (putative CJIE1 prophage), RM1221 (CJE0238),*C. jejuni* subsp. *jejuni* S3 (CJS3_0704), ICDCCJ07001, *C. hyoilei*<br>**hypothetical protein gene**	**100**	AGAGCTATAA
Campylobacter jejuni subsp. *jejuni* 414 (Accession: NZ_CM000855 NZ_ADGM01000000)	**33**	**2**	TTTATCTGCATCCATAATGGCAATGAGTGA	*C. jejuni* subsp. *jejuni* PT14 (A911_03310), NCTC 11168-BN148 (BN148_0680c), S3 (CJS3_0675), ICDCCJ07001 (ICDCCJ07001_619), M1 (CJM1_0650), IA3902 (CJSA_0644), 81116 (C8J_0632), 81-176 (CJJ81176_0703), NCTC 11168 (Cj0680c), P694a (Cj0680c), P569a (Cj0680c*)*, P179a (Cj0680c), H73020 (Cj0680c), H704a (Cj0680c), *C. jejuni* RM1221 (CJE0778), *C. jejuni* subsp. *doylei* 269.97 (JJD26997_1327)<br>**excinuclease ABC subunit B gene**	**97**	AACTTCAGGC

Strain[a]	Number of spacers	CRISPR Spacer[b]	Spacer sequence	Blast candidate[c]	% Identity[d]	10 bp downstream protospacer[e]
Neisseria meningitidis serogroup A strain Z2491 (Accession: NC_003116)	16	2	CTTCTGCCTTTTTACAAGCTCGCTTTCTTT	*N. gonorrhoeae* (NGU65994, PivNG), FA 1090 (NGO1137, NGO1164, NGO1262) **invertase related genes, phage associated protein genes**	97	CGCCGACCGG
		3	TTTGGTAAAGGTTTCTGTTGCGACCCGAAT	*N.meningitidis* NZ-05/33 (NMBNZ0533_1722), M04-240196 (NMBNZ0533_1722), M01-240149 (NMBH4476_1701), H44/76 (NMBH4476_1701) **hypothetical proteins upstream of transposase gene**	100	GTCTGATTTT
				N. lactamica isolate 3207487 (plasmid pNL3.2), *N. lactamica* (plasmid pNL9)	97	GTCTGATTTT
				N. gonorrhoeae TCDC-NG08107, NCCP11945 **intergenic region (putative phage proteins)**	93	GGCTGTTTTT
				N. gonorrhoeae NCCP11945 (NGK_1948, NGK_1990, NGK_2023) **hypothetical protein genes**	93	GTCTGATTTT
				N. gonorrhoeae **intergenic region PivNG**	93	GTCTGATTTT
				N. gonorrhoeae FA 1090 **numerous intergenic regions in prophages**	93	GTCTGATTTT
		7	AAATTCGTTTCAGATAGCAAACGCAGTAGT	*N. gonorrhoeae* TCDC-NG08107, *N. gonorrhoeae* NCCP11945 **intergenic region (putative phage proteins)**	97	GGACGATTTC
		12	GGGTAGCCAGTGCTAAAACCGCACCCGCTT	*N. lactamica* plasmid pNL9	93	TGCGGGCATA
				N. meningitidis plasmid pJS-B	100	TACGAACATT
		13	CCAAATAGAAATACATACGCCGAGTAATTA	*N. lactamica* plasmid pNL9	93	AGCTGCTTTG
				N. meningitidis plasmid pJS-B	97	AGCCGCTTTG
		14	TTTCTTTTTGTAATTGTTCTGCCTTTTTTA	*N. lactamica* plasmid pNL9	100	ATTGGATTTT

Strain[a]	Number of spacers	CRISPR Spacer[b]	Spacer sequence	Blast candidate[c]	% Identity[d]	10 bp downstream protospacer[e]
				N. meningitidis plasmid pJS-B	100	ATTGGATTTT
		15	TACCCACGGCGGAAACCATTGCCACAAAAC	*N. meningitidis* strain alpha522 draft genome (NMALPHA522_0671), H44/76 (NMBH4476_0684), 053442 (NMCC_0153), *N. meningitidis* serogroup C FAM18 (NMC1864) **hypothetical protein gene**	100	CCATGATTAC
				N.meningitidis M04-240196 (NMBM04240196_0048, NMBM04240196_0749) **putative membrane protein gene**	100	CCATGATTAC
Pasteurella multocida str. Pm70 (Accession: NC_002663)	5	1-5		no significant BLAST hits		
Pasteurella multocida subsp. *gallicida* X73 (Accession: CM001580 AMBP01000000)	20	9	AAAGAATACACCCTTATTCCAAAAAGTTTG	*P. multocida* 1.8 kb plasmid	100	GCGACAGATG
		15	GTCTGAACAGTATTAACACTTCCTGTTTCT	*P. multocida* subsp. *multocida* str. HN06(PMCN06_2098) **hypothetical protein gene**	97	GGATCGTACT
Francisella tularensis subsp. *novicida* U112 (Accession: NC_008601)	13	1-13		no significant BLAST hits		
Francisella novicida FTG FTG_scaffold1 genomic scaffold (Accession: NZ_DS995363 NZ_ABXZ01000000)	22	15	ATCTCAAAAGCAGCTCTTTCGCGTGTAATATCGTT	*F. cf. novicida* 3523 (FN3523_1002) **phage protein gene**	91	TGGATTAGAT
		19	CTATCTAAGAGAACTTACAAGACAAGAGAAAATACT	*F. cf. novicida* 3523 (FN3523_0993) **hypothetical protein gene**	94	TGGTGGTAAA
Francisella tularensis	10	2	AGCCCTATCAGAAATATATGCAAGTTTGAATATAG	*F. cf. novicida* 3523 (FN3523_1009) **phage-related baseplate assembly protein gene**	89	AGGGTGTAGC

Strain[a]	Number of spacers	CRISPR Spacer[b]	Spacer sequence	Blast candidate[c]	% Identity[d]	10 bp downstream protospacer[e]
subsp. *novicida GA99-3548* supercont1.3 (Accession: DS264589 ABAH01000000)		**3**	AGATAACTCTTATATTGATTTGTATATTGAAGATA	*F. cf. novicida* 3523 (FN3523_1006) **hypothetical protein gene**	**94**	TGATTAGCAT
		**4**	CGCAAAAAAAGGCGAATTTGAGCAGAAAATTTGGGC	*F. cf. novicida* 3523 (FN3523_0999) **hypothetical protein gene**	**91**	TGGTATTGAT

[a]Selected strains used in this study. No potential protospacers were found for *Streptococcus mutans* UA159, *Campylobacter jejuni* subsp. *jejuni* NCTC 11168, *Pasteurella multocida* str. Pm70 and *Francisella tularensis* subsp. *novicida* U112. Therefore, closely related strains were analyzed for the presence of type II CRISPR-Cas arrays. Spacer sequences from selected arrays were then used to search for protospacer candidates.

[b]Numbering of spacers starts from the leader proximal end based on RNAseq data (15). Spacers with no significant protospacer BLAST hit are not listed in the table.

[c]A BLAST candidate was considered a potential protospacer when the identity to the spacer was ≥ 90% and when the protospacer originated either from phage, plasmid or genomic DNA related to the analyzed species. For each identified protospacer, the strain name, the protospacer-containing gene locus and the potential function of the gene are given.

[d]Percentage identity between spacer and protospacer sequence.

[e]10 nt sequence located directly 3' of the protospacer sequence. The identified sequences for each bacterial species were aligned using GeneDoc (http://www.nrbsc.org/gfx/genedoc/). The degree of conservation is indicated with a color code (black: 100%, dark grey: ≥ 80%, light grey: ≥ 60%). These sequences were used to create the logo plot represented in Figure 5.

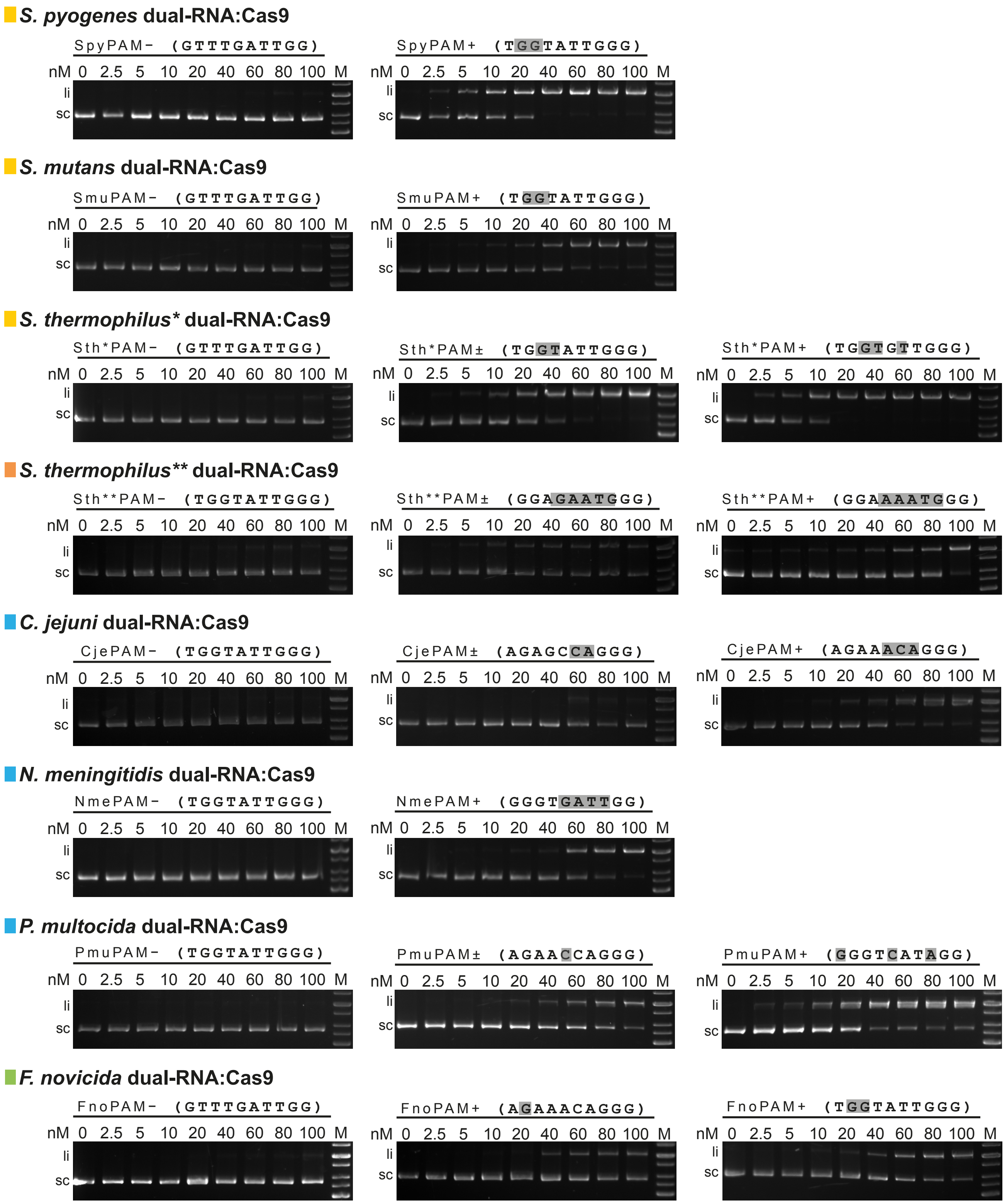

Supplementary Figure S9. Cas9 orthologs cleave plasmid DNA in the presence of their cognate dual-RNA and specific PAM. Agarose gel electrophoresis analysis of dual-RNA:Cas9 titration (0-100 nM dual-RNA-Cas9 complex) on plasmid DNA (5 nM) containing *speM* protospacer and adjacent WT PAM (PAM+), imperfect PAM (PAM±) or no PAM (PAM–). For *S. pyogenes*, *S. mutans*, *S. thermophilus**, *S. thermophilus*** and *N. meningitidis,* the PAM sequence has already been published (27,28,53,54). For the other bacterial species, PAMs were predicted based on the downstream sequence of protospacer identified in the investigated or related strains (see Supplementary Table S2 and Materials and Methods). The 10 bp sequence located directly downstream of the crRNA-targeted *speM* protospacer is shown. The nucleotide(s) predicted to belong to the PAM sequence are shaded in grey. li: linear cleavage product, sc: supercoiled plasmid DNA, M: 1 kb DNA ladder.

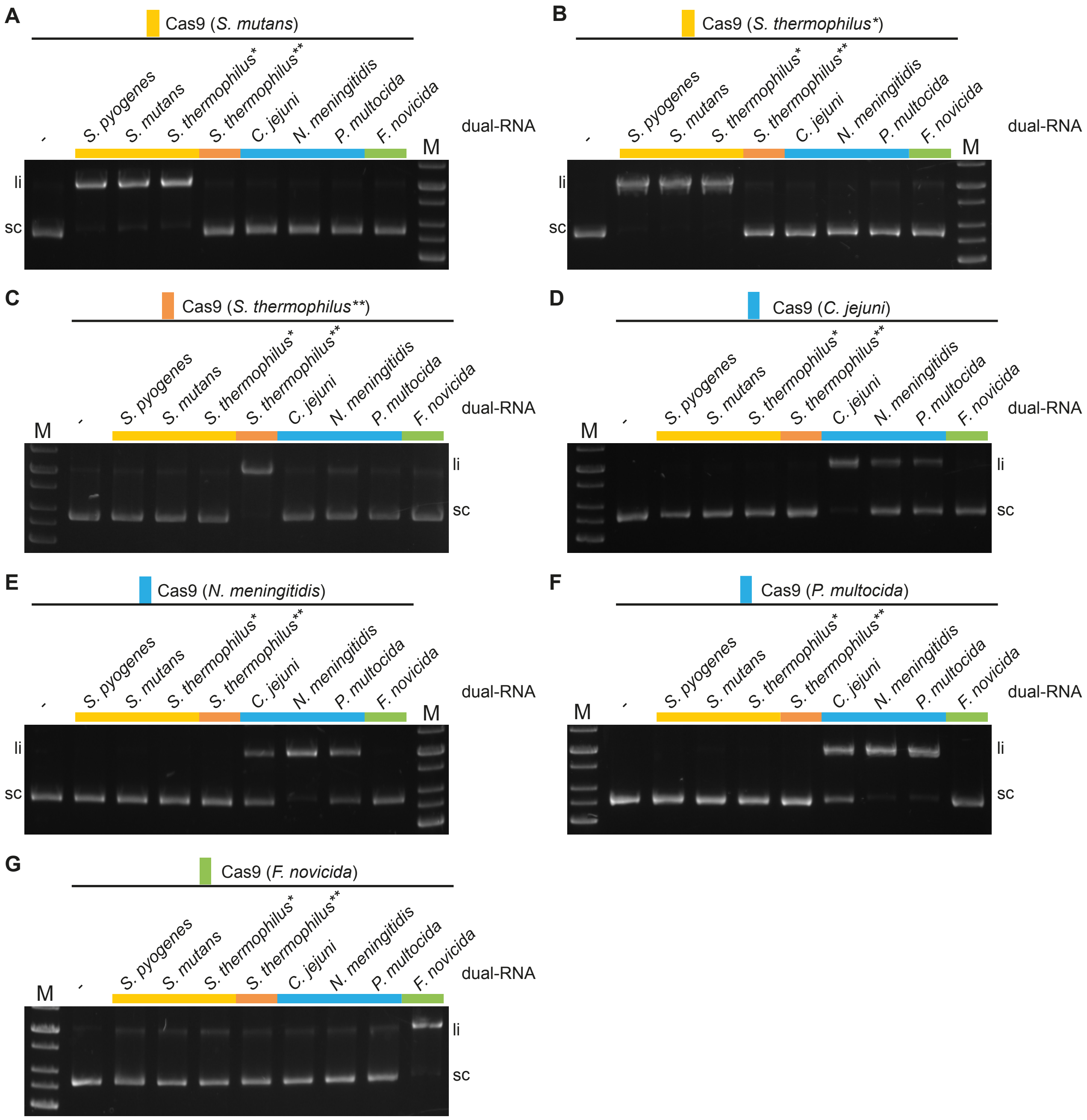

Supplementary Figure S10. Summary of *in vitro* plasmid cleavage assays of Cas9 orthologs in combination with dual-RNAs. Agarose gel electrophoresis of cleavage assays. (**A**) *S. mutans* Cas9 (50 nM), (**B**) *S. thermophilus** Cas9 (25 nM), (**C**) *S. thermophilus*** Cas9 (100 nM), (**D**) *C. jejuni* Cas9 (100 nM), (**E**) *N. meningitidis* Cas9 (100 nM), (**F**) *P. multocida* Cas9 (25 nM), (**G**) *F. novicida* Cas9 (100 nM) in complex with equimolar concentrations of each of the dual-RNA orthologs were incubated with plasmid DNA (5 nM) containing *speM* protospacer sequence and the PAM sequence specific to the Cas9 ortholog analyzed. li: linear cleavage product, sc: supercoiled plasmid DNA, M: 1 kb DNA ladder.

Supplementary Table S4. Cas9 is present in bacteria from 12 different phyla and diverse habitats.

Strain[a]	Class	Isolation/habitat[b]
**Actinobacteria**		
Actinobacteridae		
Acidothermus cellulolyticus 11B	*Acidothermaceae*	extremophile (hot water spring)
Actinomyces coleocanis DSM 15436	*Actinomycetaceae*	dog genital tract
Actinomyces georgiae F0490	*Actinomycetaceae*	oral cavity
Actinomyces naeslundii str. Howell 279	*Actinomycetaceae*	oral cavity
Actinomyces sp. ICM47	*Actinomycetaceae*	ND
Actinomyces sp. oral taxon 175 str. F0384	*Actinomycetaceae*	oral cavity
Actinomyces sp. oral taxon 180 str. F0310	*Actinomycetaceae*	oral cavity
Actinomyces sp. oral taxon 181 str. F0379	*Actinomycetaceae*	oral cavity
Actinomyces sp. oral taxon 848 str. F0332	*Actinomycetaceae*	oral cavity
Actinomyces turicensis ACS-279-V-Col4	*Actinomycetaceae*	genital tract
Bifidobacterium bifidum S17	*Bifidobacteriaceae*	gastrointestinal tract/feces
Bifidobacterium dentium Bd1	*Bifidobacteriaceae*	oral cavity
Bifidobacterium longum DJO10A	*Bifidobacteriaceae*	gastrointestinal tract/feces
Bifidobacterium sp. 12_1_47BFAA	*Bifidobacteriaceae*	gastrointestinal tract/feces
Corynebacterium accolens ATCC 49726	*Corynebacterineae*	wound
Corynebacterium diphteriae NCTC 13129	*Corynebacterineae*	oral cavity
Corynebacterium matruchotii ATCC 14266	*Corynebacterineae*	oral cavity
Gardnerella vaginalis 5-1	*Bifidobacteriaceae*	genital tract
Mobiluncus curtisii ATCC 35242	*Actinomycetaceae*	genital tract
Mobiluncus mulieris 28-1	*Actinomycetaceae*	genital tract
Scardovia inopinata F0304	*Bifidobacteriaceae*	oral cavity
Scardovia wiggsiae F0424	*Bifidobacteriaceae*	oral cavity
Coriobacteridae		
Coriobacterium glomerans PW2	*Coriobacteriaceae*	invertebrate (red soldier bug)
Eggerthella sp. YY7918	*Coriobacteriaceae*	gastrointestinal tract/feces
Gordonibacter pamelaeae 7-10-1-b	*Coriobacteriaceae*	gastrointestinal tract/feces
Olsenella uli DSM 7084	*Coriobacteriaceae*	oral cavity
**Bacteroidetes**		
Bacteroidia		
Anaerophaga sp. HS1	*Marinilabiliaceae*	extremophile (hot water spring)
Anaerophaga thermohalophila DSM 12881	*Marinilabiliaceae*	environmental sample (oil residue)
Bacteroides cellulosilyticus DSM 14838	*Bacteroidaceae*	gastrointestinal tract/feces
Bacteroides coprophilus DSM 18228	*Bacteroidaceae*	gastrointestinal tract/feces
Bacteroides coprosuis DSM 18011	*Bacteroidaceae*	pig feces
Bacteroides dorei DSM 17855	*Bacteroidaceae*	gastrointestinal tract/feces
Bacteroides eggerthii 1_2_48FAA	*Bacteroidaceae*	gastrointestinal tract/feces
Bacteroides faecis MAJ27	*Bacteroidaceae*	gastrointestinal tract/feces
Bacteroides fluxus YIT 12057	*Bacteroidaceae*	gastrointestinal tract/feces
Bacteroides fragilis NCTC9343	*Bacteroidaceae*	gastrointestinal tract/feces
Bacteroides nordii CL02T12C05	*Bacteroidaceae*	gastrointestinal tract/feces
Bacteroides oleiciplenus YIT 12058	*Bacteroidaceae*	gastrointestinal tract/feces
Bacteroides sp. 2_1_16	*Bacteroidaceae*	gastrointestinal tract/feces
Bacteroides sp. 203	*Bacteroidaceae*	gastrointestinal tract/feces
Bacteroides sp. 3_1_19	*Bacteroidaceae*	gastrointestinal tract/feces
Bacteroides sp. 3_1_33FAA	*Bacteroidaceae*	gastrointestinal tract/feces
Bacteroides sp. 9_1_42FAA	*Bacteroidaceae*	gastrointestinal tract/feces
Bacteroides sp. D2	*Bacteroidaceae*	gastrointestinal tract/feces
Bacteroides uniformis CL03T00C23	*Bacteroidaceae*	gastrointestinal tract/feces
Bacteroides vulgatus CL09T03C04	*Bacteroidaceae*	gastrointestinal tract/feces
Bacteroidetes oral taxon 274 str. F0058	*Bacteroidaceae*	oral cavity
Barnesiella intestinihominis YIT 11860	*Bacteroidaceae*	gastrointestinal tract/feces

Strain[a]	Class	Isolation/habitat[b]
**Bacteroidia (continued)**		
Marinilabilia sp. AK2	*Marinilabiliaceae*	extremophile (solar saltern)
Odoribacter laneus YIT 12061	*Porphyromonadaceae*	gastrointestinal tract/feces
Parabacteroides johnsonii DSM 18315	*Bacteroidaceae*	gastrointestinal tract/feces
Parabacteroides sp. D13	*Bacteroidaceae*	gastrointestinal tract/feces
Porphyromonas catoniae F0037	*Porphyromonadaceae*	oral cavity
Porphyromonas sp. oral taxon 279 str. F0450	*Porphyromonadaceae*	oral cavity
Prevotella bivia JCVIHMP010	*Prevotellaceae*	genital tract
Prevotella buccae ATCC 33574	*Prevotellaceae*	oral cavity
Prevotella buccalis ATCC 35310	*Prevotellaceae*	oral cavity
Prevotella denticola F0289	*Prevotellaceae*	oral cavity
Prevotella disiens FB035-09AN	*Prevotellaceae*	oral cavity
Prevotella histicola F0411	*Prevotellaceae*	oral cavity
Prevotella intermedia 17	*Prevotellaceae*	oral cavity
Prevotella melaninogenica D18	*Prevotellaceae*	oral cavity/rumen
Prevotella micans F0438	*Prevotellaceae*	oral cavity
Prevotella multiformis DSM 16608	*Prevotellaceae*	oral cavity
Prevotella nigrescens ATCC 33563	*Prevotellaceae*	oral cavity
Prevotella oralis ATCC 33269	*Prevotellaceae*	oral cavity
Prevotella oulorum F0390	*Prevotellaceae*	oral cavity
Prevotella ruminicola 23	*Prevotellaceae*	rumen
Prevotella saccharolytica F0055	*Prevotellaceae*	oral cavity
Prevotella sp. C561	*Prevotellaceae*	oral cavity
Prevotella sp. MSX73	*Prevotellaceae*	oral cavity
Prevotella sp. oral taxon 306 str. F0472	*Prevotellaceae*	oral cavity
Prevotella sp. oral taxon 317 str. F0108	*Prevotellaceae*	oral cavity
Prevotella sp. oral taxon 472 str. F0295	*Prevotellaceae*	oral cavity
Prevotella stercorea DSM 18206	*Prevotellaceae*	gastrointestinal tract/feces
Prevotella tannerae ATCC 51259	*Prevotellaceae*	oral cavity
Prevotella timonensis CRIS 5C-B1	*Prevotellaceae*	wound (breast abscess)
Prevotella veroralis F0319	*Prevotellaceae*	oral cavity
Tannerella sp. 6_1_58FAA_CT1	*Porphyromonadaceae*	gastrointestinal tract/feces
**Cytophagia**		
Belliella baltica DSM 15883	*Cyclobacteriaceae*	environmental sample (groundwater)
Indibacter alkaliphilus LW1	*Cyclobacteriaceae*	extremophile (soda lake)
Nitritalea halalkaliphila LW7	*Cyclobacteriaceae*	extremophile (saline soda lake)
**Flavobacteria**		
Bergeyella zoohelcum ATCC 43767	*Flavobacteriaceae*	oral cavity
Capnocytophaga canimorsus Cc5	*Flavobacteriaceae*	dog and cat oral cavity/zoonotic infections
Capnocytophaga gingivalis ATCC 33624	*Flavobacteriaceae*	oral cavity
Capnocytophaga ochracea DSM 7271	*Flavobacteriaceae*	oral cavity
Capnocytophaga sp. CM59	*Flavobacteriaceae*	oral cavity
Capnocytophaga sp. oral taxon 324 str. F0483	*Flavobacteriaceae*	oral cavity
Capnocytophaga sp. oral taxon 326 str. F0382	*Flavobacteriaceae*	oral cavity
Capnocytophaga sp. oral taxon 329 str. F0087	*Flavobacteriaceae*	oral cavity
Capnocytophaga sp. oral taxon 335 str. F0486	*Flavobacteriaceae*	oral cavity
Capnocytophaga sp. oral taxon 380 str. F0488	*Flavobacteriaceae*	oral cavity
Capnocytophaga sp. oral taxon 412 str. F0487	*Flavobacteriaceae*	oral cavity
Capnocytophaga sputigena ATCC 33612	*Flavobacteriaceae*	oral cavity
Chryseobacterium sp. CF314	*Flavobacteriaceae*	vegetation
Flavobacteriaceae bacterium S85	*Flavobacteriaceae*	environmental sample (seawater)
Flavobacterium branchiophilum FL-15	*Flavobacteriaceae*	fish pathogen
Flavobacterium columnare ATCC 49512	*Flavobacteriaceae*	fish pathogen
Flavobacterium psychrophilum JIP02/86	*Flavobacteriaceae*	fish pathogen
Fluviicola taffensis DSM 16823	*Cryomorphaceae*	environmental sample (fresh water)
Galbibacter sp. ck-I2-15	*Flavobacteriaceae*	extremophile (deep sea sediment)
Joostella marina DSM 19592	*Flavobacteriaceae*	environmental sample (seawater)
Kordia algicida OT-1	*Flavobacteriaceae*	environmental sample (seawater)
Myroides injenensis M09-0166	*Flavobacteriaceae*	human clinical specimens
Myroides odoratus DSM 2801	*Flavobacteriaceae*	fish

Strain[a]	Class	Isolation/habitat[b]
**Flavobacteria (continued)**		
Ornithobacterium rhinotracheale DSM 15997	*Flavobacteriaceae*	bird respiratory tract
Psychroflexus torquis ATCC 700755	*Flavobacteriaceae*	extremophile (antarctic ice)
Riemerella anatipestifer ATCC 11845 = DSM 15868	*Flavobacteriaceae*	bird
Weeksella virosa DSM 16922	*Flavobacteriaceae*	genital tract/urine
Zunongwangia profunda SM-A87	*Flavobacteriaceae*	extremophile (deep sea sediment)
**Sphingobacteria**		
Mucilaginibacter paludis DSM 18603	*Sphingobacteriaceae*	food (fermented)
Niabella soli DSM 19437	*Chitinophagaceae*	environmental sample (soil)
Sphingobacterium spiritivorum ATCC 33861	*Sphingobacteriaceae*	human clinical specimens
**Firmicutes**		
**Bacilli**		
Alicycliphilus denitrificans K601	*Alicyclobacillaceae*	environmental sample (sewage)
Alicyclobacillus hesperidum URH17-3-68	*Alicyclobacillaceae*	extremophile (hot water spring)
Bacillus cereus Rock1-15	*Bacillaceae*	environmental sample (soil)
Bacillus smithii 7 3 47FAA	*Bacillaceae*	human clinical specimens
Bacillus thuringiensis serovar *finitimus* YBT-020	*Bacillaceae*	environmental sample (soil)
Brevibacillus laterosporus GI-9	*Paenibacillaceae*	environmental sample (soil)
Catellicoccus marimammalium M35/04/3	*Enterococcaceae*	grey seal gastrointestinal tract
Dolosigranulum pigrum ATCC 51524	*Carnobacteriaceae*	human clinical specimens
Enterococcus faecalis TX0012	*Enterococcaceae*	gastrointestinal tract/feces
Enterococcus faecium 1231408	*Enterococcaceae*	gastrointestinal tract/feces
Enterococcus hirae ATCC 9790	*Enterococcaceae*	gastrointestinal tract/feces
Enterococcus italicus DSM 15952	*Enterococcaceae*	food (fermented)
Enterococcus sp. 7L76	*Enterococcaceae*	gastrointestinal tract/feces
Facklamia hominis CCUG 36813	*Aerococcaceae*	burbuncle (human)
Fructobacillus fructosus KCTC 3544	*Leuconostocaceae*	vegetation
Gemella haemolysans ATCC 10379	*Streptococcaceae*	oral cavity
Gemella moribillum M424	*Streptococcaceae*	gastrointestinal tract/feces
Lactobacillus animalis KCTC 3501	*Lactobacillaceae*	food (fermented)
Lactobacillus brevis subsp. *gravesensis* ATCC 27305	*Lactobacillaceae*	food (fermented)
Lactobacillus buchneri ATCC 11577	*Lactobacillaceae*	food (fermented)
Lactobacillus casei str. Zhang	*Lactobacillaceae*	gastrointestinal tract/feces
Lactobacillus coryniformis subsp. *coryniformis* KCTC 3167	*Lactobacillaceae*	food (fermented)
Lactobacillus coryniformis subsp. *torquens* KCTC 3535	*Lactobacillaceae*	food (fermented)
Lactobacillus crispatus FB049-03	*Lactobacillaceae*	genital tract
Lactobacillus curvatus CRL 705	*Lactobacillaceae*	food (fermented)
Lactobacillus delbrueckii subsp. *bulgaricus* 2038	*Lactobacillaceae*	food (fermented)
Lactobacillus farciminis KCTC 3681	*Lactobacillaceae*	food (fermented)
Lactobacillus fermentum ATCC 14931	*Lactobacillaceae*	food (fermented)
Lactobacillus florum 2F	*Lactobacillaceae*	vegetation
Lactobacillus gasseri JV-V03	*Lactobacillaceae*	oral cavity
Lactobacillus hominis CRBIP 24.179	*Lactobacillaceae*	gastrointestinal tract/feces
Lactobacillus iners LactinV 11V1-d	*Lactobacillaceae*	genital tract/urine
Lactobacillus jensenii 269-3	*Lactobacillaceae*	genital tract/blood
Lactobacillus johnsonii DPC 6026	*Lactobacillaceae*	pig gastrointestinal tract
Lactobacillus mucosae LM1	*Lactobacillaceae*	wild pig gastrointestinal tract
Lactobacillus paracasei subsp. *paracasei* 8700:2	*Lactobacillaceae*	food (fermented)
Lactobacillus pentosus IG1	*Lactobacillaceae*	food (fermented)
Lactobacillus plantarum ZJ316	*Lactobacillaceae*	gastrointestinal tract/feces
Lactobacillus rhamnosus GG	*Lactobacillaceae*	gastrointestinal tract/feces
Lactobacillus ruminis ATCC 25644	*Lactobacillaceae*	rumen
Lactobacillus salivarius UCC118	*Lactobacillaceae*	oral cavity
Lactobacillus sanfranciscensis TMW 1-1304	*Lactobacillaceae*	food (fermented)
Lactobacillus sp. 66c	*Lactobacillaceae*	ND
Lactobacillus versmoldensis KCTC 3814	*Lactobacillaceae*	food (fermented)
Leuconostoc gelidum KCTC 3527	*Leuconostocaceae*	food (fermented)
Leuconostoc pseudomesenteroides 4882	*Leuconostocaceae*	food (fermented)

Strain[a]	Class	Isolation/habitat[b]
	Bacilli (continued)	
Listeria innocua Clip11262	*Listeriaceae*	environmental sample (soil)
Listeria ivanovii FSL F6-596	*Listeriaceae*	animal and human/environmental samples
Listeria monocytogenes str. 1/2a F6854	*Listeriaceae*	animal and human/environmental samples
Listeria seeligeri FSL N1-067	*Listeriaceae*	animal and human/environmental samples
Listeriaceae bacterium TTU M1-001	*Listeriaceae*	environmental sample (soil)
Oenococcus kitaharae DSM 17330	*Leuconostocaceae*	food (fermented)
Pediococcus acidilactici DSM 20284	*Lactobacillaceae*	vegetation
Pediococcus lolii NGRI 0510Q	*Lactobacillaceae*	vegetation (fermented)
Planococcus antarcticus DSM 14505	*Planococcaceae*	extremophile (antarctic)
Sporolactobacillus vineae DSM 21990 = SL153	*Sporolactobacillaceae*	environmental sample (soil)
Staphylococcus aureus subsp. *aureus*	*Staphylococcaceeae*	human clinical specimens
Staphylococcus lugdunensis M23590	*Staphylococcaceeae*	human clinical specimens
Staphylococcus massiliensis S46	*Staphylococcaceeae*	skin
Staphylococcus pseudintermedius ED99	*Staphylococcaceeae*	dog skin
Staphylococcus simulans ACS-120-V-Sch1	*Staphylococcaceeae*	genital tract
Streptococcus agalactiae 2603V/R	*Streptococcaceae*	gastrointestinal tract/feces
Streptococcus anginosus F0211	*Streptococcaceae*	oral cavity
Streptococcus bovis ATCC 700338	*Streptococcaceae*	rumen/zoonotic infections
Streptococcus canis FSL Z3-227	*Streptococcaceae*	food (fermented)
Streptococcus constellatus subsp. *constellatus* SK53	*Streptococcaceae*	human clinical specimens
Streptococcus downei F0415	*Streptococcaceae*	monkey oral cavity
Streptococcus dysgalactiae DSM 12112	*Streptococcaceae*	various animals/zoonotic infections
Streptococcus equi subsp. *zooepidemicus* MGCS10565	*Streptococcaceae*	horse respiratory tract
Streptococcus equinus ATCC 9812	*Streptococcaceae*	ruminants alimentary tract
Streptococcus gallolyticus UCN34	*Streptococcaceae*	ruminants alimentary tract
Streptococcus gordonii str. Challis substr. CH1	*Streptococcaceae*	oral cavity
Streptococcus infantarius ATCC BAA-102	*Streptococcaceae*	gastrointestinal tract/feces
Streptococcus iniae 9117	*Streptococcaceae*	fish/human pathogen
Streptococcus macacae NCTC 11558	*Streptococcaceae*	monkey oral cavity
Streptococcus macedonicus ACA-DC 198	*Streptococcaceae*	food (fermented)
Streptococcus mitis ATCC 6249	*Streptococcaceae*	oral cavity
Streptococcus mutans UA159	*Streptococcaceae*	oral cavity
Streptococcus oralis SK1074	*Streptococcaceae*	oral cavity
Streptococcus parasanguinis F0449	*Streptococcaceae*	oral cavity
Streptococcus pasteurianus ATCC 43144	*Streptococcaceae*	blood
Streptococcus pseudoporcinus SPIN 20026	*Streptococcaceae*	genital tract
Streptococcus pyogenes SF370	*Streptococcaceae*	oral cavity/wounds
Streptococcus ratti FA-1 = DSM 20564	*Streptococcaceae*	rat oral cavity
Streptococcus salivarius JIM8777	*Streptococcaceae*	oral cavity
Streptococcus sanguinis VMC66	*Streptococcaceae*	oral cavity
Streptococcus sp. BS35b	*Streptococcaceae*	oral cavity
Streptococcus sp. C150	*Streptococcaceae*	oral cavity (expectorated sputum)
Streptococcus sp. C300	*Streptococcaceae*	oral cavity (expectorated sputum)
Streptococcus sp. F0441	*Streptococcaceae*	oral cavity
Streptococcus sp. GMD4S	*Streptococcaceae*	oral cavity
Streptococcus sp. GMD6S	*Streptococcaceae*	oral cavity
Streptococcus sp. M334	*Streptococcaceae*	oral cavity (expectorated sputum)
Streptococcus sp. oral taxon 056 str. F0418	*Streptococcaceae*	oral cavity
Streptococcus sp. oral taxon 071 str. 73H25AP	*Streptococcaceae*	oral cavity
Streptococcus suis 89/1591	*Streptococcaceae*	pig
Streptococcus thermophilus LMD-9	*Streptococcaceae*	food (fermented)
Streptococcus vestibularis ATCC 49124	*Streptococcaceae*	oral cavity
	Clostridia	
Acidaminococcus intestini RyC-MR95	*Acidaminococcaceae*	wound/abscess
Acidaminococcus sp. D21	*Acidaminococcaceae*	gastrointestinal tract/feces
Aminomonas paucivorans DSM 12260	*Syntrophoomonadaceae*	environmental sample (sewage)
Anaerococcus tetradius ATCC 35098	*Peptostreptococcaceae*	human clinical specimens
Butyrivibrio fibrisolvens 16/4	*Lachnospiraceae*	rumen
Catenibacterium mitsuokai DSM 15897	*Lachnospiraceae*	gastrointestinal tract/feces
Clostridium cellulolyticum H10	*Clostridiaceae*	vegetation (composted)

Strain[a]	Class	Isolation/habitat[b]
	**Clostridia (continued)**	
Clostridium perfringens D str. JGS1721	*Clostridiaceae*	environmental sample (vegetation/marine sediment)
Clostridium spiroforme DSM 1552	*Clostridiaceae*	gastrointestinal tract/feces
Coprococcus catus GD/7	*Lachnospiraceae*	gastrointestinal tract/feces
Coprococcus comes ATCC 27758	*Lachnospiraceae*	gastrointestinal tract/feces
Dorea longicatena DSM 13814	*Clostridiaceae*	gastrointestinal tract/feces
Eubacterium dolichum DSM 3991	*Eubacteriaceae*	gastrointestinal tract/feces
Eubacterium rectale ATCC 33656	*Eubacteriaceae*	gastrointestinal tract/feces
Eubacterium sp. AS15	*Eubacteriaceae*	oral cavity
Eubacterium ventriosum ATCC 27560	*Eubacteriaceae*	gastrointestinal tract/feces
Eubacterium yurii subsp. margaretiae ATCC 43715	*Peptostreptococcaceae*	oral cavity
Filifactor alocis ATCC 35896	*Peptostreptococcaceae*	cat and human oral cavity
Finegoldia magna ATCC 29328	*Peptostreptococcaceae*	oral cavity
Helcococcus kunzii ATCC 51366	*Clostridiales Family XI*	wound
Oribacterium sinus F0268	*Lachnospiraceae*	human clinical specimens
Peptoniphilus duerdenii ATCC BAA-1640	*Peptostreptococcaceae*	wound
Peptoniphilus sp. oral taxon 386 str. F0131	*Peptostreptococcaceae*	oral cavity
Phascolarctobacterium sp. YIT 12067	*Acidaminococcaceae*	gastrointestinal tract/feces
Phascolarctobacterium succinatutens YIT 12067	*Acidaminococcaceae*	gastrointestinal tract/feces
Pseudoramibacter alactolyticus ATCC 23263	*Clostridiaceae*	oral cavity
Roseburia intestinalis L1-82	*Lachnospiraceae*	gastrointestinal tract/feces
Roseburia inulinivorans DSM 16841	*Lachnospiraceae*	gastrointestinal tract/feces
Ruminococcus albus 8	*Ruminococcaceae*	gastrointestinal tract/feces
Ruminococcus lactaris ATCC 29176	*Ruminococcaceae*	gastrointestinal tract/feces
Subdoligranulum sp. 4_3_54A2FAA	*Ruminococcaceae*	gastrointestinal tract/feces
	**Negativicutes**	
Megasphaera sp. UPII 135-E	*Veillonellaceae*	rumen
Veillonella atypica ACS-134-V-Col7a	*Veillonellaceae*	oral cavity
Veillonella parvula ATCC17745	*Veillonellaceae*	gastrointestinal/genital tract
Veillonella sp. 6_1_27	*Veillonellaceae*	gastrointestinal tract/feces
Veillonella sp. oral taxon 780 str. F0422	*Veillonellaceae*	oral cavity
	**Proteobacteria**	
	**Alphaproteobacteria**	
Acetobacter aceti NBRC 14818	*Acetobacteraceae*	environmental sample
Azospirillum sp. B510	*Rhodospirillaceae*	vegetation
Bradyrhizobium sp. BTAi1	*Bradyrhizobiaceae*	vegetation
Caenispirillum salinarum AK4	*Rhodospirillaceae*	extremophile (solar saltern)
Dinoroseobacter shibae DFL 12	*Rhodobacteraceae*	environmental sample (seawater)
Gluconacetobacter diazotrophicus PAl5	*Acetobacteriaceae*	vegetation
Maritimibacter alkaliphilus ATCC2654	*Rhodobacteraceae*	environmental sample (seawater)
Methylocystis sp. ATCC 49242	*Methylocystaceae*	environmental sample (sewage, fresh water)
Methylosinus trichosporium OB3b	*Methylocystaceae*	environmental sample (soil, fresh water)
Nitrobacter hamburgensis X14	*Bradyrhizobiaceae*	environmental sample (soil)
Parvibaculum lavamentivorans DS-1	*Phyllobacteriaceae*	environmental sample (sewage)
Puniceispirillum marinum IMCC1322	*SAR16 clade*	environmental sample (seawater)
Rhodopseudomonas palustris BisB18	*Bradyrhizobiaceae*	environmental sample (soil)
Rhodospirillum rubrum ATCC 11170	*Rhodospirillaceae*	environmental sample (sea mud)
Rhodovulum sp. PH10	*Rhodobacteraceae*	environmental sample (soil)
Sphingobium sp. AP49	*Sphingomonadaceae*	vegetation
Sphingomonas sp. S17	*Sphingomonadaceae*	environmental sample (stromatolite)
Tistrella mobilis KA081020-065	*Rhodospirillaceae*	environmental sample (seawater)
	**Betaproteobacteria**	
Acidovorax avenae subsp. *avenae* ATCC 19860	*Comamonadaceae*	environmental sample (soil)
Acidovorax ebreus TPSY	*Comamonadaceae*	environmental sample (water)
Burkholderiales bacterium 1 1 47	*Burkholderiales*	gastrointestinal tract/feces
Kingella kingae ATCC 23330	*Neisseriaceae*	oral cavity

Strain[a]	Class	Isolation/habitat[b]
**Betaproteobacteria (continued)**		
Neisseria bacilliformis ATCC BAA-1200	*Neisseriaceae*	oral cavity
Neisseria cinerea ATCC 14685	*Neisseriaceae*	oral cavity
Neisseria flavescens SK114	*Neisseriaceae*	human clinical specimens
Neisseria lactamica 020-06	*Neisseriaceae*	oral cavity
Neisseria meningitides Z2491	*Neisseriaceae*	oral cavity
Neisseria mucosa C102	*Neisseriaceae*	oral cavity (expectorated sputum)
Neisseria sp. oral taxon 014 str. F0314	*Neisseriaceae*	oral cavity
Neisseria subflava NJ9703	*Neisseriaceae*	oral cavity
Neisseria wadsworthii 9715	*Neisseriaceae*	skin
Nitrosomonas sp. AL212	*Nitrosomonadaceae*	environmental sample (fresh water)
Parasutterella excrementihominis YIT 11859	*Alcaligenaceae*	gastrointestinal tract/feces
Ralstonia syzygii R24	*Burkholderiaceae*	environmental sample (soil)
Simonsiella muelleri ATCC 29453	*Neisseriaceae*	oral cavity
Sutterella parvirubra YIT 11816	*Alcaligenaceae*	gastrointestinal tract/feces
Sutterella wadsworthensis 3 1 45B	*Alcaligenaceae*	gastrointestinal tract/feces
Verminephrobacter aporrectodeae subsp. *tuberculatae* At4	*Comamonadaceae*	invertebrate (earthworm)
Verminephrobacter eiseniae EF01-2	*Comamonadaceae*	invertebrate (earthworm)
**Gammaproteobacteria**		
Actinobacillus minor NM305	*Pasteurellaceae*	pig respiratory tract
Actinobacillus pleuropneumoniae serovar 10 D13039	*Pasteurellaceae*	pig respiratory tract
Actinobacillus succinogenes 130Z	*Pasteurellaceae*	rumen
Actinobacillus suis H91-0380	*Pasteurellaceae*	pig pathogen
Actinobacillus ureae ATCC 25976	*Pasteurellaceae*	respiratory tract
Alcanivorax sp. W11-5	*Alcanivoracaceae*	extremophile (deep sea sediment)
Francisella tularensis subsp. *holarctica* LVS	*Francisellaceae*	engineered live vaccine strain
Francisella tularensis subsp. *novicida* U112	*Francisellaceae*	human/environmental sample (water)
Francisella tularensis subsp. *tularensis* WY96-3418	*Francisellaceae*	wound
gamma proteobacterium HTCC5015	*Unclassified*	environmental sample (seawater)
gammaproteobacterium HdN1	*Unclassified*	environmental sample (sewage)
Haemophilus parainfluenzae T3T1	*Pasteurellaceae*	oral cavity/genital tract
Haemophilus pittmaniae HK 85	*Pasteurellaceae*	oral cavity
Haemophilus sputorum HK 2154	*Pasteurellaceae*	oral cavity
Legionella pneumophila str. Paris	*Legionellaceae*	human clinical specimens
Pasteurella bettyae CCUG 2042	*Pasteurellaceae*	genital tract
Pasteurella multocida subsp. *gallicida* X73	*Pasteurellaceae*	bird pathogen
Pasteurella multocida Pm70	*Pasteurellaceae*	bird respiratory tract/zoonotic infections
**Deltaproteobacteria**		
uncultured delta proteobacterium HF0070_07E19	*Unclassified*	environmental sample (seawater)
**Epsilonproteobacteria**		
Campylobacter coli 2962	*Campylobacteraceae*	animals/human pathogen
Campylobacter jejuni NCTC11168	*Campylobacteraceae*	bird
Campylobacter jejuni subsp. *doylei* 269-97	*Campylobacteraceae*	blood
Campylobacter lari	*Campylobacteraceae*	gastrointestinal tract/feces
Helicobacter canadensis MIT 98-5491	*Helicobacteriaceae*	gastrointestinal tract/feces
Helicobacter cinaedi CCUG 18818	*Helicobacteriaceae*	gastrointestinal tract/feces
Helicobacter hepaticus ATCC 51449	*Helicobacteriaceae*	mouse liver
Helicobacter mustelae 12198	*Helicobacteriaceae*	ferret
Helicobacter pullorum MIT 98-5489	*Helicobacteriaceae*	bird/zoonotic infections
Nitratifractor salsuginis DSM 16511	*Unclassified*	extremophile (deep sea sediment)
Wolinella succinogenes DSM 1740	*Helicobacteraceae*	rumen
**Fusobacteria**		
Fusobacterium nucleatum subsp. *vincentii* ATCC 49256	*Fusobacteriaceae*	oral cavity
Fusobacterium sp. 1_1_41FAA	*Fusobacteriaceae*	gastrointestinal tract/feces
Fusobacterium sp. 3_1_27	*Fusobacteriaceae*	gastrointestinal tract/feces
Fusobacterium sp. 3_1_36A2	*Fusobacteriaceae*	gastrointestinal tract/feces
Ilyobacter polytropus DSM 2926	*Fusobacteriaceae*	environmental sample (sea mud)

Strain[a]	Class	Isolation/habitat[b]
**Fusobacteria** (continued)		
Streptobacillus moniliformis DSM 12112	*Leptotrichiaceae*	rodent/human pathogen
**Spirochaetes**		
Leptospira inadai serovar Lyme str. 10	*Leptospiraceae*	human clinical specimens
Sphaerochaeta globus str. Buddy	*Spirochaetaceae*	extremophile (marine hot spring)
Treponema denticola ATCC 35405	*Spirochaetaceae*	oral cavity
Treponema phagedenis F0421	*Spirochaetaceae*	monkey genital tracts
Treponema sp. JC4	*Spirochaetaceae*	rumen
Treponema vincentii ATCC 35580	*Spirochaetaceae*	oral cavity
**Tenericutes**		
Mollicutes		
Mycoplasma canis PG 14	*Mycoplasmataceae*	dog oral cavity
Mycoplasma cynos C142	*Mycoplasmataceae*	dog respiratory tract
Mycoplasma gallisepticum str. F	*Mycoplasmataceae*	bord pathogen
Mycoplasma iowae 695	*Mycoplasmataceae*	bird
Mycoplasma mobile 163K	*Mycoplasmataceae*	fish pathogen
Mycoplasma ovipneumoniae SC01	*Mycoplasmataceae*	goat respiratory tract
Mycoplasma synoviae 53	*Mycoplasmataceae*	bird pathogen
Solobacterium moorei F0204	*Erysipelotrichaceae*	gastrointestinal tract/feces
**Elusimicrobia**		
Elusimicrobium minutum Pei191	*Elusimicrobiaceae*	invertebrate (scarab beetle)
Uncultured Termite group 1 bacterium phylotype Rs-D17	*Elusimicrobiaceae*	invertebrate
**Fibrobacteres**		
Fibrobacter succinogenes S85	*Fibrobacteraceae*	rumen
**Ignavibacteria**		
Ignavibacterium album JCM 16511	*Ignavibacteriaceae*	extremophile (hot water spring)
**Planktomycetes**		
Blastopirellula marina DSM 3645	*Planctomycetaceae*	environmental sample (seawater)
**Verrucomicrobia**		
Diplosphaera colitermitum TAV2	*Opitutaceae*	invertebrate (termite)
Akkermansia muciniphila ATCC BAA-835	*Verrucomicrobiaceae*	gastrointestinal tract/feces
**Unclassified**		
candidate division TM7 single-cell isolate TM7c	*Unclassified*	oral cavity
uncultured bacterium	*Unclassified*	environmental sample (groundwater)
uncultured bacterium	*Unclassified*	environmental sample (groundwater)
uncultured bacterium T3_7_42578	*Unclassified*	invertebrate (honeybee)

[a]Single strains representing every species found to harbor the *cas9* gene are listed.
[b]The origin of the specific strain and/or typical habitat of the species are given for every strain. ND, no data available. Note that if not specified otherwise, isolates from body sites and feces are human commensals and pathogens.

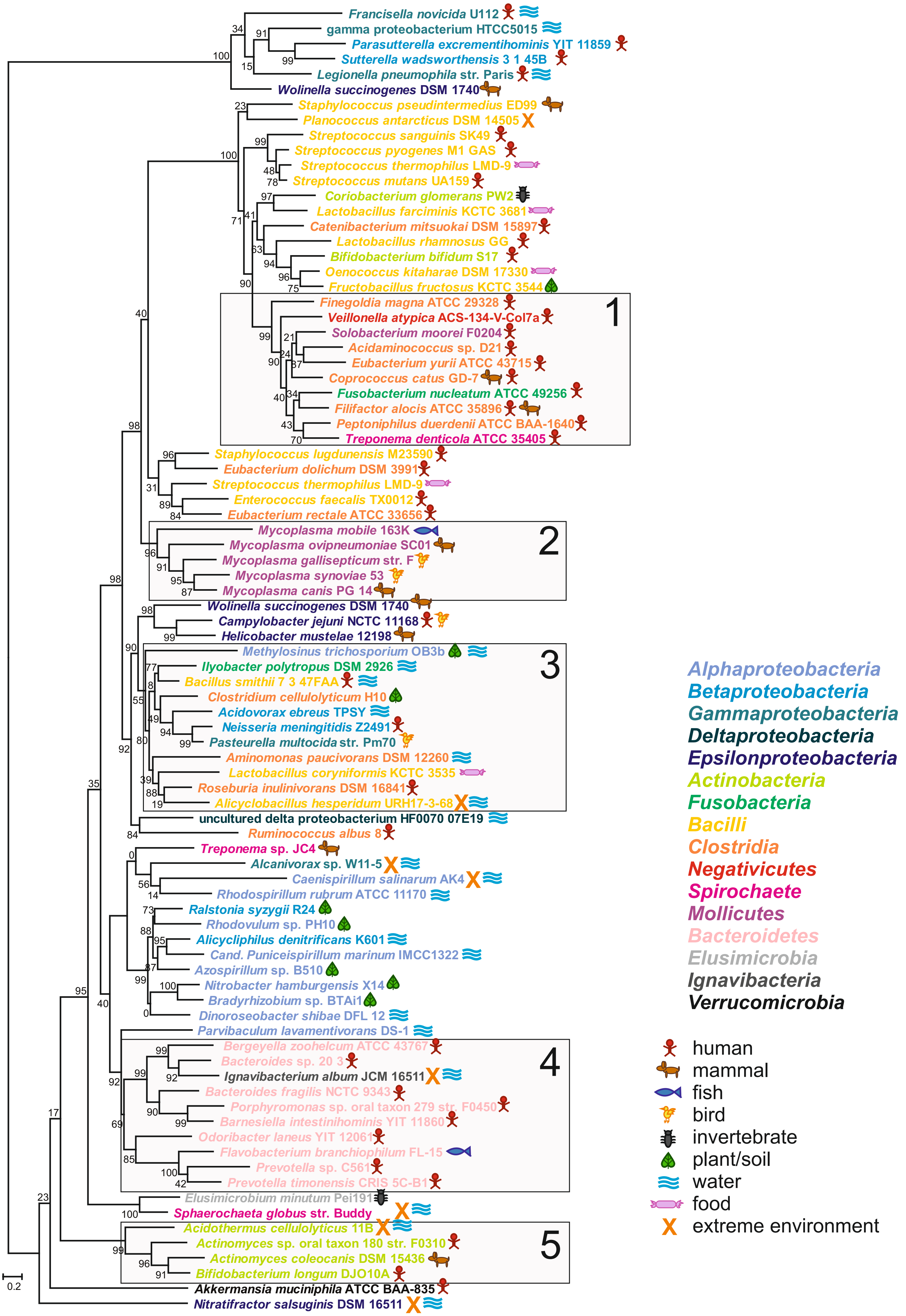
Francisella novicida U112
gamma proteobacterium HTCC5015
Parasutterella excrementihominis YIT 11859
Sutterella wadsworthensis 3 1 45B
Legionella pneumophila str. Paris
Wolinella succinogenes DSM 1740
Staphylococcus pseudintermedius ED99
Planococcus antarcticus DSM 14505
Streptococcus sanguinis SK49
Streptococcus pyogenes M1 GAS
Streptococcus thermophilus LMD-9
Streptococcus mutans UA159
Coriobacterium glomerans PW2
Lactobacillus farciminis KCTC 3681
Catenibacterium mitsuokai DSM 15897
Lactobacillus rhamnosus GG
Bifidobacterium bifidum S17
Oenococcus kitaharae DSM 17330
Fructobacillus fructosus KCTC 3544
1
Finegoldia magna ATCC 29328
Veillonella atypica ACS-134-V-Col7a
Solobacterium moorei F0204
Acidaminococcus sp. D21
Eubacterium yurii ATCC 43715
Coprococcus catus GD-7
Fusobacterium nucleatum ATCC 49256
Filifactor alocis ATCC 35896
Peptoniphilus duerdenii ATCC BAA-1640
Treponema denticola ATCC 35405
Staphylococcus lugdunensis M23590
Eubacterium dolichum DSM 3991
Streptococcus thermophilus LMD-9
Enterococcus faecalis TX0012
Eubacterium rectale ATCC 33656
2
Mycoplasma mobile 163K
Mycoplasma ovipneumoniae SC01
Mycoplasma gallisepticum str. F
Mycoplasma synoviae 53
Mycoplasma canis PG 14
Wolinella succinogenes DSM 1740
Campylobacter jejuni NCTC 11168
Helicobacter mustelae 12198
3
Methylosinus trichosporium OB3b
Ilyobacter polytropus DSM 2926
Bacillus smithii 7 3 47FAA
Clostridium cellulolyticum H10
Acidovorax ebreus TPSY
Neisseria meningitidis Z2491
Pasteurella multocida str. Pm70
Aminomonas paucivorans DSM 12260
Lactobacillus coryniformis KCTC 3535
Roseburia inulinivorans DSM 16841
Alicyclobacillus hesperidum URH17-3-68
uncultured delta proteobacterium HF0070 07E19
Ruminococcus albus 8
Treponema sp. JC4
Alcanivorax sp. W11-5
Caenispirillum salinarum AK4
Rhodospirillum rubrum ATCC 11170
Ralstonia syzygii R24
Rhodovulum sp. PH10
Alicycliphilus denitrificans K601
Cand. Puniceispirillum marinum IMCC1322
Azospirillum sp. B510
Nitrobacter hamburgensis X14
Bradyrhizobium sp. BTAi1
Dinoroseobacter shibae DFL 12
Parvibaculum lavamentivorans DS-1
4
Bergeyella zoohelcum ATCC 43767
Bacteroides sp. 20 3
Ignavibacterium album JCM 16511
Bacteroides fragilis NCTC 9343
Porphyromonas sp. oral taxon 279 str. F0450
Barnesiella intestinihominis YIT 11860
Odoribacter laneus YIT 12061
Flavobacterium branchiophilum FL-15
Prevotella sp. C561
Prevotella timonensis CRIS 5C-B1
Elusimicrobium minutum Pei191
Sphaerochaeta globus str. Buddy
5
Acidothermus cellulolyticus 11B
Actinomyces sp. oral taxon 180 str. F0310
Actinomyces coleocanis DSM 15436
Bifidobacterium longum DJO10A
Akkermansia muciniphila ATCC BAA-835
Nitratifractor salsuginis DSM 16511
0.2
Alphaproteobacteria
Betaproteobacteria
Gammaproteobacteria
Deltaproteobacteria
Epsilonproteobacteria
Actinobacteria
Fusobacteria
Bacilli
Clostridia
Negativicutes
Spirochaete
Mollicutes
Bacteroidetes
Elusimicrobia
Ignavibacteria
Verrucomicrobia
human
mammal
fish
bird
invertebrate
plant/soil
water
food
extreme environment

Supplementary Figure S11. Cas9 tree topology suggests both horizontal and vertical transfer of type II CRISPR-Cas systems. See Figure 1, Supplementary Figure S4 and Supplementary Table S4. The codes for taxonomy (phyla in color) and habitat (symbols) of the bacterial strains harboring representative Cas9 orthologs are indicated (right panel). The clusters grouping evolutionary distant bacteria (1 and 3) but isolated mainly from similar sources (human for cluster 1 and mostly environmental samples for cluster 3) suggest horizontal transfer of type II systems. Clusters 2, 4 and 5 group closely related bacteria isolated from diverse habitats indicating vertical transfer of the systems.

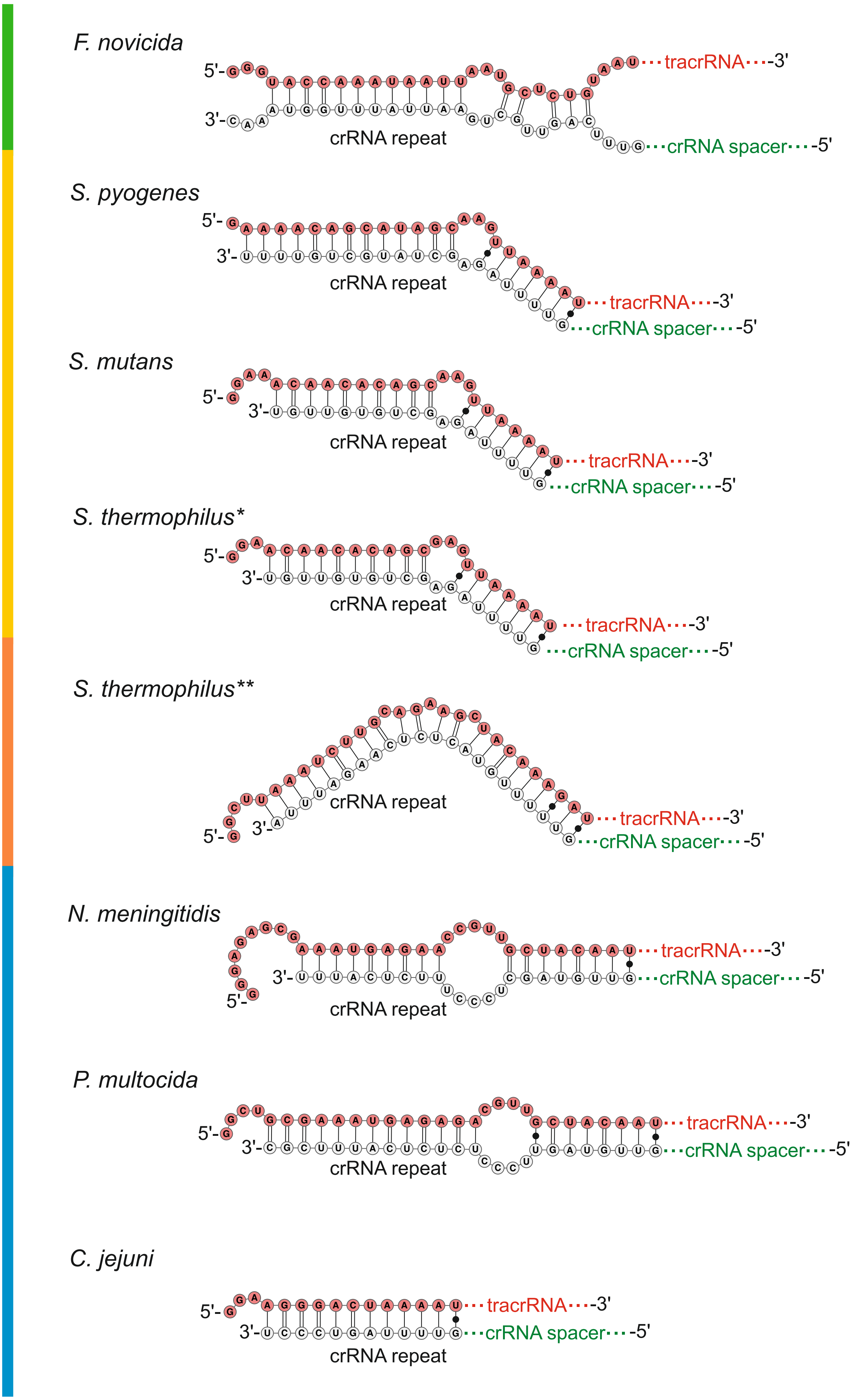
F. novicida
S. pyogenes
S. mutans
S. thermophilus*
S. thermophilus**
N. meningitidis
P. multocida
C. jejuni
tracrRNA
crRNA spacer
crRNA repeat

Supplementary Figure S12. tracrRNA:crRNA repeat duplexes form similar secondary structures in loci with closely related Cas9 orthologs. Antirepeat sequence of processed tracrRNA (red) and repeat-derived sequence of mature crRNA (grey) were co-folded for each type II CRISPR-Cas locus studied (see Materials and Methods). Color bars indicated on the left group dual-RNAs from loci with closely related Cas9 (see Figure 1 and Supplementary Figure S4). RNA duplexes belonging to the same groups display structural similarities, suggesting a role of the structure in dual-RNA recognition by Cas9.

Supplementary References

58. Sun, W., Li, G. & Nicholson, A. W. (2004) Mutational analysis of the nuclease domain of *Escherichia coli* ribonuclease III. Identification of conserved acidic residues that are important for catalytic function in vitro. *Biochemistry*, **43**, 13054-13062.

59. Sun, W., Jun, E. & Nicholson, A. W. (2001) Intrinsic double-stranded-RNA processing activity of *Escherichia coli* ribonuclease III lacking the dsRNA-binding domain. *Biochemistry*, **40**, 14976-14984.
